# Checklist of British and Irish Hymenoptera - aculeates (Apoidea, Chrysidoidea and Vespoidea)

**DOI:** 10.3897/BDJ.4.e8050

**Published:** 2016-04-07

**Authors:** George R. Else, Barry Bolton, Gavin R. Broad

**Affiliations:** ‡ Hayling Island, Portsmouth, United Kingdom; § c/o The Natural History Museum, London, United Kingdom; | The Natural History Museum, London, United Kingdom

**Keywords:** Britain, Ireland, bees, ants, wasps, fauna

## Abstract

**Background:**

The checklist of British and Irish aculeate Hymenoptera (Apoidea, Chrysidoidea and Vespoidea) is revised. Species distribution is summarised for all species at the level of country (England, Scotland, Wales, Ireland and Isle of Man).

**New information:**

The 601 native species represent an increase of 25 on the 1978 checklist, comprising mostly new discoveries. This increase is nearly balanced by the 23 species now presumed to be extinct in Britain and Ireland.

## Introduction

The checklist of British and Irish aculeates is essentially that of Else et al. (2004) but with several additions and updated taxonomy. This continues the series of chapters, starting with Broad and Livermore (2014a), Broad and Livermore (2014b) and Liston et al. (2014) comprising the first complete updating of the 1978 Hymenoptera checklist (Fitton et al. 1978).

Unlike most groups of Hymenoptera, the aculeate ants, bees and wasps are well-served by identification literature and are actively collected and recorded by a relatively large number of amateur as well as professional entomologists. We thus have a better idea of the actual size of the fauna than we do for sawflies and parasitoid wasps. There is thus a much better idea of change in the aculeate fauna and numerous extinctions and colonisations have been documented.

The aculeate Hymenoptera encompass three superfamilies and 18 families in Britain and Ireland (as discussed in Broad 2014, this figure is likely to change), with a wide range of ecologies and body forms. Figs 1, 2, 3, 4, 5, 6 depict some of this variety (and the website of the Bees, Wasps and Ants Recording Society has many more images). With multiple evolutions of eusociality, importance as pollinators, predators and parasitoids and general conspicuousness, the aculeates have always attracted attention and very good summaries of their biology can be found in a diverse array of texts for a variety of different audiences, from Lubbock (1882) to Gauld and Bolton (1988).

## Materials and methods

The rationale and background to the updated Hymenoptera checklist are laid out in Broad (2014). The aim is to account for all the names in the 1978 checklist, document all additions to and deletions from that list and to list the distributions of all species at the country level, i.e. England, Scotland, Wales, Ireland (as one unit) and the Isle of Man. We do not usually report distribution data for introduced species that only reproduce indoors or in greenhouses.

Sources of classification are listed under each superfamily. The checklist of bees is essentially similar to that of Else's (in prep.) handbook to the British fauna, except that we employ six families rather than including all bees in Apidae and, as discussed in the introduction to this checklist series (Broad 2014), we do not consider the fauna of the Channel Islands. We adopt a multi-family classification of the bees to maintain unity with larger-scale checklists (particularly that of M. Kuhlmann's Checklist of the Western Palaearctic Bees) and because this is the system employed in the most authoritative treatise on bee classification (Michener 2007), as well as most recent papers dealing with bee phylogeny.

Country-level distribution data mostly derive from the distribution atlases produced by the Bees, Wasps and Ants Recording Society (Edwards 1997, Edwards 1998, Edwards and Telfer 2001, Edwards and Telfer 2002, Edwards and Broad 2005, Edwards and Broad 2006, Edwards and Roy 2009, Collins and Roy 2012, with maps updated on their website), the catalogue of Irish Hymenoptera (O'Connor et al. 2009), and some additional literature references. Note that the Bethylidae, Dryinidae and Embolemidae are particularly poorly recorded.

The following abbreviations and conventions are employed:

[***species***] taxon deleted from the British and Irish list

NHM Natural History Museum, London

# known or suspected introductions with at least temporarily self-sustaining populations

? status (including uncertain synonymy) or identification in the British Isles uncertain

misident. has been misidentified as this name

nom. dub. *nomen dubium*, a name of unknown or doubtful application

nom. ob. *nomen oblitum*, ‘forgotten name’, does not have priority over a younger name

nom. nov. *nomen novum*, a replacement name

nom. nud. *nomen nudum*, an unavailable name, with no type specimen

preocc. name preoccupied (junior homonym)

stat. rev. *status revocatus*, revived status (e.g., raised from synonymy)

unavailable not meeting the requirements of the International Code of Zoological Nomenclature

var. variety, only available as a valid name under the provisions of article 45.6 of the ICZN

Text and spreadsheet versions of the checklists are available as supplementary files: Suppl. materials 1, 2.

## Checklists

### Apoidea

#### APOIDEA

Latreille, 1802

##### Notes

The higher-level classification of the Apoidea follows Pulawski (2013) Engel (2005) and M. Kuhlmann's online checklist of Western Palaearctic bees. The ‘traditional’ family Sphecidae is paraphyletic with respect to the bee families (here referred to as the Anthophila, an informal taxon, following Engel 2005) and in Britain and Ireland comprises the families Crabronidae and Sphecidae. Species-level classification of Crabronidae and Sphecidae follows Pulawski (2013). Figs 1, 2: habitus of selected British Apoidea.

#### Crabronidae

Latreille, 1802

#### Astatinae

Lepeletier, 1845

#### Astata

Latreille, 1796

DIMORPHA Panzer, 1806

#### Astata boops

(Schrank, 1781)

Sphex boops Schrank, 1781
abdominalis (Panzer, 1798, *Tiphia*)
pompiliformis (Panzer, 1804, *Larra*)
oculata (Jurine, 1807, *Dimorpha*)
victor Curtis, 1829
vanderlindeni Robert, 1833
agilis Smith, 1875

##### Distribution

England

#### Dryudella

Spinola, 1843

#### Dryudella pinguis

(Dahlbom, 1832)

Larra pinguis Dahlbom, 1832
stigma misident.
pinguis (Zetterstedt, 1838, *Larra*) preocc.
jaculator (Smith, 1845, *Astata*)

##### Distribution

England, Scotland, Wales

#### Bembicinae

Latreille, 1802

#### Alyssontini

Dalla Torre, 1897

#### Didineis

Wesmael, 1852

#### Didineis lunicornis

(Fabricius, 1798)

Pompilus lunicornis Fabricius, 1798
kenedii (Curtis, 1836, *Alyson*)

##### Distribution

England

#### Bembicini

Latreille, 1802

#### Argogorytes

Ashmead, 1899

#### Argogorytes fargeii

(Shuckard, 1837)

Gorytes fargeii Shuckard, 1837
campestris misident.
mongolensis Tsuneki, 1971
przewalskyi Kazenas, 1971

##### Distribution

England, Wales

#### Argogorytes mystaceus

(Linnaeus, 1761)

Sphex mystaceus Linnaeus, 1761
campestris (Linnaeus, 1761, *Vespa*)
inimica (Harris, 1776, *Vespa*)
longicornis (Rossi, 1790, *Sphex*)
bicinctus (Fabricius, 1793, *Crabro*)
arpactus (Fabricius, 1804, *Mellinus*)
flavicincta (Donovan, 1808, *Vespa*)
croceipes (Eversmann, 1849, *Gorytes*)
tonsus (Bondroit, 1933, *Gorytes*)

##### Distribution

England, Scotland, Wales, Ireland

#### Gorytes

Latreille, 1805

ARPACTUS Panzer, 1805
EUZONIA Stephens, 1829
EUSPONGUS Lepeletier, 1832
HOPLISUS Lepeletier, 1832
LAEVIGORYTES Zavadil, 1948

#### Gorytes laticinctus

(Lepeletier, 1832)

Euspongus laticinctus Lepeletier, 1832

##### Distribution

England, Wales

#### Gorytes quadrifasciatus

(Fabricius, 1804)

Mellinus quadrifasciatus Fabricius, 1804
vicinus (Lepeletier, 1832, *Euspongus*)
montivagus (Mocsáry, 1878, *Hoplisus*)

##### Distribution

England, Wales

#### Harpactus

Shuckard, 1837

ARPACTUS Jurine, 1807
HARPACTES Dahlbom, 1843
DIENOPLUS Fox, 1893

#### Harpactus tumidus

(Panzer, 1801)

Pompilus tumidus Panzer, 1801
japonensis (Tsuneki, 1963, *Dienoplus*)
transiliensis Kazenas, 1989

##### Distribution

England, Scotland, Wales, Ireland, Isle of Man

#### Lestiphorus

Lepeletier, 1832

HYPOMELLINUS Asmead, 1899
MELLINOGASTRA Asmead, 1899

#### Lestiphorus bicinctus

(Rossi, 1794)

Crabro bicinctus Rossi, 1794

##### Distribution

England

#### Nyssonini

Latreille, 1804

#### Nysson

Latreille, 1796

SYNNEVRUS Costa, 1859

#### Nysson dimidiatus

Jurine, 1807

wesmaeli Lepeletier, 1845
distinguendus Chevrier, 1867

##### Distribution

England, Wales

#### Nysson interruptus

(Fabricius, 1798)

Mellinus interruptus Fabricius, 1798
spinosus (Fabricius, 1804, *Ceropales*)
panzeri Lepeletier, 1845
shukardi Wesmael, 1852

##### Distribution

England

#### Nysson spinosus

(Forster, 1771)

Sphex spinosus Forster, 1771
bidens (Linnaeus, 1767, *Vespa*)
spinosus (Fabricius, 1775, *Crabro*) preocc.
tricinctus (Fabricius, 1793, *Mellinus*)
trilineata (Turton, 1802, *Vespa*)
geniculatus Lepeletier, 1845
malasei Gussakovskij, 1932

##### Distribution

England, Scotland, Wales, Ireland

#### Nysson trimaculatus

(Rossi, 1790)

Crabro trimaculatus Rossi, 1790

##### Distribution

England, Wales

#### Crabroninae

Latreille, 1802

#### Crabronini

Latreille, 1802

#### Crabro

Fabricius, 1775

THYREOPUS Lepeletier & Brullé, 1835
ANOTHYREUS Dahlbom, 1845
THYREOCNEMUS Costa, 1871
PARANOTHYREUS Ashmead, 1899
SYNOTHYREOPUS Ashmead, 1899
AGNOSICRABRO Pate, 1944
DYSCOLOCRABRO Pate, 1944
HEMITHYREOPUS Pate, 1944
NORUMBEGA Pate, 1947
PARANOTHYREUS Pate, 1944
PARATHYREOPUS Pate, 1944
PEMPHILIS Pate, 1944
OTHYREUS Marshakov, 1977

#### Crabro cribrarius

(Linnaeus, 1758)

Vespa cribraria Linnaeus, 1758
patellarius (Schreber, 1784, *Sphex*)
argus (Christ, 1791, *Sphex*)
longa (Christ, 1791, *Sphex*)
lunatus (Christ, 1791, *Sphex*)
palmatus Panzer, 1797
inornatus Mocsáry, 1901
hypotheticus Kokujev, 1927

##### Distribution

England, Scotland, Wales, Isle of Man

#### Crabro peltarius

(Schreber, 1784)

Sphex peltarius Schreber, 1784
patellatus Panzer, 1797
dentipes Panzer, 1797
mediatus Fabricius, 1798

##### Distribution

England, Scotland, Wales, Ireland, Isle of Man

#### Crabro scutellatus

(von Scheven, 1781)

Sphex scutellatus von Scheven, 1781
scutularius (Schreber, 1784, *Sphex*)
pterotus Panzer, 1801
reticulatus (Lepeletier & Brullé, 1835, *Ceratocolus*)

##### Distribution

England

#### Crossocerus

Lepeletier & Brullé, 1835

#### Ablepharipus

Perkins, 1913

#### Crossocerus (Ablepharipus) congener

(Dahlbom, 1844)

Crabro congener Dahlbom, 1844

##### Distribution

England

##### Notes

added by Archer (2007) based on a pers. comm. from D. Baldock

#### Crossocerus (Ablepharipus) podagricus

(Vander Linden, 1829)

Crabro podagricus Vander Linden, 1829
vicinus Dahlbom, 1842
punctata Šnoflak, 1948
snoflaki Zavadil, 1948

##### Distribution

England, Wales

#### Acanthocrabro

Perkins, 1913

CORENOCRABRO Tsuneki, 1974

#### Crossocerus (Acanthocrabro) vagabundus

(Panzer, 1798)

Crabro vagabundus Panzer, 1798
varus (Panzer, 1799, *Crabro*) preocc.
bojus (Schrank, 1802, *Crabro*)
quinquemaculatus (Lepeletier & Brullé, 1835, *Blepharipus*)
lefebvrei Lepeletier & Brullé, 1835
fasciatus (Costa, 1871, *Crabro*)
esakii (Yasumatsu, 1942, *Crabro*)
ectemiformis (Tsuneki, 1974, *Corenocrabro*)

##### Distribution

England, Wales

#### Blepharipus

Lepeletier & Brullé, 1835

COELOCRABRO Thomson, 1874

#### Crossocerus (Blepharipus) annulipes

(Lepeletier & Brullé, 1835)

Blepharipus annulipes Lepeletier & Brullé, 1835
gonager (Lepeletier & Brullé, 1835, *Blepharipus*)
nigritus (Gimmerthal, 1836, *Crabro*)
ambiguus (Dahlbom, 1842, *Crabro*)
capito (Dahlbom, 1845, *Crabro*)
parkeri (Banks, 1921, *Blepharipus*)
davidsoni (Sandhouse, 1938, *Crabro*)

##### Distribution

England, Scotland, Wales

#### Crossocerus (Blepharipus) capitosus

(Shuckard, 1837)

Crabro capitosus Shuckard, 1837
annulus (Dahlbom, 1838, *Crabro*)
yezo Tsuneki, 1960

##### Distribution

England, Scotland, Wales, Ireland

#### Crossocerus (Blepharipus) cetratus

(Shuckard, 1837)

Crabro cetratus Shuckard, 1837
vanderlindeni (Dahlbom, 1838, *Crabro*)
dilatatus (Herrich-Schäffer, 1841, *Crabro*)
inornatus (Matsumura, 1912, *Crabro*)
dentsukanus Tsuneki, 1976

##### Distribution

England, Wales, Ireland

#### Crossocerus (Blepharipus) leucostomus

(Linnaeus, 1758)

Sphex leucostoma Linnaeus, 1758
carbonarius (Dahlbom, 1838, *Crabro*)
rugosus (Herrich-Schäffer, 1841, *Crabro*)
melanarius (Wesmael, 1852, *Crabro*)
cinctipes (Provancher, 1882, *Blepharipus*)
niger (Provancher, 1888, *Crabro*)
nigror (Fox, 1895, *Crabro*)
servus (Dalla Torre, 1897, *Crabro*)
cinctitarsis (Ashmead, 1901, *Stenocrabro*)
columbiae (Bradley, 1906, *Blepharipus*)
stygius (Mickel, 1916, *Thyreopus*)
utensis (Mickel, 1916, *Thyreopus*)

##### Distribution

England, Scotland

#### Crossocerus (Blepharipus) megacephalus

(Rossi, 1790)

Crabro megacephalus Rossi, 1790
leucostoma misident.
bidens (Haliday, 1833, *Crabro*)
niger Lepeletier & Brullé, 1835
rufipes Lepeletier & Brullé, 1835
laeviceps (Smith, 1856, *Crabro*)
bison (Costa, 1884, *Crabro*)
zaidamensis (Radoszkowski, 1887, *Crabro*)
leucostomoides (Richards, 1935, *Coelocrabro*)

##### Distribution

England, Scotland, Wales, Ireland, Isle of Man

#### Crossocerus (Blepharipus) nigritus

(Lepeletier & Brullé, 1835)

Blepharipus nigritus Lepeletier & Brullé, 1835
pubescens (Shuckard, 1837, *Crabro*)
diversipes Herrich-Schäffer, 1841
inermis (Thomson, 1870, *Crabro*)
melanogaster Kohl, 1880
nigricornis (Provancher, 1888, *Blepharipus*)
sambucicola (Verhoeff, 1891, *Crabro*)
verhoeffi Tsuneki, 1967
sudai Tsuneki, 1976
babai Tsuneki, 1979

##### Distribution

England, Wales

#### Crossocerus (Blepharipus) styrius

(Kohl, 1892)

Crabro styrius Kohl, 1892
pauxillus (Gussakovskij, 1932, *Crabro*)
sugiharai (Iwata, 1938, *Crabro*)
pilicornis Tsuneki, 1977

##### Distribution

England

#### Crossocerus (Blepharipus) walkeri

(Shuckard, 1837)

Crabro walkeri Shuckard, 1837
aphidium misident.
geniculatus (Shuckard, 1837, *Crabro*)
clypearis (Schenck, 1857, *Crabro*)
cloevorax (Nielson, 1901, *Crabro*)

##### Distribution

England, Scotland, Wales

#### Crossocerus

Lepeletier & Brullé, 1835

STENOCRABRO Ashmead, 1899
ISCHNOLYNTHUS Holmberg, 1903

#### Crossocerus (Crossocerus) distinguendus

(Morawitz, 1866)

Crabro distinguendus Morawitz, 1866
mucronatus (Thomson, 1870, *Crabro*)

##### Distribution

England

##### Notes

added by Packer (1981)

#### Crossocerus (Crossocerus) elongatulus

(Vander Linden, 1829)

Crabro elongatulus Vander Linden, Crabro
annulatus Lepeletier & Brullé, 1835
varipes Lepeletier & Brullé, 1835
affinis Lepeletier & Brullé, 1835
luteipalpis Lepeletier & Brullé, 1835
morio Lepeletier & Brullé, 1835
pallidipalpis Lepeletier & Brullé, 1835
proximus (Shuckard, 1837, *Crabro*)
hyalinus (Shuckard, 1837, *Crabro*)
transversalis (Shuckard, 1837, *Crabro*)
obliquus (Shuckard, 1837, *Crabro*)
propinquus (Shuckard, 1837, *Crabro*)
brevis (Eversmann, 1849, *Crabro*)
scutellaris (Smith, 1851, *Crabro*)
sulcus (Fox, 1895, *Crabro*)
plesius (Rohwer, 1912, *Stenocrabro*)
berlandi (Richards, 1928, *Crabro*)

##### Distribution

England, Scotland, Wales, Isle of Man

##### Notes

The British population is considered to belong to the subspecies *annulatus* Lepeletier & Brullé (synonyms: *proximus, hyalinus, transversalis, obliquus, propinquus, berlandi*).

#### Crossocerus (Crossocerus) exiguus

(Vander Linden, 1829)

Crabro exiguus Vander Linden, Crabro
aphidum Lepeletier & Brullé, 1835

##### Distribution

England

#### Crossocerus (Crossocerus) ovalis

Lepeletier & Brullé, 1835

punctum (Zetterstedt, 1838, *Crabro*)
anxius (Wesmael, 1852, *Crabro*)
shuckardi (Smith, 1856, *Crabro*)
ovatus (Schulz, 1906, *Crabro*)

##### Distribution

England, Scotland, Wales

#### Crossocerus (Crossocerus) palmipes

(Linnaeus, 1767)

Sphex palmipes Linnaeus, 1767
palmarius (Schreber, 1784, *Sphex*)
scutatus (Fabricius, 1787, *Crabro*)
ornatus Lepeletier & Brullé, 1835
scutellaris (Gimmerthal, 1836, *Crabro*)
gracilis (Eversmann, 1849, *Crabro*)
decoratus (Smith, 1856, *Crabro*)

##### Distribution

England, Scotland, Isle of Man

#### Crossocerus (Crossocerus) tarsatus

(Shuckard, 1837)

Crabro tarsatus Shuckard, 1837
palmipes misident.
palmatus De Stefani Perez, 1884 preocc,

##### Distribution

England, Scotland, Wales, Ireland, Isle of Man

#### Crossocerus (Crossocerus) varus

Lepeletier & Brullé, 1835 nomen protectum

varius misspelling
pusillus Lepeletier & Brullé, 1835
striatulus Lepeletier & Brullé, 1835
spinipectus (Shuckard, 1837, *Crabro*)
striatus Lepeletier, 1845
intricatus (Smith, 1856, *Crabro*)
lepeletieri (Smith, 1856, *Crabro*)

##### Distribution

England, Scotland, Wales, Ireland, Isle of Man

#### Crossocerus (Crossocerus) wesmaeli

(Vander Linden, 1829)

Crabro wesmaeli Vander Linden, Crabro
maurus (Lepeletier & Brullé, 1835, *Ceratocolus*)
ziegleri (Lepeletier & Brullé, 1835, *Ceratocolus*)

##### Distribution

England, Scotland, Wales, Ireland

#### Cuphopterus

Morawitz, 1866

#### Crossocerus (Cuphopterus) binotatus

Lepeletier & Brullé, 1835

signatus (Panzer, 1798, *Crabro*) preocc.
monstrosus (Dahlbom, 1845, *Crabro*)
confusus (Schulz, 1906, *Crabro*)

##### Distribution

England, Wales

#### Crossocerus (Cuphopterus) dimidiatus

(Fabricius, 1781)

Crabro dimidiatus Fabricius, 1781
subpunctatus (Rossi, 1790, *Crabro*)
sexmaculatus (Olivier, 1792, *Crabro*)
signatus (Olivier, 1792, *Crabro*)
serripes (Panzer, 1797, *Crabro*)
notatus (Illiger, 1807, *Crabro*)
pauperatus (Lepeletier & Brullé, 1835, *Blepharipus*)
armipes (von Sebold, 1844, *Crabro*)

##### Distribution

England, Scotland, Wales, Ireland

#### Hoplocrabro

Thomson, 1874

#### Crossocerus (Hoplocrabro) quadrimaculatus

(Fabricius, 1793)

Crabro quadrimaculatus Fabricius, 1793
quadripunctatus (Fabricius, 1793, *Crabro*)
murorum (Latreille, 1805, *Crabro*)
levipes Vander Linden, 1829, *Crabro*)
bimaculatus Lepeletier & Brullé, 1835
quniquemaculatus (Dahlbom, 1838, *Crabro*)
rotundarius (Dahlbom, 1838, *Crabro*)

##### Distribution

England, Scotland, Wales, Ireland

#### Ectemnius

Dahlbom, 1845

#### Clytochrysus

Morawitz, 1864

#### Ectemnius (Clytochrysus) cavifrons

(Thomson, 1870)

Crabro cavifrons Thomson, 1870
cephalotes misident.

##### Distribution

England, Scotland, Wales, Ireland, Isle of Man

#### Ectemnius (Clytochrysus) lapidarius

(Panzer, 1804)

Crabro lapidarius Panzer, 1804
cinctus (Spinola, 1806, *Crabro*)
chrysostomus (Lepeletier & Brullé, 1835, *Crabro*)
comptus (Lepeletier & Brullé, 1835, *Crabro*)
xylurgus (Shuckard, 1837, *Crabro*)
interstinctus (Smith, 1856, *Crabro*)
obscurus (Smith, 1856, *Crabro*)
gracilissimus (Packard, 1866, *Crabro*)
denticulatus (Packard, 1866, *Crabro*)
effosus (Packard, 1866, *Crabro*)
papagorum (Viereck, 1908, *Crabro*)

##### Distribution

England, Scotland, Wales, Ireland, Isle of Man

#### Ectemnius (Clytochrysus) ruficornis

(Zetterstedt, 1838)

Crabro ruficornis Zetterstedt, 1838
aurilabris (Herrich-Schäffer, 1841, *Crabro*)
nigrifrons (Cresson, 1865, *Crabro*)
contiguus (Cresson, 1865, *Crabro*)
septentrionalis (Packard, 1866, *Crabro*)
planifrons (Thomson, 1870, *Crabro*)
longipalpis (Verhöff, 1892, *Crabro*)
lineatotarsis (Matsumura, 1911, *Crabro*)
chipsani (Matsumura, 1912, *Crabro*)

##### Distribution

England, Wales

#### Ectemnius (Clytochrysus) sexcinctus

(Fabricius, 1775)

Crabro sexcinctus Fabricius, 1775
planifrons misident.
quadricinctus (Fabricius, 1787, *Crabro*)
interruptefasciatus (Retzius, 1783, *Sphex*)
tibialis (Olivier, 1792, *Crabro*)
maculata (Preyssler, 1793, *Crabro*)
zonatus (Panzer, 1797, *Crabro*)
vespiformis (Panzer, 1798, *Crabro*)
octomaculatus (Schrank, 1802, *Crabro*)
cinctus (Spinola, 1806, *Crabro*)
flavipes (Lepeletier & Brullé, 1835, *Crabro*)
tetraedrus (Blanchard, 1940, *Crabro*)
saundersi (Perkins, 1899, *Crabro*)
yosemite Pate, 1946

##### Distribution

England, Wales

#### Ectemnius

Dahlbom, 1845

#### Ectemnius (Ectemnius) borealis

(Zetterstedt, 1838)

Crabro borealis Zetterstedt, 1838
bipunctatus (Zetterstedt, 1838, *Crabro*)
nigrinus (Herrich-Schäffer, 1841, *Crabro*)
parvulus (Packard, 1866, *Crabro*)
gredleri (Kohl, 1878, *Lindenius*)
proletarius (Mickel, 1916, *Crabro*)

##### Distribution

England

#### Ectemnius (Ectemnius) dives

(Lepeletier & Brullé, 1835)

Solenius dives Lepeletier & Brullé, 1835
octonotatus (Lepeletier & Brullé, 1835, *Solenius*)
octavonotatus (Lepeletier & Brullé, 1835, *Solenius*)
alatulus (Dahlbom, 1838, *Crabro*)
pictipes (Herrich-Schäffer, 1841, *Crabro*)
auratus (Smith, 1856, *Crabro*)
montanus (Cresson, 1865, *Crabro*) preocc.
cristatus (Packard, 1866, *Crabro*)
cubiceps (Packard, 1866, *Crabro*)
heraclei (Rohwer, 1908, *Crabro*)
montivagans (Strand, 1916, *Crabro*)

##### Distribution

England, Wales

#### Hypocrabro

Ashmead, 1899

XESTOCRABRO Ashmead, 1899

#### Ectemnius (Hypocrabro) continuus

(Fabricius, 1804)

Crabro continuus Fabricius, 1804
vagus misident.
sexmaculatus (Say, 1824, *Crabro*) preocc.
fuscitarsis (Herrich-Schäffer, 1841, *Crabro*)
vagatus (Smith, 1869, *Crabro*)
pumilus (Costa, 1871, *Crabro*)
granulatus (Walker, 1871, *Crabro*)
rugopunctatus (Taschenberg, 1875, *Crabro*)
validus (De Stefani Perez, 1884, *Crabro*)
bizexmaculatus (Viereck, 1910, *Crabro*)
sayi (Cockerell, 1910, *Crabro*)
giffardi (Rohwer, 1917, *Solenius*)

##### Distribution

England, Scotland, Wales, Ireland

#### Ectemnius (Hypocrabro) rubicola

(Dufour & Perris, 184)

Solenius rubicola Dufour & Perris, 1840
microstictus (Herrich-Schäffer, 1841, *Crabro*)
larvatus (Wesmael, 1852, *Crabro*)
pumilus (Costa, 1871, *Crabro*)

##### Distribution

England, Wales

#### Metacrabro

Ashmead, 1899

#### Ectemnius (Metacrabro) cephalotes

(Olivier, 1792)

Crabro cephalotes Olivier, 1792
quadricinctus misident.
floralis (Olivier, 1792, *Crabro*)
geniculatus (Olivier, 1792, *Crabro*)
tibialis (Olivier, 1792, *Crabro*)
cephalotes (Panzer, 1799, *Crabro*)
striatus (Lepeletier & Brullé, 1835, *Crabro*)
ornatus (Lepeletier & Brullé, 1835, *Crabro*)
striatulus (Lepeletier & Brullé, 1835, *Blepharipus*)
lindenius (Shuckard, 1837, *Crabro*)
shuckardi (Dahlbom, 1838, *Crabro*)
interruptus (Dahlbom, 1845, *Crabro*) preocc.
fargeii (Smith, 1856, *Crabro*)
aciculatus (Provancher, 1882, *Crabro*)
ruthenicus (Morawitz, 1892, *Crabro*)

##### Distribution

England, Scotland, Wales, Ireland

#### Ectemnius (Metacrabro) lituratus

(Panzer, 1805)

Crabro lituratus Panzer, 1805
petiolatus (Lepeletier & Brullé, 1835, *Solenius*)
fasciatus (Lepeletier & Brullé, 1835, *Ceratocolus*)
reticulatus (Lepeletier & Brullé, 1835, *Ceratoculus* [lapsus])
kollari (Dahlbom, 1845, *Crabro*)
argenteus (Schenck, 1857, *Crabro*)
vestitus (Smith, 1858, *Crabro*)
intermedius (Morawitz, 1866, *Crabro*)
luxuriosus (Costa, 1871, *Crabro*)

##### Distribution

England, Wales

#### Entomognathus

Dahlbom, 1844

KOXINGA Pate, 1944
MASHONA Pate, 1944
TONCAHUA Pate, 1944
FLORKINUS Leclercq, 1956
BIHARGNATHUS Leclercq, 1977

#### Entomognathus brevis

(Vander Linden, 1829)

Crabro brevis Vander Linden, 1829
apicalis (Lepeletier & Brullé, 1835, *Lindenius*)
nasutus (Gribodo, 1884, *Lindenius*)

##### Distribution

England, Wales

#### Lestica

Billberg, 1820

#### Clypeocrabro

Richards, 1935

THYREUS Lepeletier & Brullé, 1835 preocc.

#### Lestica (Clypeocrabro) clypeata

(Schreber, 1759)

Apis clypeata Schreber, 1759
ovata (Christ, 1791, *Sphex*)
vexillata (Panzer, 1797, *Crabro*)
lapidaria (Fabricius, 1804, *Crabro*) preocc.
clypeata (Thunberg, 1815, *Philanthus*)
nigridens (Herrich-Schäffer, 1841, *Crabro*)
quadrifer (Dufour, 1841, *Crabro*)

##### Distribution

England

#### Lindenius

Lepeletier & Brullé, 1835

CHALCOLAMPRUS Wesmael, 1852
TRACHELOSIMUS Morawitz, 1866

#### Lindenius albilabris

(Fabricius, 1793)

Crabro albilabris Fabricius, 1793
aenescens (Dahlbom, 1838, *Crabro*)

##### Distribution

England, Scotland, Wales

#### Lindenius panzeri

(Vander Linden, 1829)

Crabro panzeri Vander Linden, 1829
venustus Lepeletier & Brullé, 1835
latebrosus (Kohl, 1905, *Crabro*)
harbinensis Tsuneki, 1967
mongolicus Tsuneki, 1972

##### Distribution

England

#### Lindenius pygmaeus

(Rossi, 1794)

Crabro pygmaeus Rossi, 1794
curtus Lepeletier & Brullé, 1835
kratochvili (Šnoflak, 1948, *Crabro*)

##### Distribution

England

##### Notes

Represented by the subspecies *armatus* (Vander Linden, 1829, *Crabro*).

#### Rhopalum

Stephens, 1829

#### Rhopalum

Stephens, 1829

EUPLILIS Risso, 1826 nom. ob.
PHYSOSCELUS Lepeletier & Brullé, 1835

#### Rhopalum (Rhopalum) clavipes

(Linnaeus, 1758)

Sphex clavipes Linnaeus, 1758
rufiventris (Panzer, 1799, *Crabro*)

##### Distribution

England, Scotland, Wales, Ireland, Isle of Man

#### Corynopus

Lepeletier & Brullé, 1835

DRYPHUS Herrich-Schäffer, 1840
ALLIOGNATHUS Ashmead, 1899

#### Rhopalum (Corynopus) coarctatum

(Scopoli, 1763)

Sphex coarctata Scopoli, 1763
crassipes (Fabricius, 1798, *Crabro*)
tibiale (Fabricius, 1798, *Crabro*) preocc.
modestum Rohwer, 1908

##### Distribution

England, Scotland, Wales, Ireland

#### Rhopalum (Corynopus) gracile

Wesmael, 1852

nigrinum Kiesenwetter, 1849 preocc.
kiesenwetteri (Morawitz, 1866, *Crabro*)
simplicipes (Morawitz, 1888, *Corynopus*)

##### Distribution

England

#### Larrini

Latreille, 1810

#### Tachysphex

Kohl, 1883

SCHISTOSPHEX Arnold, 1922
ATELOSPHEX Arnold, 1923

#### Tachysphex nitidus

(Spinola, 1805)

Astata nitida Spinola, 1805
unicolor misident.
ibericus (de Saussure, 1867, *Tachytes*)

##### Distribution

England, Wales, Isle of Man

##### Notes

Added by Edwards (1998); earlier records of *unicolor* mostly refer to *nitidus*.

#### Tachysphex obscuripennis

(Schenck, 1857)

Tachytes obscuripennis Schenck, 1857
lativalvis (Thomson, 1870, *Tachytes*)

##### Distribution

England

##### Notes

Only recorded as a vagrant in Deal, Kent; resident on the Channel Isles, which lie outside of the geographical coverage of this checklist.

#### Tachysphex pompiliformis

(Panzer, 1805)

Larra pompiliformis Panzer, 1805
pectinipes misident.
nigripennis (Spinola, 1808, *Tachytes*)
dimidiata (Panzer, 1809, *Larra*)
jokischiana (Panzer, 1809, *Larra*)
parvula (Cresson, 1865, *Larrada*)
quebecensis (Provancher, 1882, *Larra*)
decorus Fox, 1894
tenuipunctus Fox, 1894
consimilis Fox, 1894
rufoniger Bingham, 1897
projectus Nurse, 1903
argyrotrichus Rohwer, 1911
granulosus Mickel, 1916
erythraeus Mickel, 1916
angularis Mickel, 1916

##### Distribution

England, Scotland, Wales, Ireland, Isle of Man

#### Tachysphex unicolor

(Panzer, 1809)

Larra unicolor Panzer, 1809
nitidus misident.
jurinii (Drapiez, 1819, *Larra*)

##### Distribution

England

#### Miscophini

Fox, 1894

#### Miscophus

Jurine, 1807

NITELOPTERUS Ashmead, 1897
HYPOMISCOPHUS Cockerell, 1898
MISCOPHINUS Ashmead, 1898

#### Miscophus ater

Lepeletier, 1845

maritimus Smith, 1858

##### Distribution

England

#### Miscophus bicolor

Jurine, 1807

dubius (Panzer, 1809, *Larra*)
metallicus Verhöff, 1890
tsunekii de Andrade, 1960

##### Distribution

England

##### Notes

added by Knowles and Else (2006)

#### Miscophus concolor

Dahlbom, 1844

bicolor misident.
moravicus Balthazar, 1957

##### Distribution

England

#### Nitela

Latreille, 1809

TENILA Brèthes, 1913
RHINONITELA Williams, 1928

#### Nitela borealis

Valkeila, 1974

##### Distribution

England

##### Notes

added by Felton (1987)

#### Nitela lucens

Gayubo & Felton, 2000

spinolae misident.

##### Distribution

England

##### Notes

Added by Felton (1987), initially as *Nitela spinolae* Latreille, 1809 but described later as a new species (Gayubo and Felton 2000).

#### Oxybelini

Leach, 1815

#### Oxybelus

Latreille, 1796

NOTOGLOSSA Dahlbom, 1845
ALEPIDASPIS Costa, 1882
ANOXYBELUS Kohl, 1924
GONIOXYBELUS Pate, 1937
ORTHOXYBELUS Pate, 1937
LATROXYBELUS Noskiewicz & Chudoba, 1950

#### Oxybelus argentatus

Curtis, 1833

mucronatus misident.
decimmaculata (Donovan, 1806, *Vespa*)
ferox Shuckard, 1837
nigricornis Shuckard, 1837

##### Distribution

England, Wales

#### Oxybelus mandibularis

Dahlbom, 1845

sericatus Gerstäcker, 1867

##### Distribution

England, Scotland, Wales, Isle of Man

#### Oxybelus uniglumis

(Linnaeus, 1758)

Vespa uniglumis Linnaeus, 1758
punctata (Fabricius, 1793, *Nomada*)
tridens (Fabricius, 1798, *Crabro*)
decimmaculata (Donovan, 1806, *Vespa*)
pygmaeus Olivier, 1812
quadrinotatus Say, 1824
impatiens Smith, 1856
interruptus Cresson, 1865
fallax Gerstäcker, 1867
brodiei Provancher, 1883
hispanicus Giner Marí, 1943

##### Distribution

England, Scotland, Wales, Ireland, Isle of Man

#### Trypoxylini

Lepeletier, 1845

#### Trypoxylon

Latreille, 1796

APIUS Panzer, 1806
APIUS Jurine, 1807
TRYPARGILUM Richards, 1934
ASACONOTON Arnold, 1959

#### Trypoxylon attenuatum

Smith, 1851

##### Distribution

England, Scotland, Wales, Ireland

#### Trypoxylon clavicerum

Lepeletier & Serville, 1828

tibiale Zetterstedt, 1840
batumicum Antropov, 1985

##### Distribution

England, Wales, Ireland

#### Trypoxylon figulus

(Linnaeus, 1758)

Sphex figulus Linnaeus, 1758
fuliginosa (Scopoli, 1763, *Sphex*)
apicale Fox, 1891
yezo Tsuneki, 1956
barbarum de Beaumont, 1957
fieuzeti Giner Marí, 1959

##### Distribution

England, Wales

#### Trypoxylon medium

de Beaumont, 1945

##### Distribution

England, Scotland, Wales

##### Notes

added by Pulawski (1984)

#### Trypoxylon minus

de Beaumont, 1945

koma Tsuneki, 1956

##### Distribution

England

##### Notes

added by Felton (1988)

#### Dinetinae

Fox, 1895

#### Dinetus

Panzer, 1806

DINETUS Jurine, 1807 preocc.

#### Dinetus pictus

(Fabricius, 1793)

Crabro pictus Fabricius, 1793
guttatus (Fabricius, 1793, *Sphex*) preocc.

##### Distribution

England

#### Mellininae

Latreille, 1802

#### Mellinus

Fabricius, 1790

#### Mellinus arvensis

(Linnaeus, 1758)

Vespa arvensis Linnaeus, 1758
vagus (Linnaeus, 1758, *Sphex*)
superbus (Harris, 1776, *Vespa*)
tricinctus (Schrank, 1781, *Vespa*)
clavatus (Retzius, 1783, *Sphex*)
infundibuliformis (Geoffroy, 1785, *Vespa*)
petiolatus (Geoffroy, 1785, *Vespa*)
bipunctatus (Fabricius, 1787, *Crabro*)
gibbus (Villers, 1789, *Sphex*) preocc.
melanostictus (Gmelin, 1790, *Vespa*)
arthriticus (Rossi, 1790, *Crabro*)
rachiticus (Rossi, 1790, *Crabro*)
annularis (Christ, 1791, *Sphex*)
succinctus (Olivier, 1792, *Vespa*)
diversus (Olivier, 1792, *Vespa*)
labiatus (Olivier, 1792, *Crabro*)
quinquemaculatus (Fabricius, 1793, *Philanthus*)
u-flavum (Panzer, 1794, *Crabro*)
capistratus (Schrank, 1796, *Crabro*)
pratensis Jurine, 1807
compactus Handlirsch, 1888

##### Distribution

England, Scotland, Wales, Ireland, Isle of Man

#### Mellinus crabroneus

(Thunberg, 1791)

Sphex crabronea Thunberg, 1791
sabulosus (Fabricius, 1787, *Crabro*) preocc.
ruficornis (Villers, 1789, *Sphex*) preocc.
sabulosus (Olivier, 1792, *Crabro*) preocc.
ruficornis Fabricius, 1793 preocc.
frontalis (Panzer, 1797, *Crabro*)
petiolatus (Panzer, 1797, *Crabro*)
fulvicornis Fabricius, 1804

##### Distribution

England, Wales

#### Pemphredoninae

Dahlbom, 1835

#### Pemphredonini

Dahlbom, 1835

#### Diodontus

Curtis, 1834

XYLOCELIA Rohwer, 1915
NEODIODONTUS Tsuneki, 1972
CORENIUS Tsuneki, 1974

#### Diodontus insidiosus

Spooner, 1938

friesei misident.

##### Distribution

England

#### Diodontus luperus

Shuckard, 1837

##### Distribution

England, Wales

#### Diodontus minutus

(Fabricius, 1793)

Crabro minutus Fabricius, 1793
franclemonti Krombein, 1939

##### Distribution

England, Wales, Isle of Man

#### Diodontus tristis

(Vander Linden, 1829)

Pemphredon tristis Vander Linden, 1829

##### Distribution

England, Scotland, Wales

#### Passaloecus

Shuckard, 1837

XYLOECUS Shuckard, 1837
COELOECUS Verhöff, 1890
HEROECUS Verhöff, 1890

#### Passaloecus clypealis

Faester, 1947

angustus Gussakovskij, 1952

##### Distribution

England

#### Passaloecus corniger

Shuckard, 1837

##### Distribution

England, Wales

#### Passaloecus eremita

Kohl, 1893

##### Distribution

England

##### Notes

added by Richards (1980)

#### Passaloecus gracilis

(Curtis, 1834)

Diodontus gracilis Curtis, 1834
insignis misident.
turionum misident.
brevicornis Morawitz, 1864

##### Distribution

England

#### Passaloecus insignis

(Vander Linden, 1829)

Pemphredon insignis Vander Linden, 1829
roettgeni Verhöff, 1890
shuckardi Yasumatsu, 1934

##### Distribution

England, Wales

#### Passaloecus monilicornis

Dahlbom, 1842

##### Distribution

England, Scotland, Wales, Ireland

#### Passaloecus singularis

Dahlbom, 1844

gracilis misident.
tenuis Morawitz, 1864
gertrudis Krombein, 1938

##### Distribution

England, Wales

#### Passaloecus turionum

Dahlbom, 1844

##### Distribution

England

##### Notes

added by Guichard (2002)

#### Pemphredon

Latreille, 1796

CEMONUS Panzer, 1806
DINEURUS Westwood, 1837
CERATOPHORUS Shuckard, 1837
DIPHLEBUS Westwood, 1840
CHEVRIERIA Kohl, 1883
SUSANOWO Tsuneki, 1972

##### Notes

Taxonomy follows Dollfuss (1995).

#### Pemphredon austriaca

(Kohl, 1888)

Diphlebus austriacus Kohl, 1888
enslini misident.
coracina Valkeila, 1972
tener Valkeila, 1972
nescia Merisuo, 1972

##### Distribution

England

##### Notes

Richards (1980) refers to this species as *Pemphredon enslini* (Wagner, 1932, *Dineurus*). There has been much confusion over the identities of these taxa (see Knowles in Collins and Roy 2012) but Pulawski (2013) treats *austriaca* and *enslini* as separate species.

#### Pemphredon inornata

Say, 1824

shuckardi (Morawitz, 1864, *Cemonus*)
dentata (Puton, 1871, *Cemonus*)
tenax Fox, 1829

##### Distribution

England, Scotland, Wales, Ireland

#### Pemphredon lethifer

(Shuckard, 1837)

Cemonus lethifer Shuckard, 1837
lethifera misspelling
austriaca misident.
strigatus (Chevrier, 1870, *Cemonus*)
fabricii (Müller, 1911, *Cemonus*)
fuscatus (Wagner, 1918, *Diphlebus*)
littoralis (Wagner, 1918, *Diphlebus*)
neglectus (Wagner, 1918, *Diphlebus*)
minutus (Wagner, 1918, *Diphlebus*)
enslini Wagner, 1931
brevipetiolata Wagner, 1932
platyura Gussakovskij, 1952
minor Gussakovskij, 1952
levinota Merisuo, 1972
nannophyes Merisuo, 1972
dispar Valkeila, 1972
gemina Valkeila, 1972
trichogastor Valkeila, 1972
sudaorum Tsuneki, 1977

##### Distribution

England, Scotland, Wales, Ireland, Isle of Man

#### Pemphredon lugubris

(Fabricius, 1793)

Crabro lugubris Fabricius, 1793
concolor Say, 1824
ocellaris Gimmerthal, 1836
luctuosa Shuckard, 1837
morio Cresson, 1865 preocc.
cressoni Dalla Torre, 1897
provancheri Dalla Torre, 1897
tinctipennis Cameron, 1908
shawii Rohwer, 1917
pacifica Gussakovkij, 1932

##### Distribution

England, Scotland, Wales, Ireland, Isle of Man

#### Pemphredon morio

Vander Linden, 1829

anthracinus (Smith, 1851, *Ceratophorus*)
carinata Thomson, 1870
clypealis Thomson, 1870
intermedia Tsuneki, 1951

##### Distribution

England, Wales

#### Pemphredon rugifer

(Dahlbom, 1844)

Cemonus rugifer Dahlbom, 1844
rugifera misspelling
unicolor (Panzer, 1798, *Sphex*) preocc.
pilosa (Gimmerthal, 1836, *Cemonus*)
wesmaeli (Morawitz, 1864, *Cemonus*)
scotica Perkins, 1929
solivaga (Bondroit, 1931, *Cemonus*)
bucharicus Gussakovskij, 1952
mortifer Valkeila, 1972
punctifer Valkeila, 1972
scytica Valkeila, 1972

##### Distribution

England, Scotland

#### Spilomena

Shuckard, 1838

CELIA Shuckard, 1837 preocc.
MICROGLOSSA Rayment, 1930 preocc.
MICROGLOSSELLA Rayment, 1935
TAIALIA Tsuneki, 1971

#### Spilomena beata

Blüthgen, 1953

exspectata Valkeila, 1957

##### Distribution

England

#### Spilomena differens

Blüthgen, 1953

curruca misident.

##### Distribution

England

##### Notes

Has been called *curruca* (Dahlbom, 1844, *Celia*) in several publications, following the synonymy by Dollfuss (1986), but was reinstated as a valid species by Vikberg (2000).

#### Spilomena curruca

(Dahlbom, 1844)

Celia curruca Dahlbom, 1844
pulawskii Dolfuss, 1983
nikkoensis Tsuneki, 1971

##### Distribution

England

#### Spilomena enslini

Blüthgen, 1953

##### Distribution

England, Ireland

#### Spilomena troglodytes

(Vander Linden, 1829)

Stigmus troglodytes Vander Linden, 1829
minutissimus (Radoszkowski, 1877, *Stigmus*)
vagans Blüthgen, 1953

##### Distribution

England, Scotland, Wales

#### Stigmus

Panzer, 1805

ANTRONIUS Zetterstedt, 1838
GONOSTIGMUS Rohwer, 1911
ATOPOSTIGMUS Krombein, 1973

#### Stigmus pendulus

Panzer, 1804

ater Jurine, 1807

##### Distribution

England

##### Notes

added by Allen (1987)

#### Stigmus solskyi

Morawitz, 1864

europaeus Tsuneki, 1954
verhoeffi Tsuneki, 1954

##### Distribution

England, Wales

#### Psenini

Costa, 1858

#### Mimesa

Shuckard, 1837

APORIA Wesmael, 1852 preocc.
APORINA Gussakovskij, 1937 preocc.

#### Mimesa bicolor

(Jurine, 1807)

Psen bicolor Jurine, 1807
equestris misident.
rufa (Panzer, 1805, *Psen*)

##### Distribution

England

#### Mimesa bruxellensis

Bondroit, 1934

rossicus (Gussakovskij, 1937, *Psen*)

##### Distribution

England, Wales

#### Mimesa equestris

(Fabricius, 1804)

Trypoxylon equestre Fabricius, 1804
bicolor misident.

##### Distribution

England, Scotland, Wales

#### Mimesa lutaria

(Fabricius, 1787)

Sphex lutarius Fabricius, 1787
shuckardi Wesmael, 1852
basirufa Packard, 1867
nebrascensis Smith, 1908
dispar (Gussakovskij, 1937, *Psen*)
mallochi Finnamore, 1980

##### Distribution

England, Wales

#### Mimumesa

Malloch, 1933

#### Mimumesa atratina

(Morawitz, 1891)

Mimesa atratina Morawitz, 1891
carbonaria (Tournier, 1899, *Mimesa*)
belgica (Bondroit, 1932, *Mimesa*)
longula (Gussakovskij, 1932, *Mimesa*)
sameshimai (Yasumatsu, 1937, *Psen*)

##### Distribution

England

#### Mimumesa dahlbomi

(Wesmael, 1852)

Mimesa dahlbomi Wesmael, 1852

##### Distribution

England, Scotland, Wales

#### Mimumesa littoralis

(Bondroit, 1934)

Mimesa littoralis Bondroit, 1934
unicolor misident.
fulvitarsis (Gussakovskij, 1934, *Psen*)
fulvitarsis (Gussakovskij, 1937, *Psen*) preocc.
celtica (Spooner, 1948, *Mimesa*)

##### Distribution

England, Wales, Ireland

#### Mimumesa spooneri

(Richards, 1948)

Mimesa spooneri Richards, 1948

##### Distribution

England

#### Mimumesa unicolor

(Vander Linden, 1829)

Psen unicolor Vander Linden, 1829
borealis (Dahlbom, 1842, *Mimesa*)
palliditarsis (Saunders, 1904, *Mimesa*)
oresterus (van Lith, 1976, *Psen*)

##### Distribution

England

##### Notes

added by Else and Felton (1994)

#### Psen

Latreille, 1796

PSENUS Rafinesque, 1815
PSENIA Stephens, 1829
DAHLBOMIA Wissmann, 1849
MESOPORA Wesmael, 1852
CAENOPSEN Cameron, 1899

#### Psen ater

(Olivier, 1792)

Crabro ater Olivier, 1792
ater (Fabricius, 1794, *Sphex*) preocc.
compressicornis (Fabricius, 1804, *Pelopoeus*)
atratus (Jurine, 1807, *Psen*)
serraticornis (Jurine, 1807, *Psen*)

##### Distribution

England

#### Psenulus

Kohl, 1897

DIODONTUS misident.
NEOFOXIA Viereck, 1901
STENOMELLINUS Schulz, 1911
EOPSENULUS Gussakovskij, 1934
NIPPONOPSEN Yasumatsu, 1938

##### Notes

*Diodontus* is a misidentification by American authors in the older literature

#### Psenulus concolor

(Dahlbom, 1843)

Psen concolor Dahlbom, 1843
intermedius (Schenck, 1857, *Psen*)
ambiguus (Schenck, 1857, *Psen*)

##### Distribution

England, Wales

#### Psenulus pallipes

(Panzer, 1798)

Sphex pallipes Panzer, 1798
atratus (Fabricius, 1804, *Trypoxylon*)
montanus (Costa, 1861, *Psen*)
haemorrhoidalis (Costa, 1871, *Psen*)
minutus (Tournier, 1899, *Psen*)
chevrieri (Tournier, 1899, *Psen*)
nigricornis (Tournier, 1899, *Psen*)
pygmaeus (Tournier, 1899, *Psen*)
rubicola Harttig, 1931
brevitarsis Merisuo, 1937

##### Distribution

England, Wales, Ireland

#### Psenulus schencki

(Tournier, 1889)

Psen schencki Tournier, 1889
simplex (Tournier, 1899, *Psen*)
longulus (Tournier, 1899, *Psen*)

##### Distribution

England

#### Philanthinae

Latreille, 1802

#### Cercerini

Lepeletier, 1845

#### Cerceris

Latreille, 1802

NECTANEBUS Spinola, 1839
DIAMMA Dahlbom, 1844
DIDESMUS Dahlbom, 1845
APIRAPTRIX Shestakov, 1923
PARACERCERIS Brèthes, 1913
BUCERCERIS Minkiewicz, 1934
STERCOBATA Gussakovskij, 1935
APICERCERIS Pate, 1937

#### Cerceris arenaria

(Linnaeus, 1758)

Sphex arenarius Linnaeus, 1758
xanthocephala (Forster, 1771, *Sphex*)
exulta (Harris, 1776, *Vespa*)
petulans (Harris, 1776, *Vespa*)
serripes (Fabricius, 1781, *Vespa*)
arenosa (Gmelin, 1790, *Vespa*)
aurita (Fabricius, 1794, *Philanthus*)
striolata Schletterer, 1887

##### Distribution

England, Wales

#### Cerceris quadricincta

(Panzer, 1799)

Philanthus quadricinctus Panzer, 1799
fasciata Spinola, 1806

##### Distribution

England

#### Cerceris quinquefasciata

(Rossi, 1792)

Crabro quinquefasciatus Rossi, 1792
interrupta misident.
nasuta Dahlbom, 1844 preocc.
subdepressa Lepeletier, 1845

##### Distribution

England

#### Cerceris ruficornis

(Fabricius, 1793)

Philanthus ruficornis Fabricius, 1793
labiata misident.
bidens (Schrank, 1802, *Crabro*)
cunicularia (Schrank, 1802, *Crabro*)
quadricincta (Fabricius, 1804, *Mellinus*)
trifidus (Fabricius, 1804, *Philanthus*)
nasuta Latreille, 1809
laminifera Costa, 1867

##### Distribution

England

#### Cerceris rybyensis

(Linnaeus, 1771)

Sphex rybyensis Linnaeus, 1771
ornata (Fabricius, 1790, *Philanthus*)
apifalco (Christ, 1791, *Sphex*)
semicincta (Panzer, 1797, *Philanthus*)
hortorum (Panzer, 1799, *Philanthus*)
variabilis (Schrank, 1802, *Crabro*)
biguttata (Thunberg, 1815, *Philanthus*)
colon (Thunberg, 1815, *Philanthus*)
kashmirensis Nurse, 1903
dittrichi Schulz, 1904
reginae Eck, 1979

##### Distribution

England

#### Cerceris sabulosa

(Panzer, 1799)

Philanthus sabulosus Panzer, 1799
emarginata (Panzer, 1799, *Philanthus*)
tricincta (Spinola, 1805, *Philanthus*)
pygmaea (Thunberg, 1815, *Philanthus*)
minuta Lepeletier, 1845
superba Shestakov, 1923

##### Distribution

England

#### Philanthini

Latreille, 1802

#### Philanthus

Fabricius, 1790

SYMBLEPHILUS Panzer, 1806
SIMBLEPHILUS Jurine, 1807
CHEILOPOGONUS Westwood, 1835
ANTHOPHILUS Dahlbom, 1844
EPHIPHILANTHUS Ashmead, 1899
PSEUDOPHILANTHUS Ashmead, 1899
OCLOCLETES Banks, 1913

#### Philanthus triangulum

(Fabricius, 1775)

Vespa triangulum Fabricius, 1775
ruspatrix (Linnaeus, 1767, *Vespa*) nom. ob.
fasciatus (Geoffroy, 1785, *Vespa*)
maculatus (Christ, 1791, *Sphex*)
limbatus (Olivier, 1792, *Vespa*)
androgynus (Rossi, 1792, *Crabro*)
pictus Panzer, 1797
discolor Panzer, 1799
apivorus Latreille, 1799
allionii Dahlbom, 1845

##### Distribution

England, Wales

#### Sphecidae

Latreille, 1802

#### Ammophilinae

André, 1886

#### Ammophila

Kirby, 1798

SPHEX misident.
MISCUS Jurine, 1807
COLOPTERA Latreille, 1845
ARGYRAMMOPHILA Gussakovskij, 1928
APYCNEMIA Leclercq, 1961

#### Ammophila pubescens

Curtis, 1836

campestris misident.
arvensis (Dahlbom, 1843, *Miscus*)
susterai Šnoflak, 1943
adriaansei Wilcke, 1945

##### Distribution

England, Wales

#### Ammophila sabulosa

(Linnaeus, 1758)

Sphex sabulosus Linnaeus, 1758
hortensis (Poda, 1761, *Sphex*)
frischii (Geoffroy, 1785, *Ichneumon*)
dimidiata (Christ, 1791, *Sphex*) preocc.
vulgaris Kirby, 1798
pulvillata Sowerby, 1805
mucronata (Jurine, 1807, *Sphex*)
cyanescens Dahlbom, 1845
vischu Cameron, 1889
kamtschatica Gussakovskij, 1932

##### Distribution

England, Scotland, Wales, Ireland

#### Podalonia

Fernald, 1927

PSAMMOPHILA Dahlbom, 1842 preocc.
PODALONIA Spinola, 1853 suppressed

#### Podalonia affinis

(Kirby, 1798)

Ammophila affinis Kirby, 1798
lutaria misident.
ariasi (Mercet, 1906, *Ammophila*)

##### Distribution

England, Wales

#### Podalonia hirsuta

(Scopoli, 1763)

Sphex hirsutus Scopoli, 1763
viatica misident.
arenaria (Fabricius, 1787, *Sphex*) preocc.
arenosa (Gmelin, 1790, *Sphex*)
argentea (Kirby, 1798, *Ammophila*)

##### Distribution

England, Wales

#### Anthophila

Latreille, 1804

##### Notes

Engel (2005) proposed that the informal name Anthophila, without formal rank, be used for the bees (here denoted as an 'infraorder' due to restrictions in the available fields). Note that 'apiformes' and the corresponding 'spheciformes') is an equivalent term proposed by Brothers (1975)

#### Andrenidae

Latreille, 1802

#### Andreninae

Latreille, 1802

#### Andrena

Fabricius, 1775

##### Notes

Taxonomy mostly follows Gusenleitner and Schwarz (2002).

#### Andrena

Fabricius, 1775

ANTHRENA Illiger, 1801
ANTHOCHARESSA Gistel, 1850

#### Andrena (Andrena) apicata

Smith, 1847

##### Distribution

England, Wales, Ireland

#### Andrena (Andrena) clarkella

(Kirby, 1802)

Melitta clarkella Kirby, 1802

##### Distribution

England, Scotland, Wales, Ireland, Isle of Man

#### Andrena (Andrena) fucata

Smith, 1847

##### Distribution

England, Scotland, Wales, Ireland, Isle of Man

#### Andrena (Andrena) fulva

(Müller, 1766)

Apis fulva Müller, 1766
armata (Gmelin, 1790, *Apis*)

##### Distribution

England, Scotland, Wales, Ireland

#### Andrena (Andrena) helvola

(Linnaeus, 1758)

Apis helvola Linnaeus, 1758
subdentata (Kirby, 1802, *Melitta*)

##### Distribution

England, Scotland, Wales

#### Andrena (Andrena) lapponica

Zetterstedt, 1838

##### Distribution

England, Scotland, Wales, Ireland, Isle of Man

#### Andrena (Andrena) praecox

(Scopoli, 1763)

Apis praecox Scopoli, 1763
smithella (Kirby, 1802, *Melitta*)
clypeata Smith, 1855 preocc.

##### Distribution

England, Wales, Ireland

#### Andrena (Andrena) synadelpha

Perkins, 1914

ambigua Perkins, 1895, preocc.

##### Distribution

England, Scotland, Wales

#### Andrena (Andrena) varians

(Kirby, 1802)

Melitta varians Kirby, 1802
varians (Rossius, 1792, *Apis*): misident.
angulosa (Kirby, 1802, *Melitta*)

##### Distribution

England, Wales

#### Charitandrena

Hedicke, 1933

#### Andrena (Charitandrena) hattorfiana

(Fabricius, 1775)

Nomada hattorfiana Fabricius, 1775
lathamana (Kirby, 1802, *Melitta*)

##### Distribution

England, Wales

#### Chlorandrena

Pérez, 1890

#### Andrena (Chlorandrena) humilis

Imhoff, 1832

fulvescens Smith, 1847

##### Distribution

England, Wales, Isle of Man

#### Chrysandrena

Hedicke, 1933

#### Andrena (Chrysandrena) fulvago

(Christ, 1791)

Apis fulvago Christ, 1791
constricta Smith, 1849

##### Distribution

England, Wales

#### Cnemidandrena

Hedicke, 1933

#### Andrena (Cnemidandrena) denticulata

(Kirby, 1802)

Melitta denticulata Kirby, 1802
listerella (Kirby, 1802, *Melitta*)

##### Distribution

England, Scotland, Wales, Ireland, Isle of Man

#### Andrena (Cnemidandrena) fuscipes

(Kirby, 1802)

Melitta fuscipes Kirby, 1802

##### Distribution

England, Scotland, Wales, Ireland, Isle of Man

#### Andrena (Cnemidandrena) nigriceps

(Kirby, 1802)

Melitta nigriceps Kirby, 1802
lanifrons (Kirby, 1802, *Melitta*)

##### Distribution

England, Scotland, Wales

#### Andrena (Cnemidandrena) simillima

Smith, 1851

##### Distribution

England

#### Andrena (Cnemidandrena) tridentata

(Kirby, 1802)

Melitta tridentata Kirby, 1802
rufitarsis (Kirby, 1802, *Melitta*)

##### Distribution

England

##### Notes

Probably extinct in Britain.

#### Euandrena

Hedicke, 1933

XANTHANDRENA Lanham, 1949
GEANDRENA LaBerge, 1964

#### Andrena (Euandrena) bicolor

Fabricius, 1775

picicornis (Kirby, 1802, *Melitta*)
pilosula (Kirby, 1802, *Melitta*)
gwynana (Kirby, 1802, *Melitta*)
proxima Smith, 1847 preocc.
aestiva Smith, 1849
consimilis Smith, 1849 preocc.

##### Distribution

England, Scotland, Wales, Ireland, Isle of Man

#### Andrena (Euandrena) ruficrus

Nylander, 1848

##### Distribution

England, Scotland

#### Holandrena

Pérez, 1890

#### Andrena (Holandrena) labialis

(Kirby, 1802)

Melitta labialis Kirby, 1802
separata Smith, 1847

##### Distribution

England, Wales, Isle of Man

#### Hoplandrena

Pérez, 1890

#### Andrena (Hoplandrena) bucephala

Stephens, 1846

eximia Smith, 1847
longipes Smith, 1847

##### Distribution

England, Wales

#### Andrena (Hoplandrena) ferox

Smith, 1847

distincta Smith, 1847

##### Distribution

England

#### Andrena (Hoplandrena) rosae

Panzer, 1801

eximia misident.
zonalis (Kirby, 1802, *Melitta*)
stragulata Illiger, 1806
strangulata misspelling

##### Distribution

England, Wales, Ireland

##### Notes

*stragulata* is the summer brood of *rosae*

#### Andrena (Hoplandrena) scotica

Perkins, 1916

carantonica misident.
jacobi Perkins, 1921
johnsoni Perkins, 1921

##### Distribution

England, Scotland, Wales, Ireland, Isle of Man

##### Notes

Although usually referred to as *scotica* in Britain (e.g. Else et al. 2004), Schwarz et al. (1996) and Gusenleitner and Schwarz (2002) have treated *scotica* as a synonym of *carantonica* Peréz, 1916; however, Else (in prep.), following P. Westrich’s (in lit.) interpretation of the type of *carantonica*, regards this is a separate species, with *carantonica* a junior synonym of *trimmerana*.

#### Andrena (Hoplandrena) trimmerana

(Kirby, 1802)

Melitta trimmerana Kirby, 1802
spinigera (Kirby, 1802, *Melitta*)
carantonica Peréz, 1916

##### Distribution

England, Wales

#### Leucandrena

Hedicke, 1933

#### Andrena (Leucandrena) argentata

Smith, 1844

##### Distribution

England

#### Andrena (Leucandrena) barbilabris

(Kirby, 1802)

Melitta barbilabris Kirby, 1802
sericea (Christ, 1791, *Apis*) preocc.
albicrus (Kirby, 1802, *Melitta*)

##### Distribution

England, Scotland, Wales, Ireland, Isle of Man

#### Margandrena

Warncke, 1968

#### Andrena (Margandrena) marginata

Fabricius, 1776

cetii (Schrank, 1781, *Apis*)
schrankella (Kirby, 1802, *Melitta*)
frontalis Smith, 1849

##### Distribution

England, Scotland, Wales, Ireland

#### Melandrena

Pérez, 1890

GYMNANDRENA Hedicke, 1933
CRYPTANDRENA Lanham, 1949 preocc.
BYTHANDRENA Lanham, 1950

#### Andrena (Melandrena) cineraria

(Linnaeus, 1758)

Apis cineraria Linnaeus, 1758

##### Distribution

England, Scotland, Wales, Ireland, Isle of Man

#### Andrena (Melandrena) nigroaenea

(Kirby, 1802)

Melitta nigroaenea Kirby, 1802
aprilina Smith, 1848

##### Distribution

England, Scotland, Wales, Ireland, Isle of Man

##### Notes

The population on the Isles of Scilly and Channel Islands has been described as the subspecies *sarnia* Richards, 1979.

#### Andrena (Melandrena) nitida

(Müller, 1776)

Apis nitida Müller, 1776
pubescens Olivier, 1789
consimilis Smith, 1847

##### Distribution

England, Wales, Ireland

#### Andrena (Melandrena) thoracica

(Fabricius, 1775)

Apis thoracica Fabricius, 1775
melanocephala (Kirby, 1802, *Melitta*)

##### Distribution

England, Wales

#### Andrena (Melandrena) vaga

Panzer, 1799

##### Distribution

England

##### Notes

Probably extinct in Britain.

#### Micrandrena

Ashmead, 1899

ANDRENELLA Hedicke, 1933

#### Andrena (Micrandrena) alfkenella

Perkins, 1914

moricella Perkins, 1914

##### Distribution

England

#### Andrena (Micrandrena) falsifica

Perkins, 1915

##### Distribution

England

#### Andrena (Micrandrena) floricola

Eversmann, 1852

##### Notes

Probably extinct in Britain.

#### Andrena (Micrandrena) minutula

(Kirby, 1802)

Melitta minutula Kirby, 1802
parvula (Kirby, 1802, *Melitta*)
nigrifrons Smith, 1855 preocc.

##### Distribution

England, Wales, Ireland

#### Andrena (Micrandrena) minutuloides

Perkins, 1914

parvuloides Perkins, 1914

##### Distribution

England

#### Andrena (Micrandrena) nana

(Kirby, 1802)

Melitta nana Kirby, 1802

##### Distribution

England

##### Notes

Probably extinct in Britain.

#### Andrena (Micrandrena) nanula

Nylander, 1848

##### Notes

Probably extinct in Britain.

#### Andrena (Micrandrena) niveata

Friese, 1887

spreta misident.

##### Distribution

England, Wales

#### Andrena (Micrandrena) semilaevis

Pérez, 1903

nana misident.
saundersella Perkins, 1914

##### Distribution

England, Scotland, Wales, Ireland, Isle of Man

#### Andrena (Micrandrena) subopaca

Nylander, 1848

##### Distribution

England, Scotland, Wales, Ireland

#### Notandrena

Pérez, 1890

#### Andrena (Notandrena) chrysosceles

(Kirby, 1802)

Melitta chrysosceles Kirby, 1802
connectens (Kirby, 1802, *Melitta*)

##### Distribution

England, Scotland, Wales

#### Andrena (Notandrena) nitidiuscula

Schenck, 1853

lucens Imhoff, 1868

##### Distribution

England

#### Oreomelissa

Hirashima & Tadauchi, 1975

#### Andrena (Oreomelissa) coitana

(Kirby, 1802)

Melitta coitana Kirby, 1802
shawella (Kirby, 1802, *Melitta*)

##### Distribution

England, Scotland, Wales, Ireland, Isle of Man

#### Plastandrena

Hedicke, 1933

SCHIZANDRENA Hedicke, 1933
GLYPHANDRENA Hedicke, 1933
MITSUKURIELLA Hirashima & LaBerge, 1965
MITSUKURIAPIS Hirashima, LaBerge & Ikudomem 1884

#### Andrena (Plastandrena) bimaculata

(Kirby, 1802)

Melitta bimaculata Kirby, 1802
articulata Smith, 1847
conjuncta Smith, 1847
decorata Smith, 1847
vitrea Smith, 1847

##### Distribution

England, Wales

#### Andrena (Plastandrena) nigrospina

Thomson, 1872

##### Distribution

England

##### Notes

Although treated as a synonym of *pilipes* by Gusenleitner and Schwarz (2002) here *nigrospina* is treated as a valid species, following Baker (1994).

#### Andrena (Plastandrena) pilipes

Fabricius, 1781

carbonaria misident.
spectabilis Smith, 1853
praetexta Smith, 1872

##### Distribution

England

#### Andrena (Plastandrena) tibialis

(Kirby, 1802)

Melitta tibialis Kirby, 1802
mouffetella (Kirby, 1802, *Melitta*)
atriceps (Kirby, 1802, *Melitta*)

##### Distribution

England

#### Poecilandrena

Hedicke, 1933

#### Andrena (Poecilandrena) labiata

Fabricius, 1781

cingulata misident.

##### Distribution

England, Wales, Ireland

#### Poliandrena

Warncke, 1968

#### Andrena (Poliandrena) florea

Fabricius, 1793

rubricata Smith, 1847

##### Distribution

England

#### Andrena (Poliandrena) polita

Smith, 1847

##### Distribution

England

##### Notes

Probably extinct in Britain.

#### Proxiandrena

Schmid-Egger, 2005

#### Andrena (Proxiandrena) proxima

(Kirby, 1802)

Melitta proxima Kirby, 1802
digitalis (Kirby, 1802, *Melitta*)

##### Distribution

England, Wales

#### Ptilandrena

Robertson, 1902

EREMANDRENA LaBerge, 1964

#### Andrena (Ptilandrena) angustior

(Kirby, 1802)

Melitta angustior Kirby, 1802
lacinia Smith, 1847

##### Distribution

England, Scotland, Wales, Ireland, Isle of Man

#### Simandrena

Pérez, 1890

PLATANDRENA Viereck, 1924
STENANDRENA Timberlake, 1949

#### Andrena (Simandrena) congruens

Schmiedeknecht, 1884

confinis Stöckhert, 1930

##### Distribution

England, Wales

#### Andrena (Simandrena) dorsata

(Kirby, 1802)

Melitta dorsata Kirby, 1802
collinsonana (Kirby, 1802, *Melitta*)
lewinella (Kirby, 1802, *Melitta*)
nudiuscula (Kirby, 1802, *Melitta*)
subincana (Kirby, 1802, *Melitta*)

##### Distribution

England, Wales

#### Andrena (Simandrena) lepida

Schenck, 1861

##### Distribution

England

##### Notes

Probably extinct in Britain.

#### Taeniandrena

Hedicke, 1933

#### Andrena (Taeniandrena) lathyri

Alfken, 1899

##### Distribution

England

#### Andrena (Taeniandrena) ovatula

(Kirby, 1802)

Melitta ovatula Kirby, 1802
afzeliella (Kirby, 1802, *Melitta*)
barbata (Kirby, 1802, *Melitta*)
fuscata (Kirby, 1802, *Melitta*)
picipes (Kirby, 1802, *Melitta*)
albofasciata Thomson, 1870

##### Distribution

England, Wales

#### Andrena (Taeniandrena) similis

Smith, 1849

ocreata Christ, 1791 nom. dub.

##### Distribution

England, Scotland, Wales

#### Andrena (Taeniandrena) wilkella

(Kirby, 1802)

Melitta wilkella Kirby, 1802
barbatula (Kirby, 1802, *Melitta*)
convexiuscula (Kirby, 1802, *Melitta*)
xanthura (Kirby, 1802, *Melitta*)

##### Distribution

England, Scotland, Wales, Ireland, Isle of Man

#### Tarsandrena

Osytshnjuk, 1984

#### Andrena (Tarsandrena) tarsata

Nylander, 1848

analis misident.

##### Distribution

England, Scotland, Wales, Ireland, Isle of Man

#### Trachandrena

Robertson, 1902

#### Andrena (Trachandrena) haemorrhoa

(Fabricius, 1781)

Apis haemorrhoa Fabricius, 1781
albicans misident.

##### Distribution

England, Scotland, Wales, Ireland, Isle of Man

#### Zonandrena

Hedicke, 1933

#### Andrena (Zonandrena) flavipes

Panzer, 1799

contigua (Kirby, 1802, *Melitta*)
fulvicrus (Kirby, 1802, *Melitta*)
extricata Smith, 1849

##### Distribution

England, Wales, Ireland

#### Andrena (Zonandrena) gravida

Imhoff, 1832

fasciata Nylander, 1852 preocc.
picicrus Schenck, 1853

##### Distribution

England

#### Panurginae

Leach, 1815

#### Panurgini

Leach, 1815

#### Panurgus

Panzer, 1806

#### Panurgus banksianus

(Kirby, 1802)

Apis banksiana Kirby, 1802
ursinus misident.

##### Distribution

England, Wales

#### Panurgus calcaratus

(Scopoli, 1763)

Apis calcarata Scopoli, 1763
linnaeella (Kirby, 1802, *Apis*)

##### Distribution

England, Wales

#### Apidae

Latreille, 1802

#### Apinae

Latreille, 1802

#### Anthophorini

Dahlbom, 1835

#### Anthophora

Latreille, 1803

#### Anthophora

Latreille, 1803

#### Anthophora (Anthophora) plumipes

(Pallas, 1772)

Apis plumipes Pallas, 1772
acervorum misident.
pilipes (Fabricius, 1775, *Apis*)

##### Distribution

England, Wales

#### Clisodon

Patton, 1879

#### Anthophora (Clisodon) furcata

(Panzer, 1798)

Apis furcata Panzer, 1798

##### Distribution

England, Scotland, Wales

#### Dasymegilla

Brooks, 1988

#### Anthophora (Dasymegilla) quadrimaculata

(Panzer, 1798)

Apis quadrimaculata Panzer, 1798
subglobosa (Kirby, 1802, *Apis*)

##### Distribution

England, Wales

#### Heliophila

Klug, 1807

SAROPODA Latreille, 1809

#### Anthophora (Heliophila) bimaculata

(Panzer, 1798)

Apis bimaculata Panzer, 1798

##### Distribution

England, Ireland

#### Pyganthophora

Brooks, 1988

#### Anthophora (Pyganthophora) retusa

(Linnaeus, 1758)

Apis retusa Linnaeus, 1758
haworthana (Kirby, 1802, *Apis*)
pennipes (Kirby, 1802, *Apis*)

##### Distribution

England

#### Apini

Latreille, 1802

#### Apis

Linnaeus, 1758

#### Apis mellifera

Linnaeus, 1758

mellifica Linnaeus, 1761

##### Distribution

England, Scotland, Wales, Ireland, Isle of Man

##### Notes

There have been various subspecies names proposed for honey bees, with the supposedly native north west European population being *A. mellifera mellifera*. See Engel (1999) for a full taxonomy of *Apis mellifera*.

#### Bombini

Latreille, 1802

#### Bombus

Latreille, 1802

##### Notes

Various subspecific names have been used for British *Bombus* but these are generally poorly justified and we have treated these as synonymous here. Subgeneric classification follows Williams et al. (2008).

#### Bombus

Latreille, 1802

TERRESTRIBOMBUS Vogt, 1911

##### Notes

Full synonymy for this subgenus is given by Williams et al. (2012), who shed some light on the confusing classification of *cryptarum* and *magnus* relative to *lucorum.*

#### Bombus (Bombus) cryptarum

(Fabricius, 1775)

Apis cryptarum Fabricius, 1775

##### Distribution

Scotland, Ireland

##### Notes

added by Bertsch et al. (2005)

#### Bombus (Bombus) lucorum

(Linnaeus, 1761)

Apis lucorum Linnaeus, 1761

##### Distribution

England, Scotland, Wales, Ireland, Isle of Man

#### Bombus (Bombus) magnus

Vogt, 1911

##### Distribution

Scotland, Ireland

#### Bombus (Bombus) terrestris

(Linnaeus, 1758)

Apis terrestris Linnaeus, 1758
audax (Harris, 1776, *Apis*)

##### Distribution

England, Scotland, Wales, Ireland, Isle of Man

#### Cullumanobombus

Vogt, 1911

#### Bombus (Cullumanobombus) cullumanus

(Kirby, 1802)

Apis cullumana Kirby, 1802

##### Distribution

England

##### Notes

Extinct in Britain since the 1940s.

#### Kallobombus

Dalla Torre, 1880

#### Bombus (Kallobombus) soroeensis

(Fabricius, 1777)

Apis soroeensis Fabricius, 1777

##### Distribution

England, Scotland, Wales

#### Megabombus

Dalla Torre, 1880

HORTOBOMBUS Vogt, 1911

#### Bombus (Megabombus) hortorum

(Linnaeus, 1761)

Apis hortorum Linnaeus, 1761
flavonigrescens Smith, 1846
ivernicus Sladen, 1912
splendida Stelfox, 1938

##### Distribution

England, Scotland, Wales, Ireland, Isle of Man

##### Notes

Populations in Ireland have been referred to the subspecies *B. hortorum ivernicus*.

#### Bombus (Megabombus) ruderatus

(Fabricius, 1775)

Apis ruderata Fabricius, 1775
perniger (Harris, 1776, *Apis*)
harrisellus (Kirby, 1802, *Apis*)
tunstallanus (Kirby, 1802, *Apis*)

##### Distribution

England

##### Notes

English populations have been referred to the subspecies *B. ruderatus perniger*.

#### Melanobombus

Dalla Torre, 1880

LAPIDARIOBOMBUS Vogt, 1911

#### Bombus (Melanobombus) lapidarius

(Linnaeus, 1758)

Apis lapidaria Linnaeus, 1758

##### Distribution

England, Scotland, Wales, Ireland, Isle of Man

#### Psithyrus

Lepeletier, 1832

ALLOPSITHYRUS Popov, 1931
ASHTONIPSITHYRUS Frison, 1927
FERNALDAEPSITHYRUS Frison, 1927
METAPSITHYRUS Popov, 1931

#### Bombus (Psithyrus) barbutellus

(Kirby, 1802)

Apis barbutella Kirby, 1802

##### Distribution

England, Scotland, Wales, Ireland

#### Bombus (Psithyrus) bohemicus

(Seidl, 1837)

Apis bohemicus Seidl, 1837
distinctus (Pérez, 1884, *Psithyrus*)

##### Distribution

England, Scotland, Wales, Ireland, Isle of Man

#### Bombus (Psithyrus) campestris

(Panzer, 1801)

Apis campestris Panzer, 1801
rossiellus (Kirby, 1802, *Apis*)
leeanus (Kirby, 1802, *Apis*)
francisanus (Kirby, 1802, *Apis*)
swynnertoni (Richards, 1936, *Psithyrus*)

##### Distribution

England, Scotland, Wales, Ireland, Isle of Man

##### Notes

Populations in Western Scotland have been referred to the subspecies *B. campestris swynnertoni*.

#### Bombus (Psithyrus) rupestris

(Fabricius, 1793)

Apis rupestris Fabricius, 1793
albinellus (Kirby, 1802, *Apis*)

##### Distribution

England, Wales, Ireland

#### Bombus (Psithyrus) sylvestris

(Lepeletier, 1832)

Psithyrus sylvestris Lepeletier, 1832
quadricolor misident.

##### Distribution

England, Scotland, Wales, Ireland

#### Bombus (Psithyrus) vestalis

(Geoffroy, 1785)

Apis vestalis Geoffroy, 1785

##### Distribution

England, Scotland, Wales, Ireland

#### Pyrobombus

Dalla Torre, 1880

PRATOBOMBUS Vogt, 1911

#### Bombus (Pyrobombus) hypnorum

(Linnaeus, 1758)

Apis hypnorum Linnaeus, 1758

##### Distribution

England, Scotland, Wales

##### Notes

added by Goulson and Williams (2001)

#### Bombus (Pyrobombus) jonellus

(Kirby, 1802)

Apis jonella Kirby, 1802
nivalis misident.
scrimshiranus (Kirby, 1802, *Apis*)
atrocorbiculosus Vogt, 1911
hebridensis Wild, 1931
vogtii Richards, 1933 preocc.
monapiae Kruseman, 1953
vogtianus Rasmont, 1983

##### Distribution

England, Scotland, Wales, Ireland, Isle of Man

##### Notes

The following populations have been given subspecies names: *B. jonellus hebridensis* on the Hebrides; *B. jonellus monapiae* on the Isle of Man; *B. jonellus vogtianus* on the Shetlands (*vogtianus* is a replacement name for the preccupied *vogtii*).

#### Bombus (Pyrobombus) monticola

Smith, 1849

lapponicus misident.
scoticus Pittioni, 1942

##### Distribution

England, Scotland, Wales, Ireland, Isle of Man

#### Bombus (Pyrobombus) pratorum

(Linnaeus, 1761)

Apis pratorum Linnaeus, 1761
subinterruptus (Kirby, 1802, *Apis*)
donovanella (Kirby, 1802, *Apis*)
burrellana (Kirby, 1802, *Apis*)

##### Distribution

England, Scotland, Wales, Ireland, Isle of Man

#### Subterraneobombus

Vogt, 1911

#### Bombus (Subterraneobombus) distinguendus

Morawitz, 1869

##### Distribution

England, Scotland, Wales, Ireland, Isle of Man

#### Bombus (Subterraneobombus) subterraneus

(Linnaeus, 1758)

Apis subterranea Linnaeus, 1758
collinus Smith, 1844
latreillellus (Kirby, 1802, *Apis*)

##### Distribution

England, Wales

##### Notes

Extinct in Britain since the 1980s but an attempt is being made to reintroduce the species to southern England. British populations have been referred to the subspecies *B. subterraneus latreillellus* whereas the introduced individuals from Sweden have been ascribed to the nominate subspecies.

#### Thoracobombus

Dalla Torre, 1880

POMOBOMBUS Krüger, 1917

#### Bombus (Thoracobombus) humilis

Illiger, 1806

solstitialis Panzer, 1806
helferanus Seidl, 1837
venustus Smith, 1876 preocc.
anglicus Yarrow, 1978

##### Distribution

England, Scotland, Wales, Isle of Man

#### Bombus (Thoracobombus) muscorum

(Linnaeus, 1758)

Apis muscorum Linnaeus, 1758
arcticus misident.
smithianus misident.
pallidus Evans, 1901 preocc.
laevis Vogt, 1909
sladeni Vogt, 1911
allenellus Stelfox, 1933
orcadensis Richards, 1935
scyllonius Richards, 1935
celticus Yarrow, 1978
agricolae Baker, 1996

##### Distribution

England, Scotland, Wales, Ireland, Isle of Man

##### Notes

The following populations have been given subspecies names: *B. muscorum sladeni* on the Irish mainland and southern England; *B. muscorum allenellus* on the Aran islands; *B. muscorum orcadensis* on Orkney; *B. muscorum scyllonius* on the Isles of Scilly; *B. muscorum celticus* in mainland Scotland and Northern England; *B. muscorum agricolae* on the Shetland islands.

#### Bombus (Thoracobombus) pascuorum

(Scopoli, 1763)

Apis pascuorum Scopoli, 1763
vulgo (Harris, 1776, *Apis*)
agrorum (Fabricius, 1787, *Apis*) preocc.
floralis (Gmelin, 1790, *Apis*)
francillonellus (Kirby, 1802, *Apis*)
sowerbianus (Kirby, 1802, *Apis*)
beckwithella (Kirby, 1802, *Apis*)
curtisellus (Kirby, 1802, *Apis*)
forsterellus (Kirby, 1802, *Apis*)
cognatus Stevens, 1846
smithianus White, 1851
septentrionalis Vogt, 1909

##### Distribution

England, Scotland, Wales, Ireland, Isle of Man

##### Notes

The following populations have been given subspecies names: *B. pascuorum vulgo* in southern England and Wales; *B. pascuorum floralis* in Ireland; *B. pascuorum septentrionalis* in Scotland and Northern England.

#### Bombus (Thoracobombus) ?pomorum

(Panzer, 1805)

Bremus pomorum Panzer, 1805

##### Distribution

England

##### Notes

There is confusion surrounding the dates of capture of the supposedly British specimens of pomorum, collected near Deal in Kent (summarised by Alford 1975).

#### Bombus (Thoracobombus) ruderarius

(Müller, 1776)

Apis ruderaria Müller, 1776
derhamellus (Kirby, 1802, *Apis*)
raiellus (Kirby, 1802, *Apis*)

##### Distribution

England, Scotland, Wales, Ireland

#### Bombus (Thoracobombus) sylvarum

(Linnaeus, 1761)

Apis sylvarum Linnaeus, 1761
nigrescens Pérez, 1879
distinctus Vogt, 1909

##### Distribution

England, Scotland, Wales, Ireland

#### Eucerini

Latreille, 1802

#### Eucera

Scopoli, 1770

#### Eucera

Scopoli, 1770

#### Eucera (Eucera) longicornis

(Linnaeus, 1758)

Apis longicornis Linnaeus, 1758
linguaria (Fabricius, 1775, *Apis*)

##### Distribution

England, Wales

#### Eucera (Eucera) nigrescens

Pérez, 1879

tuberculata misident. [?]

##### Distribution

England

##### Notes

Probably extinct in Britain.

#### Melectini

Westwood, 1840

#### Melecta

Latreille, 1802

#### Melecta albifrons

(Forster, 1771)

Apis albifrons Forster, 1771
punctata (Fabricius, 1775, *Apis*)
armata (Panzer, 1799, *Andrena*) preocc.

##### Distribution

England, Wales

#### Melecta luctuosa

(Scopoli, 1770)

Apis luctuosa Scopoli, 1770

##### Distribution

England

##### Notes

Probably extinct in Britain.

#### Nomadinae

Latreille, 1802

#### Epeolini

Roberston, 1903

#### Epeolus

Latreille, 1802

#### Epeolus cruciger

(Panzer, 1799)

Nomada crucigera Panzer, 1799
rufipes Thomson, 1870

##### Distribution

England, Wales

#### Epeolus variegatus

(Linnaeus, 1758)

Apis variegata Linnaeus, 1758
notatus misident.
productus Thomson, 1870

##### Distribution

England, Wales, Scotland, Isle of Man

#### Nomadini

Latreille, 1802

#### Nomada

Scopoli, 1763

#### Nomada argentata

Herrich-Schäffer, 1839

atrata Smith, 1846

##### Distribution

England, Ireland

#### Nomada armata

Herrich-Schäffer, 1839

kirbyella Stephens, 1846

##### Distribution

England, Wales

#### Nomada baccata

Smith, 1844

alboguttata misident.

##### Distribution

England

#### Nomada conjungens

Herrich-Schäffer, 1839

##### Distribution

England

#### Nomada errans

Lepeletier, 1841

##### Distribution

England

#### Nomada fabriciana

(Linnaeus, 1767)

Apis fabriciana Linnaeus, 1767
fabriciella (Kirby, 1802, *Apis*)
quadrinotata (Kirby, 1802, *Apis*)

##### Distribution

England, Scotland, Wales, Ireland, Isle of Man

#### Nomada ferruginata

(Linnaeus, 1767)

Apis ferruginata Linnaeus, 1767
lateralis misident.
xanthosticta (Kirby, 1802, *Apis*)
bridgmaniana Smith, 1876

##### Distribution

England, Scotland, Wales

#### Nomada flava

Panzer, 1798

##### Distribution

England, Wales, Ireland

#### Nomada flavoguttata

(Kirby, 1802)

Apis flavoguttata Kirby, 1802
rufocincta (Kirby, 1802, *Apis*)

##### Distribution

England, Scotland, Wales, Ireland, Isle of Man

#### Nomada flavopicta

(Kirby, 1802)

Apis flavopicta Kirby, 1802
jacobaeae misident. [?]

##### Distribution

England, Wales

#### Nomada fucata

Panzer, 1798

##### Distribution

England, Wales

#### Nomada fulvicornis

Fabricius, 1793

lineola Panzer, 1798
sexcincta (Kirby, 1802, *Apis*)
caprea (Kirby, 1802, *Apis*)
cornigera (Kirby, 1802, *Apis*)
subcornuta (Kirby, 1802, *Apis*)

##### Distribution

England, Wales

#### Nomada goodeniana

(Kirby, 1802)

Apis goodeniana Kirby, 1802
succincta misident.
alternata (Kirby, 1802, *Apis*)

##### Distribution

England, Scotland, Wales, Ireland, Isle of Man

#### Nomada guttulata

Schenck, 1861

##### Distribution

England

#### Nomada hirtipes

Pérez, 1884

bucephalae Perkins, 1917

##### Distribution

England, Wales

#### Nomada integra

Brullé, 1832

ferruginata misident.
germanica misident.
pleurosticta misident.
stigma misident.
cinctiventris Friese, 1921

##### Distribution

England, Wales

#### Nomada lathburiana

(Kirby, 1802)

Apis lathburiana Kirby, 1802
rufiventris (Kirby, 1802, *Apis*)

##### Distribution

England, Wales

#### Nomada leucophthalma

(Kirby, 1802)

Apis leucophthalma Kirby, 1802
borealis Zetterstedt, 1838
inquilina Smith, 1844

##### Distribution

England, Scotland, Wales, Ireland, Isle of Man

#### Nomada marshamella

(Kirby, 1802)

Apis marshamella Kirby, 1802
alternata misident.

##### Distribution

England, Scotland, Wales, Ireland, Isle of Man

#### Nomada obtusifrons

Nylander, 1848

mistura Smith, 1851

##### Distribution

England, Scotland, Wales, Ireland, Isle of Man

#### Nomada panzeri

Lepeletier, 1841

ruficornis misident.

##### Distribution

England, Scotland, Wales, Ireland

#### Nomada roberjeotiana

Panzer, 1799

tormentillae Alfken, 1901

##### Distribution

England, Scotland, Wales, Isle of Man

#### Nomada ruficornis

(Linnaeus, 1758)

Apis ruficornis Linnaeus, 1758
bifida Thomson, 1872

##### Distribution

England, Scotland, Wales, Ireland, Isle of Man

#### Nomada rufipes

Fabricius, 1793

solidaginis misident.
picta (Kirby, 1802, *Apis*)
rufopicta (Kirby, 1802, *Apis*)

##### Distribution

England, Scotland, Wales, Ireland, Isle of Man

#### Nomada sexfasciata

Panzer, 1799

connexa (Kirby, 1802, *Apis*)
schaefferella (Kirby, 1802, *Apis*)

##### Distribution

England

#### Nomada sheppardana

(Kirby, 1802)

Apis sheppardana Kirby, 1802
furva misident.
dalii Curtis, 1832

##### Distribution

England, Wales

#### Nomada signata

Jurine, 1807

##### Distribution

England, Wales

#### Nomada striata

Fabricius, 1793

hillana (Kirby, 1802, *Apis*)
ochrostoma (Kirby, 1802, *Apis*)
vidua Smith, 1844

##### Distribution

England, Scotland, Wales, Ireland

#### Xylocopinae

Latreille, 1802

#### Ceratinini

Latreille, 1802

#### Ceratina

Latreille, 1802

#### Euceratina

Hirashima, Moure & Daly, 1971

#### Ceratina (Euceratina) cyanea

(Kirby, 1802)

Apis cyanea Kirby, 1802

##### Distribution

England

#### Xylocopini

Latreille, 1802

#### Xylocopa

Latreille, 1809

#### Xylocopa

Latreille, 1809

#### Xylocopa (Xylocopa) violacea

(Linnaeus, 1758)

Apis violacea Linnaeus, 1758

##### Distribution

England

#### Colletidae

Lepeletier, 1841

#### Colletinae

Lepeletier, 1841

#### Colletes

Latreille, 1802

#### Colletes cunicularius

(Linnaeus, 1761)

Apis cunicularia Linnaeus, 1761

##### Distribution

England, Wales

##### Notes

The British population has been described as a separate subspecies, *C. cunicularius celticus* O’Toole, 1974, and Else, Field & O’Toole (in Edwards 1997) suggest that the British population may be specifically distinct.

#### Colletes daviesanus

Smith, 1846

##### Distribution

England, Scotland, Wales, Ireland

#### Colletes floralis

Eversmann, 1852

montanus Morawitz, 1876

##### Distribution

England, Scotland, Ireland

#### Colletes fodiens

(Geoffroy, 1785)

Apis fodiens Geoffroy, 1785

##### Distribution

England, Scotland, Wales

#### Colletes halophilus

Verhoeff, 1944

##### Distribution

England

#### Colletes hederae

Schmidt & Westrich, 1993

##### Distribution

England

##### Notes

added by Cross (2002)

#### Colletes marginatus

Smith, 1846

##### Distribution

England, Wales

#### Colletes similis

Schenck, 1853

picistigma Thomson, 1872

##### Distribution

England, Wales, Ireland, Isle of Man

#### Colletes succinctus

(Linnaeus, 1758)

Apis succincta Linnaeus, 1758

##### Distribution

England, Scotland, Wales, Ireland, Isle of Man

#### Hylaeinae

Viereck, 1916

#### Hylaeus

Fabricius, 1793

#### Abrupta

Popov, 1939

#### Hylaeus (Abrupta) cornutus

Curtis, 1831

plantarius Smith, 1842

##### Distribution

England

#### Hylaeus

Fabricius, 1793

#### Hylaeus (Hylaeus) communis

Nylander, 1852

rupestris (Smith, 1872, *Prosopis*)

##### Distribution

England, Scotland, Wales, Ireland

#### Koptogaster

Alfken, 1912

#### Hylaeus (Koptogaster) punctulatissimus

Smith, 1842

##### Distribution

England

##### Notes

Probably extinct in Britain

#### Lamdopsis

Popov, 1939

#### Hylaeus (Lamdopsis) annularis

(Kirby, 1802)

Mellita annularis Kirby, 1802
euryscapus misident.
spilotus Forster, 1871
masoni (Saunders, 1894, *Prosopis*)

##### Distribution

England

##### Notes

Notton and Dathe (2008) designated as lectotype a female specimen that corresponds to the species here called *annularis*, with the species generally called *annularis* taking the name *dilatatus*.

#### Hylaeus (Lamdopsis) dilatatus

(Kirby, 1802)

Mellita dilatata Kirby, 1802
annularis misident.

##### Distribution

England, Wales

#### Paraprosopis

Popov, 1939

#### Hylaeus (Paraprosopis) pictipes

Nylander, 1852

varipes (Smith, 1853, *Prosopis*)

##### Distribution

England

#### Prosopis

Fabricius, 1804

NESOPROSOPIS Perkins, R.C.L., 1899

#### Hylaeus (Prosopis) brevicornis

Nylander, 1852

rubicola (Smith, 1869, *Prosopis*) preocc.

##### Distribution

England, Scotland, Wales, Ireland, Isle of Man

#### Hylaeus (Prosopis) confusus

Nylander, 1852

##### Distribution

England, Scotland, Wales, Ireland

##### Notes

The subspecies *perkinsi* Blüthgen, 1926, is recognised from Britain.

#### Hylaeus (Prosopis) incongruus

Förster, 1871

gibbus misident.
genalis Thomson, 1872

##### Distribution

England, Wales

#### Hylaeus (Prosopis) pectoralis

Förster, 1871

kriechbaumeri Förster, 1871
palustris (Perkins, 1900, *Prosopis*)

##### Distribution

England

#### Hylaeus (Prosopis) signatus

(Panzer, 1798)

Sphex signata Panzer, 1798

##### Distribution

England, Wales

#### Spatulariella

Popov, 1939

#### Hylaeus (Spatulariella) hyalinatus

Smith, 1842

##### Distribution

England, Scotland, Wales, Ireland, Isle of Man

#### Halictidae

Thomson, 1869

#### Halictinae

Thomson, 1869

#### Halictus

Latreille, 1804

#### Halictus

Latreille, 1804

#### Halictus (Halictus) eurygnathus

Blüthgen, 1931

quadricinctus misident.
tetrazonius misident.

##### Distribution

England

#### Halictus (Halictus) maculatus

Smith, 1848

##### Distribution

England

##### Notes

Probably extinct in Britain.

#### Halictus (Halictus) rubicundus

(Christ, 1791)

Apis rubicunda Christ, 1791
quadrifasciatus Smith, 1870
nesiotis Perkins, 1922

##### Distribution

England, Scotland, Wales, Ireland, Isle of Man

#### Seladonia

Robertson, 1918

#### Halictus (Seladonia) confusus

Smith, 1853

alpinus Alfken, 1907
confusus perkinsi Blüthgen, 1926
flavipes misident.

##### Distribution

England

#### Halictus (Seladonia) subauratus

(Rossi, 1792)

Apis subaurata Rossi, 1792
gramineus Smith, 1849

##### Notes

Probably extinct in Britain.

#### Halictus (Seladonia) tumulorum

(Linnaeus, 1758)

Apis tumulorum Linnaeus, 1758

##### Distribution

England, Scotland, Wales, Ireland

#### Lasioglossum

Curtis, 1833

##### Notes

Nomenclature for some species ('*Evylaeus*' *s. l.*) updated from Pesenko (2007) but, as Pesenko’s study was limited to the Palaearctic region, we do not follow his elevation of *Evylaeus* to a valid genus, nor have we adopted his numerous subgenera.

#### Dialictus

Robertson, 1902

#### Lasioglossum (Dialictus) cupromicans

(Pérez, 1903)

Halictus cupromicans Pérez, 1903

##### Distribution

England, Scotland, Wales, Ireland, Isle of Man

##### Notes

The Irish population has been described as the subspecies *hibernicum* Ebmer, 1970, with other populations referred to the subspecies *scoticum* Ebmer, 1970.

#### Lasioglossum (Dialictus) leucopus

(Kirby, 1802)

Melitta leucopus Kirby, 1802
aeratum (Kirby, 1802, *Melitta*)
semiaeneum (Brullé, 1832, *Halictus*)
viridaeneum (Blüthgen, 1918, *Halictus*)

##### Distribution

England, Scotland, Wales, Ireland, Isle of Man

#### Lasioglossum (Dialictus) morio

(Fabricius, 1793)

Hylaeus morio Fabricius, 1793

##### Distribution

England, Scotland, Wales, Isle of Man

#### Lasioglossum (Dialictus) smeathmanellum

(Kirby, 1802)

Melitta smeathmanella Kirby, 1802

##### Distribution

England, Scotland, Wales, Isle of Man

#### Hemihalictus

Cockerell, 1897

#### Lasioglossum (Hemihalictus) angusticeps

(Perkins, 1895)

Halictus angusticeps Perkins, 1895

##### Distribution

England

#### Lasioglossum (Hemihalictus) brevicorne

(Schenck, 1869)

Halictus brevicornis Schenck, 1869

##### Distribution

England

#### Lasioglossum (Hemihalictus) minutissimum

(Kirby, 1802)

Melitta minutissima Kirby, 1802
arnoldi (Saunders, 1910, *Halictus*)

##### Distribution

England, Wales, Ireland

#### Lasioglossum (Hemihalictus) nitidiusculum

(Kirby, 1802)

Melitta nitidiuscula Kirby, 1802

##### Distribution

England, Scotland, Wales, Ireland, Isle of Man

#### Lasioglossum (Hemihalictus) parvulum

(Schenck, 1853)

Hylaeus parvulus Schenck, 1853
minutum misident.

##### Distribution

England, Wales

#### Lasioglossum (Hemihalictus) pauperatum

(Brullé, 1832)

Halictus pauperatus Brullé, 1832
breviceps (Saunders, 1879, *Halictus*)

##### Distribution

England

#### Lasioglossum (Hemihalictus) punctatissimum

(Schenck, 1853)

Hylaeus punctatissimus Schenck, 1853
longiceps (Saunders, 1879, *Halictus*)

##### Distribution

England, Scotland, Wales, Ireland, Isle of Man

#### Lasioglossum (Hemihalictus) puncticolle

(Morawitz, 1872)

Halictus puncticollis Morawitz, 1872

##### Distribution

England

#### Lasioglossum (Hemihalictus) rufitarse

(Zetterstedt, 1838)

Halictus rufitarsis Zetterstedt, 1838
atricorne (Smith, 1870, *Halictus*)

##### Distribution

England, Scotland, Wales, Ireland

#### Lasioglossum (Hemihalictus) semilucens

(Alfken, 1914)

Halictus semilucens Alfken, 1914

##### Distribution

England

#### Lasioglossum (Hemihalictus) sexstrigatum

(Schenck, 1870)

Halictus sexstrigatus Schenck, 1870
sabulosum (Warncke, 1986, *Halictus*)

##### Distribution

England

##### Notes

added by Hawkins (2011)

#### Lasioglossum (Hemihalictus) villosulum

(Kirby, 1802)

Melitta villosula Kirby, 1802
punctulatum (Kirby, 1802, *Melitta*)

##### Distribution

England, Scotland, Wales, Ireland, Isle of Man

#### Lasioglossum

Curtis, 1833

#### Lasioglossum (Lasioglossum) laevigatum

(Kirby, 1802)

Melitta laevigata Kirby, 1802
lugubris (Kirby, 1802, *Melitta*)

##### Distribution

England, Wales

#### Lasioglossum (Lasioglossum) lativentre

(Schenck, 1853)

Hylaeus lativentris Schenck, 1853
decipiens (Perkins, 1913, *Halictus*)

##### Distribution

England, Wales

#### Lasioglossum (Lasioglossum) prasinum

(Smith, 1848)

Halictus prasinus Smith, 1848

##### Distribution

England, Wales

#### Lasioglossum (Lasioglossum) quadrinotatum

(Kirby, 1802)

Melitta quadrinotata Kirby, 1802

##### Distribution

England

#### Lasioglossum (Lasioglossum) sexnotatum

(Kirby, 1802)

Melitta sexnotata Kirby, 1802
nitidum misident.

##### Distribution

England

#### Lasioglossum (Lasioglossum) xanthopus

(Kirby, 1802)

Melitta xanthopus Kirby, 1802
tricingulum Curtis, 1833

##### Distribution

England, Wales

#### Leuchalictus

Warncke, 1975

#### Lasioglossum (Leuchalictus) leucozonium

(Schrank, 1781)

Apis leucozonia Schrank, 1781
similis (Smith, 1853, *Halictus*)

##### Distribution

England, Scotland, Wales, Ireland

#### Lasioglossum (Leuchalictus) zonulum

(Smith, 1848)

Halictus zonulus Smith, 1848

##### Distribution

England, Wales

#### Sphecodogastra

Ashmead, 1899

#### Lasioglossum (Sphecodogastra) albipes

(Fabricius, 1781)

Apis albipes Fabricius, 1781

##### Distribution

England, Scotland, Wales, Ireland, Isle of Man

#### Lasioglossum (Sphecodogastra) calceatum

(Scopoli, 1763)

Apis calceata Scopoli, 1763
cylindricum (Fabricius, 1793, *Hylaeus*)
fulvocinctum (Kirby, 1802, *Melitta*)
obovatum (Kirby, 1802, *Melitta*)

##### Distribution

England, Scotland, Wales, Ireland, Isle of Man

#### Lasioglossum (Sphecodogastra) fratellum

(Pérez, 1903)

Halictus fratellus Pérez, 1903
nigrum misident.
subfasciatum (Nylander, 1848, *Halictus*)
freygessneri (Alfken, 1905, *Halictus*)

##### Distribution

England, Scotland, Wales, Ireland

#### Lasioglossum (Sphecodogastra) fulvicorne

(Kirby, 1802)

Melitta fulvicornis Kirby, 1802
subfasciatum misident.

##### Distribution

England, Scotland, Wales

#### Lasioglossum (Sphecodogastra) laeve

(Kirby, 1802)

Melitta laevis Kirby, 1802

##### Distribution

England

##### Notes

Probably extinct in Britain.

#### Lasioglossum (Sphecodogastra) laticeps

(Schenck, 1869)

Halictus laticeps Schenck, 1869
semipunctulatum misident.

##### Distribution

England

#### Lasioglossum (Sphecodogastra) malachurum

(Kirby, 1802)

Melitta malachura Kirby, 1802
longulum (Smith, 1848, *Halictus*)

##### Distribution

England

#### Lasioglossum (Sphecodogastra) pauxillum

(Schenck, 1853)

Hylaeus pauxillus Schenck, 1853
immarginatum (Schenck, 1853, *Hylaeus*)

##### Distribution

England, Wales

#### Sphecodes

Latreille, 1804

#### Sphecodes crassus

Thomson, 1870

variegatus von Hagens, 1874

##### Distribution

England, Scotland, Wales

#### Sphecodes ephippius

(Linnaeus, 1767)

Sphex ephippia Linnaeus, 1767
divisus (Kirby, 1802, *Melitta*)
similis Wesmael, 1835

##### Distribution

England, Wales, Isle of Man

#### Sphecodes ferruginatus

von Hagens, 1882

##### Distribution

England, Wales

#### Sphecodes geoffrellus

(Kirby, 1802)

Melitta geofrella Kirby, 1802
affinis von Hagens, 1882
fasciatus von Hagens, 1882

##### Distribution

England, Scotland, Wales, Ireland, Isle of Man

#### Sphecodes gibbus

(Linnaeus, 1758)

Sphex gibba Linnaeus, 1758
picea (Kirby, 1802, *Melitta*)
sphecoides (Kirby, 1802, *Melitta*)

##### Distribution

England, Scotland, Wales

#### Sphecodes hyalinatus

von Hagens, 1882

##### Distribution

England, Scotland, Wales, Ireland

#### Sphecodes longulus

von Hagens, 1882

##### Distribution

England, Wales

#### Sphecodes miniatus

von Hagens, 1882

dimidiatus von Hagens, 1882

##### Distribution

England

#### Sphecodes monilicornis

(Kirby, 1802)

Melitta monilicornis Kirby, 1802
subquadratus Smith, 1845

##### Distribution

England, Scotland, Wales, Ireland, Isle of Man

#### Sphecodes niger

von Hagens, 1874

##### Distribution

England

#### Sphecodes pellucidus

Smith, 1845

pilifrons Thomson, 1870

##### Distribution

England, Scotland, Wales, Ireland

#### Sphecodes puncticeps

Thomson, 1870

##### Distribution

England, Wales

#### Sphecodes reticulatus

Thomson, 1870

##### Distribution

England, Wales

#### Sphecodes rubicundus

von Hagens, 1875

ruficrus misident.
rufiventris misident.

##### Distribution

England, Wales

#### Sphecodes scabricollis

Wesmael, 1835

##### Distribution

England, Wales

#### Sphecodes spinulosus

von Hagens, 1875

##### Distribution

England, Wales

#### Rophitinae

Schenck, 1866

#### Dufourea

Lepeletier, 1841

#### Dufourea halictula

(Nylander, 1852)

Rhophites halictulus Nylander, 1852

##### Distribution

England

##### Notes

Probably extinct in Britain.

#### Dufourea minuta

Lepeletier, 1841

vulgaris Schenck, 1861

##### Distribution

England

##### Notes

Probably extinct in Britain.

#### Rophites

Spinola, 1808

#### Rophites quinquespinosus

Spinola, 1808

##### Distribution

England

##### Notes

Probably extinct in Britain.

#### Megachilidae

Latreille, 1802

#### Megachilinae

Latreille, 1802

#### Anthidiini

Ashmead, 1899

#### Anthidium

Fabricius, 1804

#### Anthidium manicatum

(Linnaeus, 1758)

Apis manicata Linnaeus, 1758

##### Distribution

England, Scotland, Wales

##### Notes

Represented by the subspecies *nigrithorax* Dalla Torre, 1877

#### Stelis

Panzer, 1806

#### Stelis breviuscula

Nylander, 1848

##### Distribution

England

##### Notes

added by Else and Spooner (1987)

#### Stelis ornatula

(Klug, 1807)

Gyrodroma ornatula Klug, 1807
octomaculata Smith, 1843

##### Distribution

England, Wales

#### Stelis phaeoptera

(Kirby, 1802)

Apis phaeoptera Kirby, 1802

##### Distribution

England, Wales

#### Stelis punctulatissima

(Kirby, 1802)

Apis punctulatissima Kirby, 1802
aterrima (Panzer, 1798, *Apis*) preocc.

##### Distribution

England, Scotland, Wales

#### Megachilini

Latreille, 1802

#### Coelioxys

Latreille, 1809

#### Allocoelioxys

Tkalcu, 1974

#### Coelioxys (Allocoelioxys) afra

Lepeletier, 1841

##### Notes

Probably extinct in Britain.

#### Boreocoelioxys

Mitchell, 1973

#### Coelioxys (Boreocoelioxys) inermis

(Kirby, 1802)

Apis inermis Kirby, 1802
Coelioxys (Boreocoelioxys) inermis *acuminata* Nylander, 1852 preocc.

##### Distribution

England, Wales, Ireland

#### Coelioxys (Boreocoelioxys) mandibularis

Nylander, 1848

##### Distribution

England, Wales

#### Coelioxys

Latreille, 1809

#### Coelioxys (Coelioxys) conoidea

(Illiger, 1806)

Anthophora conoidea Illiger, 1806
vectis Curtis, 1831

##### Distribution

England, Wales, Ireland

#### Coelioxys (Coelioxys) elongata

Lepeletier, 1841

sponsa Smith, 1855

##### Distribution

England, Scotland, Wales, Ireland

#### Coelioxys (Coelioxys) quadridentata

(Linnaeus, 1758)

Apis quadridentata Linnaeus, 1758

##### Distribution

England, Wales

#### Coelioxys (Coelioxys) rufescens

Lepeletier & Serville, 1825

umbrina Smith, 1843

##### Distribution

England, Wales

#### Megachile

Latreille, 1802

#### Eutricharaea

Thomson, 1872

#### Megachile (Eutricharaea) leachella

Curtis, 1828

dorsalis Pérez, 1879 synonymy by Gusenleitner and Schwarz (2012)
argentata misident.

##### Distribution

England, Wales

#### Megachile

Latreille, 1802

#### Megachile (Megachile) centuncularis

(Linnaeus, 1758)

Apis centuncularis Linnaeus, 1758

##### Distribution

England, Scotland, Wales, Ireland, Isle of Man

#### Megachile (Megachile) lapponica

Thomson, 1872

##### Distribution

England

##### Notes

Probably extinct in Britain.

#### Megachile (Megachile) ligniseca

(Kirby, 1802)

Apis ligniseca Kirby, 1802

##### Distribution

England, Wales, Ireland

#### Megachile (Megachile) versicolor

Smith, 1844

##### Distribution

England, Scotland, Wales, Ireland, Isle of Man

##### Notes

Includes the subspecies *hiberniae* Perkins, 1925.

#### Pseudomegachile

Friese, 1899

#### Megachile (Pseudomegachile) ericetorum

(Lepeletier, 1841)

Megachile ericetorum Lepeletier, 1841
fasciata (Smith, 1844, *Megachile*)
rufitarsis (Smith, 1844, *Megachile*)

##### Distribution

England

##### Notes

Probably extinct in Britain.

#### Xanthosarus

Robertson, 1903

#### Megachile (Xanthosarus) circumcincta

(Kirby, 1802)

Apis circumcincta Kirby, 1802

##### Distribution

England, Scotland, Wales

#### Megachile (Xanthosarus) maritima

(Kirby, 1802)

Apis maritima Kirby, 1802

##### Distribution

England, Wales, Ireland, Isle of Man

#### Megachile (Xanthosarus) willughbiella

(Kirby, 1802)

Apis willughbiella Kirby, 1802

##### Distribution

England, Scotland, Wales, Ireland, Isle of Man

##### Notes

Includes the nominate subspecies and *hibernica* Perkins, 1925

#### Osmiini

Newman, 1834

#### Chelostoma

Latreille, 1809

#### Chelostoma campanularum

(Kirby, 1802)

Apis campanularum Kirby, 1802

##### Distribution

England

#### Chelostoma florisomne

(Linnaeus, 1758)

Apis florisomnis Linnaeus, 1758
maxillosum (Linnaeus, 1767, *Apis*)

##### Distribution

England, Wales

#### Heriades

Spinola, 1808

#### Heriades rubicola

Peréz, 1890

##### Distribution

England

##### Notes

added by Else (in prep.)

#### Heriades truncorum

(Linnaeus, 1758)

Apis truncorum Linnaeus, 1758

##### Distribution

England

#### Hoplitis

Klug, 1807

#### Alcidamea

Cresson, 1864

#### Hoplitis (Alcidamea) claviventris

(Thomson, 1872)

Osmia claviventris Thomson, 1872
leucomelana misident.

##### Distribution

England, Wales

#### Hoplitis (Alcidamea) leucomelana

(Kirby, 1802)

Apis leucomelana Kirby, 1802
parvula (Dufour & Perris, 1840, *Osmia*)

##### Distribution

England

##### Notes

Probably extinct in Britain.

#### Anthocopa

Lepeletier & Serville, 1825

#### Hoplitis (Anthocopa) spinulosa

(Kirby, 1802)

Apis spinulosa Kirby, 1802

##### Distribution

England, Wales

#### Osmia

Panzer, 1806

#### Helicosmia

Thomson, 1872

#### Osmia (Helicosmia) aurulenta

Panzer, 1799

##### Distribution

England, Scotland, Wales, Ireland

#### Osmia (Helicosmia) caerulescens

(Linnaeus, 1758)

Apis caerulescens Linnaeus, 1758
aenea (Linnaeus, 1761, *Apis*)

##### Distribution

England, Scotland, Wales

#### Osmia (Helicosmia) niveata

(Fabricius, 1804)

Apis niveata Fabricius, 1804
fulviventris (Panzer, 1798, *Apis*)

##### Distribution

England

#### Osmia (Helicosmia) leaiana

(Kirby, 1802)

Apis leaiana Kirby, 1802
fulviventris misident.

##### Distribution

England, Wales

#### Melanosmia

Schmiedeknecht, 1884

#### Osmia (Melanosmia) inermis

(Zetterstedt, 1838)

Anthophora inermis Zetterstedt, 1838
parietina misident.

##### Distribution

Scotland

#### Osmia (Melanosmia) parietina

Curtis, 1828

##### Distribution

England, Scotland, Wales

#### Osmia (Melanosmia) pilicornis

Smith, 1846

##### Distribution

England, Wales

#### Osmia (Melanosmia) uncinata

Gerstäcker, 1869

##### Distribution

Scotland

##### Notes

added by Else in Else and Spooner (1987)

#### Osmia (Melanosmia) xanthomelana

(Kirby, 1802)

Apis xanthomelana Kirby, 1802
atricapilla Curtis, 1828

##### Distribution

England, Wales

#### Neosmia

Tkalc, 1974

#### Osmia (Neosmia) bicolor

(Schrank, 1781)

Apis bicolor Schrank, 1781

##### Distribution

England, Wales

#### Osmia

Panzer, 1806

#### Osmia (Osmia) bicornis

(Linnaeus, 1758)

Apis bicornis Linnaeus, 1758
rufa (Linnaeus, 1758, *Apis*)
hedera Smith, 1844

##### Distribution

England, Scotland, Wales, Ireland

#### Melittidae

Schenck, 1860

#### Dasypodainae

Börner, 1919

##### Notes

According to Danforth et al. (2006), Dasypodainae may be better treated as a separate family, but this requires further corroboration.

#### Dasypoda

Latreille, 1802

#### Dasypoda hirtipes

(Fabricius, 1793)

Andrena hirtipes Fabricius, 1793
altercator (Harris, 1776, *Apis*) nom. dub.
plumipes (Panzer, 1797, *Andrena*)
swammerdamella (Kirby, 1802, *Melitta*)

##### Distribution

England, Wales

#### Macropidinae

Robertson, 1904

#### Macropis

Panzer, 1809

#### Macropis europaea

Warncke, 1973

labiata misident.

##### Distribution

England

#### Melittinae

Schenck, 1860

#### Melitta

Kirby, 1802

#### Cilissia

Leach, 1815

PSEUDOCILISSA Radoszkowski, 1891

#### Melitta (Cilissia) dimidiata

Morawitz, 1876

##### Distribution

England

#### Melitta (Cilissia) haemorrhoidalis

(Fabricius, 1775)

Andrena haemorrhoidalis Fabricius, 1775
chrysura Kirby, 1802

##### Distribution

England, Scotland, Wales, Isle of Man

#### Melitta

Kirby, 1802

#### Melitta (Melitta) leporina

(Panzer, 1799)

Apis leporina Panzer, 1799

##### Distribution

England, Wales

#### Melitta (Melitta) tricincta

Kirby, 1802

melanura (Nylander, 1852, *Kirbya*)

##### Distribution

England, Wales

### Chrysidoidea

#### CHRYSIDOIDEA

##### Notes

Some distribution records taken from Archer and Burn (2012). Fig. 3: habitus of selected British Chrysidoidea.

#### Bethylidae

Haliday, 1833

##### Notes

Taxonomy mostly follows Gordh and Móczár (1990). Distribution data from Perkins (1976).

#### Bethylinae

Haliday, 1833

#### Bethylus

Latreille, 1802

PERISEMUS Förster, 1856
ANOXUS Thomson, 1862 synonymy by Polaszek and Krombein (1994)
EPISEMUS Thomson, 1862
DIGONIOZUS Kieffer, 1905

#### Bethylus boops

(Thomson, 1862)

Anoxus boops Thomson, 1862

##### Distribution

England

##### Notes

added by Burn (1997)

#### Bethylus cephalotes

(Förster, 1860)

Perisemus cephalotes Förster, 1860
fuscicornis var. tibialis Kieffer, 1905

##### Distribution

England, Scotland, Wales, Isle of Man

#### Bethylus dendrophilus

Richards, 1939

##### Distribution

England

#### Bethylus fuscicornis

(Jurine, 1807)

Omalus fuscicornis Jurine, 1807
sygenesiae Haliday, 1834
fulvicornis Curtis, 1838
triareolatus Förster, 1851
variabilis (Thomson, 1862, *Episemus*)
hyalinus (Marshall, 1874, *Perisemus*) synonymy by Rond (1994)
fuscicornis var. maurus Kieffer, 1905
brevipennis Hellén, 1920
berlandi Arle, 1929

##### Distribution

England, Scotland, Wales

#### Goniozus

Förster, 1856

PARASIEROLA Cameron, 1883
PROGONIOZUS Kieffer, 1905
PERISIEROLA Kieffer, 1914

#### Goniozus claripennis

(Förster, 1851)

Bethylus claripennis Förster, 1851
fuscipennis (Förster, 1851, *Bethylus*)
distigmus Thomson, 1862
audouinii Westwood, 1874
claripennis var. fuscipennis Kieffer, 1905 preocc.
claripennis var. tibialis Kieffer, 1905

##### Distribution

England

#### Epyrinae

Kieffer, 1914

#### Cephalonomia

Westwood, 1833

HOLOPEDINA Förster, 1850
CEPHALOMIA Kirchner, 1867

#### Cephalonomia formiciformis

Westwood, 1833

polypori (Förster, 1850, *Holopedina*)
brevipennis Kieffer, 1906
formiciformis var. sulcata Kieffer, 1906

##### Distribution

England, Ireland

##### Notes

*Cephalonomia brevipennis* is listed as a separate species (described from English specimens, now missing) by Gordh and Móczár (1990) but we follow Richards (1939) who believed this not to be a separate species from *formiciformis*.

#### # Cephalonomia gallicola

(Ashmead, 1887)

Sclerochroa gallicola Ashmead, 1887
nubilipennis (Ashmead, 1887, *Holopedina*)
xambeui Girad, 1898
quadridentata Duchaussoy, 1920
strandi Hoffer, 1936

#### Cephalonomia hammi

Richards, 1939

##### Distribution

England

#### # Cephalonomia tarsalis

(Ashmead, 1893)

Ateleopterus tarsalis Ashmead, 1893
carinata Kieffer, 1907
meridionalis Brethes, 1913
kiefferi Fouts, 1920

#### # Cephalonomia waterstoni

Gahan, 1931

#### Epyris

Westwood, 1832

DOLUS Motshultsky, 1863
MUELLERELLA Saussure, 1892
HOMOGLENUS Kieffer, 1904 synonymy by Terayama (2003)
PAREPYRIS Kieffer, 1913
PSILEPYRIS Kieffer, 1913
ARTIEPYRIS Kieffer, 1913

#### Epyris bilineatus

Thomson, 1862

fraternus Westwood, 1874
saeva Westwood, 1874
multidentatus Keiffer, 1906
multidentatus var. angustipennis Kieffer, 1906

##### Distribution

England

#### Epyris niger

Westwood, 1832

##### Distribution

England

#### [? Epyris tricolor

Cameron, 1888]

##### Notes

Supposedly described from the New Forest, the type is lost and the species has not been satisfactorily interpreted since.

#### Holepyris

Kieffer, 1905

MISEPYRIS Kieffer, 1913
PAREPYRIS Brethes, 1913

#### # Holepyris glabratus

(Fabricius, 1798)

Tiphia glabrata Fabricius, 1798
hawaiiensis (Ashmead, 1901, *Holepyris*)

#### # Holepyris sylvanidis

(Brethes, 1913)

Parepyris sylvanidis Brethes, 1913
zeae (Turner & Waterston, 1921, *Rhabdepyris*)

#### Laelius

Ashmead, 1893

##### Notes

see Notton et al. (2014) for synonymy and distribution

#### Laelius femoralis

(Förster, 1860)

Bethylus femoralis Förster, 1860
microneurus (Kieffer, 1906, *Allepyris*)
nigricrus (Kieffer, 1906, *Allepyris*)

##### Distribution

England

#### # Laelius pedatus

(Say, 1836)

Bethylus pedatus Say, 1836

##### Distribution

England

##### Notes

Added by Notton et al. (2014); probably an accidental introduction.

#### Plastanoxus

Kieffer, 1905

SNAPPANIA Hedqvist, 1975

#### Plastanoxus chittendenii

(Ashmead, 1893)

Anoxus chittendenii Ashmead, 1893

##### Distribution

England

#### Plastanoxus # munroi

Richards, 1939

#### Plastanoxus # westwoodi

(Kieffer, 1914)

Cephalonomia westwoodi Kieffer, 1914
kiefferi Gahan, 1931

#### Pristocerinae

Kieffer, 1914

#### Pristocera

Klug, 1808

ACREPYRIS Kieffer, 1905
MANGESIA Kieffer, 1911
TRICHELOBRACHIUM Kieffer, 1914

#### Pristocera depressa

(Fabricius, 1805)

Bethylus depressus Fabricius, 1805
roubali (Menozzi, 1925, *Pseudisobrachium*)

##### Distribution

England

#### Pseudisobrachium

Kieffer, 1904

MONEPYRIS Kieffer, 1905
XESTOBETHYLUS Cameron, 1909
PLUTOBETHYLUS Kieffer, 1910
LYSSEPYRIS Kieffer, 1913
XANTEPYRIS Kieffer, 1913
PARISOBRACHIUM Kieffer, 1914
AFRISOBRACHIUM Benoit, 1957
EDAPHOLIGON Oglobin, 1963

#### Pseudisobrachium subcyaneum

(Haliday, 1838)

Epyris subcyaneus Haliday, 1838
halidaii (Westwood, 1874, *Epyris*)
carpentieri Kieffer, 1906
carpentieri var. septemfasciatum Kieffer, 1906
cantianum Chitty, 1906
concolor Kieffer, 1906

##### Distribution

England

#### Chrysididae

Latreille, 1802

#### Cleptinae

Morice, 1900

#### Cleptes

Latreille, 1802

#### Cleptes

Latreille, 1802

#### Cleptes (Cleptes) semiauratus

(Linnaeus, 1761)

Sphex semiaurata Linnaeus, 1761
auratus (Panzer, 1798, *Ichneumon*)
pallipes Lepeletier, 1805
diana Mocsáry, 1889

##### Distribution

England, Wales

##### Notes

Synonymy follows Rosa et al. (2015).

#### Leiocleptes

Móczár, 1962

#### Cleptes (Leiocleptes) nitidulus

(Fabricius, 1793)

Ichneumon nitidulus Fabricius, 1793

##### Distribution

England

#### Chrysidinae

Latreille, 1802

##### Notes

Some distribution data from Morgan (1984).

#### Chrysidini

Latreille, 1802

#### Chrysis

Linnaeus, 1761

##### Notes

The number of species recognised in the *Chrysis ignita* group has varied from author to author. Soon et al. (2014) analysed species limits and found there to be 15 described European species plus cryptic species, a result more in line with Morgan’s (Morgan 1984) recognition of multiple species than, for example, Kunz’s (Kunz 1994) much more conservative treatment.

#### Chrysis angustula

Schenck, 1856

brevidens Tournier, 1879

##### Distribution

England, Scotland, Wales

#### Chrysis corusca

Valkeila, 1971

##### Distribution

England

##### Notes

added by Soon et al. (2014)

#### Chrysis fulgida

Linnaeus, 1761

##### Distribution

England

#### Chrysis gracillima

Förster, 1853

saussurei Chevrier, 1862

##### Distribution

England

##### Notes

added by Morgan (1984)

#### Chrysis ignita

(Linnaeus, 1758)

Sphex ignita Linnaeus, 1758

##### Distribution

England, Scotland, Wales, Ireland, Isle of Man

#### Chrysis illigeri

Wesmael, 1839

chrysoprasina Hellén, 1919 preocc.
helleni Linsenmaier, 1959
succincta misident.

##### Distribution

England

#### Chrysis impressa

Schenck, 1856

##### Distribution

England, Scotland, Wales, Ireland, Isle of Man

#### Chrysis longula

Abeille de Perrin, 1879

##### Distribution

England, Scotland

#### Chrysis mediata

Linsenmaier, 1951

##### Distribution

England, Wales, Ireland

#### Chrysis pseudobrevitarsis

Linsenmaier, 1951

##### Distribution

England

#### Chrysis ruddii

Shuckard, 1836

auripes Wesmael, 1839

##### Distribution

England, Scotland, Wales, Isle of Man

#### Chrysis schencki

Linsenmaier, 1968

schenckiana Linsenmaier, 1959 preocc.

##### Distribution

England

#### Chrysis terminata

Dahlbom, 1854

##### Distribution

England

##### Notes

added by Soon et al. (2014)

#### Chrysis vanlithi

Linsenmaier, 1959

rutiliventris misident.

##### Distribution

England, Scotland, Wales, Ireland, Isle of Man

#### Chrysis viridula

Linnaeus, 1761

bidentata Linnaeus, 1767

##### Distribution

England, Wales

#### Chrysura

Dahlbom, 1845

#### Chrysura hirsuta

(Gerstäcker, 1869)

Chrysis hirsuta Gerstäcker, 1869
osmiae (Thomson, 1870, *Chrysis*)

##### Distribution

Scotland

#### Chrysura radians

(Harris, 1776)

Chrysis radians Harris, 1776
pustulosa (Abeille de Perrin, 1878, *Chrysis*)

##### Distribution

England, Wales

#### Pseudospinolia

Linsenmaier, 1951

#### Pseudospinolia neglecta

(Shuckard, 1836)

Chrysis neglecta Shuckard, 1836

##### Distribution

England, Wales

#### Trichrysis

Lichtenstein, 1876

#### Trichrysis cyanea

(Linnaeus, 1758)

Sphex cyanea Linnaeus, 1758

##### Distribution

England, Scotland, Wales

#### Elampini

Dahlbom, 1854

#### Elampus

Spinola, 1806

NOTOZUS Förster, 1853

#### Elampus panzeri

(Fabricius, 1804)

Chrysis panzeri Fabricius, 1804
scutellaris (Panzer, 1798, *Chrysis*) preocc.
constrictus misident.

##### Distribution

England

#### Hedychridium

Abeille de Perrin, 1878

#### Hedychridium ardens

(Latreille, 1801)

Chrysis ardens Latreille, 1801

##### Distribution

England, Scotland, Wales, Ireland, Isle of Man

#### Hedychridium coriaceum

(Dahlbom, 1854)

Hedychrum coriaceum Dahlbom, 1854

##### Distribution

England

#### Hedychridium cupreum

(Dahlbom, 1845)

Hedychrum cupreum Dahlbom, 1845
integrum (Dahlbom, 1854, *Hedychrum*) synonymy by Morgan (1984)

##### Distribution

England, Wales

#### Hedychridium roseum

(Rossi, 1790)

Chrysis rosea Rossi, 1790

##### Distribution

England

#### Hedychrum

Latreille, 1802

#### Hedychrum niemelai

Linsenmaier, 1959

nobile misident.

##### Distribution

England

#### Hedychrum nobile

(Scopoli, 1763)

Sphex nobilis Scopoli, 1763
lucidula (Fabricius, 1775, *Chrysis*)
regia (Fabricius, 1793, *Chrysis*)

##### Distribution

England

##### Notes

added by Baldock and Hawkins (2013); tentative identification but subsequently confirmed (e.g. Earwaker and Sutton 2015).

#### Hedychrum rutilans

Dahlbom, 1854

intermedium misident.

##### Distribution

England

#### Omalus

Panzer, 1801

#### Omalus aeneus

(Fabricius, 1787)

Chrysis aenea Fabricius, 1787

##### Distribution

England, Wales

#### Omalus puncticollis

(Mocsáry, 1887)

Ellampus puncticollis Mocsáry, 1887

##### Distribution

England, Scotland, Wales

##### Notes

Treated as a subspecies of *aeneus* in Fauna Europaea.

#### Philoctetes

Abeille de Perrin, 1879

#### Philoctetes truncatus

(Dahlbom, 1831)

Chrysis truncata Dahlbom, 1831

##### Distribution

England

#### Pseudomalus

Ashmead, 1902

#### Pseudomalus auratus

(Linnaeus, 1758)

Sphex aurata Linnaeus, 1758

##### Distribution

England, Scotland, Wales, Ireland

#### Pseudomalus violaceus

(Scopoli, 1763)

Sphex violacea Scopoli, 1763

##### Distribution

England, Wales

#### Dryinidae

Haliday, 1833

##### Notes

Some distributional data and synonymy from Olmi (1984), supplemented by Perkins (1976) and Olmi (1989).

#### Anteoninae

Perkins, 1912

#### Anteon

Jurine, 1807

CHELOGYNUS Haliday, 1838
NEOCHELOGYNUS Perkins, 1905

#### Anteon arcuatum

Kieffer, 1905

imberbis Kieffer, 1905
bensoni Richards, 1939
jurineanum in part, Perkins, 1976

##### Distribution

England, Scotland, Wales, Ireland

#### Anteon brachycerum

(Dalman, 1823)

Dryinus brachycerus Dalman, 1823
lyde (Walker, 1837, *Dryinus*)
nigricornis Kieffer, 1905
triareolatus Kieffer, 1905
brevicollis Kieffer, 1905
flavitarsis Kieffer, 1905
indivisus Kieffer, 1905
nigroclavatus Kieffer, 1905
curvatus Kieffer, 1906
obscuricornis Kieffer, 1906
suffolciensis (Chitty, 1908, *Antaeon*)
curvus Kieffer, 1914

##### Distribution

England, Scotland, Wales, Ireland

#### Anteon ephippiger

(Dalman, 1818)

Gonatopus ephippiger Dalman, 1818
collaris (Dalman, 1818, *Gonatopus*)
facialis (Thomson, 1860, *Dryinus*)
albidicollis Kieffer, 1905
rubrifrons Kieffer, 1905
rufovariegatus (Berland, 1928, *Chelogynus*)
albidocolle Richards, 1939
pyonganensis Moczar, 1983

##### Distribution

England, Scotland, Wales, Ireland

#### Anteon exiguum

(Haupt, 1941)

Chelogynus exiguus Haupt, 1941
subarcticus Hellén, 1935 nom. nud.
flaviscapus Jansson, 1950
subarcticus Hellén, 1953

##### Distribution

England, Wales

##### Notes

added by Burn (1995)

#### Anteon faciale

(Thomson, 1860)

Dryinus facialis Thomson, 1860
pseudohilare Burn, 1990

##### Distribution

England

#### Anteon flavicorne

(Dalman, 1818)

Gonatopus flavicornis Dalman, 1818
sericeus Kieffer, 1905
subflavicornis Haupt, 1941

##### Distribution

England, Scotland, Wales, Ireland

#### Anteon fulviventre

(Haliday, 1828)

Dryinus fulviventris Haliday, 1828
fuscipes (Thomson, 1860, *Dryinus*)
similis Kieffer, 1905
gracilicollis Kieffer, 1905
flavinervis Kieffer, 1905
parvulus Kieffer, 1905
xanthostigma Kieffer, 1905
flaviscapus Kieffer, 1905
parvus Kieffer, 1906
alutaceus (Richards, 1935, *Chelogynus*)

##### Distribution

England, Scotland, Wales, Ireland, Isle of Man

#### Anteon gaullei

Kieffer, 1905

cameroni Kieffer, 1905
trivialis Kieffer, 1905
rufulocollis (Chitty, 1908, *Antaeon*)

##### Distribution

England, Scotland, Wales, Ireland

#### Anteon infectum

(Haliday, 1837)

Dryinus infectus Haliday, 1837
luteicornis misident. *sensu*Richards (1939) and Perkins (1976)
inclytus (Haliday, 1837, *Dryinus*)
lateralis (Thomson, 1860, *Dryinus*)
fusiformis Kieffer, 1905
punctatus Kieffer, 1905
ellimani (Chitty, 1908, *Antaeon*)

##### Distribution

England, Scotland, Ireland

#### Anteon jurineanum

Latreille, 1809

brevicornis (Dalman, 1818, *Gonatopus*)
cursor (Haliday, 1828, *Dryinus*)
otiartes (Walker, 1837, *Dryinus*)
sisithrus (Walker, 1837, *Dryinus*)
nanus (Haliday, 1837, *Dryinus*)
crenulatus Kieffer, 1905
thomsoni Kieffer, 1905
vicinus Kieffer, 1905
marginatus Kieffer, 1905
rectus Kieffer, 1905
scoticus Kieffer, 1905
barbatus (Chitty, 1908, *Antaeon*)
brunneipes (Berland, 1928, *Xenanteon*)
nec jurineanum Richards, 1939
nec jurineanum Perkins, 1976

##### Distribution

England, Scotland, Wales, Ireland

#### Anteon pubicorne

(Dalman, 1818)

Gonatopus pubicornis Dalman, 1818
tenuicornis (Dalman, 1823, *Dryinus*)
cephalotes (Ljungh, 1824, *Gonatopus*)
lucidus (Haliday, 1828, *Dryinus*)
penidas (Walker, 1837, *Dryinus*)
alorus (Walker, 1837, *Dryinus*)
fuscoclavatus Kieffer, 1905
triangularis Kieffer, 1905
divisus Kieffer, 1905
vulgaris Kieffer, 1905
breviventralis (Chitty, 1908, *Antaeon*)
delicatulus (Chitty, 1908, *Antaeon*)
serratus (Maneval, 1935, *Chelogynus*)
exiguus (Haupt, 1941, *Chelogynus*)
mongolicum Moczar, 1983

##### Distribution

England, Scotland, Wales, Ireland

##### Notes

Included as a synonym of *brachycerum* by Richards (1978).

#### Anteon reticulatum

Kieffer, 1905

##### Distribution

England

##### Notes

added by Olmi (1989)

#### Anteon scapulare

(Haliday, 1837)

Dryinus scapularis Haliday, 1837
longiforceps Kieffer, 1905
carinatus Kieffer, 1905
lanionis (Haupt, 1941, *Chelogynus*)

##### Distribution

England

#### Anteon tripartitum

Kieffer, 1905

tricarinatus Kieffer, 1905
kiefferi (Chitty, 1908, *Antaeon*)
angusticollis (Berland, 1928, *Chelogynus*)
prehensor (Maneval, 1935, *Chelogynus*)
berlandi (Richards, 1936, *Chelogynus*)
silvaticus (Ponomarenko, 1970, *Chelogynus*)

##### Distribution

England, Scotland, Ireland

#### Lonchodryinus

Kieffer, 1905

PRENANTEON Kieffer, 1913
PSILANTEON Kieffer, 1913

#### Lonchodryinus ruficornis

(Dalman, 1818)

Gonatopus ruficornis Dalman, 1818
basalis (Dalman, 1818, *Gonatopus*)
frontalis (Dalman, 1818, *Gonatopus*)
fuscicornis (Dalman, 1818, *Gonatopus*)
longicornis (Dalman, 1823, *Dryinus*)
crassimanus (Haliday, 1828, *Dryinus*)
daos (Walker, 1837, *Dryinus*)
ilus (Walker, 1837, *Dryinus*)
misor (Walker, 1837, *Dryinus*)
lapponicus (Thomson, 1860, *Dryinus*)
retusus (Thomson, 1860, *Dryinus*)
lepidus (Förster, 1861, *Chelogynus*)
subapterus (Kieffer, 1905, *Anteon*)
aequalis (Kieffer, 1905, *Anteon*)
melanocera (Kieffer, 1905, *Anteon*)
vitellinipes (Kieffer, 1905, *Anteon*)
procericornis (Kieffer, 1905, *Anteon*)
declivis (Kieffer, 1905, *Anteon*)
pallidinervis (Kieffer, 1905, *Anteon*)
integer (Kieffer, 1905, *Anteon*)
curvinervis (Kieffer, 1905, *Anteon*)
fractinervis (Kieffer, 1905, *Anteon*)
hyalinipennis (Kieffer, 1905, *Anteon*)
longifilis (Kieffer, 1906, *Anteon*)
halidayi (Kieffer, 1906, *Anteon*)
morleyi (Chitty, 1908, *Antaeon*)
luffnessensis (Chitty, 1908, *Antaeon*)
beaumonti (Chitty, 1908, *Antaeon*)
walkeri (Kieffer, 1914, *Chelogynus*)
parcepunctatus (Kieffer, 1914, *Prenanteon*)
palustris (Oglobin, 1924, *Prenanteon*)
filicornis (Oglobin, 1924, *Prenanteon*)
euscelisi (Haupt, 1941, *Prenanteon*)
semenovi (Ponomarenko, 1970, *Prenanteon*)
foveatus (Richards, 1971, *Prenanteon*)
pektusanense (Moczar, 1983, *Prenanteon*)
clavatum (Moczar, 1983, *Prenanteon*)

##### Distribution

England, Scotland, Wales, Ireland, Isle of Man

#### Aphelopinae

Perkins, 1912

#### Aphelopus

Dalman, 1823

ANTAPHELOPUS Benoit, 1951
GYMNAPHELOPUS Benoit, 1951

##### Notes

Data on five species reared in Wales from Jervis (1977).

#### Aphelopus atratus

(Dalman, 1823)

Dryinus atratus Dalman, 1823
holomelas Richards, 1939

##### Distribution

England, Scotland, Wales, Ireland

#### Aphelopus camus

Richards, 1939

heidelbergensis Richards, 1939

##### Distribution

England, Wales

#### Aphelopus melaleucus

(Dalman, 1818)

Gonatopus melaleucus Dalman, 1818
albipes (Ratzeburg, 1848, *Ceraphron*)

##### Distribution

England, Scotland, Wales, Ireland

#### Aphelopus nigriceps

Kieffer, 1905

##### Distribution

England, Scotland, Wales, Ireland

#### Aphelopus querceus

Olmi, 1984

##### Distribution

England

##### Notes

added by Burn (1995)

#### Aphelopus serratus

Richards, 1939

##### Distribution

England, Scotland, Wales, Ireland

#### Bocchinae

Richards, 1939

#### Mystrophorus

Förster, 1856

#### Mystrophorus formicaeformis

Ruthe, 1859

##### Distribution

England

#### Dryininae

Kieffer, 1906

#### Dryinus

Latreille, 1804

CAMPYLONYX Westwood, 1835
PARADRYINUS Perkins, 1905
CHLORODRYINUS Perkins, 1905
PLASTODRYINUS Kieffer, 1906
MESODRYINUS Kieffer, 1906
HESPERODRYINUS Perkins, 1907

#### Dryinus collaris

(Linnaeus, 1767)

Sphex collaris Linnaeus, 1767
formicarius Latreille, 1805
ampuliciformis (Westwood, 1835, *Campylonyx*)
corsicae (Kieffer, 1914, *Lestodryinus*)

##### Distribution

England

#### Dryinus niger

Kieffer, 1904

brittanicus (Richards, 1939, *Mesodryinus*)

##### Distribution

England

#### Gonatopodinae

Kieffer, 1906

#### Gonatopus

Ljungh, 1810

DICONDYLUS Haliday, 1829-30
LABEO Haliday, 1833
PSEUDOGONATOPUS Perkins, 1905
NEOGONATOPUS Perkins, 1905
PACHYGONATOPUS Perkins, 1905
CHALCOGONATOPUS Perkins, 1905
EUGONATOPUS Perkins, 1905
PLATYGONATOPUS Kieffer, 1906
AGONATOPUS Perkins, 1907
AGONATOPOIDES Perkins, 1907
EUCAMPTONYX Perkins, 1907
CYRTOGONATOPUS Kieffer, 1907
DIGONATOPUS Kieffer, 1913
LABERIUS Kieffer, 1914
TRICHOGONATOPUS Hellén, 1930
METAGONATOPUS Oglobin, 1932
ALLOGONATOPUS Haupt, 1938
DONISTHORPINA Richards, 1939
PLECTROGONATOPUS Richards, 1939
TETRODONTOCHELYS Richards, 1939
EPIGONATOPOIDES Richards, 1939
RHYNCHOGONATOPUS Benoit, 1953
MADECAGONATOPUS Benoit, 1953
CYRTOGONATOPOIDES Ponomarenko, 1966
MEGAGONATOPUS Olmi & Currado, 1976
TETRADONTOCHELYS Perkins, 1976

#### Gonatopus albosignatus

Kieffer, 1905

separatus (Richards, 1939, *Pseudogonatopus*) synonymy by Burn and Olmi (2011)

##### Distribution

England, Wales

#### Gonatopus bicolor

(Haliday, 1828)

Dryinus bicolor Haliday, 1828
vitripennis (Haliday, 1833, *Labeo*)
excisus (Westwood, 1833, *Antaeon*)
conjunctus Kieffer, 1905
bifarius Kieffer, 1906
decretorius Haupt, 1916
lindbergi (Heikinheimo, 1957, *Dicondylus*)

##### Distribution

England, Scotland, Wales, Ireland

#### Gonatopus clavipes

(Thunberg, 1827)

Gelis clavipes Thunberg, 1827
sepsoides Westwood, 1833
pilosus Thomson, 1860
nigerrimus (Förster, 1861, *Labeo*)
pusillus Szépligeti, 1901, *Labeo*)
hispanicus Kieffer, 1905
sociabilis Kieffer, 1907
borealis Sahlberg, 1910
wagneri Strand, 1919
barbatellus Richards, 1939
campestris Ponomarenko, 1965
rhaensis Ponomarenko, 1970

##### Distribution

England, Scotland, Wales, Ireland

#### Gonatopus distinctus

Kieffer, 1905

septemdentatus Sahlberg, 1910
robustus (Ceballos, 1927, *Dicondylus*)

##### Distribution

England, Wales

#### Gonatopus distinguendus

Kieffer, 1905

flavicornis Thomson, 1860 preocc.
luteicornis Kieffer, 1905
excavatus Sahlberg, 1910
liechtensteini Picard, 1932
procerus (Haupt, 1938, *Allogonatopus*)
thomsoni Hellén, 1953
rossicus Ponomarenko, 1965

##### Distribution

England, Wales, Ireland

#### Gonatopus formicicolus

(Richards, 1939)

Donisthorpina formicicola Richards, 1939

##### Distribution

England, Wales

#### Gonatopus helleni

Raatikainen, 1961

dichromus Kieffer, 1906
rufescens Hellén, 1935

##### Distribution

England

##### Notes

added by Burn (1997)

#### Gonatopus lunatus

Klug, 1810

bifasciatus Kieffer, 1904
gracilicornis Kieffer, 1904
filicornis Kieffer, 1905
gracilis Kieffer, 1905
marshalli Kieffer, 1905
myrmecophilus Kieffer, 1905
gracilipes Kieffer, 1906
raptoripes Strand, 1919

##### Distribution

England, Wales

#### Gonatopus pedestris

Dalman, 1818

ljunghii Westwood, 1833
leucostomus Sahlberg, 1910
arnoldii (Ponomarenko, 1966, *Pachygonatopus*)

##### Distribution

England

#### Gonatopus striatus

Kieffer, 1905

richardsi (Moczar, 1965, *Plectrogonatopus*)
tauricus (Ponomarenko, 1965, *Agonatopoides*)

##### Distribution

England, Scotland, Wales, Ireland

#### Haplogonatopus

Perkins, 1905

MONOGONATOPUS Richards, 1939

#### Haplogonatopus oratorius

(Westwood, 1833)

Gonatopus oratorius Westwood, 1833

##### Distribution

England

#### Embolemidae

Förster, 1856

#### Embolemus

Westwood, 1833

MYRMECOMORPHUS Westwood, 1833
POLYPLANUS Nees, 1834
EMBOLIMUS Agassiz, 1846
FORMILA De Romand, 1846
PEDINOMMA Förster, 1856
AMPULICOMORPHA Ashmead, 1893
AMPULICIMORPHA Brues, 1933

#### Embolemus ruddii

(Westwood, 1833)

Myrmecomorphus ruddii Westwood, 1833
rufescens (Westwood, 1833, *Myrmecomorphus*)
sickershusanus (Nees, 1834, *Polyplanus*)
antennalis (Kieffer, 1906, *Pedinomma*) synonymy by Hilpert (1989)
holochlora (Kieffer, 1906, *Pedinomma*)
hypochlora (Kieffer, 1906, *Pedinomma*)
rufus Kieffer, 1906

##### Distribution

England, Scotland, Wales

### Vespoidea

#### VESPOIDEA

##### Notes

Some distribution data for Mutillidae, Sapygidae, Tiphiidae and Vespidae from Archer (2014). Figs 4, 5, 6: habitus of selected British Vespoidea.

#### Formicidae

Latreille, 1809

##### Notes

Nomenclature, synonymy and higher classification based on Bolton (1995), Bolton (2003) and AntWeb, with additional references given.

#### Dolichoderinae

Forel, 1878

##### Notes

[LINEPITHEMA Mayr, 1866 # *humile* (Mayr, 1868, *Hypoclinea*)] Although this highly invasive species has the potential to establish itself in hothouses, given the correct climate, we are unaware of any established colonies being found in Britain. Any introductions have been of isolated workers.

#### Tapinoma

Förster, 1850

MICROMYRMA Dufour, 1857
SEMONIUS Forel, 1910
TAPINOPTERA Santschi, 1925
ZATAPINOMA Wheeler, 1928
NEOCLYSTOPSENELLA Kurian, 1955

#### Tapinoma erraticum

(Latreille, 1798)

Formica erratica Latreille, 1798
caerulescens (Losana, 1834, *Formica*)
glabrella (Nylander, 1849, *Formica*)
collina Förster, 1850
bononiensis Emery, 1925
breve Emery, 1925
tauridis Emery, 1925
transcaucasica Karavaiev, 1927

##### Distribution

England

#### Tapinoma subboreale

Seifert, 2012

ambiguum misident.
madeirense misident.

##### Distribution

England

##### Notes

Seifert (2012) clarified the identity of the north European *Tapinoma*.

#### # Tapinoma melanocephalum

(Fabricius, 1793)

Formica melanocephala Fabricius, 1793
nana (Jerdon, 1851, *Formica*) preocc.
pellucida (Smith, 1857, *Myrmica*)
familiaris (Smith, 1860, *Formica*) preocc.
australe Santschi, 1928
australis Santschi, 1928

#### Formicinae

Latreille, 1802

#### Formica

Linnaeus, 1758

FORMICINA Schuckard, 1840
NEOFORMICA Wheeler, 1913
RAPTIFORMICA Forel, 1913
SERVIFORMICA Forel, 1913
COPTOFORMICA Müller, 1923
ADFORMICA Lomnicki, 1925

#### Formica aquilonia

Yarrow, 1955

schmidti Ruzsky, 1920 preocc.

##### Distribution

Scotland, Ireland

#### Formica cunicularia

Latreille, 1798

fuscorufibarbis Forel, 1874
glauca Ruzsky, 1896
rubescens Forel, 1904
caucasica Wheeler, 1913
volgensis Ruzsky, 1914
katuniensis Ruzsky, 1915
montivaga Santschi, 1928
montaniformis Kuznetsov-Ugamsky, 1929
fuscoides Dlussky, 1967

##### Distribution

England, Wales

#### Formica exsecta

Nylander, 1846

exsectopressilabris Forel, 1874
rubens Forel, 1874
etrusca Emery, 1909
dalcqi Bondroit, 1918
sudetica Scholz, 1924
wheeleri Creighton, 1935
kontuniemii Betrem, 1954
nemoralis Dlussky, 1964

##### Distribution

England, Scotland

#### Formica fusca

Linnaeus, 1758

libera Scopoli, 1763
flavipes Geoffroy, 1785
barbata Razoumowski, 1789
tristis Christ, 1791
glebaria Nylander, 1846
marcida Wheeler, 1913
pallipes Kuznetsov-Ugamsky, 1926
rufipes Stitz, 1930

##### Distribution

England, Scotland, Wales, Ireland, Isle of Man

#### Formica lemani

Bondroit, 1917

borealis Vashkevich, 1924

##### Distribution

England, Scotland, Wales, Ireland, Isle of Man

#### Formica lugubris

Zetterstedt, 1838

congerens Nylander, 1846
santschii Wheeler, 1913
nylanderi Bondroit, 1920
unicolor Ruzsky, 1926
montana Sadil, 1953

##### Distribution

England, Scotland, Wales, Ireland

#### Formica picea

Nylander, 1846

candida misident.
glabra White, 1884 preocc.
transkaucasica Nasonov, 1889
orientalis Ruzsky, 1915
piceoinplana Emery, 1925
inplana Emery, 1925
lochmatteri Stärcke, 1935

##### Distribution

England, Wales

##### Notes

Not a homonym under article 23.9.5 of the Code (Seifert 2004).

#### Formica pratensis

Retzius, 1783

nigricans Bondroit, 1912
cordieri Bondroit, 1917
grouvellei Bondroit, 1918
ciliata Ruzsky, 1926
thyssei Stärcke, 1942
pratensoides Gösswald, 1951

##### Distribution

England

##### Notes

Probably extinct in Britain (Edwards and Broad 2005).

#### Formica rufa

Linnaeus, 1761

ferruginea Christ, 1791
dorsata Panzer, 1798
major Nylander, 1849
piniphila Schenck, 1852
apicalis Smith, 1858
rufopratensis Forel, 1874
meridionalis Nasonov, 1889
gaullei Bondroit, 1917

##### Distribution

England, Wales

#### Formica rufibarbis

Fabricius, 1793

nicaeensis Leach, 1825
stenoptera Förster, 1850
cinereorufibarbis Forel, 1874
defensor Smith, 1878
fraterna Smith, 1878
piligera Lomnicki, 1925

##### Distribution

England

#### Formica sanguinea

Latreille, 1798

dominula Nylander, 1846
fusciceps Emery, 1895
mollesonae Ruzsky, 1903
clarior Ruzsky, 1905
flavorubra Forel, 1909
borea Santschi, 1925
strennua Santschi, 1925
griseopubescens Kuznetsov-Ugamsky, 1926
monticola Kuznetsov-Ugamsky, 1926
rotundata Kuznetzsov-Ugamsky, 1926
arenicola Kuznetsov-Ugamsky, 1928
leninei Santschi, 1928
tristis Karavaiev, 1929

##### Distribution

England, Scotland, Wales

#### Lasius

Fabricius, 1804

CHTHONOLASIUS Ruzsky, 1912
DENDROLASIUS Ruzsky, 1912
CAUTOLASIUS Wilson, 1955
AUSTROLASIUS Faber, 1967

#### Lasius alienus

(Foerster, 1850)

Formica aliena Foerster, 1850
americanus Emery, 1893
pannonica Röszler, 1942

##### Distribution

England, Scotland, Wales, Ireland

#### Lasius brunneus

(Latreille, 1798)

Formica brunnea Latreille, 1798
pallida (Latreille, 1798, *Formica*)
timida (Förster, 1850, *Formica*)
alienobrunneus Forel, 1874
nigrobrunneus (Donisthorpe, 1926, *Acanthomyops*)

##### Distribution

England, Wales

#### Lasius emarginatus

(Olivier, 1792)

Formica emarginata Olivier, 1792
brunneoemarginatus Forel, 1874
brunneoides Forel, 1874
nigroemarginatus Forel, 1874
illyricus Zimmermann, 1935
pontica Stärcke, 1944

##### Distribution

England

##### Notes

added by Pontin (2008); further records are given by Smith and Williams (2008)

#### Lasius flavus

(Fabricius, 1781)

Formica flava Fabricius, 1781
ruficornis (Fabricius, 1804, *Formica*)
brevicornis Emery, 1893
fuscoides Ruzsky, 1902
odoratus Ruzsky, 1905
claripennis Wheeler, 1917
microps Wheeler, 1917
morbosa (Bondroit, 1918, *Formicina*)
ibericus Santschi, 1925
apennina Menozzi, 1925
olivacea Karavaiev, 1926
helvus Cook, 1953

##### Distribution

England, Scotland, Wales, Ireland, Isle of Man

#### Lasius fuliginosus

(Latreille, 1798)

Formica fuliginosa Latreille, 1798

##### Distribution

England, Scotland, Wales, Ireland, Isle of Man

#### Lasius meridionalis

(Bondroit, 1920)

Formicina meridionalis Bondroit, 1920

##### Distribution

England, Wales

#### Lasius mixtus

(Nylander, 1846)

Formica mixta Nylander, 1846

##### Distribution

England, Scotland, Wales, Ireland

#### # Lasius neglectus

Van Loon, Boomsma & Andrasfalvy, 1990

##### Distribution

England

##### Notes

added by Fox (2009)

#### Lasius niger

(Linnaeus, 1758)

Formica nigra Linnaeus, 1758
nigerrima (Christ, 1791, *Formica*)
pallescens (Schenck, 1852, *Formica*)
alienoniger Forel, 1874
emeryi Ruzsky, 1905
nitidus (Kuznetzsov-Ugamsky, 1927, *Acanthomyops*)
minimus (Kuznetsov-Ugamsky, 1928, *Acanthomyops*)
transylvanica Röszler, 1943

##### Distribution

England, Scotland, Wales, Ireland, Isle of Man

#### Lasius platythorax

Seifert, 1991

##### Distribution

England, Scotland, Wales, Ireland

##### Notes

added by Seifert (1992)

#### Lasius psammophilus

Seifert, 1992

##### Distribution

England, Wales

##### Notes

added by Seifert (1992)

#### Lasius sabularum

(Bondroit, 1918)

Formicina sabularum Bondroit, 1918

##### Distribution

England, Wales, Isle of Man

#### Lasius umbratus

(Nylander, 1846)

Formica umbrata Nylander, 1846
aphidicola (Walsh, 1863, *Formica*)
exacutus Ruzsky, 1902
affinoumbratus Donisthorpe, 1914
belgarum (Bondroit, 1918, *Formicina*)
silvestrii Wheeler, 1928
hirtiscapus Stärcke, 1937
osakana Santschi, 1941
nyaradi (Röszler, 1943, *Chthonolasius*)
epinotalis Buren, 1944

##### Distribution

England, Scotland, Wales, Ireland, Isle of Man

#### Paratrechina

Motschulsky, 1863

#### # Paratrechina longicornis

(Latreille, 1802)

Formica longicornis Latreille, 1802
vagans (Jerdon, 1851, *Formica*)
gracilescens (Nylander, 1856, *Formica*)
currens Motschoulsky, 1863

#### Plagiolepis

Mayr, 1861

APOROMYRMEX Faber, 1969
PARAPLAGIOLEPIS Faber, 1969

#### # Plagiolepis schmitzii

Forel, 1895

barbara Santschi, 1911
crosi Santschi, 1920
madeirensis Emery, 1921

##### Notes

Two nests persisted on the Isle of Wight from 2007-2008 (BB, pers. obs., specimens in NHM, BB coll. and B. Seifert coll.) but appear to have died out since. A native of the Azores and Madeira.

#### Myrmicinae

Lepeletier, 1835

##### Notes

The tribal classification follows Ward et al. (2015).

#### Attini

Smith, 1858

#### Pheidole

Westwood, 1841

#### # Pheidole megacephala

(Fabricius, 1793)

Formica megacephala Fabricius, 1793
edax (Forskål, 1775, *Formica*)
trinodis (Losana, 1834, *Myrmica*)
pusilla (Heer, 1852, *Oecophthora*)
laevigata (Smith, 1855, *Myrmica*)
agilis (Smith, 1857, *Myrmica*)
testacea (Smith, 1858, *Atta*)
janus Smith, 1858
suspiciosa (Smith, 1859, *Myrmica*)
laevigata Mayr, 1862
perniciosa (Gerstäcker, 1862, *Oecophthora*)
picata Forel, 1891
scabrior Forel, 1891
gietleni Forel, 1905
bernhardae Emery, 1915

##### Notes

A tramp species, in the past regarded as a proper introduction in Britain but no established colonies have been reported in the period 1970–present. On a world-wide scale its importance as a tramp species seems to be diminishing. The origin of *megacephala* is certainly Afrotropical as it is a member of a large and successful species group that is otherwise confined to that region.

#### Crematogastrini

Forel, 1893

#### # Cardiocondyla

Emery, 1869

##### Notes

*Cardiocondyla britteni* Crawley, 1920 was described from a worker casually introduced in Britain; included here only because the type-locality is Britain.

#### # Crematogaster

Lund, 1831

##### Notes

*Crematogaster scutellaris* (Olivier, 1792, *Formica*) is an occasional accidental import with cork from southern Europe. It has never established in Britain and, with the decrease in the cork trade, imports of this species will probably become even rarer.

#### Formicoxenus

Mayr, 1855

SYMMYRMICA Wheeler, 1904

#### Formicoxenus nitidulus

(Nylander, 1846)

Myrmica nitidula Nylander, 1846
laeviuscula (Förster, 1850, *Myrmica*)
picea (Wasmann, 1906, *Leptothorax*)

##### Distribution

England, Scotland

#### Leptothorax

Mayr, 1855

DORONOMYRMEX Kutter, 1945
MYCHOTHORAX Ruzsky, 1904

##### Notes

Many species previously placed in *Leptothorax* have been transferred to *Temnothorax* by Bolton (2003).

#### Leptothorax acervorum

(Fabricius, 1793)

Formica acervorum Fabricius, 1793
lacteipennis (Zetterstedt, 1838, *Myrmica*)
nigrescens Ruzsky, 1905
superus Ruzsky, 1905
kamtschaticum Ruzsky, 1920
orientalis (Kuznetsov-Ugamsky, 1928, *Mychothorax*)

##### Distribution

England, Scotland, Wales, Ireland, Isle of Man

#### Myrmecina

Curtis, 1829

ARCHAEOMYRMEX Mann, 1921

#### Myrmecina graminicola

(Latreille, 1802)

Formica graminicola Latreille, 1802
latreillei Curtis, 1829
striatula (Nylander, 1849, *Myrmica*)
bidens (Förster, 1850, *Myrmica*)
kutteri Forel, 1914
grouvellei Bondroit, 1918
gotlandica Karavaiev, 1930
oelandica Karavaiev, 1930
dentata Santschi, 1939

##### Distribution

England, Wales

#### Strongylognathus

Mayr, 1853

MYRMUS Schenck, 1853

#### Strongylognathus testaceus

(Schenck, 1852)

Eciton testaceum Schenck, 1852
emarginatus (Schenck, 1852, *Myrmus*)
diveri Donisthorpe, 1936

##### Distribution

England

#### Temnothorax

Mayr, 1861

MACROMISCHA Roger, 1863
DICHOTHORAX Emery, 1895
MYRMOXENUS Ruzsky, 1902
PROTOMOGNATHUS Wheeler, 1905
ANTILLAEMYRMEX Mann, 1920
CROESOMYRMEX Mann, 1920
CHALEPOXENUS Menozzi, 1923
MYRMAMMOPHILUS Menozzi, 1925
MYRAFANT Smith, 1950
ICOTHORAX Hamann & Klemm, 1967

#### Temnothorax albipennis

(Curtis, 1854)

Stenamma albipennis Curtis, 1854
tuberum misident.; Orledge (1998)
tuberointerruptus (Bondroit, 1918, *Leptothorax*)

##### Distribution

England, Wales

#### Temnothorax interruptus

(Schenck, 1852)

Myrmica interrupta Schenck, 1852
simpliciuscula (Nylander, 1856, *Myrmica*)
tuberoaffinis (Bondroit, 1918, *Leptothorax*)

##### Distribution

England

#### Temnothorax nylanderi

(Foerster, 1850)

Myrmica nylanderi Foerster, 1850
cingulata (Schenck, 1852, *Myrmica*)
nylanderocorticalis (Forel, 1874, *Leptothorax*)
nylanderotuberum (Ruzsky, 1902, *Leptothorax*)

##### Distribution

England, Wales

#### # Temnothorax unifasciatus

(Latreille, 1798)

Formica unifasciata Latreille, 1798
anoplogynus (Emery, 1869, *Leptothorax*)
unifasciatointerruptus (Forel, 1874, *Leptothorax*)
kirillovi (Ruzsky, 1905, *Leptothorax*)
brauneri (Karavaiev, 1937, *Leptothorax*)
salina (Karavaiev, 1937, *Leptothorax*)
ucrainicus (Arnol’di, 1977, *Leptothorax*)

##### Distribution

England

##### Notes

Recorded from England as an accidental introduction by Fox (2011).

#### Tetramorium

Mayr, 1855

TETROGMUS Roger, 1857
ANERGATES Forel, 1874
XIPHOMYRMEX Forel, 1887
TRIGLYPHOTHRIX Forel, 1890
RHOPTROMYRMEX Mayr, 1901
ATOPULA Emery, 1912
MACROMISCHOIDES Wheeler, 1920
LOBOMYRMEX Kratochvil, 1941
TELEUTOMYRMEX Kutter, 1950

#### Tetramorium atratulum

(Schenck, 1852)

Myrmica atratula Schenck, 1852
friedlandi (Creighton, 1934, *Anergates*)

##### Distribution

England

#### # Tetramorium bicarinatum

(Nylander, 1846)

Myrmica bicarinata Nylander, 1846
guineense misident.
cariniceps (Guérin-Méneville, 1852, *Myrmica*)
kollari (Mayr, 1853, *Myrmica*)
modesta (Smith, 1860, *Myrmica*)
reticulata (Smith, 1862, *Myrmica*)

#### Tetramorium caespitum

(Linnaeus, 1758)

Formica caespitum Linnaeus, 1758
fusca (Leach, 1825, *Formica*)
fuscula (Nylander, 1846, *Myrmica*)
modesta (Foerster, 1850, *Myrmica*)
himalayanum Viehmeyer, 1914
hammi Donisthorpe, 1915
immigrans Santschi, 1927
indocile Santschi, 1927
transbaicalense Ruzsky, 1936
transversinodis (Enzmann, 1946, *Myrmica*)
fusciclavum Consani & Zngheri, 1952
jiangxiense Wang & Xiao, 1988

##### Distribution

England, Scotland, Wales, Ireland, Isle of Man

##### Notes

*Tetramorium caespitum* s.l. is now recognised as comprising several species but the species present in Britain is apparently the true *caespitum*, at least on the basis of specimens from southern England (Schlick-Steiner et al. 2006).

#### # Tetramorium simillimum

(Smith, 1851)

Myrmica simillima Smith, 1851
parallela (Smith, 1859, *Myrmica*)
pygmaeum Emery, 1877
denticulatum Forel, 1902
bantouana Santschi, 1910
opacior Forel, 1913
exoleta Santschi, 1914
brevispinosa (Borgmeier, 1928, *Wasmannia*)
insulare Santschi, 1928

#### Myrmicini

Lepeletier, 1835

#### Myrmica

Latreille, 1804

SIFOLINIA Emery, 1907
SOMMIMYRMA Menozzi, 1925
SYMBIOMYRMA Arnol’di, 1930
PARAMYRMICA Cole, 1957
DODECAMYRMICA Arnol’di, 1968

#### Myrmica hirsuta

Elmes, 1978

##### Distribution

England, Wales

#### Myrmica karavajevi

(Arnol’di, 1930)

Symbiomyrma karavajevi Arnol’di, 1930
laurae misident.
pechei (Samsinak, 1957, *Sifolinia*)
faniensis van Boven, 1970
winterae (Kutter, 1973, *Sifolinia*)

##### Distribution

England, Wales

#### Myrmica lobicornis

Nylander, 1846

denticornis Curtis, 1854
arduennae Bondroit, 1911
angustifrons Stärcke, 1927
brunescens Karavaiev, 1929
burtshakabramovitshi Karavaiev, 1929
starki Karavaiev, 1929
foreli Santschi, 1931
alpestris Arnol’di, 1934
kievensis Karavaiev, 1934
lissahorensis Stitz, 1939

##### Distribution

England, Scotland, Wales

#### Myrmica lonae

Finzi, 1926

##### Distribution

Scotland

##### Notes

added by Seifert (2000)

#### Myrmica rubra

(Linnaeus, 1758)

Formica rubra Linnaeus, 1758
laevinodis Nylander, 1846
longiscapus Curtis, 1854
champlaini Forel, 1901
europaea Finzi, 1926
bruesi Weber, 1947
microrubra Seifert, 1993

##### Distribution

England

#### Myrmica ruginodis

Nylander, 1846

dimidiata Say, 1836
diluta Nylander, 1849
ruginodolaevinodis Forel, 1874
silvestrii Wheeler, 1928
sontica Santschi, 1937
yoshiokai Weber, 1947
macrogyna Brian & Brian, 1949
microgyna Brian & Brian, 1949
mutata Sadil, 1952

##### Distribution

England, Scotland, Wales, Ireland, Isle of Man

#### Myrmica sabuleti

Meinert, 1861

scabrinodolobicornis Forel, 1874

##### Distribution

England, Scotland, Wales, Ireland, Isle of Man

#### Myrmica scabrinodis

Nylander, 1846

rugulosoides Forel, 1915
pilosiscapus Bondroit, 1920
ahngeri Karavaiev, 1926
scabrinodosabuleti Sadil, 1952

##### Distribution

England, Scotland, Wales, Ireland, Isle of Man

#### Myrmica schencki

Viereck, 1903

kutteri Finzi, 1926
subopaca Arnol'di, 1934
betuliana Ruzsky, 1946
schenckioides Boer & Noordijk, 2005

##### Distribution

England, Wales, Ireland

#### Myrmica specioides

Bondroit, 1918

silvestrianum Emery, 1924
striata Finzi, 1926
sancta Karavaiev, 1926
nevodovskii (Karavaiev, 1926, *Leptothorax*)
turcica Santschi, 1931
puerilis Stärcke, 1942
dolens Stärcke, 1942
balcanica Sadil, 1952
scabrinodoides Sadil, 1952
tschuliensis Arnol’di, 1976
kozakorum Radchenko & Elmes, 2010

##### Distribution

England

#### Myrmica sulcinodis

Nylander, 1846

perelegans Curtis, 1854
nigripes Ruzsky, 1895
myrmecophila Wasmann, 1910
sulcinodoruginodis Donisthorpe, 1915
sulcinodoscabrinodis Forel, 1915
derzhavini Ruzsky, 1920
vicaria Kuznetsov-Ugamsky, 1928
eximia Kupyanskaya, 1990

##### Distribution

England, Scotland, Wales

#### Myrmica vandeli

Bondroit, 1920

##### Distribution

England, Wales

##### Notes

added by Elmes et al. (2003)

#### Solenopsidini

Forel, 1893

#### Monomorium

Mayr, 1855

#### # Monomorium floricola

(Jerdon, 1851)

Atta floricola Jerdon, 1851
# Monomorium floricola *cinnabari* Roger, 1863
# Monomorium floricola *poecilum* Roger, 1863
# Monomorium floricola *specularis* Mayr, 1866
# Monomorium floricola *impressum* Smith, 1876
# Monomorium floricola *philippinensis* Forel, 1910
# Monomorium floricola *furina* Forel, 1911
# Monomorium floricola *floreanum* Stitz, 1932
# Monomorium floricola *angusticlava* Donisthorpe, 1947

#### # Monomorium pharaonis

(Linnaeus, 1758)

Formica pharaonis Linnaeus, 1758
antiguensis (Fabricius, 1793, *Formica*)
domestica (Shuckard, 1838, *Myrmica*)
minuta (Jerdon, 1851, *Atta*)
vastator (Smith, 1857, *Myrmica*)
contigua (Smith, 1858, *Myrmica*)
fragilis (Smith, 1858, *Myrmica*)

#### Solenopsis

Westwood, 1840

DIPLORHOPTRUM Mayr, 1855
OCTELLA Forel, 1915
SYNSOLENOPSIS Forel, 1918
DIAGYNE Santschi, 1923
EUOPHTHALMA Creighton, 1930
LABAUCHENA Santschi, 1930
OEDALEOCERUS Creighton, 1930
BISOLENOPSIS Kusnezov, 1953
PARANAMYRMA Kusnezov, 1953
GRANISOLENOPSIS Kusnezov, 1957
LILIDRIS Kusnezov, 1957

#### Solenopsis fugax

(Latreille, 1798)

Formica fugax Latreille, 1798
flavidula (Nylander, 1849, *Myrmica*)
latroides Ruzsky, 1905
orientalis Ruzsky, 1905
kasalinensis Emery, 1909
pontica Santschi, 1934
scythica Santschi, 1934
furtiva Santschi, 1934
balachowskyi Bernard, 1950
banyulensis Bernard, 1950
duboscqui Bernard, 1950
laevithorax Bernard, 1950
monticola Bernard, 1950
nicaeensis Bernard, 1950
provincialis Bernard, 1950
pygmaea Bernard, 1950
richardi Bernard, 1950
robusta Bernard, 1950
rugosa Bernard, 1950
tertialis Ettershank, 1966
avium (Bernard, 1978, *Diplorhoptrum*)
delta (Bernard, 1978, *Diplorhoptrum*)
insulare (Bernard, 1978, *Diplorhoptrum*)
pilosum (Bernard, 1978, *Diplorhoptrum*)

##### Distribution

England

#### Stenammini

Ashmead, 1905

#### Stenamma

Westwood, 1839

ASEMORHOPTRUM Mayr, 1861
THERYELLA Santschi, 1921

#### Stenamma debile

(Förster, 1850)

Myrmica debilis Förster, 1850
minkii (Förster, 1850, *Myrmica*)
golosejevi Karavaiev, 1926
ucrainicum Arnol’di, 1928
polonicum Begdon, 1932
orousseti Casevitz-Weulerrse, 1990

##### Distribution

England, Wales, Ireland

##### Notes

added by DuBois (1993)

#### Stenamma westwoodii

Westwood, 1839

##### Distribution

England

##### Notes

Records from Ireland may relate to *debile* (Fox in Collins and Roy 2012).

#### Ponerinae

Lepeletier, 1835

#### Hypoponera

Santschi, 1938

##### Notes

*Hypoponera gibbinota* (Forel, 1912, *Ponera*) was described from a worker casually introduced to Britain.

#### # Hypoponera punctatissima

(Roger, 1859)

Ponera punctatissima Roger, 1859
androgyna (Roger, 1859, *Ponera*)
tarda (Charsley, 1877, *Ponera*)
jugata (Forel, 1892, *Ponera*)
brevis (Santschi, 1911, *Ponera*)
cognata (Santschi, 1912, *Ponera*)
durbanensis (Forel, 1914, *Ponera*)
incisa (Santschi, 1914, *Ponera*)
sordida (Santschi, 1914, *Ponera*)
petri (Forel, 1916, *Ponera*)
exacta (Santschi, 1923, *Ponera*)
mina (Wheeler, 1927, *Ponera*)
mumfordi (Wheeler, 1931, *Ponera*)
argonautorum (Arnol’di, 1932, *Ponera*)
mesoepinotalis (Weber, 1942, *Ponera*)
breviceps (Bernard, 1953, *Ponera*)
ursoidea (Bernard, 1953, *Ponera*)
sulcitana (Stefani, 1970, *Ponera*)

##### Distribution

England, Scotland, Wales, Ireland

#### # Hypoponera ergatandria

(Forel, 1893)

Ponera ergatandria Forel, 1893
schauinslandi (Emery, 1899, *Ponera*) synonymy by Seifert (2013)
kalakauae (Forel, 1899, *Ponera*)
aemula (Santschi, 1911, *Ponera*)
bondroiti (Forel, 1911, *Ponera*)

##### Distribution

England

#### Ponera

Latreille, 1804

PSEUDOCRYPTOPONE Wheeler, 1933
SELENOPONE Wheeler, 1933
PTEROPONERA Bernard, 1950

#### Ponera coarctata

(Latreille, 1802)

Formica coarctata Latreille, 1802
contracta (Latreille, 1802, *Formica*)
lucida Emery, 1898
atlantis Santschi, 1921

##### Distribution

England, Wales

#### Ponera testacea

Emery, 1895

crassisquama Emery, 1916

##### Distribution

England

##### Notes

added by Attewell et al. (2010)

#### Mutillidae

Latreille, 1802

##### Notes

Nomenclature follows Lelej (2002), who includes full synonymy. Only names that have been used in the British literature are included here.

#### Mutillinae

Latreille, 1802

#### Mutilla

Linnaeus, 1758

#### Mutilla europaea

Linnaeus, 1758

##### Distribution

England, Scotland

#### Smicromyrme

Thomson, 1870

#### Smicromyrme rufipes

(Fabricius, 1787)

Mutilla rufipes Fabricius, 1787

##### Distribution

England

#### Myrmosinae

Fox, 1894

#### Myrmosa

Latreille, 1796

#### Myrmosa atra

Panzer, 1801

melanocephala (Fabricius, 1793, *Mutilla*) preocc.

##### Distribution

England, Wales, Ireland, Isle of Man

##### Notes

The nominate subspecies occurs in England and Wales, *atra erythrocephala* Yarrow, 1954 in Ireland and the Isle of Man.

#### Pompilidae

Latreille, 1805

PSAMMOCHARIDAE Banks, 1910

##### Notes

Classification follows Wahis (1986), Wahis (2006). Some distribution data from Day (1988).

#### Pepsinae

Lepeletier, 1845

#### Pepsini

Lepeletier, 1845

#### Auplopus

Spinola, 1841

PILPOMUS Costa, 1859
PSEUDAGENIA Kohl, 1844

#### Auplopus carbonarius

(Scopoli, 1763)

Sphex carbonaria Scopoli, 1763
punctum (Fabricius, 1804, *Ceropales*)
canaliculatus (Schenck, 1857, *Agenia*)
submarginatus Lepeletier, 1845
albigena Lepeletier, 1845
collinus Haupt, 1962
silvalis Haupt, 1962

##### Distribution

England, Wales

#### Caliadurgus

Pate, 1946

CALICURGUS Lepeletier, 1845

#### Caliadurgus fasciatellus

(Spinola, 1808)

Pompilus fasciatellus Spinola, 1808
calcaratus (Dahlbom, 1829, *Pompilus*)
maculipennis (Dahlbom, 1829, *Pompilus*)
albispinus (Herrich-Schäffer, 1830, *Pompilus*)
curtus (Zetterstedt, 1838, *Pompilus*)
gyllenhali (Dahlbom, 1843, *Priocnemis*)
labiatus (Lepeletier, 1845, *Anoplius*)
unimacula (Lepeletier, 1845, *Anoplius*)
odontellus (Lepeletier, 1845, *Calicurgus*)
bivirgulatus (Costa, 1881, *Pompilus*)
fuscopennis (Verhoeff, 1892, *Priocnemis*)

##### Distribution

England, Wales

#### Cryptocheilus

Panzer, 1806

CALICURGUS Brullé, 1833
CHLOROCHEILUS Wolf, 1965

#### Adonta

Bilberg, 1820

SALIUS Fabricius, 1804
HOMONOTUS Dahlbom, 1845

#### Cryptocheilus (Adonta) notatus

(Rossi, 1792)

Sphex notata Rossi, 1792
guttus (Spinola, 1808, *Pompilus*)
affinis Vander Linden, 1827, *Pompilus*)
iracundus (Dufour, 1841, *Pompilus*)
apricus (Lepeletier, 1845, *Calicurgus*)
melanius (Lepeletier, 1845, *Calicurgus*)
binotatus (Marquet, 1879, *Priocnemis*) preocc.
marquetii (Dalla Torre, 1897, *Salius*)
orientalis Haupt, 1927

##### Distribution

England, Wales

#### Dipogon

Fox, 1897

#### Deuteragenia

Sustera, 1912

#### Dipogon (Deuteragenia) bifasciatus

(Geoffroy, 1785)

Ichneumon bifasciatus Geoffroy, 1785
hircanus (Fabricius, 1798, *Pompilus*)
intermedius (Dahlbom, 1843, *Agenia*)

##### Distribution

England

#### Dipogon (Deuteragenia) subintermedius

(Magretti, 1886)

Pogonius subintermedius Magretti, 1886
nitidus (Haupt, 1927, *Deuteragenia*)

##### Distribution

England, Scotland, Wales

#### Dipogon (Deuteragenia) variegatus

(Linnaeus, 1758)

Sphex variegata Linnaeus, 1758
erythropus (Kohl, 1888, *Agenia*)
structor (Ferton, 1897, *Agenia*)
faggiolii (Haupt, 1927, *Deuteragenia*)

##### Distribution

England, Scotland, Wales, Ireland

#### Priocnemis

Schiødte, 1837

#### Priocnemis

Schiødte, 1837

MACULIPENNIS Junco, 1946

#### Priocnemis (Priocnemis) agilis

(Shuckard, 1837)

Pompilus agilis Shuckard, 1837
obtusiventris Schiødte, 1837
fraterculus Junco, 1946

##### Distribution

England, Scotland, Wales

#### Priocnemis (Priocnemis) confusor

Wahis, 2006

gracilis Haupt, 1927 preocc.
gussakowskiji Wolf, 2004 preocc.

##### Distribution

England, Ireland

#### Priocnemis (Priocnemis) cordivalvata

Haupt, 1927

##### Distribution

England

#### Priocnemis (Priocnemis) exaltata

(Fabricius, 1775)

Sphex exaltata Fabricius, 1775
gibba (Scopoli, 1763, *Sphex*)
revo (Harris, 1780, *Sphex*)
nudipes Dahlbom, 1845
longicornis Haupt, 1927
valkeilai Wolf, 1959

##### Distribution

England, Scotland, Wales, Ireland, Isle of Man

#### Priocnemis (Priocnemis) fennica

Haupt, 1927

##### Distribution

England, Wales, Ireland

#### Priocnemis (Priocnemis) hyalinata

(Fabricius, 1793)

Sphex hyalinata Fabricius, 1793
femoralis (Dahlbom, 1829, *Pompilus*)
discrepans (Costa, 1887, *Pseudagenia*)
trifurcus Radoszkowski, 1888
vitripennis Verhoeff, 1892
notatulus (Saunders, 1896, *Salius*)
pseudofemoralis Sustera, 1938
taigaica Wolf, 1967

##### Distribution

England, Wales

#### Priocnemis (Priocnemis) parvula

Dahlbom, 1845

minor (Zetterstedt, 1879, *Pompilus*)
mocsaryi Gussakovskij, 1930
klosei Haupt, 1937
haupti Sustera, 1938
vinetorum Blüthgen, 1944
minutalis Wahis, 1979 nom. auct.

##### Distribution

England, Scotland, Wales

#### Priocnemis (Priocnemis) propinqua

(Lepeletier, 1845)

Calicurgus propinquus Lepeletier, 1845
agenoides Dubois, 1920

##### Distribution

England

#### Priocnemis (Priocnemis) pusilla

(Schiødte, 1837)

Pompilus pusillus Schiødte, 1837

##### Distribution

England, Wales

#### Priocnemis (Priocnemis) schioedtei

Haupt, 1927

##### Distribution

England, Scotland, Wales

#### Umbripennis

Junco, 1946

PRIOCNEMISSUS Haupt, 1949

#### Priocnemis (Umbripennis) coriacea

Dahlbom, 1843

capciosus Junco, 1946

##### Distribution

England

#### Priocnemis (Umbripennis) perturbator

(Harris, 1780)

Sphex perturbator Harris, 1780
fusca misident.
ambustor (Panzer, 1804, *Ichneumon*)
serripes (Dahlbom, 1829, *Pompilus*)
ambulator (Lepeletier, 1845, *Calicurgus*)
sepicola (Smith, 1851, *Pompilus*)
ater Wolf, 1960

##### Distribution

England, Scotland, Wales, Ireland

#### Priocnemis (Umbripennis) susterai

Haupt, 1927

clementi Haupt, 1927
gasconia Wolf, 1975

##### Distribution

England, Wales

#### Pompilinae

Latreille, 1805

#### Agenioideus

Ashmead, 1902

APOROIDEUS Ashmead, 1902
GYMNOCHARES Banks, 1917

#### Agenioideus cinctellus

(Spinola, 1808)

Pompilus cinctellus Spinola, 1808
clypeatus (Dahlbom, 1829, *Pompilus*)
punctipes (Dahlbom, 1832, *Pompilus*)
tibialis (Lepeletier, 1845, *Anoplius*)

##### Distribution

England, Wales

#### Agenioideus sericeus

(Vander Linden, 1827)

Pompilus sericeus Vander Linden, 1827
vicinus (Lepeletier, 1845, *Pompilus*)
subserricornis (Kohl, 1879, *Pompilus*)
declivus (Tournier, 1889, *Pompilus*)
gaullei (Tournier, 1889, *Pompilus*)
hungaricus (Moczar, 1944, *Anospilus*)

##### Distribution

England

##### Notes

added by Baldock (2006)

#### Anoplius

Dufour, 1834

#### Anoplius

Dufour, 1834

#### Anoplius (Anoplius) caviventris

(Aurivillius, 1907)

Pompilus caviventris Aurivillius, 1907
cardui (Perkins, 1917, *Pompilus*)
carbonarius Haupt, 1937
atricolor Moczar, 1944

##### Distribution

England, Wales

#### Anoplius (Anoplius) concinnus

(Dahlbom, 1845)

Pompilus concinnus Dahlbom, 1845
vacillans (Wesmael, 1851, *Pompilus*)
approximatus (Smith, 1877, *Pompilus*)
bifidus (Morawitz, 1891, *Pompilus*)
distinguendus (Morawitz, 1891, *Pompilus*)
haereticus (Tournier, 1889, *Pompilus*)

##### Distribution

England, Scotland, Wales, Ireland

#### Anoplius (Anoplius) nigerrimus

(Scopoli, 1763)

Sphex nigerrima Scopoli, 1763
nigrus (Fabricius, 1775, *Sphex*)
incisus (Tischbein, 1850, *Pompilus*)
melanarius (Schenck, 1857, *Pompilus*) preocc.
excerptus (Tournier, 1889, *Pompilus*)
difficilis (Tournier, 1889, *Pompilus*)
wheeleri Banks, 1939
banksi Dreisbach, 1950

##### Distribution

England, Scotland, Wales, Ireland, Isle of Man

#### Arachnophroctonus

Howard, 1901

POMPILINUS Ashmead, 1902

#### Anoplius (Arachnophroctonus) infuscatus

(Vander Linden, 1827)

Pompilus infuscatus Vander Linden, 1827
minor (Herrich-Schäffer, 1830, *Pompilus*)
sericatus (Shuckard, 1835, *Pompilus*)
Anoplius (Arachnophroctonus) infuscatus *chalybeatus* (Schiødte, 1837, *Pompilus*)
Anoplius (Arachnophroctonus) infuscatus *difformis* (Schiødte, 1837, *Pompilus*)
dispar (Dahlbom, 1843, *Pompilus*)
Anoplius (Arachnophroctonus) infuscatus *sabulicola* (Thomson, 1874, *Pompilus*)
Anoplius (Arachnophroctonus) infuscatus *meticulosa* (Costa, 1882, *Pompilus*)
Anoplius (Arachnophroctonus) infuscatus *aeruginosus* (Tournier, 1890, *Pompilus*)
Anoplius (Arachnophroctonus) infuscatus *aerarius* (Tournier, 1890, *Pompilus*)
Anoplius (Arachnophroctonus) infuscatus *argentatus* (Tournier, 1890, *Pompilus*)
Anoplius (Arachnophroctonus) infuscatus *calcatus* (Tournier, 1890, *Pompilus*)
Anoplius (Arachnophroctonus) infuscatus *onus* (Tournier, 1890, *Pompilus*)
Anoplius (Arachnophroctonus) infuscatus *stellatus* (Tournier, 1890, *Pompilus*)
Anoplius (Arachnophroctonus) infuscatus *utendus* (Tournier, 1890, *Pompilus*)
Anoplius (Arachnophroctonus) infuscatus *vivus* (Tournier, 1890, *Pompilus*)
Anoplius (Arachnophroctonus) infuscatus *xysticus* (Tournier, 1890, *Pompilus*)
petulans Haupt, 1962
cinctellus (Haupt, 1962, *Paracyphonyx*)
lusitanicus Wolf & Diniz, 1970
Anoplius (Arachnophroctonus) infuscatus *fortunatus* Wolf, 1975
Anoplius (Arachnophroctonus) infuscatus *simii* Wolf, 1978

##### Distribution

England, Wales

#### Anoplius (Arachnophroctonus) viaticus

(Linnaeus, 1758)

Sphex viatica Linnaeus, 1758
fuscus (Linnaeus, 1761, *Sphex*)
paganus (Dahlbom, 1843, *Pompilus*)
propinquus (Smith, 1879, *Pompilus*)
tibialis (Tournier, 1890, *Pompilus*) preocc.
delatorius (Tournier, 1890, *Pompilus*)
immixtus (Tournier, 1890, *Pompilus*)
pleropicus (Tournier, 1890, *Pompilus*)
valesicus (Tournier, 1890, *Pompilus*)
macrurus (Dalla Torre, 1897, *Pompilus*)
holomelas (Mantero, 1905, *Pompilus*) preocc.

##### Distribution

England, Wales, Ireland

#### Aporus

Spinola, 1808

#### Aporus unicolor

Spinola, 1808

femoralis Vander Linden, 1827
castor (Kohl, 1838, *Pompilus*)

##### Distribution

England, Wales

#### Arachnospila

Kincaid, 1900

#### Ammosphex

Wilcke, 1942

ANOPOMPILINUS Dreisbach, 1949
ARIDOPOMPILUS Wolf, 1965
BOREOPOMPILUS Wolf, 1965
HOLARCTOPOMPILUS Wolf, 1965
SAXATILIPOMPILUS Wolf, 1965

#### Arachnospila (Ammosphex) anceps

(Wesmael, 1851)

Pompilus anceps Wesmael, 1851
vaga (Harris, 1870, *Sphex*) preocc.
unguicularis (Thomson, 1870, *Pompilus*)
crobaci (Tournier, 1890, *Pompilus*)
expleta (Tournier, 1890, *Pompilus*)
lustrica (Tournier, 1890, *Pompilus*)
nava (Tournier, 1890, *Pompilus*)
radiosa (Tournier, 1890, *Pompilus*)
saxea (Tournier, 1890, *Pompilus*)
peninsulana (Wolf, 1966, *Pompilus*)
serica Wolf & Moczar, 1972

##### Distribution

England, Scotland, Wales, Ireland, Isle of Man

#### Arachnospila (Ammosphex) consobrina

(Dahlbom, 1843)

Pompilus consobrinus Dahlbom, 1843
ater (Brullé, 1840, *Pompilus*) preocc.
excisa (Pérez, 1895, *Pompilus*) preocc.
nivariae (Dalla Torre, 1897, *Pompilus*)
guimarensis (Saunders, 1904, *Pompilus*)
lanuginosa (Haupt, 1927, *Psammochares*)
heringi (Haupt, 1928, *Psammochares*)
emissa (Haupt, 1930, *Psammochares*)
laufferi (Junco, 1960, *Pompilus*)
alpina (Wolf, 1965, *Pompilus*)
pyrenaica (Wolf, 1965, *Pompilus*)
continentalis (Wolf, 1966, *Pompilus*)
sicula (Wolf, 1966, *Pompilus*)

##### Distribution

England, Wales

#### Arachnospila (Ammosphex) trivialis

(Dahlbom, 1843)

Pompilus trivialis Dahlbom, 1843
gibba misident.
aerumnata (Tournier, 1889, *Pompilus*)
corruptor (Haupt, 1927, *Psammochares*)
michalki (Blüthgen, 1961, *Ammosphex*)
insubrica (Wolf, 1965, *Pompilus*)

##### Distribution

England, Wales

#### Arachnospila (Ammosphex) wesmaeli

(Thomson, 1870)

Pompilus wesmaeli Thomson, 1870

##### Distribution

England, Wales

#### Anoplochares

Banks, 1939

#### Arachnospila (Anoplochares) minutula

(Dahlbom, 1842)

Pompilus minutulus Dahlbom, 1842
cellularis (Dahlbom, 1843, *Pompilus*)
neglecta (Dahlbom, 1843, *Pompilus*)
inermis (Lepeletier, 1845, *Anoplius*)
anoplius (Dalla Torre, 1897, *Pompilus*)
simplicicra (Priesner, 1960, *Pompilus*)
apenninusurata Wolf, 1970

##### Distribution

England, Wales

#### Arachnospila (Anoplochares) spissa

(Schiødte, 1837)

Pompilus spissus Schiødte, 1837
apennina Wolf, 1970

##### Distribution

England, Scotland, Wales

#### Arachnospila

Kincaid, 1900

PYCNOPOMPILUS Ashmead, 1902

#### Arachnospila (Arachnospila) rufa

(Haupt, 1927)

Psammochares rufus Haupt, 1927
adulterina (Haupt, 1937, *Psammochares*)
melanota Wolf, 1975

##### Distribution

England

#### Episyron

Schiødte, 1837

SPILOPOMPILUS Ashmead, 1902

#### Episyron gallicum

(Tournier, 1889)

Pompilus gallicus Tournier, 1889
intermedius Haupt, 1930
tertius Blüthgen, 1944

##### Distribution

England

##### Notes

added by Baldock (2006)

#### Episyron rufipes

(Linnaeus, 1758)

Sphex rufipes Linnaeus, 1758
laevigata (Rossius, 1794, *Sphex*)
gracilis (Lepeletier, 1845, *Pompilus*) preocc.
septemmaculatus (Wesmael, 1851, *Pompilus*)
argyrolepis (Costa, 1887, *Pompilus*)
compressus (Tournier, 1889, *Pompilus*) preocc.
aequatus (Tournier, 1889, *Pompilus*)
pygidialis (Tournier, 1889, *Pompilus*)
deuterus (Dalla Torre, 1897, *Pompilus*)
ephialtes (Dalla Torre, 1897, *Pompilus*)
sardous Wolf, 1961

##### Distribution

England, Wales, Ireland

#### Evagetes

Lepeletier, 1845

SOPHROPOMPILUS Ashmead, 1902
ASTHENOCTENUS Arnold, 1937
ASTHENOCTENIDIA Pate, 1946
LEUCHIMON Haupt, 1930
TRICHOSYRON Haupt, 1930
PSAMMOCHAROIDES Moczar, 1946
STREPTOSELLA Dreisbach, 1950
CARINEVAGETES Wolf, 1970
CONTEMPTEVAGETES Wolf, 1970

#### Evagetes crassicornis

(Shuckard, 1835)

Pompilus crassicornis Shuckard, 1835
dahlbomi (Thomson, 1870, *Pompilus*)
subarcticus Wolf, 1964

##### Distribution

England, Scotland, Wales, Ireland, Isle of Man

#### Evagetes dubius

(Vander Linden, 1827)

Aporus dubius Vander Linden, 1827
bicolor Lepeletier, 1845
servillei Costa, 1882
rattus (Dalla Torre, 1897, *Pompilus*)
obscurodubius Wolf, 1970
theodori Wolf, 1970

##### Distribution

England

#### Evagetes pectinipes

(Linnaeus, 1758)

Sphex pectinipes Linnaeus, 1758
quadrispinosus (Kohl, 1886, *Pompilus*)
aculeatus (Thomson, 1870, *Pompilus*)
minotaurus Wolf, 1970

##### Distribution

England

#### Homonotus

Dahlbom, 1843

ISONOTUS Dahlbom, 1842
WESMAELINIUS Costa, 1886

#### Homonotus sanguinolentus

(Fabricius, 1793)

Sphex sanguinolenta Fabricius, 1793
dispar (Latreille, 1809, *Pompilus*)
bidens (Lepeletier, 1845, *Anoplius*)
affinis (Eversmann, 1849, *Pompilus*) preocc.
nigrus (Marquet, 1879, *Ferrola*)
nasutus Morawitz, 1888
doctor (Dalla Torre, 1897, *Pompilus*)

##### Distribution

England

#### Pompilus

Fabricius, 1798

CHIONOPOMPILUS Priesner, 1955

#### Pompilus cinereus

(Fabricius, 1775)

Sphex cinerea Fabricius, 1775
plumbeus (Fabricius, 1787, *Sphex*)
pulcher Fabricius, 1798
pruinosus Smith, 1879 preocc.
chevrieri Tournier, 1889
leprosus Dalla Torre, 1897
lusitanicus Wolf & Diniz, 1970
gotlandicus Wolf, 1972

##### Distribution

England, Scotland, Wales, Ireland, Isle of Man

#### Ceropalinae

Radoszkowski, 1888

#### Ceropales

Latreille, 1796

CERATOPALES Schulz, 1906
HYPSICERAEUS Morice & Durrant, 1915

#### Ceropales maculata

(Fabricius, 1775)

Evania maculata Fabricius, 1775
rustica (Müller, 1776, *Sphex*)
multicolor (Fourcroy, 1785, *Ichneumon*)
frontalis (Panzer, 1799, *Pompilus*)
perligerus (Costa, 1882, *Priocnemis*)
balearica Costa, 1893

##### Distribution

England, Scotland, Wales, Ireland, Isle of Man

#### Ceropales variegata

(Fabricius, 1798)

Evania variegata Fabricius, 1798
destefanii Costa, 1887

##### Distribution

England

#### Sapygidae

Latreille, 1810

#### Monosapyga

Pic, 1920

#### Monosapyga clavicornis

(Linnaeus, 1758)

Apis clavicornis Linnaeus, 1758

##### Distribution

England, Wales

#### Sapyga

Latreille, 1796

#### Sapyga quinquepunctata

(Fabricius, 1781)

Scolia quinquepunctata Fabricius, 1781

##### Distribution

England, Wales

#### Tiphiidae

Leach, 1915

#### Methochinae

#### Methocha

Latreille, 1804

METHOCA misspelling

##### Notes

Taxonomy follows Agnoli (2005).

#### Methocha articulata

Latreille, 1792

formicaria (Latreille, 1792, *Mutilla*) nom. dub.
ichneumonides Latreille, 1805
mutillaria Latreille, 1806
sanvitali (Latreille, 1809, *Tengyra*)
italica (Costa, 1858, *Spinolia*)

##### Distribution

England, Wales

#### Tiphiinae

#### Tiphia

Fabricius, 1775

#### Tiphia femorata

Fabricus, 1775

##### Distribution

England, Wales

#### Tiphia minuta

Vander Linden, 1827

##### Distribution

England, Scotland, Wales, Ireland, Isle of Man

#### Vespidae

Laicharting, 1781

#### Eumeninae

##### Notes

Taxonomy mostly follows van der Vecht and Fischer (1972) and Fauna Europaea (Mitroiu et al. 2004).

#### Ancistrocerus

Wesmael, 1836

#### Ancistrocerus antilope

(Panzer, 1798)

Vespa antilope Panzer, 1798
pictus (Curtis, 1826, *Odynerus*)

##### Distribution

England, Scotland, Wales

#### Ancistrocerus claripennis

Thomson, 1874

quadratus (Panzer, 1799, *Vespa*) nom. dub.

##### Distribution

England

##### Notes

Although this species has been referred to as *Ancistrocerus quadratus* in British faunistic works (e.g. Richards 1980, Archer 2003, Archer 2014), the type of *Vespa quadrata* Panzer is lost and its identification as this species, whilst probable, is not certain (Blüthgen 1959, van der Vecht and Fischer 1972), therefore *claripennis* is the name most usually used for this species (e.g. in Fauna Europaea: Mitroiu et al. 2004, accessed 2016).

#### Ancistrocerus gazella

(Panzer, 1798)

Vespa gazella Panzer, 1798
emarginata (Fabricius, 1793, *Vespa*) preocc.

##### Distribution

England, Scotland, Wales, Ireland

#### Ancistrocerus nigricornis

(Curtis, 1826)

Odynerus nigricornis Curtis, 1826
callosus (Thomson, 1870, *Odynerus*)
excisus (Thomson, 1870, *Odynerus*)
sexpunctata (Christ, 1791, *Vespa*)

##### Distribution

England, Scotland, Wales, Ireland, Isle of Man

#### Ancistrocerus oviventris

(Wesmael, 1836)

Odynerus oviventris Wesmael, 1836
constans (Herrich-Schäffer, 1839, *Odynerus*)
viduus (Herrich-Schäffer, 1839, *Odynerus*)

##### Distribution

England, Scotland, Wales, Ireland, Isle of Man

##### Notes

The subspecies *hibernicus* Blüthgen, 1937 occurs in Ireland and the Outer Hebrides.

#### Ancistrocerus parietinus

(Linnaeus, 1758)

Vespa parietina Linnaeus, 1758
domesticus (Christ, 1791, *Sphex*)
affinis (Herrich-Schäffer, 1839, *Odynerus*)

##### Distribution

England, Scotland, Wales, Ireland, Isle of Man

#### Ancistrocerus parietum

(Linnaeus, 1758)

Vespa parietum Linnaeus, 1758

##### Distribution

England, Scotland, Wales, Ireland, Isle of Man

#### Ancistrocerus scoticus

(Curtis, 1826)

Odynerus scoticus Curtis, 1826
trimarginatus misident.
albotricinctus (Zetterstedt, 1838, *Odynerus*)

##### Distribution

England, Scotland, Wales, Ireland, Isle of Man

#### Ancistrocerus trifasciatus

(Müller, 1776)

Vespa trifasciata Müller, 1776
trimarginatus (Zetterstedt, 1838, *Odynerus*)
tricinctus (Herrich-Schäffer, 1839, *Odynerus*)

##### Distribution

England, Scotland, Wales, Ireland, Isle of Man

#### Eumenes

Latreille, 1802

#### Eumenes coarctatus

(Linnaeus, 1758)

Vespa coarctata Linnaeus, 1758
papillarius (Christ, 1791, *Sphex*)

##### Distribution

England, Wales

#### Euodynerus

Dalla Torre, 1904

#### Pareuodynerus

Blüthgen, 1938

#### Euodynerus (Pareuodynerus) quadrifasciatus

(Fabricius, 1793)

Vespa quadrifasciata Fabricius, 1793
tomentosus (Thomson, 1870, *Odynerus*)

##### Distribution

England

#### Gymnomerus

Blüthgen, 1938

#### Gymnomerus laevipes

(Shuckard, 1837)

Odynerus laevipes Shuckard, 1837

##### Distribution

England, Wales

#### Microdynerus

Thomson, 1874

#### Microdynerus exilis

(Herrich-Schäffer, 1839)

Odynerus exilis Herrich-Schäffer, 1839
bivittatus (Lepeletier, 1841, *Odynerus*)

##### Distribution

England

#### Odynerus

Latreille, 1802

#### Odynerus

Latreille, 1802

#### Odynerus (Odynerus) melanocephalus

(Gmelin, 1790)

Vespa melanocephala Gmelin, 1790

##### Distribution

England, Wales

#### Odynerus (Odynerus) spinipes

(Linnaeus, 1758)

Vespa spinipes Linnaeus, 1758
quinquefasciata (Fabricius, 1793, *Vespa*) preocc.
muticus (Zetterstedt, 1838, *Odynerus*)

##### Distribution

England, Scotland, Wales, Ireland, Isle of Man

#### Spinicoxa

Blüthgen, 1938

#### Odynerus (Spinicoxa) reniformis

(Gmelin, 1790)

Vespa reniformis Gmelin, 1790

##### Distribution

England

#### Odynerus (Spinicoxa) simillimus

Morawitz, 1867

##### Distribution

England

#### Pseudepipona

Saussure, 1856

#### Pseudepipona herrichii

(Saussure, 1855)

Odynerus herrichii Saussure, 1855
variegata misident.
basalis (Smith, 1857, *Odynerus*)

##### Distribution

England

#### Symmorphus

Wesmael, 1836

#### Symmorphus bifasciatus

(Linnaeus, 1761)

Vespa bifasciata Linnaeus, 1761
sinuatus (Fabricius, 1793, *Vespa*) preocc.
mutinensis (Baldeni, 1894, *Odynerus*)
sinuatissimus Richards, 1935

##### Distribution

England, Scotland, Wales, Ireland, Isle of Man

#### Symmorphus connexus

(Curtis, 1826)

Odynerus connexus Curtis, 1826
bifasciatus misident.

##### Distribution

England

#### Symmorphus crassicornis

(Panzer, 1798)

Vespa crassicornis Panzer, 1798

##### Distribution

England, Wales

#### Symmorphus gracilis

(Brullé, 1832)

Odynerus gracilis Brullé, 1832

##### Distribution

England, Wales

#### Polistinae

##### Notes

Taxonomy follows Carpenter (1996).

#### Polistes

Latreille, 1802

#### Polistes dominula

(Christ, 1791)

Vespa dominula Christ, 1791
gallicus misident.
italica Herrich-Schäffer, 1840 nom. nud.
pectoralis Herrich-Schäffer, 1841
lefebvrei Guérin, 1844
bucharensis Erichson, 1849
maculatus Rudow, 1889
merceti Dusmet, 1903
rufescens Buysson, 1912
ornata Weyrauch, 1938
pacfica Weyrauch, 1939 preocc.
pseudopacificus Giordani Soika, 1970
muchei Gusenleitner, 1976

##### Distribution

England

#### # Polistes gallicus

(Linnaeus, 1767)

Vespa gallica Linnaeus, 1767
pictior Radoszkowski, 1872
foederata Kohl, 1898
omissa (Weyrauch, 1938, *Polistula*)

##### Distribution

Ireland

##### Notes

Added by McClenaghan (1979) (as *Polistes omissa*), who collected a single male in Co. Down; it is unknown whether a nest had been established but this southern European species is an unlikely colonist.

#### Vespinae

##### Notes

Nomenclature follows Carpenter and Kojima (1997) and Kimsey and Carpenter (2012).

#### Dolichovespula

Rohwer, 1916

PSEUDOVESPULA Bischoff, 1931
BOREOVESPULA Blüthgen, 1943
METAVESPULA Blüthgen, 1943

#### Dolichovespula media

(Retzius, 1783)

Vespa medius Retzius, 1783
geerii (Lepeletier, 1836, *Vespa*)
crassa (Herrich-Schäffer, 1841, *Vespa*)
similis (Schenck, 1853, *Vespa*)
rufoscutellata (Schenck, 1853, *Vespa*)
flavicincta (Schenck, 1853, *Vespa*)
lineolata (Pérez, 1910, *Vespa*)
conjugens Paul, 1943
sugare Ishikawa, 1969
borealis Lee, 1986 preocc.

##### Distribution

England, Scotland, Wales, Isle of Man

##### Notes

added by Falk (1982)

#### Dolichovespula norwegica

(Fabricius, 1781)

Vespa norwegica Fabricius, 1781
britannica (Leach, 1814, *Vespa*)
marginata (Kirby, 1837, *Vespa*) preocc.
borealis (Zetterstedt, 1838, *Vespa*) preocc.
albida (Sladen, 1918, *Vespa*)
arctica (Friese, 1919, *Vespa*) preocc.
zetterstedti Blüthgen, 1937

##### Distribution

England, Scotland, Wales, Ireland, Isle of Man

#### Dolichovespula saxonica

(Fabricius, 1793)

Vespa saxonica Fabricius, 1793
bavarica (von Schrank, 1802, *Vespa*)
tridens (Schenck, 1853, *Vespa*)
nipponica Yamane, 1975
kamtschatkensis Eck, 1983
nigrescens Eck, 1983

##### Distribution

England, Wales

##### Notes

added by Allen and Archer (1989)

#### Dolichovespula sylvestris

(Scopoli, 1763)

Vespa sylvestris Scopoli, 1763
parietum (Harris, 1776, *Vespa*)
holsatica (Fabricius, 1793, *Vespa*)
frontalis (Latreille, 1802, *Vespa*)
campanaria (Fowler, 1833, *Vespa*)
anglica (Smith, 1843, *Vespa*)
pilosella (Costa, 1858, *Vespa*)
sumptuosa (De Buysson, 1905, *Vespa*)
xinjiangensis Lee, 1986

##### Distribution

England, Scotland, Wales, Ireland, Isle of Man

#### Vespa

Linnaeus, 1758

MACROVESPA Dalla Torre, 1904
NYCTOVESPA van der Vecht, 1959

#### Vespa crabro

Linnaeus, 1758

vexator Harris, 1776
major Retzius, 1783
pratensis Geoffroy, 1785
germana Christ, 1791
crabroniformis Smith, 1852
borealis Radoszkowski, 1863
anglica Gribodo, 1892 preocc.
oberthuri du Buysson, 1902
flavo-fasciata Cameron, 1903
tartarea du Buysson, 1905
altaica Pérez, 1910
caspica Pérez, 1910
vulgata Birula, 1925
meridionalis Birula, 1925
chinensis Birula, 1925 preocc.
birulai Bequaert, 1931
gribodoi Bequaert, 1931

##### Distribution

England, Wales

##### Notes

British populations are considered to belong to the subspecies *gribodoi*.

#### Vespula

Thomson, 1869

PSEUDOVESPA Schmiedeknecht, 1881
PARAVESPULA Blüthgen, 1938
ALLOVESPULA Blüthgen, 1943
RUGOVESPULA Archer, 1982

#### Vespula austriaca

(Panzer, 1799)

Vespa austriaca Panzer, 1799
borealis (Smith, 1843, *Vespa*) preocc.
arborea (Smith, 1849, *Vespa*)
biloba (Schilling, 1850, *Vespa*)

##### Distribution

England, Scotland, Wales, Ireland

#### Vespula germanica

(Fabricius, 1793)

Vespa germanica Fabricius, 1793
maculata (Scopoli, 1763, *Vespa*) preocc.
macularis (Olivier, 1792, *Vespa*)

##### Distribution

England, Scotland, Wales, Ireland

#### Vespula rufa

(Linnaeus, 1758)

Vespa rufa Linnaeus, 1758
schrenckii (Radoszkowski, 1861, *Vespa*)
sibiria (André, 1884, *Vespa*)
grahami Archer, 1981
obscura Lee, 1986
yichunensis Lee, 1986

##### Distribution

England, Scotland, Wales, Ireland, Isle of Man

#### Vespula vulgaris

(Linnaeus, 1758)

Vespa vulgaris Linnaeus, 1758
sexcincta (Panzer, 1799, *Vespa*)

##### Distribution

England, Scotland, Wales, Ireland, Isle of Man

## Supplementary Material

Supplementary material 1 Checklist of the British and Irish aculeates Data type: formatted text
Brief description: Word document version of the checklist
File: oo_83170.docx Else, G., Bolton, B. & Broad, G.R.

Supplementary material 2 Checklist of the British and Irish aculeates Data type: spreadsheet
Brief description: Excel spreadsheet version of the checklist
File: oo_83127.xlsx Else, G., Bolton, B. & Broad, G.R.

## Figures and Tables

**Figure 1a.**
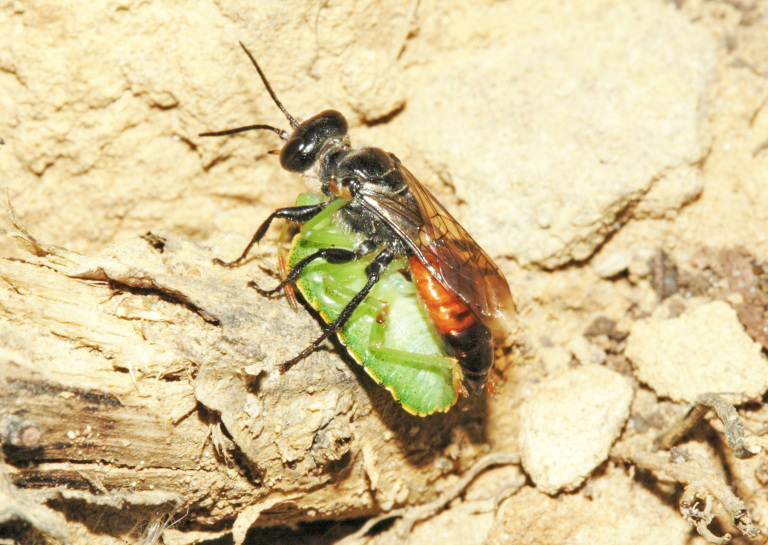
*Astata boops* (Schrank) (Crabronidae) with gorse shieldbug prey (courtesy of J. Early)

**Figure 1b.**
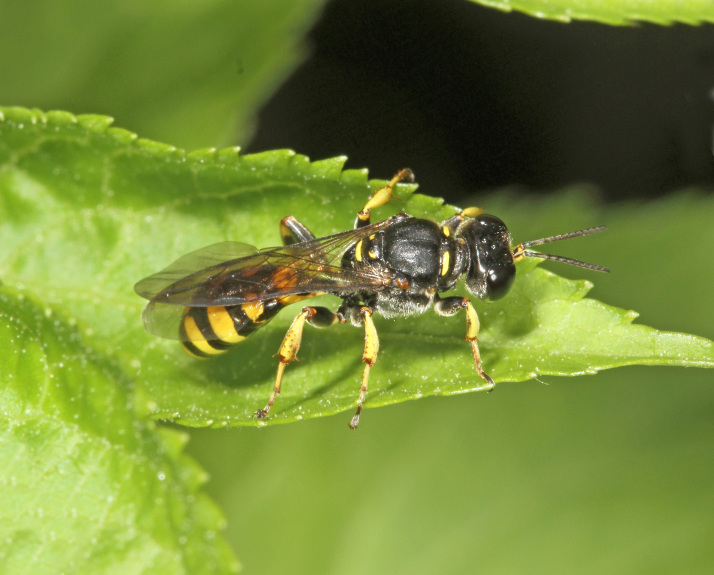
*Crossocerus binotatus* (Lepeletier & Brullé) female (Crabronidae) (courtesy of J. Early)

**Figure 1c.**
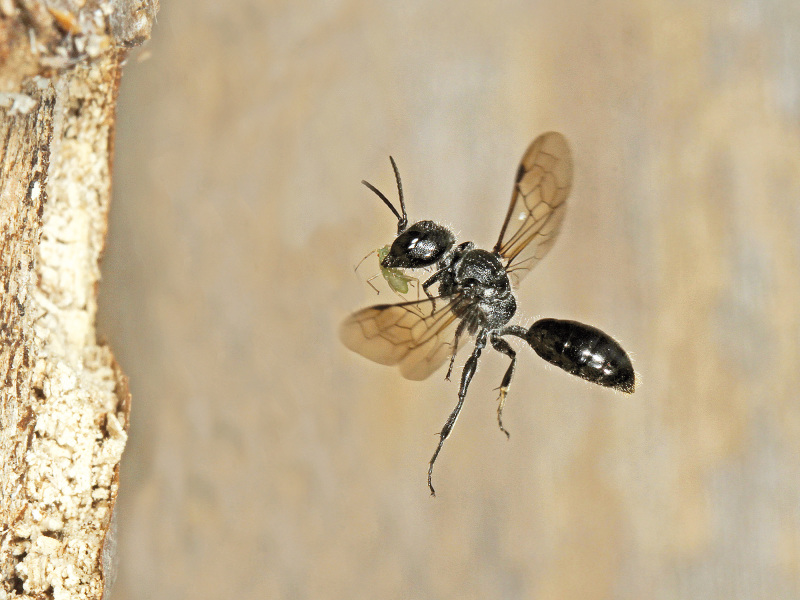
*Pemphredon lugubris* (Fabricius) (Crabronidae) approaching nest with aphid prey (courtesy of J. Early)

**Figure 1d.**
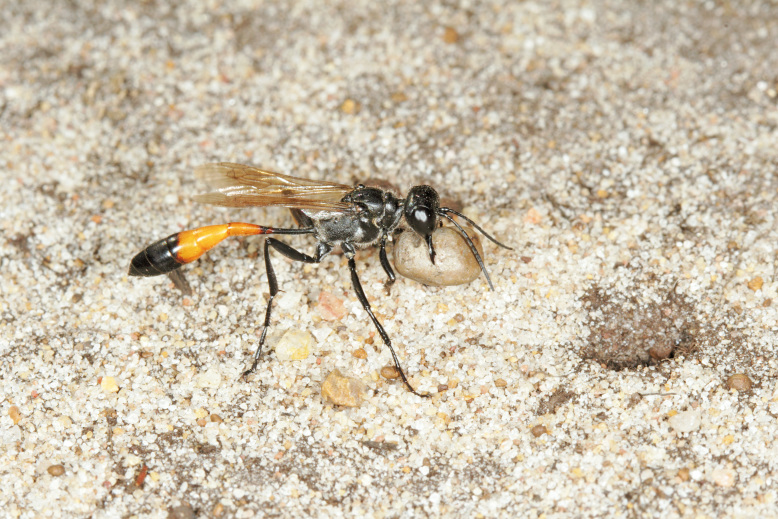
*Ammophila pubescens* Curtis (Sphecidae) with pebble (courtesy of J. Early)

**Figure 2a.**
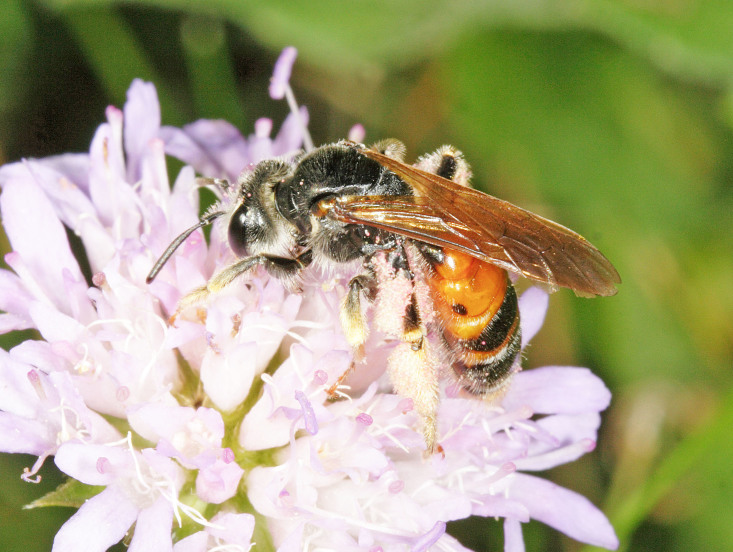
*Andrena hattorfiana* (Fabricius) (Andrenidae) female on *Knautia arvensis* (courtesy of J. Early)

**Figure 2b.**
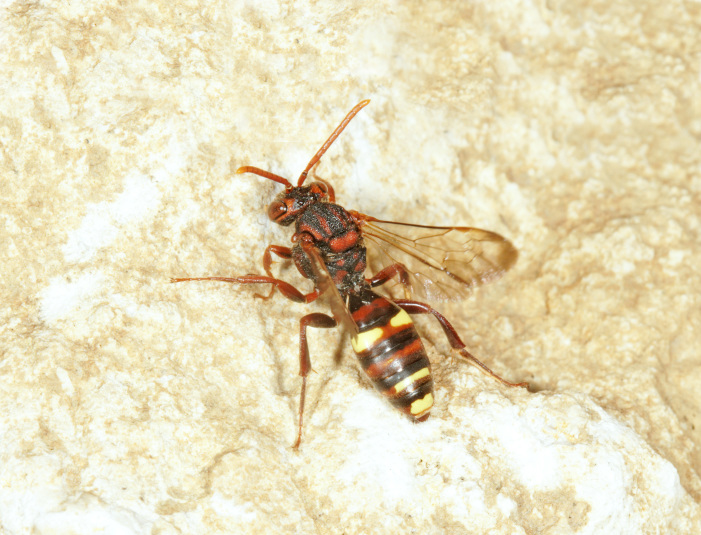
*Nomada panzeri* Lepeletier (Apidae) female (courtesy of J. Early)

**Figure 2c.**
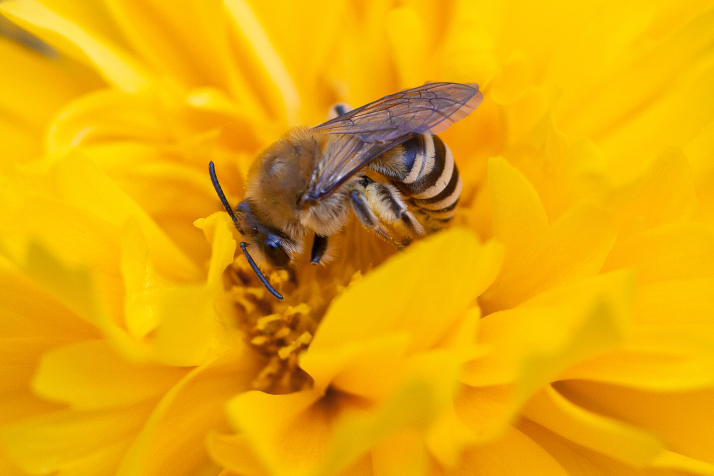
*Colletes* sp. (Colletidae) (courtesy of P. Adams)

**Figure 2d.**
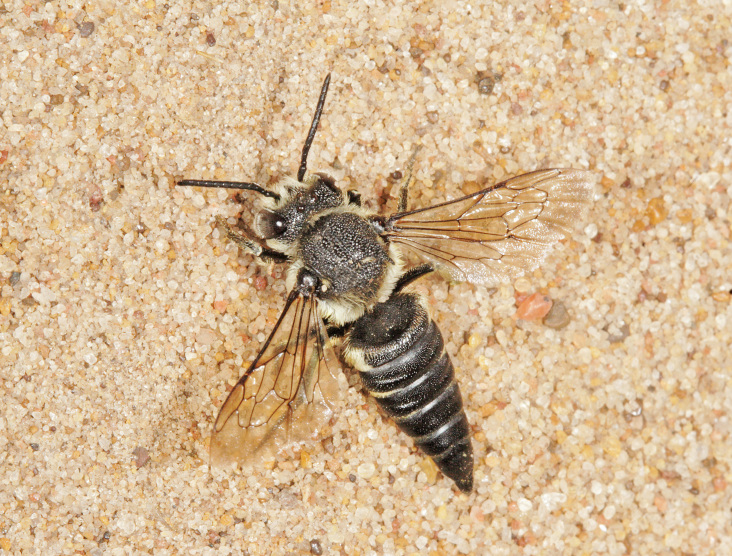
*Coelioxys conoidea* (Illiger) (Megachilidae) female (courtesy of J. Early)

**Figure 3a.**
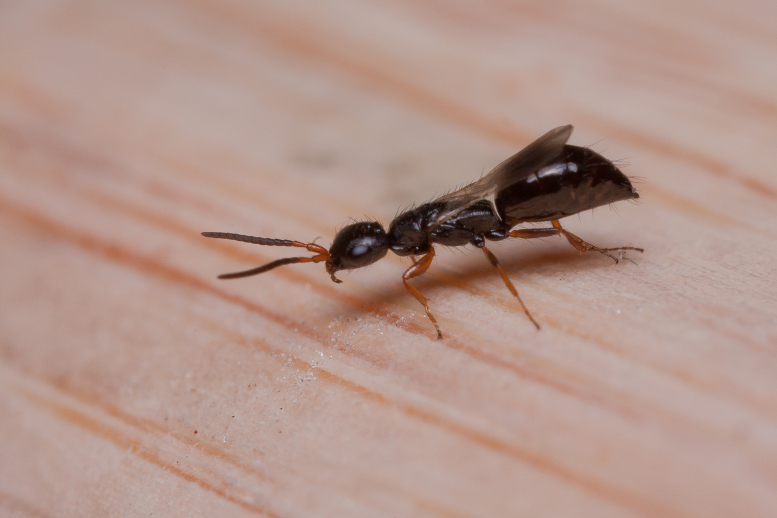
*Laelius* sp. (Bethylidae) female (courtesy of P. Adams)

**Figure 3b.**
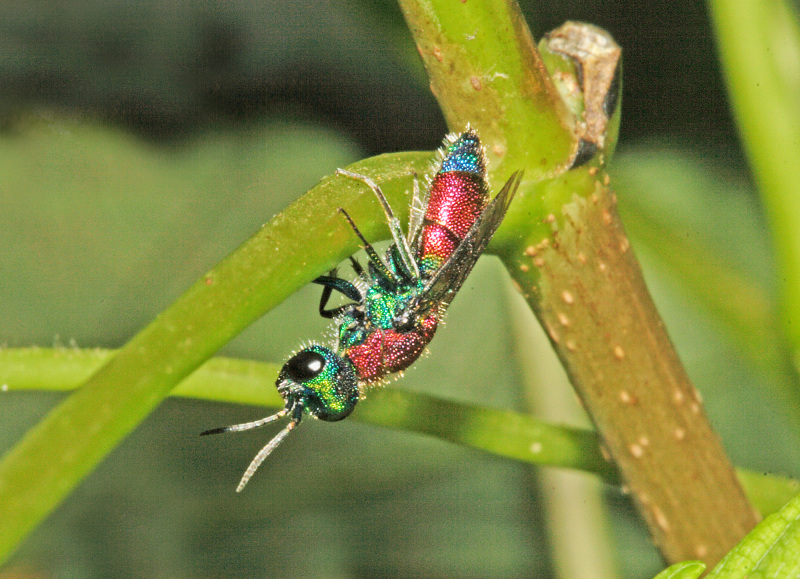
*Chrysis viridula* Linnaeus (Chrysididae) female (courtesy of J. Early)

**Figure 3c.**
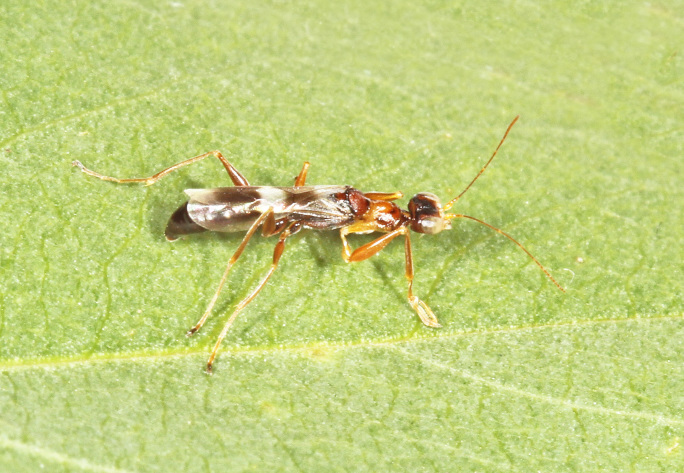
*Dryinus collaris* (Linnaeus) (Dryinidae) female (courtesy of J. Early)

**Figure 4a.**
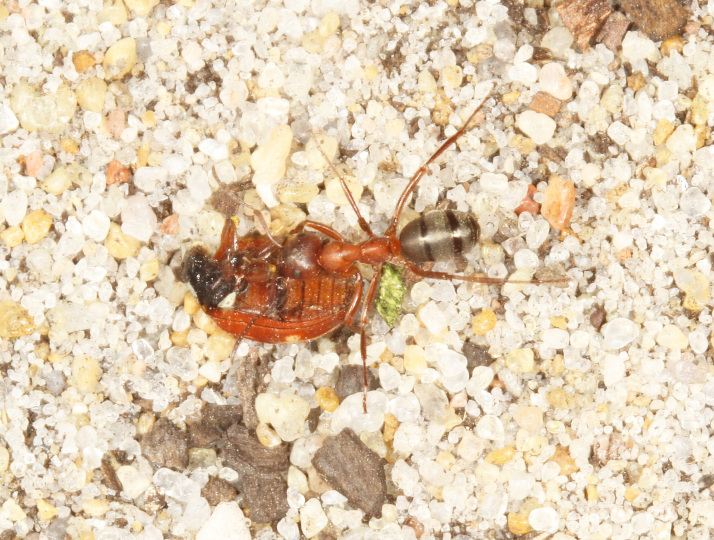
*Formica rufa* Linnaeus (Formicidae) worker with ladybird (courtesy of J. Early)

**Figure 4b.**
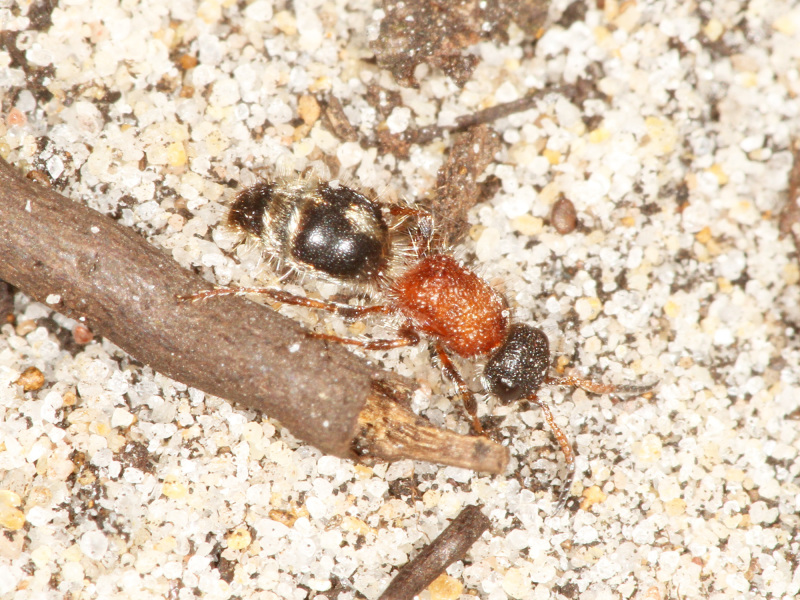
*Smicromyrme rufipes* (Fabricius) (Mutillidae) female (courtesy of J. Early)

**Figure 4c.**
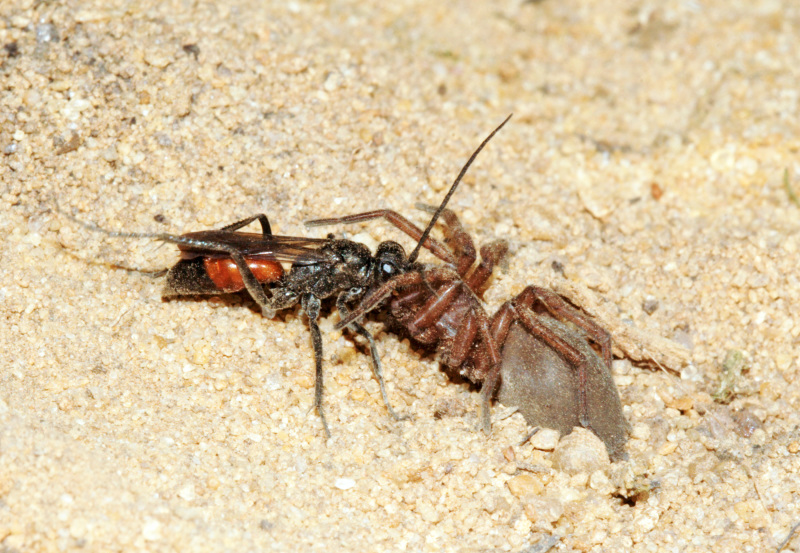
*Cryptocheilus notatus* (Rossi) (Pompilidae) female with *Drassodes cupreus* prey (courtesy of J. Early)

**Figure 4d.**
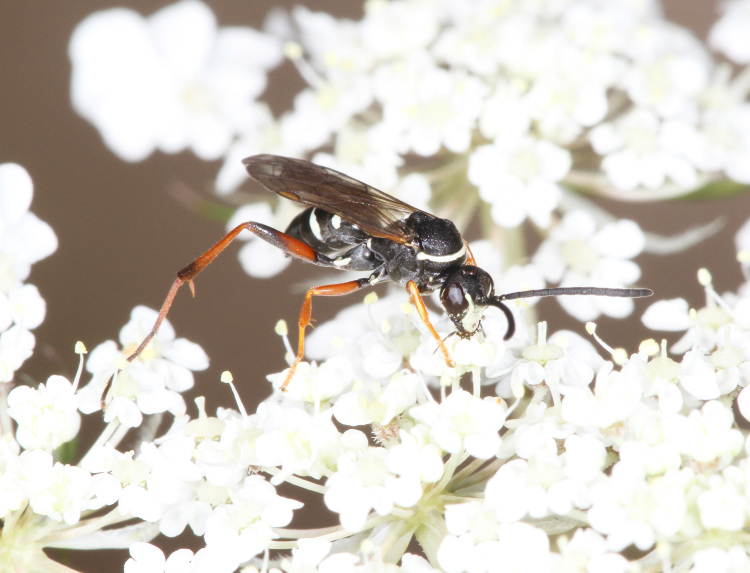
*Ceropales maculata* (Fabricius) (Pompilidae) female (courtesy of J. Early)

**Figure 5a.**
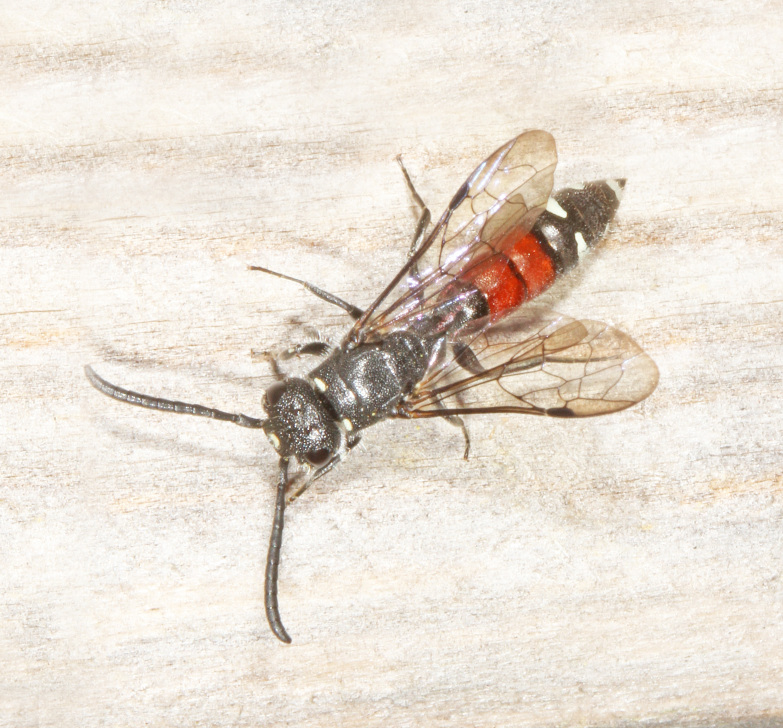
*Sapyga quinquepunctata* (Fabricius) (Sapygidae) female (courtesy of J. Early)

**Figure 5b.**
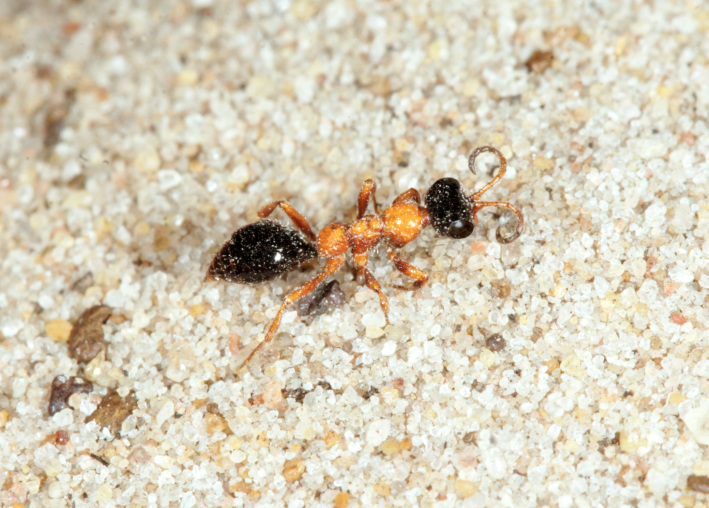
*Methocha articulata* Latreille (Tiphiidae) female (courtesy of J. Early)

**Figure 5c.**
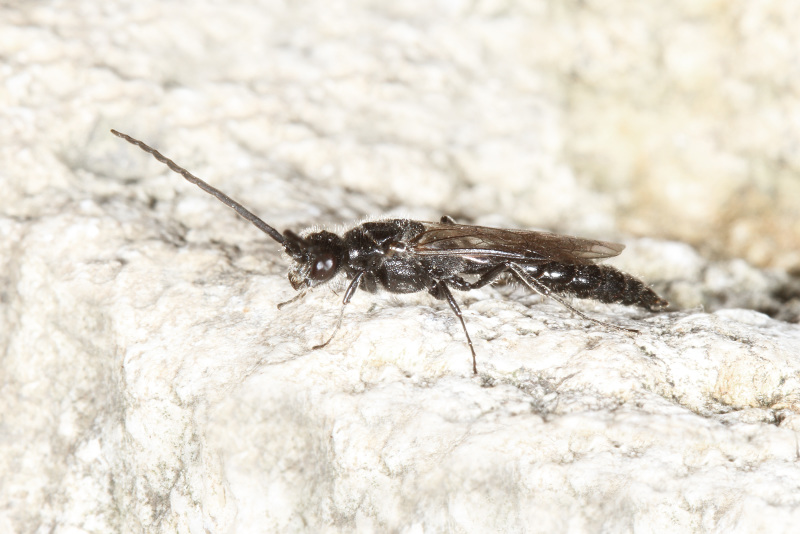
*Methocha articulata* Latreille (Tiphiidae) male (courtesy of J. Early)

**Figure 5d.**
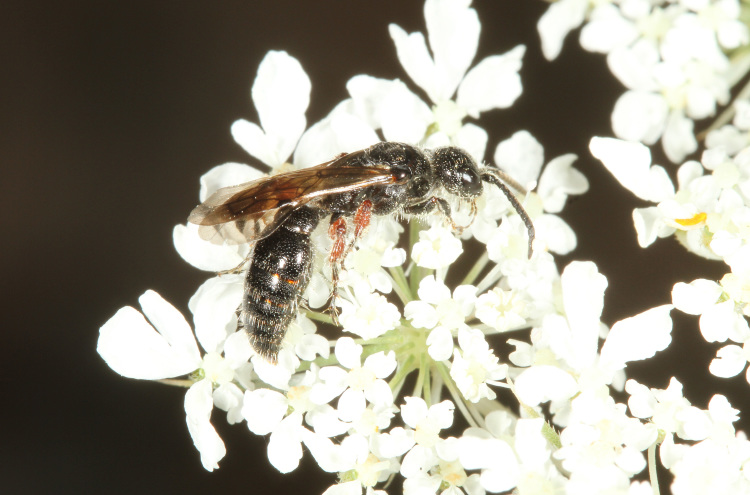
*Tiphia femorata* Fabricius (Tiphiidae) female on *Daucus carota* (courtesy of J. Early)

**Figure 6a.**
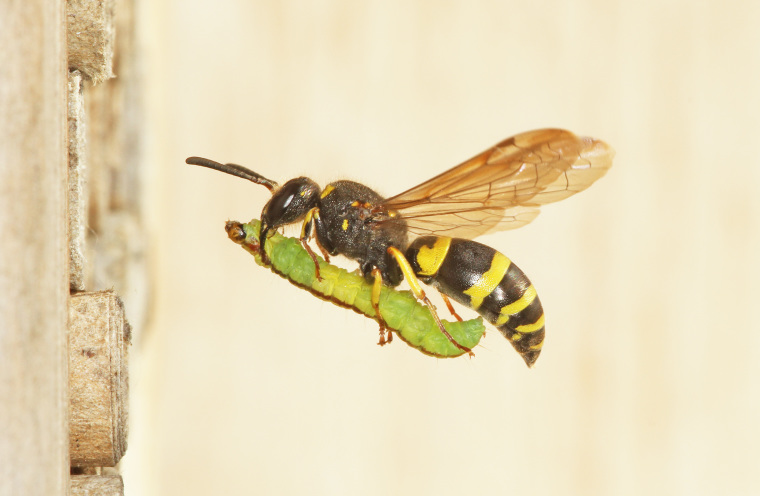
*Ancistrocerus nigricornis* (Curtis) approaching nest with prey (courtesy of J. Early)

**Figure 6b.**
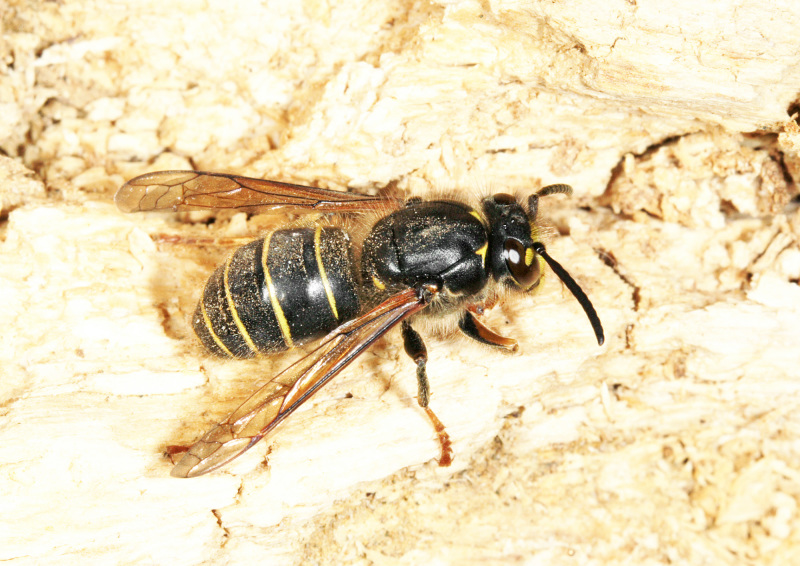
*Dolichovespula media* (Retzius) dark worker (courtesy of J. Early)

**Figure 6c.**
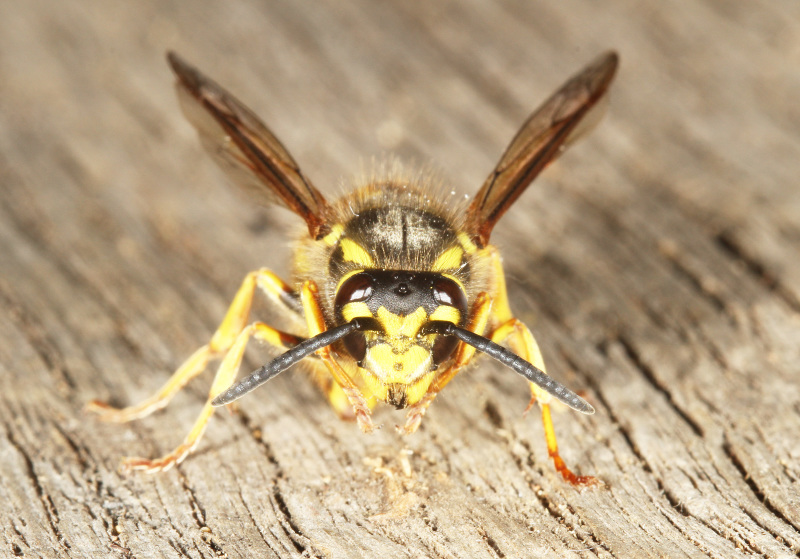
*Dolichovespula saxonica* (Fabricius) queen (courtesy of J. Early)
