# Supplementary material for: Checklist of British and Irish Hymenoptera - aculeates (Apoidea, Chrysidoidea and Vespoidea)
Source: Biodivers Data J. 2016 Apr 7;(4):e8050. doi: 10.3897/BDJ.4.e8050 (PMC4867696; doi:10.3897/BDJ.4.e8050)
Supplement: Supplementary material 1 — Checklist of the British and Irish aculeates [file biodiversity_data_journal-4-e8050-s001.docx]

**‘Aculeates’ – the aculeate Hymenoptera**

George Else, Barry Bolton (Formicidae) & Gavin R. Broad

Distribution data are taken from BWARS atlases (Edwards, 1997, 1998, Edwards & Telfer, 2001, 2002, Edwards & Broad, 2005, 2006, Edwards & Roy, 2009, Collins & Roy, 2012), with some Irish records from O’Connor *et al.* (2009); additional references are given. Synonymies for known introductions have been kept to a minimum.

Superfamily **APOIDEA** Latreille, 1802**^[[1]](#footnote-1)^**

Family **Crabronidae** Latreille, 1802

Subfamily Astatinae Lepeletier, 1845

***ASTATA*** Latreille, 1796

*DIMORPHA* Panzer, 1806

***boops*** (Schrank, 1781, *Sphex*) E

*abdominalis* (Panzer, 1798, *Tiphia*)

*pompiliformis* (Panzer, 1804, *Larra*)

*oculata* (Jurine, 1807, *Dimorpha*)

*victor* Curtis, 1829

*vanderlindeni* Robert, 1833

*agilis* Smith, 1875

***DRYUDELLA*** Spinola, 1843

***pinguis*** (Dahlbom, 1832, *Larra*) E S W

*stigma* misident.

*pinguis* (Zetterstedt, 1838, *Larra*) preocc.

*jaculator* (Smith, 1845, *Astata*)

Subfamily BEMBICINAE Latreille, 1802

Tribe ALYSSONTINI Dalla Torre, 1897

***DIDINEIS*** Wesmael, 1852

***lunicornis*** (Fabricius, 1798, *Pompilus*) E

*kenedii* (Curtis, 1836, *Alyson*)

Tribe BEMBICINI Latreille, 1802

***ARGOGORYTES*** Ashmead, 1899

***fargeii*** (Shuckard, 1837, *Gorytes*) E W

*campestris* misident.

*mongolensis* Tsuneki, 1971

*przewalskyi* Kazenas, 1971

***mystaceus*** (Linnaeus, 1761, *Sphex*) E S W I

*campestris* (Linnaeus, 1761, *Vespa*)

*inimica* (Harris, 1776, *Vespa*)

*longicornis* (Rossi, 1790, *Sphex*)

*bicinctus* (Fabricius, 1793, *Crabro*)

*arpactus* (Fabricius, 1804, *Mellinus*)

*flavicincta* (Donovan, 1808, *Vespa*)

*croceipes* (Eversmann, 1849, *Gorytes*)

*tonsus* (Bondroit, 1933, *Gorytes*)

***GORYTES*** Latreille, 1805

*ARPACTUS* Panzer, 1805

*EUZONIA* Stephens, 1829

*EUSPONGUS* Lepeletier, 1832

*HOPLISUS* Lepeletier, 1832

*LAEVIGORYTES* Zavadil, 1948

***laticinctus*** (Lepeletier, 1832, *Euspongus*) E W

***quadrifasciatus*** (Fabricius, 1804, *Mellinus*) E W

*vicinus* (Lepeletier, 1832, *Euspongus*)

*montivagus* (Mocsáry, 1878, *Hoplisus*)

***HARPACTUS*** Shuckard, 1837

*ARPACTUS* Jurine, 1807 preocc.

*HARPACTES* Dahlbom, 1843

*DIENOPLUS* Fox, 1893

***tumidus*** (Panzer, 1801, *Pompilus*) E S W I M

*japonensis* (Tsuneki, 1963, *Dienoplus*)

*transiliensis* Kazenas, 1989

***LESTIPHORUS*** Lepeletier, 1832

*HYPOMELLINUS* Asmead, 1899

*MELLINOGASTRA* Asmead, 1899

***bicinctus*** (Rossi, 1794, *Crabro*) E

Tribe NYSSONINI Latreille, 1804

***NYSSON*** Latreille, 1796

*SYNNEVRUS* Costa, 1859

***dimidiatus*** Jurine, 1807 E W

*wesmaeli* Lepeletier, 1845

*distinguendus* Chevrier, 1867

***interruptus*** (Fabricius, 1798, *Mellinus*) E

*spinosus* (Fabricius, 1804, *Ceropales*)

*panzeri* Lepeletier, 1845

*shukardi* Wesmael, 1852

***spinosus*** (Forster, 1771, *Sphex*) E S W I

?*bidens* (Linnaeus, 1767, *Vespa*)

*spinosus* (Fabricius, 1775, *Crabro*) preocc.

*tricinctus* (Fabricius, 1793, *Mellinus*)

*trilineata* (Turton, 1802, *Vespa*)

*geniculatus* Lepeletier, 1845

*malasei* Gussakovskij, 1932

***trimaculatus*** (Rossi, 1790, *Crabro*) E W

Subfamily Crabroninae Latreille, 1802

Tribe CrabroninI Latreille, 1802

***CRABRO*** Fabricius, 1775

*THYREOPUS* Lepeletier & Brullé, 1835

*ANOTHYREUS* Dahlbom, 1845

*THYREOCNEMUS* Costa, 1871

*PARANOTHYREUS* Ashmead, 1899

*SYNOTHYREOPUS* Ashmead, 1899

*AGNOSICRABRO* Pate, 1944

*DYSCOLOCRABRO* Pate, 1944

*HEMITHYREOPUS* Pate, 1944

*NORUMBEGA* Pate, 1947

*PARANOTHYREUS* Pate, 1944

*PARATHYREOPUS* Pate, 1944

*PEMPHILIS* Pate, 1944

*OTHYREUS* Marshakov, 1977

***cribrarius*** (Linnaeus, 1758, *Vespa*) E S W M

*patellarius* (Schreber, 1784, *Sphex*)

*argus* (Christ, 1791, *Sphex*)

*longa* (Christ, 1791, *Sphex*)

*lunatus* (Christ, 1791, *Sphex*)

*palmatus* Panzer, 1797

*inornatus* Mocsáry, 1901

*hypotheticus* Kokujev, 1927

***peltarius*** (Schreber, 1784, *Sphex*) E S W I M

*patellatus* Panzer, 1797

*dentipes* Panzer, 1797

*mediatus* Fabricius, 1798

***scutellatus*** (von Scheven, 1781, *Sphex*) E

*scutularius* (Schreber, 1784, *Sphex*)

*pterotus* Panzer, 1801

*reticulatus* (Lepeletier & Brullé, 1835, *Ceratocolus*)

***CROSSOCERUS*** Lepeletier & Brullé, 1835

subgenus ***ABLEPHARIPUS*** Perkins, 1913

***congener*** (Dahlbom, 1844, *Crabro*) E added by Archer (2007)^[[2]](#footnote-2)^

***podagricus*** (Vander Linden, 1829, *Crabro*) E W

*vicinus* Dahlbom, 1842

*punctata* Šnoflak, 1948

*snoflaki* Zavadil, 1948

subgenus ***ACANTHOCRABRO*** Perkins, 1913

*CORENOCRABRO* Tsuneki, 1974

***vagabundus*** (Panzer, 1798, *Crabro*) E W

*varus* (Panzer, 1799, *Crabro*) preocc.

*bojus* (Schrank, 1802, *Crabro*)

*quinquemaculatus* (Lepeletier & Brullé, 1835, *Blepharipus*)

*lefebvrei* Lepeletier & Brullé, 1835

*fasciatus* (Costa, 1871, *Crabro*)

*esakii* (Yasumatsu, 1942, *Crabro*)

*ectemiformis* (Tsuneki, 1974, *Corenocrabro*)

subgenus ***BLEPHARIPUS*** Lepeletier & Brullé, 1835

*COELOCRABRO* Thomson, 1874

***annulipes*** (Lepeletier & Brullé, 1835, *Blepharipus*) E S W

*gonager* (Lepeletier & Brullé, 1835, *Blepharipus*)

*nigritus* (Gimmerthal, 1836, *Crabro*)

*ambiguus* (Dahlbom, 1842, *Crabro*)

*capito* (Dahlbom, 1845, *Crabro*)

*parkeri* (Banks, 1921, *Blepharipus*)

*davidsoni* (Sandhouse, 1938, *Crabro*)

***capitosus*** (Shuckard, 1837, *Crabro*) E S W I

*annulus* (Dahlbom, 1838, *Crabro*)

*yezo* Tsuneki, 1960

***cetratus*** (Shuckard, 1837, *Crabro*) E W I

*vanderlindeni* (Dahlbom, 1838, *Crabro*)

*dilatatus* (Herrich-Schäffer, 1841, *Crabro*)

*inornatus* (Matsumura, 1912, *Crabro*)

*dentsukanus* Tsuneki, 1976

***leucostomus*** (Linnaeus, 1758, *Sphex*) E S

*carbonarius* (Dahlbom, 1838, *Crabro*)

*rugosus* (Herrich-Schäffer, 1841, *Crabro*)

*melanarius* (Wesmael, 1852, *Crabro*)

*cinctipes* (Provancher, 1882, *Blepharipus*)

*niger* (Provancher, 1888, *Crabro*)

*nigror* (Fox, 1895, *Crabro*)

*servus* (Dalla Torre, 1897, *Crabro*)

*cinctitarsis* (Ashmead, 1901, *Stenocrabro*)

*columbiae* (Bradley, 1906, *Blepharipus*)

*stygius* (Mickel, 1916, *Thyreopus*)

*utensis* (Mickel, 1916, *Thyreopus*)

***megacephalus*** (Rossi, 1790, *Crabro*) E S W I M

*leucostoma* misident.

*bidens* (Haliday, 1833, *Crabro*)

*niger* Lepeletier & Brullé, 1835

*rufipes* Lepeletier & Brullé, 1835

*laeviceps* (Smith, 1856, *Crabro*)

*bison* (Costa, 1884, *Crabro*)

?*zaidamensis* (Radoszkowski, 1887, *Crabro*)

*leucostomoides* (Richards, 1935, *Coelocrabro*)

***nigritus*** (Lepeletier & Brullé, 1835, *Blepharipus*) E W

*pubescens* (Shuckard, 1837, *Crabro*)

*diversipes* Herrich-Schäffer, 1841

*inermis* (Thomson, 1870, *Crabro*)

*melanogaster* Kohl, 1880

*nigricornis* (Provancher, 1888, *Blepharipus*)

*sambucicola* (Verhoeff, 1891, *Crabro*)

*verhoeffi* Tsuneki, 1967

*sudai* Tsuneki, 1976

*babai* Tsuneki, 1979

***styrius*** (Kohl, 1892, *Crabro*) E

*pauxillus* (Gussakovskij, 1932, *Crabro*)

*sugiharai* (Iwata, 1938, *Crabro*)

*pilicornis* Tsuneki, 1977

***walkeri*** (Shuckard, 1837, *Crabro*) E S W

*aphidium* misident.

*geniculatus* (Shuckard, 1837, *Crabro*)

*clypearis* (Schenck, 1857, *Crabro*)

*cloevorax* (Nielson, 1901, *Crabro*)

subgenus ***CROSSOCERUS*** Lepeletier & Brullé, 1835

*STENOCRABRO* Ashmead, 1899

*ISCHNOLYNTHUS* Holmberg, 1903

***distinguendus*** (Morawitz, 1866, *Crabro*) E added by Packer (1981)

*mucronatus* (Thomson, 1870, *Crabro*)

***elongatulus*** (Vander Linden, 1829, *Crabro*)^[[3]](#footnote-3)^ E S W M

*annulatus* Lepeletier & Brullé, 1835

*varipes* Lepeletier & Brullé, 1835

*affinis* Lepeletier & Brullé, 1835

*luteipalpis* Lepeletier & Brullé, 1835

*morio* Lepeletier & Brullé, 1835

*pallidipalpis* Lepeletier & Brullé, 1835

*proximus* (Shuckard, 1837, *Crabro*)

*hyalinus* (Shuckard, 1837, *Crabro*)

*transversalis* (Shuckard, 1837, *Crabro*)

*obliquus* (Shuckard, 1837, *Crabro*)

*propinquus* (Shuckard, 1837, *Crabro*)

*brevis* (Eversmann, 1849, *Crabro*)

*scutellaris* (Smith, 1851, *Crabro*)

*sulcus* (Fox, 1895, *Crabro*)

*plesius* (Rohwer, 1912, *Stenocrabro*)

*berlandi* (Richards, 1928, *Crabro*)

***exiguus*** (Vander Linden, 1829, *Crabro*) E

*aphidum* Lepeletier & Brullé, 1835

***ovalis*** Lepeletier & Brullé, 1835 E S W

*punctum* (Zetterstedt, 1838, *Crabro*)

*anxius* (Wesmael, 1852, *Crabro*)

*shuckardi* (Smith, 1856, *Crabro*)

*ovatus* (Schulz, 1906, *Crabro*)

***palmipes*** (Linnaeus, 1767, *Sphex*) E S M

*palmarius* (Schreber, 1784, *Sphex*)

*scutatus* (Fabricius, 1787, *Crabro*)

*ornatus* Lepeletier & Brullé, 1835

*scutellaris* (Gimmerthal, 1836, *Crabro*)

*gracilis* (Eversmann, 1849, *Crabro*)

*decoratus* (Smith, 1856, *Crabro*)

***tarsatus*** (Shuckard, 1837, *Crabro*) E S W I M

*palmipes* misident.

*palmatus* De Stefani Perez, 1884 preocc,

***varus*** Lepeletier & Brullé, 1835 *nomen protectum* E S W I M

*varius* misspelling

*pusillus* Lepeletier & Brullé, 1835

*striatulus* Lepeletier & Brullé, 1835

*spinipectus* (Shuckard, 1837, *Crabro*)

*striatus* Lepeletier, 1845

*intricatus* (Smith, 1856, *Crabro*)

*lepeletieri* (Smith, 1856, *Crabro*)

***wesmaeli*** (Vander Linden, 1829, *Crabro*) E S W I

*maurus* (Lepeletier & Brullé, 1835, *Ceratocolus*)

*ziegleri* (Lepeletier & Brullé, 1835, *Ceratocolus*)

subgenus ***CUPHOPTERUS*** Morawitz, 1866

***binotatus*** Lepeletier & Brullé, 1835 E W

*signatus* (Panzer, 1798, *Crabro*) preocc.

*monstrosus* (Dahlbom, 1845, *Crabro*)

*confusus* (Schulz, 1906, *Crabro*)

***dimidiatus*** (Fabricius, 1781, *Crabro*) E S W I

*subpunctatus* (Rossi, 1790, *Crabro*)

*sexmaculatus* (Olivier, 1792, *Crabro*)

*signatus* (Olivier, 1792, *Crabro*)

*serripes* (Panzer, 1797, *Crabro*)

*notatus* (Illiger, 1807, *Crabro*)

*pauperatus* (Lepeletier & Brullé, 1835, *Blepharipus*)

*armipes* (von Sebold, 1844, *Crabro*)

subgenus ***HOPLOCRABRO*** Thomson, 1874

***quadrimaculatus*** (Fabricius, 1793, *Crabro*) E S W I

*quadripunctatus* (Fabricius, 1793, *Crabro*)

*murorum* (Latreille, 1805, *Crabro*)

*levipes* (Vander Linden, 1829, *Crabro*)

*bimaculatus* Lepeletier & Brullé, 1835

*quniquemaculatus* (Dahlbom, 1838, *Crabro*)

*rotundarius* (Dahlbom, 1838, *Crabro*)

***ECTEMNIUS*** Dahlbom, 1845

subgenus ***CLYTOCHRYSUS*** Morawitz, 1864

***cavifrons*** (Thomson, 1870, *Crabro*) E S W I M

*cephalotes* misident.

***lapidarius*** (Panzer, 1804, *Crabro*) E S W I M

?*cinctus* (Spinola, 1806, *Crabro*)

*chrysostomus* (Lepeletier & Brullé, 1835, *Crabro*)

*comptus* (Lepeletier & Brullé, 1835, *Crabro*)

*xylurgus* (Shuckard, 1837, *Crabro*)

*interstinctus* (Smith, 1856, *Crabro*)

*obscurus* (Smith, 1856, *Crabro*)

*gracilissimus* (Packard, 1866, *Crabro*)

*denticulatus* (Packard, 1866, *Crabro*)

*effosus* (Packard, 1866, *Crabro*)

*papagorum* (Viereck, 1908, *Crabro*)

***ruficornis*** (Zetterstedt, 1838, *Crabro*) E W

*aurilabris* (Herrich-Schäffer, 1841, *Crabro*)

*nigrifrons* (Cresson, 1865, *Crabro*)

*contiguus* (Cresson, 1865, *Crabro*)

*septentrionalis* (Packard, 1866, *Crabro*)

*planifrons* (Thomson, 1870, *Crabro*)

*longipalpis* (Verhöff, 1892, *Crabro*)

*lineatotarsis* (Matsumura, 1911, *Crabro*)

*chipsani* (Matsumura, 1912, *Crabro*)

***sexcinctus*** (Fabricius, 1775, *Crabro*) E W

*planifrons* misident.

*quadricinctus* (Fabricius, 1787, *Crabro*)

*interruptefasciatus* (Retzius, 1783, *Sphex*)

*tibialis* (Olivier, 1792, *Crabro*)

*8 maculata* (Preyssler, 1793, *Crabro*)

*zonatus* (Panzer, 1797, *Crabro*)

*vespiformis* (Panzer, 1798, *Crabro*)

*octomaculatus* (Schrank, 1802, *Crabro*)

*cinctus* (Spinola, 1806, *Crabro*)

*flavipes* (Lepeletier & Brullé, 1835, *Crabro*)

*tetraedrus* (Blanchard, 1940, *Crabro*)

*saundersi* (Perkins, 1899, *Crabro*)

*yosemite* Pate, 1946

subgenus ***ECTEMNIUS*** Dahlbom, 1845

***borealis*** (Zetterstedt, 1838, *Crabro*) E

*bipunctatus* (Zetterstedt, 1838, *Crabro*)

*nigrinus* (Herrich-Schäffer, 1841, *Crabro*)

*parvulus* (Packard, 1866, *Crabro*)

*gredleri* (Kohl, 1878, *Lindenius*)

*proletarius* (Mickel, 1916, *Crabro*)

***dives*** (Lepeletier & Brullé, 1835, *Solenius*) E W

*octonotatus* (Lepeletier & Brullé, 1835, *Solenius*)

*octavonotatus* (Lepeletier & Brullé, 1835, *Solenius*)

*alatulus* (Dahlbom, 1838, *Crabro*)

*pictipes* (Herrich-Schäffer, 1841, *Crabro*)

*auratus* (Smith, 1856, *Crabro*)

*montanus* (Cresson, 1865, *Crabro*) preocc.

*cristatus* (Packard, 1866, *Crabro*)

*cubiceps* (Packard, 1866, *Crabro*)

*heraclei* (Rohwer, 1908, *Crabro*)

*montivagans* (Strand, 1916, *Crabro*)

subgenus ***HYPOCRABRO*** Ashmead, 1899

*XESTOCRABRO* Ashmead, 1899

***continuus*** (Fabricius, 1804, *Crabro*) E S W I

*vagus* misident.

*sexmaculatus* (Say, 1824 *Crabro*) preocc.

*fuscitarsis* (Herrich-Schäffer, 1841, *Crabro*)

*vagatus* (Smith, 1869, *Crabro*)

*pumilus* (Costa, 1871, *Crabro*)

*granulatus* (Walker, 1871, *Crabro*)

*rugopunctatus* (Taschenberg, 1875, *Crabro*)

*validus* (De Stefani Perez, 1884, *Crabro*)

*bizexmaculatus* (Viereck, 1910, *Crabro*)

*sayi* (Cockerell, 1910, *Crabro*)

*giffardi* (Rohwer, 1917, *Solenius*)

***rubicola*** (Dufour & Perris, 1840, *Solenius*) E W

*microstictus* (Herrich-Schäffer, 1841, *Crabro*)

*larvatus* (Wesmael, 1852, *Crabro*)

*pumilus* (Costa, 1871, *Crabro*)

subgenus ***METACRABRO*** Ashmead, 1899

***cephalotes*** (Olivier, 1792, *Crabro*) E S W I

*quadricinctus* misident.

*floralis* (Olivier, 1792, *Crabro*)

*geniculatus* (Olivier, 1792, *Crabro*)

*tibialis* (Olivier, 1792, *Crabro*)

?*cephalotes* (Panzer, 1799, *Crabro*)

*striatus* (Lepeletier & Brullé, 1835, *Crabro*)

*ornatus* (Lepeletier & Brullé, 1835, *Crabro*)

*striatulus* (Lepeletier & Brullé, 1835, *Blepharipus*)

*lindenius* (Shuckard, 1837, *Crabro*)

*shuckardi* (Dahlbom, 1838, *Crabro*)

*interruptus* (Dahlbom, 1845, *Crabro*) preocc.

*fargeii* (Smith, 1856, *Crabro*)

*aciculatus* (Provancher, 1882, *Crabro*)

*ruthenicus* (Morawitz, 1892, *Crabro*)

***lituratus*** (Panzer, 1805, *Crabro*) E W

*petiolatus* (Lepeletier & Brullé, 1835, *Solenius*)

*fasciatus* (Lepeletier & Brullé, 1835, *Ceratocolus*)

*reticulatus* (Lepeletier & Brullé, 1835, *Ceratoculus* [*lapsus*])

*kollari* (Dahlbom, 1845, *Crabro*)

*argenteus* (Schenck, 1857, *Crabro*)

*vestitus* (Smith, 1858, *Crabro*)

*intermedius* (Morawitz, 1866, *Crabro*)

*luxuriosus* (Costa, 1871, *Crabro*)

***ENTOMOGNATHUS*** Dahlbom, 1844

*KOXINGA* Pate, 1944

*MASHONA* Pate, 1944

*TONCAHUA* Pate, 1944

*FLORKINUS* Leclercq, 1956

*BIHARGNATHUS* Leclercq, 1977

***brevis*** (Vander Linden, 1829, *Crabro*) E W

*apicalis* (Lepeletier & Brullé, 1835, *Lindenius*)

*nasutus* (Gribodo, 1884, *Lindenius*)

***LESTICA*** Billberg, 1820

subgenus ***CLYPEOCRABRO*** Richards, 1935

*THYREUS* Lepeletier & Brullé, 1835 preocc.

***clypeata*** (Schreber, 1759, *Apis*) E

*ovata* (Christ, 1791, *Sphex*)

*vexillata* (Panzer, 1797, *Crabro*)

*lapidaria* (Fabricius, 1804, *Crabro*) preocc.

*clypeata* (Thunberg, 1815, *Philanthus*)

*nigridens* (Herrich-Schäffer, 1841, *Crabro*)

*quadrifer* (Dufour, 1841, *Crabro*)

***LINDENIUS*** Lepeletier & Brullé, 1835

*CHALCOLAMPRUS* Wesmael, 1852

*TRACHELOSIMUS* Morawitz, 1866

***albilabris*** (Fabricius, 1793, *Crabro*) E S W

*aenescens* (Dahlbom, 1838, *Crabro*)

***panzeri*** (Vander Linden, 1829, *Crabro*) E

*venustus* Lepeletier & Brullé, 1835

*latebrosus* (Kohl, 1905, *Crabro*)

*harbinensis* Tsuneki, 1967

*mongolicus* Tsuneki, 1972

***pygmaeus*** (Rossi, 1794, *Crabro*)^[[4]](#footnote-4)^ E

*curtus* Lepeletier & Brullé, 1835

*kratochvili* (Šnoflak, 1948, *Crabro*)

***RHOPALUM*** Stephens, 1829

subgenus ***RHOPALUM*** Stephens, 1829

*EUPLILIS* Risso, 1826 nom. ob.

*PHYSOSCELUS* Lepeletier & Brullé, 1835

***clavipes*** (Linnaeus, 1758, *Sphex*) E S W I M

*rufiventris* (Panzer, 1799, *Crabro*)

subgenus ***CORYNOPUS*** Lepeletier & Brullé, 1835

*DRYPHUS* Herrich-Schäffer, 1840

*ALLIOGNATHUS* Ashmead, 1899

***coarctatum*** (Scopoli, 1763, *Sphex*) E S W I

*crassipes* (Fabricius, 1798, *Crabro*)

*tibiale* (Fabricius, 1798, *Crabro*) preocc.

*modestum* Rohwer, 1908

***gracile*** Wesmael, 1852 E

*nigrinum* Kiesenwetter, 1849 preocc.

*kiesenwetteri* (Morawitz, 1866, *Crabro*)

*simplicipes* (Morawitz, 1888, *Corynopus*)

Tribe LARRINI Latreille, 1810

***TACHYSPHEX*** Kohl, 1883

*SCHISTOSPHEX* Arnold, 1922

*ATELOSPHEX* Arnold, 1923

***nitidus*** (Spinola, 1805, *Astata*) E W M added by Edwards (1998)^[[5]](#footnote-5)^

*unicolor* misident.

*ibericus* (de Saussure, 1867, *Tachytes*)

***obscuripennis*** (Schenck, 1857, *Tachytes*) E^[[6]](#footnote-6)^

*lativalvis* (Thomson, 1870, *Tachytes*)

***pompiliformis*** (Panzer, 1805, *Larra*) E S W I M

*pectinipes* misident.

*nigripennis* (Spinola, 1808, *Tachytes*)

*dimidiata* (Panzer, 1809, *Larra*)

*jokischiana* (Panzer, 1809, *Larra*)

*parvula* (Cresson, 1865, *Larrada*)

*quebecensis* (Provancher, 1882, *Larra*)

*decorus* Fox, 1894

*tenuipunctus* Fox, 1894

*consimilis* Fox, 1894

*rufoniger* Bingham, 1897

*projectus* Nurse, 1903

*argyrotrichus* Rohwer, 1911

*granulosus* Mickel, 1916

*erythraeus* Mickel, 1916

*angularis* Mickel, 1916

***unicolor*** (Panzer, 1809, *Larra*) E

*nitidus* misident.

*jurinii* (Drapiez, 1819, *Larra*)

Tribe MISCOPHINI Fox, 1894

***MISCOPHUS*** Jurine, 1807

*NITELOPTERUS* Ashmead, 1897

*HYPOMISCOPHUS* Cockerell, 1898

*MISCOPHINUS* Ashmead, 1898

***ater*** Lepeletier, 1845 E

*maritimus* Smith, 1858

***bicolor*** Jurine, 1807 E added by Knowles & Else (2006)

*dubius* (Panzer, 1809, *Larra*)

*metallicus* Verhöff, 1890

*tsunekii* de Andrade, 1960

***concolor*** Dahlbom, 1844 E

*bicolor* misident.

*moravicus* Balthazar, 1957

***NITELA*** Latreille, 1809

*TENILA* Brèthes, 1913

*RHINONITELA* Williams, 1928

***borealis*** Valkeila, 1974 E added by Felton (1987)

***lucens*** Gayubo & Felton, 2000 E added by Felton (1987)

*spinolae* misident.^[[7]](#footnote-7)^

Tribe OXYBELINI Leach, 1815

***OXYBELUS*** Latreille, 1796

*NOTOGLOSSA* Dahlbom, 1845

*ALEPIDASPIS* Costa, 1882

*ANOXYBELUS* Kohl, 1924

*GONIOXYBELUS* Pate, 1937

*ORTHOXYBELUS* Pate, 1937

*LATROXYBELUS* Noskiewicz & Chudoba, 1950

***argentatus*** Curtis, 1833 E W

*mucronatus* misident.

?*decimmaculata* (Donovan, 1806, *Vespa*)

*ferox* Shuckard, 1837

*nigricornis* Shuckard, 1837

***mandibularis*** Dahlbom, 1845 E S W M

*sericatus* Gerstäcker, 1867

***uniglumis*** (Linnaeus, 1758, *Vespa*) E S W I M

*punctata* (Fabricius, 1793, *Nomada*)

*tridens* (Fabricius, 1798, *Crabro*)

?*decimmaculata* (Donovan, 1806, *Vespa*)

*pygmaeus* Olivier, 1812

*quadrinotatus* Say, 1824

*impatiens* Smith, 1856

*interruptus* Cresson, 1865

*fallax* Gerstäcker, 1867

*brodiei* Provancher, 1883

*hispanicus* Giner Marí, 1943

Tribe TRYPOXYLINI Lepeletier, 1845

***TRYPOXYLON*** Latreille, 1796

*APIUS* Panzer, 1806

*APIUS* Jurine, 1807 preocc.

*TRYPARGILUM* Richards, 1934

*ASACONOTON* Arnold, 1959

***attenuatum*** Smith, 1851 E S W I

***clavicerum*** Lepeletier & Serville, 1828 E W I

*tibiale* Zetterstedt, 1840

*batumicum* Antropov, 1985

***figulus*** (Linnaeus, 1758, *Sphex*) E W

*fuliginosa* (Scopoli, 1763, *Sphex*)

*apicale* Fox, 1891

*yezo* Tsuneki, 1956

*barbarum* de Beaumont, 1957

*fieuzeti* Giner Marí, 1959

***medium*** de Beaumont, 1945 E S W added by Pulawski (1984)

***minus*** de Beaumont, 1945 E added by Felton (1988)

*koma* Tsuneki, 1956

Subfamily DINETINAE Fox, 1895

***DINETUS*** Panzer, 1806

*DINETUS* Jurine, 1807 preocc.

***pictus*** (Fabricius, 1793, *Crabro*) E

*guttatus* (Fabricius, 1793, *Sphex*) preocc.

Subfamily Mellininae Latreille, 1802

***MELLINUS*** Fabricius, 1790

***arvensis*** (Linnaeus, 1758, *Vespa*) E S W I M

*vagus* (Linnaeus, 1758, *Sphex*)

*superbus* (Harris, 1776, *Vespa*)

*tricinctus* (Schrank, 1781, *Vespa*)

*clavatus* (Retzius, 1783, *Sphex*)

?*infundibuliformis* (Geoffroy, 1785, *Vespa*)

?*petiolatus* (Geoffroy, 1785, *Vespa*)

*bipunctatus* (Fabricius, 1787, *Crabro*)

?*gibbus* (Villers, 1789, *Sphex*) preocc.

*melanostictus* (Gmelin, 1790, *Vespa*)

?*arthriticus* (Rossi, 1790, *Crabro*)

?*rachiticus* (Rossi, 1790, *Crabro*)

*annularis* (Christ, 1791, *Sphex*)

*succinctus* (Olivier, 1792, *Vespa*)

*diversus* (Olivier, 1792, *Vespa*)

*labiatus* (Olivier, 1792, *Crabro*)

*quinquemaculatus* (Fabricius, 1793, *Philanthus*)

*u-flavum* (Panzer, 1794, *Crabro*)

*capistratus* (Schrank, 1796, *Crabro*)

*pratensis* Jurine, 1807

*compactus* Handlirsch, 1888

***crabroneus*** (Thunberg, 1791, *Sphex*) E W

*sabulosus* (Fabricius, 1787, *Crabro*) preocc.

*ruficornis* (Villers, 1789, *Sphex*) preocc.

*sabulosus* (Olivier, 1792, *Crabro*) preocc.

*ruficornis* Fabricius, 1793 preocc.

*frontalis* (Panzer, 1797, *Crabro*)

*petiolatus* (Panzer, 1797, *Crabro*)

*fulvicornis* Fabricius, 1804

Subfamily Pemphredoninae Dahlbom, 1835

Tribe PemphredoninI Dahlbom, 1835

***DIODONTUS*** Curtis, 1834

*XYLOCELIA* Rohwer, 1915

*NEODIODONTUS* Tsuneki, 1972

*CORENIUS* Tsuneki, 1974

***insidiosus*** Spooner, 1938 E

*friesei* misident.

***luperus*** Shuckard, 1837 E W

***minutus*** (Fabricius, 1793, *Crabro*) E W M

*franclemonti* Krombein, 1939

***tristis*** (Vander Linden, 1829, *Pemphredon*) E S W

***PASSALOECUS*** Shuckard, 1837

*XYLOECUS* Shuckard, 1837

*COELOECUS* Verhöff, 1890

*HEROECUS* Verhöff, 1890

***clypealis*** Faester, 1947 E

*angustus* Gussakovskij, 1952

***corniger*** Shuckard, 1837 E W

***eremita*** Kohl, 1893 E added by Richards (1980)

***gracilis*** (Curtis, 1834, *Diodontus*) E

*insignis* misident.

*turionum* misident.

*brevicornis* Morawitz, 1864

***insignis*** (Vander Linden, 1829, *Pemphredon*) E W

*roettgeni* Verhöff, 1890

*shuckardi* Yasumatsu, 1934

***monilicornis*** Dahlbom, 1842 E S W I

***singularis*** Dahlbom, 1844 E W

*gracilis* misident.

*tenuis* Morawitz, 1864

*gertrudis* Krombein, 1938

***turionum*** Dahlbom, 1844 E added by Guichard (2002)

***PEMPHREDON*** Latreille, 1796^[[8]](#footnote-8)^

*CEMONUS* Panzer, 1806

*DINEURUS* Westwood, 1837

*CERATOPHORUS* Shuckard, 1837

*DIPHLEBUS* Westwood, 1840

*CHEVRIERIA* Kohl, 1883

*SUSANOWO* Tsuneki, 1972

***austriaca*** (Kohl, 1888, *Diphlebus*) E

*enslini* misident.^[[9]](#footnote-9)^

*coracina* Valkeila, 1972

*tener* Valkeila, 1972

*nescia* Merisuo, 1972

***inornata*** Say, 1824 E S W I

*shuckardi* (Morawitz, 1864, *Cemonus*)

*dentata* (Puton, 1871, *Cemonus*)

*tenax* Fox, 1829

***lethifer*** (Shuckard, 1837, *Cemonus*) E S W I M

*lethifera* misspelling

*austriaca* misident.

*strigatus* (Chevrier, 1870, *Cemonus*)

*fabricii* (Müller, 1911, *Cemonus*)

*fuscatus* (Wagner, 1918, *Diphlebus*)

*littoralis* (Wagner, 1918, *Diphlebus*)

*neglectus* (Wagner, 1918, *Diphlebus*)

*minutus* (Wagner, 1918, *Diphlebus*)

*enslini* Wagner, 1931

*brevipetiolata* Wagner, 1932

*platyura* Gussakovskij, 1952

*minor* Gussakovskij, 1952

*levinota* Merisuo, 1972

*nannophyes* Merisuo, 1972

*dispar* Valkeila, 1972

*gemina* Valkeila, 1972

*trichogastor* Valkeila, 1972

?*sudaorum* Tsuneki, 1977

***lugubris*** (Fabricius, 1793, *Crabro*) E S W I M

*concolor* Say, 1824

*ocellaris* Gimmerthal, 1836

*luctuosa* Shuckard, 1837

*morio* Cresson, 1865 preocc.

*cressoni* Dalla Torre, 1897

*provancheri* Dalla Torre, 1897

*tinctipennis* Cameron, 1908

*shawii* Rohwer, 1917

*pacifica* Gussakovkij, 1932

***morio*** Vander Linden, 1829 E W

*anthracinus* (Smith, 1851, *Ceratophorus*)

*carinata* Thomson, 1870

*clypealis* Thomson, 1870

*intermedia* Tsuneki, 1951

***rugifer*** (Dahlbom, 1844, *Cemonus*) E S

*rugifera* misspelling

*unicolor* (Panzer, 1798, *Sphex*) preocc.

*pilosa* (Gimmerthal, 1836, *Cemonus*)

*wesmaeli* (Morawitz, 1864, *Cemonus*)

*scotica* Perkins, 1929

*solivaga* (Bondroit, 1931, *Cemonus*)

*bucharicus* Gussakovskij, 1952

*mortifer* Valkeila, 1972

*punctifer* Valkeila, 1972

*scytica* Valkeila, 1972

***SPILOMENA*** Shuckard, 1838

*CELIA* Shuckard, 1837 preocc.

*MICROGLOSSA* Rayment, 1930 preocc.

*MICROGLOSSELLA* Rayment, 1935

*TAIALIA* Tsuneki, 1971

***beata*** Blüthgen, 1953 E

*exspectata* Valkeila, 1957

***differens*** Blüthgen, 1953^[[10]](#footnote-10)^ E

*curruca* misident.

***curruca*** (Dahlbom, 1844, *Celia*) E

*pulawskii* Dolfuss, 1983

*nikkoensis* Tsuneki, 1971

***enslini*** Blüthgen, 1953 E I

***troglodytes*** (Vander Linden, 1829, *Stigmus*) E S W

*minutissimus* (Radoszkowski, 1877, *Stigmus*)

*vagans* Blüthgen, 1953

***STIGMUS*** Panzer, 1805

*ANTRONIUS* Zetterstedt, 1838

*GONOSTIGMUS* Rohwer, 1911

*ATOPOSTIGMUS* Krombein, 1973

***pendulus*** Panzer, 1804 E added by Allen (1987)

*ater* Jurine, 1807

***solskyi*** Morawitz, 1864 E W

*europaeus* Tsuneki, 1954

*verhoeffi* Tsuneki, 1954

Tribe PSENINI Costa, 1858

***MIMESA*** Shuckard, 1837

*APORIA* Wesmael, 1852 preocc.

*APORINA* Gussakovskij, 1937 preocc.

***bicolor*** (Jurine, 1807, *Psen*) E

*equestris* misident.

?*rufa* (Panzer, 1805, *Psen*)

***bruxellensis*** Bondroit, 1934 E W

*rossicus* (Gussakovskij, 1937, *Psen*)

***equestris*** (Fabricius, 1804, *Trypoxylon*) E S W

*bicolor* misident.

***lutaria*** (Fabricius, 1787, *Sphex*) E W

*shuckardi* Wesmael, 1852

*basirufa* Packard, 1867

*nebrascensis* Smith, 1908

*dispar* (Gussakovskij, 1937, *Psen*)

*mallochi* Finnamore, 1980

***MIMUMESA*** Malloch, 1933

***atratina*** (Morawitz, 1891, *Mimesa*) E

*carbonaria* (Tournier, 1899, *Mimesa*)

*belgica* (Bondroit, 1932, *Mimesa*)

*longula* (Gussakovskij, 1932, *Mimesa*)

*sameshimai* (Yasumatsu, 1937, *Psen*)

***dahlbomi*** (Wesmael, 1852, *Mimesa*) E S W

***littoralis*** (Bondroit, 1934, *Mimesa*) E W I

*unicolor* misident.

*fulvitarsis* (Gussakovskij, 1934, *Psen*)

*fulvitarsis* (Gussakovskij, 1937, *Psen*) preocc.

*celtica* (Spooner, 1948, *Mimesa*)

***spooneri*** (Richards, 1948, *Mimesa*) E

***unicolor*** (Vander Linden, 1829, *Psen*) E added by Else & Felton (1994)

*borealis* (Dahlbom, 1842, *Mimesa*)

*palliditarsis* (Saunders, 1904, *Mimesa*)

*oresterus* (van Lith, 1976, *Psen*)

***PSEN*** Latreille, 1796

*PSENUS* Rafinesque, 1815

*PSENIA* Stephens, 1829

*DAHLBOMIA* Wissmann, 1849

*MESOPORA* Wesmael, 1852

*CAENOPSEN* Cameron, 1899

***ater*** (Olivier, 1792, *Crabro*) E

*ater* (Fabricius, 1794, *Sphex*) preocc.

*compressicornis* (Fabricius, 1804, *Pelopoeus*)

*atratus* (Jurine, 1807, *Psen*)

*serraticornis* (Jurine, 1807, *Psen*)

***PSENULUS*** Kohl, 1897

*DIODONTUS* misident.^[[11]](#footnote-11)^

*NEOFOXIA* Viereck, 1901

*STENOMELLINUS* Schulz, 1911

*EOPSENULUS* Gussakovskij, 1934

*NIPPONOPSEN* Yasumatsu, 1938

***concolor*** (Dahlbom, 1843, *Psen*) E W

*intermedius* (Schenck, 1857, *Psen*)

*ambiguus* (Schenck, 1857, *Psen*)

***pallipes*** (Panzer, 1798, *Sphex*) E W I

*atratus* (Fabricius, 1804, *Trypoxylon*)

*montanus* (Costa, 1861, *Psen*)

*haemorrhoidalis* (Costa, 1871, *Psen*)

*minutus* (Tournier, 1899, *Psen*)

*chevrieri* (Tournier, 1899, *Psen*)

*nigricornis* (Tournier, 1899, *Psen*)

*pygmaeus* (Tournier, 1899, *Psen*)

*rubicola* Harttig, 1931

*brevitarsis* Merisuo, 1937

***schencki*** (Tournier, 1889, *Psen*) E

*simplex* (Tournier, 1899, *Psen*)

*longulus* (Tournier, 1899, *Psen*)

Subfamily Philanthinae Latreille, 1802

Tribe CERCERINI Lepeletier, 1845

***CERCERIS*** Latreille, 1802

*NECTANEBUS* Spinola, 1839

*DIAMMA* Dahlbom, 1844 preocc.

*DIDESMUS* Dahlbom, 1845

*APIRAPTRIX* Shestakov, 1923

*PARACERCERIS* Brèthes, 1913

*BUCERCERIS* Minkiewicz, 1934

*STERCOBATA* Gussakovskij, 1935

*APICERCERIS* Pate, 1937

***arenaria*** (Linnaeus, 1758, *Sphex*) E W

*xanthocephala* (Forster, 1771, *Sphex*)

*exulta* (Harris, 1776, *Vespa*)

*petulans* (Harris, 1776, *Vespa*)

*serripes* (Fabricius, 1781, *Vespa*)

*arenosa* (Gmelin, 1790, *Vespa*)

*aurita* (Fabricius, 1794, *Philanthus*)

*striolata* Schletterer, 1887

***quadricincta*** (Panzer, 1799, *Philanthus*) E

*fasciata* Spinola, 1806

***quinquefasciata*** (Rossi, 1792, *Crabro*) E

*interrupta* misident.

*nasuta* Dahlbom, 1844 preocc.

*subdepressa* Lepeletier, 1845

***ruficornis*** (Fabricius, 1793, *Philanthus*) E

*labiata* misident.

*bidens* (Schrank, 1802, *Crabro*)

*cunicularia* (Schrank, 1802, *Crabro*)

*quadricincta* (Fabricius, 1804, *Mellinus*)

*trifidus* (Fabricius, 1804, *Philanthus*)

?*nasuta* Latreille, 1809

*laminifera* Costa, 1867

***rybyensis*** (Linnaeus, 1771, *Sphex*) E

*ornata* (Fabricius, 1790, *Philanthus*)

*apifalco* (Christ, 1791, *Sphex*)

*semicincta* (Panzer, 1797, *Philanthus*)

*hortorum* (Panzer, 1799, *Philanthus*)

*variabilis* (Schrank, 1802, *Crabro*)

*biguttata* (Thunberg, 1815, *Philanthus*)

*colon* (Thunberg, 1815, *Philanthus*)

*kashmirensis* Nurse, 1903

*dittrichi* Schulz, 1904

*reginae* Eck, 1979

***sabulosa*** (Panzer, 1799, *Philanthus*) E

*emarginata* (Panzer, 1799, *Philanthus*)

*tricincta* (Spinola, 1805, *Philanthus*)

?*pygmaea* (Thunberg, 1815, *Philanthus*)

*minuta* Lepeletier, 1845

*superba* Shestakov, 1923

Tribe PhilanthinI Latreille, 1802

***PHILANTHUS*** Fabricius, 1790

*SYMBLEPHILUS* Panzer, 1806

*SIMBLEPHILUS* Jurine, 1807

*CHEILOPOGONUS* Westwood, 1835

*ANTHOPHILUS* Dahlbom, 1844

*EPHIPHILANTHUS* Ashmead, 1899

*PSEUDOPHILANTHUS* Ashmead, 1899

*OCLOCLETES* Banks, 1913

***triangulum*** (Fabricius, 1775, *Vespa*) E W

*ruspatrix* (Linnaeus, 1767, *Vespa*) nom. ob.

*fasciatus* (Geoffroy, 1785, *Vespa*)

*maculatus* (Christ, 1791, *Sphex*)

*limbatus* (Olivier, 1792, *Vespa*)

*androgynus* (Rossi, 1792, *Crabro*)

*pictus* Panzer, 1797

*discolor* Panzer, 1799

*apivorus* Latreille, 1799

*allionii* Dahlbom, 1845

Family **Sphecidae** Latreille, 1802

Subfamily AMMOPHILINAE André, 1886

***AMMOPHILA*** Kirby, 1798

*SPHEX* misident.

*MISCUS* Jurine, 1807

*COLOPTERA* Latreille, 1845

*ARGYRAMMOPHILA* Gussakovskij, 1928

*APYCNEMIA* Leclercq, 1961

***pubescens*** Curtis, 1836 E W

*campestris* misident.

*arvensis* (Dahlbom, 1843, *Miscus*)

*susterai* Šnoflak, 1943

*adriaansei* Wilcke, 1945

***sabulosa*** (Linnaeus, 1758, *Sphex*) E S W I

*hortensis* (Poda, 1761, *Sphex*)

*frischii* (Geoffroy, 1785, *Ichneumon*)

*dimidiata* (Christ, 1791, *Sphex*) preocc.

*vulgaris* Kirby, 1798

*pulvillata* Sowerby, 1805

*mucronata* (Jurine, 1807, *Sphex*)

*cyanescens* Dahlbom, 1845

?*vischu* Cameron, 1889

*kamtschatica* Gussakovskij, 1932

***PODALONIA*** Fernald, 1927

*PSAMMOPHILA* Dahlbom, 1842 preocc.

*PODALONIA* Spinola, 1853 suppressed

***affinis*** (Kirby, 1798, *Ammophila*) E W

*lutaria* misident.

*ariasi* (Mercet, 1906, *Ammophila*)

***hirsuta*** (Scopoli, 1763, *Sphex*) E W

*viatica* misident.

*arenaria* (Fabricius, 1787, *Sphex*) preocc.

*arenosa* (Gmelin, 1790, *Sphex*)

*argentea* (Kirby, 1798, *Ammophila*)

**Anthophila Latreille, 1804^[[12]](#footnote-12)^**

Family **Andrenidae** Latreille, 1802

Subfamily Andreninae Latreille, 1802

***ANDRENA*** Fabricius, 1775^[[13]](#footnote-13)^

subgenus ***ANDRENA*** Fabricius, 1775

*ANTHRENA* Illiger, 1801

*ANTHOCHARESSA* Gistel, 1850

***apicata*** Smith, 1847 E W I

***clarkella*** (Kirby, 1802, *Melitta*) E S W I M

***fucata*** Smith, 1847 E S W I M

***fulva*** (Müller, 1766, *Apis*) E S W I

*armata* (Gmelin, 1790, *Apis*)

***helvola*** (Linnaeus, 1758, *Apis*) E S W

*subdentata* (Kirby, 1802, *Melitta*)

***lapponica*** Zetterstedt, 1838 E S W I M

***praecox*** (Scopoli, 1763, *Apis*) E W I

*smithella* (Kirby, 1802, *Melitta*)

*clypeata* Smith, 1855 preocc.

***synadelpha*** Perkins, 1914 E S W

*ambigua* Perkins, 1895, preocc.

***varians*** (Kirby, 1802, *Melitta*) E W

*varians* (Rossius, 1792, *Apis*): misident.

*angulosa* (Kirby, 1802, *Melitta*)

subgenus ***CHARITANDRENA*** Hedicke, 1933

***hattorfiana*** (Fabricius, 1775, *Nomada*) E W

*lathamana* (Kirby, 1802, *Melitta*)

subgenus ***CHLORANDRENA*** Pérez, 1890

***humilis*** Imhoff, 1832 E W M

*fulvescens* Smith, 1847

subgenus ***CHRYSANDRENA*** Hedicke, 1933

***fulvago*** (Christ, 1791, *Apis*) E W

*constricta* Smith, 1849

subgenus ***CNEMIDANDRENA*** Hedicke, 1933

***denticulata*** (Kirby, 1802, *Melitta*) E S W I M

*listerella* (Kirby, 1802, *Melitta*)

***fuscipes*** (Kirby, 1802, *Melitta*) E S W I M

***nigriceps*** (Kirby, 1802, *Melitta*) E SW

*lanifrons* (Kirby, 1802, *Melitta*)

***simillima*** Smith, 1851 E

***tridentata*** (Kirby, 1802, *Melitta*) E^[[14]](#footnote-14)^

*rufitarsis* (Kirby, 1802, *Melitta*)

subgenus ***EUANDRENA*** Hedicke, 1933

*XANTHANDRENA* Lanham, 1949

*GEANDRENA* LaBerge, 1964

***bicolor*** Fabricius, 1775 E S W I M

*picicornis* (Kirby, 1802, *Melitta*)

*pilosula* (Kirby, 1802, *Melitta*)

*gwynana* (Kirby, 1802, *Melitta*)

*proxima* Smith, 1847 preocc.

*aestiva* Smith, 1849

*consimilis* Smith, 1849 preocc.

***ruficrus*** Nylander, 1848 E S

subgenus ***HOLANDRENA*** Pérez, 1890

***labialis*** (Kirby, 1802, *Melitta*) E W M

*separata* Smith, 1847

subgenus ***HOPLANDRENA*** Pérez, 1890

***bucephala*** Stephens, 1846 E W

*eximia* Smith, 1847

*longipes* Smith, 1847

***ferox*** Smith, 1847 E

*distincta* Smith, 1847

***rosae*** Panzer, 1801 E W I

*eximia* misident.

*zonalis* (Kirby, 1802, *Melitta*)

*stragulata* Illiger, 1806^[[15]](#footnote-15)^

*strangulata* misspelling

***scotica*** Perkins, 1916^[[16]](#footnote-16)^ E S W I M

*carantonica* misident.

*jacobi* Perkins, 1921

*johnsoni* Perkins, 1921

***trimmerana*** (Kirby, 1802, *Melitta*) E W

*spinigera* (Kirby, 1802, *Melitta*)

*carantonica* Peréz, 1916

subgenus ***LEUCANDRENA*** Hedicke, 1933

***argentata*** Smith, 1844 E

***barbilabris*** (Kirby, 1802, *Melitta*) E S W I M

*sericea* (Christ, 1791, *Apis*) preocc.

*albicrus* (Kirby, 1802, *Melitta*)

subgenus ***MARGANDRENA*** Warncke, 1968

***marginata*** Fabricius, 1776 E S W I

*cetii* (Schrank, 1781, *Apis*)

*schrankella* (Kirby, 1802, *Melitta*)

*frontalis* Smith, 1849

subgenus ***MELANDRENA*** Pérez, 1890

*GYMNANDRENA* Hedicke, 1933

*CRYPTANDRENA* Lanham, 1949 preocc.

*BYTHANDRENA* Lanham, 1950

***cineraria*** (Linnaeus, 1758, *Apis*) E S W I M

***nigroaenea*** (Kirby, 1802, *Melitta*)^[[17]](#footnote-17)^ E S W I M

*aprilina* Smith, 1848

***nitida*** (Müller, 1776, *Apis*) E W I

*pubescens* Olivier, 1789

*consimilis* Smith, 1847

***thoracica*** (Fabricius, 1775, *Apis*) E W

*melanocephala* (Kirby, 1802, *Melitta*)

***vaga*** Panzer, 1799 E^[[18]](#footnote-18)^

subgenus ***MICRANDRENA*** Ashmead, 1899

*ANDRENELLA* Hedicke, 1933

***alfkenella*** Perkins, 1914 E

*moricella* Perkins, 1914

***falsifica*** Perkins, 1915 E

***floricola*** Eversmann, 1852^[[19]](#footnote-19)^

***minutula*** (Kirby, 1802, *Melitta*) E W I

*parvula* (Kirby, 1802, *Melitta*)

*nigrifrons* Smith, 1855 preocc.

***minutuloides*** Perkins, 1914 E

*parvuloides* Perkins, 1914

***nana*** (Kirby, 1802, *Melitta*) E^[[20]](#footnote-20)^

***nanula*** Nylander, 1848^[[21]](#footnote-21)^

***niveata*** Friese, 1887 E W

*spreta* misident.

***semilaevis*** Pérez, 1903 E S W I M

*nana* misident.

*saundersella* Perkins, 1914

***subopaca*** Nylander, 1848 E S W I

subgenus ***NOTANDRENA*** Pérez, 1890

***chrysosceles*** (Kirby, 1802, *Melitta*) E S W

*connectens* (Kirby, 1802, *Melitta*)

***nitidiuscula*** Schenck, 1853 E

*lucens* Imhoff, 1868

subgenus ***OREOMELISSA*** Hirashima & Tadauchi, 1975

***coitana*** (Kirby, 1802, *Melitta*) E S W I M

*shawella* (Kirby, 1802, *Melitta*)

subgenus ***PLASTANDRENA*** Hedicke, 1933

*SCHIZANDRENA* Hedicke, 1933

*GLYPHANDRENA* Hedicke, 1933

*MITSUKURIELLA* Hirashima & LaBerge, 1965

*MITSUKURIAPIS* Hirashima, LaBerge & Ikudome 1994

***bimaculata*** (Kirby, 1802, *Melitta*) E W

*articulata* Smith, 1847

*conjuncta* Smith, 1847

*decorata* Smith, 1847

*vitrea* Smith, 1847

***nigrospina*** Thomson, 1872^[[22]](#footnote-22)^ E

***pilipes*** Fabricius, 1781 E

*carbonaria* misident.

*spectabilis* Smith, 1853

*praetexta* Smith, 1872

***tibialis*** (Kirby, 1802, *Melitta*) E

*mouffetella* (Kirby, 1802, *Melitta*)

*atriceps* (Kirby, 1802, *Melitta*)

subgenus ***POECILANDRENA*** Hedicke, 1933

***labiata*** Fabricius, 1781 E W I

*cingulata* misident.

subgenus ***POLIANDRENA*** Warncke, 1968

***florea*** Fabricius, 1793 E

*rubricata* Smith, 1847

***polita*** Smith, 1847 E^[[23]](#footnote-23)^

subgenus ***PROXIANDRENA*** Schmid-Egger, 2005

***proxima*** (Kirby, 1802, *Melitta*) E W

*digitalis* (Kirby, 1802, *Melitta*)

subgenus ***PTILANDRENA*** Robertson, 1902

*EREMANDRENA* LaBerge, 1964

***angustior*** (Kirby, 1802, *Melitta*) E S W I M

*lacinia* Smith, 1847

subgenus ***SIMANDRENA*** Pérez, 1890

*PLATANDRENA* Viereck, 1924

*STENANDRENA* Timberlake, 1949

***congruens*** Schmiedeknecht, 1884 E W

*confinis* Stöckhert, 1930

***dorsata*** (Kirby, 1802, *Melitta*) E W

*collinsonana* (Kirby, 1802, *Melitta*)

*lewinella* (Kirby, 1802, *Melitta*)

*nudiuscula* (Kirby, 1802, *Melitta*)

*subincana* (Kirby, 1802, *Melitta*)

***lepida*** Schenck, 1861 E^[[24]](#footnote-24)^

subgenus ***TAENIANDRENA*** Hedicke, 1933

***lathyri*** Alfken, 1899 E

***ovatula*** (Kirby, 1802, *Melitta*) E W

*afzeliella* (Kirby, 1802, *Melitta*)

*barbata* (Kirby, 1802, *Melitta*)

*fuscata* (Kirby, 1802, *Melitta*)

*picipes* (Kirby, 1802, *Melitta*)

*albofasciata* Thomson, 1870

***similis*** Smith, 1849 E S W

?*ocreata* Christ, 1791 nom. dub.

***wilkella*** (Kirby, 1802, *Melitta*) E S W I M

*barbatula* (Kirby, 1802, *Melitta*)

*convexiuscula* (Kirby, 1802, *Melitta*)

*xanthura* (Kirby, 1802, *Melitta*)

subgenus ***TARSANDRENA*** Osytshnjuk, 1984

***tarsata*** Nylander, 1848 E S W I M

*analis* misident.

subgenus ***TRACHANDRENA*** Robertson, 1902

***haemorrhoa*** (Fabricius, 1781, *Apis*) E S W I M

*albicans* misident.

subgenus ***ZONANDRENA*** Hedicke, 1933

***flavipes*** Panzer, 1799 E W I

*contigua* (Kirby, 1802, *Melitta*)

*fulvicrus* (Kirby, 1802, *Melitta*)

*extricata* Smith, 1849

***gravida*** Imhoff, 1832 E

*fasciata* Nylander, 1852 preocc.

*picicrus* Schenck, 1853

Subfamily PANURGINAE Leach, 1815

Tribe PANURGINI Leach, 1815

***PANURGUS*** Panzer, 1806

***banksianus*** (Kirby, 1802, *Apis*) E W

*ursinus* misident.

***calcaratus*** (Scopoli, 1763, *Apis*) E W

*linnaeella* (Kirby, 1802, *Apis*)

Family **Apidae** Latreille, 1802

Subfamily Apinae Latreille, 1802

Tribe ANTHOPHORINI Dahlbom, 1835

***ANTHOPHORA*** Latreille, 1803

subgenus ***ANTHOPHORA*** Latreille, 1803

***plumipes*** (Pallas, 1772, *Apis*) E W

*acervorum* misident.

*pilipes* (Fabricius, 1775, *Apis*)

subgenus ***CLISODON*** Patton, 1879

***furcata*** (Panzer, 1798, *Apis*) E S W

subgenus ***DASYMEGILLA*** Brooks, 1988

***quadrimaculata*** (Panzer, 1798, *Apis*) E W

*subglobosa* (Kirby, 1802, *Apis*)

subgenus ***HELIOPHILA*** Klug, 1807

*SAROPODA* Latreille, 1809

***bimaculata*** (Panzer, 1798, *Apis*) E I

subgenus ***PYGANTHOPHORA*** Brooks, 1988

***retusa*** (Linnaeus, 1758, *Apis*) E

*haworthana* (Kirby, 1802, *Apis*)

*pennipes* (Kirby, 1802, *Apis*)

Tribe ApinI Latreille, 1802

***APIS*** Linnaeus, 1758

***mellifera*** Linnaeus, 1758^[[25]](#footnote-25)^ E S W I M

*mellifica* Linnaeus, 1761

Tribe BOMBINI Latreille, 1802

***BOMBUS*** Latreille, 1802^[[26]](#footnote-26)^

subgenus ***BOMBUS*** Latreille, 1802^[[27]](#footnote-27)^

*TERRESTRIBOMBUS* Vogt, 1911

***cryptarum*** (Fabricius, 1775, *Apis*) S I added by Bertsch *et al.* (2005)

***lucorum*** (Linnaeus, 1761, *Apis*) E S W I M

***magnus*** Vogt, 1911 S I

***terrestris*** (Linnaeus, 1758, *Apis*) E S W I M

*audax* (Harris, 1776, *Apis*)

subgenus ***CULLUMANOBOMBUS*** Vogt, 1911

***cullumanus*** (Kirby, 1802, *Apis*) E^[[28]](#footnote-28)^

subgenus ***KALLOBOMBUS*** Dalla Torre, 1880

***soroeensis*** (Fabricius, 1777, *Apis*) E S W

subgenus ***MEGABOMBUS*** Dalla Torre, 1880

*HORTOBOMBUS* Vogt, 1911

***hortorum*** (Linnaeus, 1761, *Apis*) E S W I M

?*flavonigrescens* Smith, 1846

*ivernicus* Sladen, 1912^[[29]](#footnote-29)^

*splendida* Stelfox, 1938

***ruderatus*** (Fabricius, 1775, *Apis*) E

*perniger* (Harris, 1776, *Apis*)^[[30]](#footnote-30)^

*harrisellus* (Kirby, 1802, *Apis*)

*tunstallanus* (Kirby, 1802, *Apis*)

subgenus ***MELANOBOMBUS*** Dalla Torre, 1880

*LAPIDARIOBOMBUS* Vogt, 1911

***lapidarius*** (Linnaeus, 1758, *Apis*) E S W I M

subgenus ***PSITHYRUS*** Lepeletier, 1832

*ALLOPSITHYRUS* Popov, 1931

*ASHTONIPSITHYRUS* Frison, 1927

*FERNALDAEPSITHYRUS* Frison, 1927

*METAPSITHYRUS* Popov, 1931

***barbutellus*** (Kirby, 1802, *Apis*) E S W I

***bohemicus*** (Seidl, 1837, *Apis*) E S W I M

*distinctus* (Pérez, 1884, *Psithyrus*)

***campestris*** (Panzer, 1801, *Apis*) E S W I M

*rossiellus* (Kirby, 1802, *Apis*)

*leeanus* (Kirby, 1802, *Apis*)

*francisanus* (Kirby, 1802, *Apis*)

*swynnertoni* (Richards, 1936, *Psithyrus*)^[[31]](#footnote-31)^

***rupestris*** (Fabricius, 1793, *Apis*) E W I

*albinellus* (Kirby, 1802, *Apis*)

***sylvestris*** (Lepeletier, 1832, *Psithyrus*) E S W I

*quadricolor* misident.

***vestalis*** (Geoffroy, 1785, *Apis*) E S W I

subgenus ***PYROBOMBUS*** Dalla Torre, 1880

*PRATOBOMBUS* Vogt, 1911

***hypnorum*** (Linnaeus, 1758, *Apis*) E S W added by Goulson & Williams (2001)

***jonellus*** (Kirby, 1802, *Apis*) E S W I M

*nivalis* misident.

*scrimshiranus* (Kirby, 1802, *Apis*)

*atrocorbiculosus* Vogt, 1911

*hebridensis* Wild, 1931^[[32]](#footnote-32)^

*vogtii* Richards, 1933 preocc.

*monapiae* Kruseman, 1953^[[33]](#footnote-33)^

*vogtianus* Rasmont, 1983^[[34]](#footnote-34)^

***monticola*** Smith, 1849 E S W I M

*lapponicus* misident.

*scoticus* Pittioni, 1942

***pratorum*** (Linnaeus, 1761, *Apis*) E S W I M

*subinterruptus* (Kirby, 1802, *Apis*)

*donovanella* (Kirby, 1802, *Apis*)

*burrellana* (Kirby, 1802, *Apis*)

subgenus ***SUBTERRANEOBOMBUS*** Vogt, 1911

***distinguendus*** Morawitz, 1869 E S W I M

***subterraneus*** (Linnaeus, 1758, *Apis*) E W^[[35]](#footnote-35)^

*collinus* Smith, 1844

*latreillellus* (Kirby, 1802, *Apis*)^[[36]](#footnote-36)^

subgenus ***THORACOBOMBUS*** Dalla Torre, 1880

*RHODOBOMBUS* Dalla Torre, 1880

*POMOBOMBUS* Krüger, 1917

***humilis*** Illiger, 1806 E S W M

*solstitialis* Panzer, 1806

*helferanus* Seidl, 1837

*venustus* Smith, 1876 preocc.

*anglicus* Yarrow, 1978

***muscorum*** (Linnaeus, 1758, *Apis*) E S W I M

*arcticus* misident.

*smithianus* misident.

*pallidus* Evans, 1901 preocc.

*laevis* Vogt, 1909

*sladeni* Vogt, 1911^[[37]](#footnote-37)^

*allenellus* Stelfox, 1933^[[38]](#footnote-38)^

*orcadensis* Richards, 1935^[[39]](#footnote-39)^

*scyllonius* Richards, 1935^[[40]](#footnote-40)^

*celticus* Yarrow, 1978^[[41]](#footnote-41)^

*agricolae* Baker, 1996^[[42]](#footnote-42)^

***pascuorum*** (Scopoli, 1763, *Apis*) E S W I M

*vulgo* (Harris, 1776, *Apis*)^[[43]](#footnote-43)^

*agrorum* (Fabricius, 1787, *Apis*) preocc.

*floralis* (Gmelin, 1790, *Apis*)^[[44]](#footnote-44)^

*francillonellus* (Kirby, 1802, *Apis*)

*sowerbianus* (Kirby, 1802, *Apis*)

*beckwithella* (Kirby, 1802, *Apis*)

*curtisellus* (Kirby, 1802, *Apis*)

*forsterellus* (Kirby, 1802, *Apis*)

*cognatus* Stevens, 1846

*smithianus* White, 1851

*septentrionalis* Vogt, 1909^[[45]](#footnote-45)^

?***pomorum*** (Panzer, 1805, *Bremus*) E^[[46]](#footnote-46)^

***ruderarius*** (Müller, 1776, *Apis*) E S W I

*derhamellus* (Kirby, 1802, *Apis*)

*raiellus* (Kirby, 1802, *Apis*)

***sylvarum*** (Linnaeus, 1761, *Apis*) E S W I

*nigrescens* Pérez, 1879

*distinctus* Vogt, 1909

Tribe EUCERINI Latreille, 1802

***EUCERA*** Scopoli, 1770

subgenus ***EUCERA*** Scopoli, 1770

***longicornis*** (Linnaeus, 1758, *Apis*) E W

*linguaria* (Fabricius, 1775, *Apis*)

***nigrescens*** Pérez, 1879 E^[[47]](#footnote-47)^

*tuberculata* misident. [?]

Tribe MELECTINI Westwood, 1840

***MELECTA*** Latreille, 1802

***albifrons*** (Forster, 1771, *Apis*) E W

*punctata* (Fabricius, 1775, *Apis*)

*armata* (Panzer, 1799, *Andrena*) preocc.

***luctuosa*** (Scopoli, 1770, *Apis*) E^[[48]](#footnote-48)^

Subfamily NOMADINAE Latreille, 1802

Tribe EPEOLINI Roberston, 1903

***EPEOLUS*** Latreille, 1802

***cruciger*** (Panzer, 1799, *Nomada*) E W

*rufipes* Thomson, 1870

***variegatus*** (Linnaeus, 1758, *Apis*) E W S M

*notatus* misident.

*productus* Thomson, 1870

Tribe NOMADINI Latreille, 1802

***NOMADA*** Scopoli, 1763

***argentata*** Herrich-Schäffer, 1839 E I

*atrata* Smith, 1846

***armata*** Herrich-Schäffer, 1839 E W

*kirbyella* Stephens, 1846

***baccata*** Smith, 1844 E

*alboguttata* misident.

***conjungens*** Herrich-Schäffer, 1839 E

***errans*** Lepeletier, 1841 E

***fabriciana*** (Linnaeus, 1767, *Apis*) E S W I M

*fabriciella* (Kirby, 1802, *Apis*)

*quadrinotata* (Kirby, 1802, *Apis*)

***ferruginata*** (Linnaeus, 1767, *Apis*) E S W

*lateralis* misident.

*xanthosticta* (Kirby, 1802, *Apis*)

*bridgmaniana* Smith, 1876

***flava*** Panzer, 1798 E W I

***flavoguttata*** (Kirby, 1802, *Apis*) E S W I M

*rufocincta* (Kirby, 1802, *Apis*)

***flavopicta*** (Kirby, 1802, *Apis*) E W

*jacobaeae* misident. [?]

***fucata*** Panzer, 1798 E W

***fulvicornis*** Fabricius, 1793 E W

*lineola* Panzer, 1798

*sexcincta* (Kirby, 1802, *Apis*)

*capreae* (Kirby, 1802, *Apis*)

*cornigera* (Kirby, 1802, *Apis*)

***goodeniana*** (Kirby, 1802, *Apis*) E S W I M

*succincta* misident.

*alternata* (Kirby, 1802, *Apis*)

***guttulata*** Schenck, 1861 E

***hirtipes*** Pérez, 1884 E W

*bucephalae* Perkins, 1917

***integra*** Brullé, 1832 E W

*ferruginata* misident.

*germanica* misident.

*pleurosticta* misident.

*stigma* misident.

*cinctiventris* Friese, 1921

***lathburiana*** (Kirby, 1802, *Apis*) E W

*rufiventris* (Kirby, 1802, *Apis*)

***leucophthalma*** (Kirby, 1802, *Apis*) E S W I M

*borealis* Zetterstedt, 1838

*inquilina* Smith, 1844

***marshamella*** (Kirby, 1802, *Apis*) E S W I M

*alternata* misident.

***obtusifrons*** Nylander, 1848 E S W I M

*mistura* Smith, 1851

***panzeri*** Lepeletier, 1841 E S W I

*ruficornis* misident.

***roberjeotiana*** Panzer, 1799 E S W M

*tormentillae* Alfken, 1901

***ruficornis*** (Linnaeus, 1758, *Apis*) E S W I M

*bifida* Thomson, 1872

***rufipes*** Fabricius, 1793 E S W I M

*solidaginis* misident.

*picta* (Kirby, 1802, *Apis*)

*rufopicta* (Kirby, 1802, *Apis*)

***sexfasciata*** Panzer, 1799 E

*connexa* (Kirby, 1802, *Apis*)

*schaefferella* (Kirby, 1802, *Apis*)

***sheppardana*** (Kirby, 1802, *Apis*) E W

*furva* misident.

*dalii* Curtis, 1832

***signata*** Jurine, 1807 E W

***striata*** Fabricius, 1793 E S W I

*hillana* (Kirby, 1802, *Apis*)

*ochrostoma* (Kirby, 1802, *Apis*)

*vidua* Smith, 1844

***subcornuta*** (Kirby, 1802, *Apis*) E

Subfamily XYLOCOPINAE Latreille, 1802

Tribe Ceratinini Latreille, 1802

***CERATINA*** Latreille, 1802

subgenus ***EUCERATINA*** Hirashima, Moure & Daly, 1971

***cyanea*** (Kirby, 1802, *Apis*) E

Tribe XYLOCOPINI Latreille, 1802

***XYLOCOPA*** Latreille, 1809

subgenus ***XYLOCOPA*** Latreille, 1809

***violacea*** (Linnaeus, 1758, *Apis*) E

Family **Colletidae** Lepeletier, 1841

Subfamily Colletinae Lepeletier, 1841

***COLLETES*** Latreille, 1802

***cunicularius*** (Linnaeus, 1761, *Apis*)^[[49]](#footnote-49)^ E W

***daviesanus*** Smith, 1846 E S W I

***floralis*** Eversmann, 1852 E S I

*montanus* Morawitz, 1876

***fodiens*** (Geoffroy, 1785, *Apis*) E S W

***halophilus*** Verhoeff, 1944 E

***hederae*** Schmidt & Westrich, 1993 E added by Cross (2002)

***marginatus*** Smith, 1846 E W

***similis*** Schenck, 1853 E W I M

*picistigma* Thomson, 1872

***succinctus*** (Linnaeus, 1758, *Apis*) E S W I M

Subfamily HYLAEINAE Viereck, 1916

***HYLAEUS*** Fabricius, 1793

subgenus ***ABRUPTA*** Popov, 1939

***cornutus*** Curtis, 1831 E

*plantarius* Smith, 1842

subgenus ***HYLAEUS*** Fabricius, 1793

***communis*** Nylander, 1852 E S W I

*rupestris* (Smith, 1872, *Prosopis*)

subgenus ***KOPTOGASTER*** Alfken, 1912

***punctulatissimus*** Smith, 1842 E^[[50]](#footnote-50)^

subgenus ***LAMDOPSIS*** Popov, 1939

***annularis*** (Kirby, 1802, *Mellita*)^[[51]](#footnote-51)^ E

*euryscapus* misident.

*spilotus* Forster, 1871

*masoni* (Saunders, 1894, *Prosopis*)

***dilatatus*** (Kirby, 1802, *Mellita*) E W

*annularis* misident.

subgenus ***PARAPROSOPIS*** Popov, 1939

***pictipes*** Nylander, 1852 E

*varipes* (Smith, 1853, *Prosopis*)

subgenus ***PROSOPIS*** Fabricius, 1804

***brevicornis*** Nylander, 1852 E S W I M

*rubicola* (Smith, 1869, *Prosopis*) preocc.

***confusus*** Nylander, 1852 E S W I

***incongruus*** Förster, 1871 E W

*gibbus* misident.

*genalis* Thomson, 1872

***pectoralis*** Förster, 1871 E

*kriechbaumeri* Förster, 1871

*palustris* (Perkins, 1900, *Prosopis*)

***signatus*** (Panzer, 1798, *Sphex*) E W

subgenus ***SPATULARIELLA*** Popov, 1939

***hyalinatus*** Smith, 1842 E S W I M

Family **Halictidae** Thomson, 1869

Subfamily Halictinae Thomson, 1869

***HALICTUS*** Latreille, 1804

subgenus ***HALICTUS*** Latreille, 1804

***eurygnathus*** Blüthgen, 1931 E

*quadricinctus* misident.

*tetrazonius* misident.

***maculatus*** Smith, 1848 E^[[52]](#footnote-52)^

***rubicundus*** (Christ, 1791, *Apis*) E S W I M

*quadrifasciatus* Smith, 1870

*nesiotis* Perkins, 1922

subgenus ***SELADONIA*** Robertson, 1918

***confusus*** Smith, 1853^[[53]](#footnote-53)^ E

*alpinus* Alfken, 1907

*flavipes* misident.

***subauratus*** (Rossi, 1792, *Apis*)^[[54]](#footnote-54)^

*gramineus* Smith, 1849

***tumulorum*** (Linnaeus, 1758, *Apis*) E S W I

***LASIOGLOSSUM*** Curtis, 1833^[[55]](#footnote-55)^

subgenus ***DIALICTUS*** Robertson, 1902

*CHLORALICTUS* Robertson, 1902

***cupromicans*** (Pérez, 1903, *Halictus*)^[[56]](#footnote-56)^ E S W I M

***leucopus*** (Kirby, 1802, *Melitta*) E S W I M

*aeratum* (Kirby, 1802, *Melitta*)

*semiaeneum* (Brullé, 1832, *Halictus*)

*viridaeneum* (Blüthgen, 1918, *Halictus*)

***morio*** (Fabricius, 1793, *Hylaeus*) E S W M

***smeathmanellum*** (Kirby, 1802, *Melitta*) E S W M

subgenus ***HEMIHALICTUS*** Cockerell, 1897

***angusticeps*** (Perkins, 1895, *Halictus*) E

***brevicorne*** (Schenck, 1869, *Halictus*) E

***minutissimum*** (Kirby, 1802, *Melitta*) E W I

*arnoldi* (Saunders, 1910, *Halictus*)

***nitidiusculum*** (Kirby, 1802, *Melitta*) E S W I M

***parvulum*** (Schenck, 1853, *Hylaeus*) E W

*minutum* misident.

***pauperatum*** (Brullé, 1832, *Halictus*) E

*breviceps* (Saunders, 1879, *Halictus*)

***punctatissimum*** (Schenck, 1853, *Hylaeus*) E S W I M

*longiceps* (Saunders, 1879, *Halictus*)

***puncticolle*** (Morawitz, 1872, *Halictus*) E

***rufitarse*** (Zetterstedt, 1838, *Halictus*) E S W I

*atricorne* (Smith, 1870, *Halictus*)

***semilucens*** (Alfken, 1914, *Halictus*) E

***sexstrigatum*** (Schenck, 1870, *Halictus*) E added by Hawkins (2011)

*sabulosum* (Warncke, 1986, *Halictus*)

***villosulum*** (Kirby, 1802, *Melitta*) E S W I M

*punctulatum* (Kirby, 1802, *Melitta*)

subgenus ***LASIOGLOSSUM*** Curtis, 1833

***laevigatum*** (Kirby, 1802, *Melitta*) E W

*lugubris* (Kirby, 1802, *Melitta*)

***lativentre*** (Schenck, 1853, *Hylaeus*) E W

*decipiens* (Perkins, 1913, *Halictus*)

***prasinum*** (Smith, 1848, *Halictus*) E W

***quadrinotatum*** (Kirby, 1802, *Melitta*) E

***sexnotatum*** (Kirby, 1802, *Melitta*) E

*nitidum* misident.

***xanthopus*** (Kirby, 1802, *Melitta*) E W

*tricingulum* Curtis, 1833

subgenus ***LEUCHALICTUS*** Warncke, 1975

***leucozonium*** (Schrank, 1781, *Apis*) E S W I

*similis* (Smith, 1853, *Halictus*)

***zonulum*** (Smith, 1848, *Halictus*) E W

subgenus ***SPHECODOGASTRA*** Ashmead, 1899

***albipes*** (Fabricius, 1781, *Apis*) E S W I M

***calceatum*** (Scopoli, 1763, *Apis*) E S W I M

*cylindricum* (Fabricius, 1793, *Hylaeus*)

*fulvocinctum* (Kirby, 1802, *Melitta*)

*obovatum* (Kirby, 1802, *Melitta*)

***fratellum*** (Pérez, 1903, *Halictus*) E S W I

*nigrum* misident.

*subfasciatum* (Nylander, 1848, *Halictus*)

*freygessneri* (Alfken, 1905, *Halictus*)

***fulvicorne*** (Kirby, 1802, *Melitta*) E S W

*subfasciatum* misident.

***laeve*** (Kirby, 1802, *Melitta*) E^[[57]](#footnote-57)^

***laticeps*** (Schenck, 1869, *Halictus*) E

*semipunctulatum* misident.

***malachurum*** (Kirby, 1802, *Melitta*) E

*longulum* (Smith, 1848, *Halictus*)

***pauxillum*** (Schenck, 1853, *Hylaeus*) E W

*immarginatum* (Schenck, 1853, *Hylaeus*)

***SPHECODES*** Latreille, 1804

***crassus*** Thomson, 1870 E S W

*variegatus* von Hagens, 1874

***ephippius*** (Linnaeus, 1767, *Sphex*) E W M

*divisus* (Kirby, 1802, *Melitta*)

*similis* Wesmael, 1835

***ferruginatus*** von Hagens, 1882 E W

***geoffrellus*** (Kirby, 1802, *Melitta*) E S W I M

*affinis* von Hagens, 1882

*fasciatus* von Hagens, 1882

***gibbus*** (Linnaeus, 1758, *Sphex*) E S W

*picea* (Kirby, 1802, *Melitta*)

*sphecoides* (Kirby, 1802, *Melitta*)

***hyalinatus*** von Hagens, 1882 E S W I

***longulus*** von Hagens, 1882 E W

***miniatus*** von Hagens, 1882 E

*dimidiatus* von Hagens, 1882

***monilicornis*** (Kirby, 1802, *Melitta*) E S W I M

*subquadratus* Smith, 1845

***niger*** von Hagens, 1874 E

***pellucidus*** Smith, 1845 E S W I

*pilifrons* Thomson, 1870

***puncticeps*** Thomson, 1870 E W

***reticulatus*** Thomson, 1870 E W

***rubicundus*** von Hagens, 1875 E W

*ruficrus* misident.

*rufiventris* misident.

***scabricollis*** Wesmael, 1835 E W

***spinulosus*** von Hagens, 1875 E W

Subfamily ROPHITINAE Schenck, 1866

***DUFOUREA*** Lepeletier, 1841

***halictula*** (Nylander, 1852, *Rhophites*) E^[[58]](#footnote-58)^

***minuta*** Lepeletier, 1841 E^[[59]](#footnote-59)^

*vulgaris* Schenck, 1861

***ROPHITES*** Spinola, 1808

***quinquespinosus*** Spinola, 1808 E^[[60]](#footnote-60)^

Family **Megachilidae** Latreille, 1802

Subfamily Megachilinae Latreille, 1802

Tribe ANTHIDIINI Ashmead, 1899

***ANTHIDIUM*** Fabricius, 1804

***manicatum*** (Linnaeus, 1758, *Apis*)^[[61]](#footnote-61)^ E S W

***STELIS*** Panzer, 1806

***breviuscula*** Nylander, 1848 E added by Else & Spooner (1987)

***ornatula*** (Klug, 1807, *Gyrodroma*) E W

*octomaculata* Smith, 1843

***phaeoptera*** (Kirby, 1802, *Apis*) E W

***punctulatissima*** (Kirby, 1802, *Apis*) E S W

*aterrima* (Panzer, 1798, *Apis*) preocc.

Tribe MegachilinI Latreille, 1802

***COELIOXYS*** Latreille, 1809

subgenus ***ALLOCOELIOXYS*** Tkalcu, 1974

***afra*** Lepeletier, 1841^[[62]](#footnote-62)^

subgenus ***BOREOCOELIOXYS*** Mitchell, 1973

***inermis*** (Kirby, 1802, *Apis*) E W I

*acuminata* Nylander, 1852 preocc.

***mandibularis*** Nylander, 1848 E W

***rufescens*** Lepeletier & Serville, 1825 E W

*umbrina* Smith, 1843

subgenus ***COELIOXYS*** Latreille, 1809

***conoidea*** (Illiger, 1806, *Anthophora*) E W I

*vectis* Curtis, 1831

***elongata*** Lepeletier, 1841 E S W I

*sponsa* Smith, 1855

***quadridentata*** (Linnaeus, 1758, *Apis*) E W

***MEGACHILE*** Latreille, 1802

subgenus ***EUTRICHARAEA*** Thomson, 1872

***leachella*** Curtis, 1828 E W

*dorsalis* Pérez, 1879 Gusenleitner & Schwarz (2012*b*)

*argentata* misident.

subgenus ***MEGACHILE*** Latreille, 1802

***centuncularis*** (Linnaeus, 1758, *Apis*) E S W I M

***lapponica*** Thomson, 1872 E^[[63]](#footnote-63)^

***ligniseca*** (Kirby, 1802, *Apis*) E W I

***versicolor*** Smith, 1844^[[64]](#footnote-64)^ E S W I M

subgenus ***PSEUDOMEGACHILE*** Friese, 1899

***ericetorum*** (Lepeletier, 1841, *Megachile*) E^[[65]](#footnote-65)^

*fasciata* (Smith, 1844, *Megachile*)

*rufitarsis* (Smith, 1844, *Megachile*)

subgenus ***XANTHOSARUS*** Robertson, 1903

***circumcincta*** (Kirby, 1802, *Apis*) E S W

***maritima*** (Kirby, 1802, *Apis*) E W I M

***willughbiella*** (Kirby, 1802, *Apis*)^[[66]](#footnote-66)^ E SW I M

Tribe OSMIINI Newman, 1834

***CHELOSTOMA*** Latreille, 1809

***campanularum*** (Kirby, 1802, *Apis*) E

***florisomne*** (Linnaeus, 1758, *Apis*) E W

*maxillosum* (Linnaeus, 1767, *Apis*)

***HERIADES*** Spinola, 1808

***rubicola*** Peréz, 1890 E added by Else (in prep.)

***truncorum*** (Linnaeus, 1758, *Apis*) E

***HOPLITIS*** Klug, 1807

subgenus ***ALCIDAMEA***

***claviventris*** (Thomson, 1872, *Osmia*) E W

*leucomelana* misident.

***leucomelana*** (Kirby, 1802, *Apis*) E^[[67]](#footnote-67)^

*parvula* (Dufour & Perris, 1840, *Osmia*)

subgenus ***ANTHOCOPA*** Lepeletier & Serville, 1825

***spinulosa*** (Kirby, 1802, *Apis*) E W

***OSMIA*** Panzer, 1806

subgenus ***HELICOSMIA*** Thomson, 1872

***aurulenta*** Panzer, 1799 E S W I

***caerulescens*** (Linnaeus, 1758, *Apis*) E S W

*aenea* (Linnaeus, 1761, *Apis*)

***niveata*** (Fabricius, 1804, *Apis*) E

*fulviventris* (Panzer, 1798, *Apis*)

***leaiana*** (Kirby, 1802, *Apis*) E W

*fulviventris* misident.

subgenus ***MELANOSMIA*** Schmiedeknecht, 1884

***inermis*** (Zetterstedt, 1838, *Anthophora*) S

*parietina* misident.

***parietina*** Curtis, 1828 E S W

***pilicornis*** Smith, 1846 E W

***uncinata*** Gerstäcker, 1869 S added by Else in Else & Spooner (1987)

***xanthomelana*** (Kirby, 1802, *Apis*) E W

*atricapilla* Curtis, 1828

subgenus ***NEOSMIA*** Tkalc , 1974

***bicolor*** (Schrank, 1781, *Apis*) E W

subgenus ***OSMIA*** Panzer, 1806

***bicornis*** (Linnaeus, 1758, *Apis*) E S W I

*rufa* (Linnaeus, 1758, *Apis*) E S W

*hedera* Smith, 1844

Family **Melittidae** Schenck, 1860

Subfamily DASYPODAINAE Börner, 1919^[[68]](#footnote-68)^

***DASYPODA*** Latreille, 1802

***hirtipes*** (Fabricius, 1793, *Andrena*) E W

?*altercator* (Harris, 1776, *Apis*) nom. dub.

*plumipes* (Panzer, 1797, *Andrena*)

*swammerdamella* (Kirby, 1802, *Melitta*)

Subfamily MACROPIDINAE Robertson, 1904

***MACROPIS*** Panzer, 1809

***europaea*** Warncke, 1973 E

*labiata* misident.

Subfamily Melittinae Schenck, 1860

***MELITTA*** Kirby, 1802

*PSEUDOCILISSA* Radoszkowski, 1891

***dimidiata*** Morawitz, 1876 E

***haemorrhoidalis*** (Fabricius, 1775, *Andrena*) E S W M

*chrysura* Kirby, 1802

***leporina*** (Panzer, 1799, *Apis*) E W

***tricincta*** Kirby, 1802 E W

*melanura* (Nylander, 1852, *Kirbya*)

Superfamily **CHRYSIDOIDEA^[[69]](#footnote-69)^**

Family **Bethylidae** Haliday, 1833^[[70]](#footnote-70)^

Subfamily Bethylinae Haliday, 1833

***BETHYLUS*** Latreille, 1802

*PERISEMUS* Förster, 1856

*ANOXUS* Thomson, 1862 Polaszek & Krombein (1994)

*EPISEMUS* Thomson, 1862

*DIGONIOZUS* Kieffer, 1905

***boops*** (Thomson, 1862, *Anoxus*) E added by Burn (1997)

***cephalotes*** (Förster, 1860, *Perisemus*) E S W M

*fuscicornis* var. *tibialis* Kieffer, 1905

***dendrophilus*** Richards, 1939 E

***fuscicornis*** (Jurine, 1807, *Omalus*) E S W

*sygenesiae* Haliday, 1834

*fulvicornis* Curtis, 1838

*triareolatus* Förster, 1851

*variabilis* (Thomson, 1862, *Episemus*)

*hyalinus* (Marshall, 1874, *Perisemus*) de Rond (1994)

*fuscicornis* var. *maurus* Kieffer, 1905

*brevipennis* Hellén, 1920

*berlandi* Arle, 1929

***GONIOZUS*** Förster, 1856

*PARASIEROLA* Cameron, 1883

*PROGONIOZUS* Kieffer, 1905

*PERISIEROLA* Kieffer, 1914

***claripennis*** (Förster, 1851, *Bethylus*) E

*fuscipennis* (Förster, 1851, *Bethylus*)

*distigmus* Thomson, 1862

*audouinii* Westwood, 1874

*claripennis* var. *fuscipennis* Kieffer, 1905 preocc.

*claripennis* var. *tibialis* Kieffer, 1905

Subfamily Epyrinae Kieffer, 1914

***CEPHALONOMIA*** Westwood, 1833

*HOLOPEDINA* Förster, 1850

*CEPHALOMIA* Kirchner, 1867

***formiciformis*** Westwood, 1833 E I

*polypori* (Förster, 1850, *Holopedina*)

*brevipennis* Kieffer, 1906^[[71]](#footnote-71)^

*formiciformis* var. *sulcata* Kieffer, 1906

# ***gallicola*** (Ashmead, 1887, *Sclerochroa*)

*nubilipennis* (Ashmead, 1887, *Holopedina*)

*xambeui* Girad, 1898

*quadridentata* Duchaussoy, 1920

*strandi* Hoffer, 1936

***hammi*** Richards, 1939 E

# ***tarsalis*** (Ashmead, 1893, *Ateleopterus*)

*carinata* Kieffer, 1907

*meridionalis* Brethes, 1913

*kiefferi* Fouts, 1920

# ***waterstoni*** Gahan, 1931

***EPYRIS*** Westwood, 1832

*DOLUS* Motshultsky, 1863

*MUELLERELLA* Saussure, 1892

*HOMOGLENUS* Kieffer, 1904 Terayama (2003)

*PAREPYRIS* Kieffer, 1913

*PSILEPYRIS* Kieffer, 1913

*ARTIEPYRIS* Kieffer, 1913

***bilineatus*** Thomson, 1862 E

*fraternus* Westwood, 1874

*saeva* Westwood, 1874

*multidentatus* Keiffer, 1906

*multidentatus* var. *angustipennis* Kieffer, 1906

***niger*** Westwood, 1832 E

[?***tricolor*** Cameron, 1888^[[72]](#footnote-72)^]

***HOLEPYRIS*** Kieffer, 1905

*MISEPYRIS* Kieffer, 1913

*PAREPYRIS* Brethes, 1913

# ***glabratus*** (Fabricius, 1798, *Tiphia*)

*hawaiiensis* (Ashmead, 1901, *Holepyris*)

# ***sylvanidis*** (Brethes, 1913, *Parepyris*)

*zeae* (Turner & Waterston, 1921, *Rhabdepyris*)

***LAELIUS*** Ashmead, 1893^[[73]](#footnote-73)^

***femoralis*** (Förster, 1860, *Bethylus*) E

*microneurus* Kieffer, 1906 Notton *et al.* (2014)

*nigricrus* Kieffer, 1906

# ***pedatus*** (Say, 1836, *Bethylus*) E added by Notton *et al.* (2014)

***PLASTANOXUS*** Kieffer, 1905

*SNAPPANIA* Hedqvist, 1975

***chittendenii*** (Ashmead, 1893, *Anoxus*) E

# ***munroi*** Richards, 1939

# ***westwoodi*** (Kieffer, 1914, *Cephalonomia*)

*kiefferi* Gahan, 1931

Subfamily Pristocerinae Kieffer, 1914

***PRISTOCERA*** Klug, 1808

*ACREPYRIS* Kieffer, 1905

*MANGESIA* Kieffer, 1911

*TRICHELOBRACHIUM* Kieffer, 1914

***depressa*** (Fabricius, 1805, *Bethylus*) E

*roubali* (Menozzi, 1925, *Pseudisobrachium*)

***PSEUDISOBRACHIUM*** Kieffer, 1904

*MONEPYRIS* Kieffer, 1905

*XESTOBETHYLUS* Cameron, 1909

*PLUTOBETHYLUS* Kieffer, 1910

*LYSSEPYRIS* Kieffer, 1913

*XANTEPYRIS* Kieffer, 1913

*PARISOBRACHIUM* Kieffer, 1914

*AFRISOBRACHIUM* Benoit, 1957

*EDAPHOLIGON* Oglobin, 1963

***subcyaneum*** (Haliday, 1838, *Epyris*) E

*halidaii* (Westwood, 1874, *Epyris*)

*carpentieri* Kieffer, 1906

*carpentieri* var. *septemfasciatum* Kieffer, 1906

*cantianum* Chitty, 1906

*concolor* Kieffer, 1906

Family **Chrysididae** Latreille, 1802

Subfamily Cleptinae Morice, 1900

***CLEPTES*** Latreille, 1802

subgenus ***CLEPTES*** Latreille, 1802

***semiauratus*** (Linnaeus, 1761, *Sphex*)^[[74]](#footnote-74)^ E W

*auratus* (Panzer, 1798, *Ichneumon*)

*pallipes* Lepeletier, 1805

*diana* Mocsáry, 1889

subgenus ***LEIOCLEPTES*** Móczár, 1962

***nitidulus*** (Fabricius, 1793, *Ichneumon*) E

Subfamily Chrysidinae Latreille, 1802^[[75]](#footnote-75)^

Tribe CHRYSIDINI Latreille, 1802

***CHRYSIS*** Linnaeus, 1761^[[76]](#footnote-76)^

***angustula*** Schenck, 1856 E S W

*brevidens* Tournier, 1879

***corusca*** Valkeila, 1971 E added by Soon *et al.* (2014)

***fulgida*** Linnaeus, 1761 E

***gracillima*** Förster, 1853 E added by Morgan (1984)

*saussurei* Chevrier, 1862

***ignita*** (Linnaeus, 1758, *Sphex*) E S W I M

***illigeri*** Wesmael, 1839 E

*chrysoprasina* Hellén, 1919 preocc.

*helleni* Linsenmaier, 1959

*succincta* misident.

***impressa*** Schenck, 1856 E S W I M

***longula*** Abeille de Perrin, 1879 E S

***mediata*** Linsenmaier, 1951 E W I

***pseudobrevitarsis*** Linsenmaier, 1951 E

***ruddii*** Shuckard, 1836 E S W M

*auripes* Wesmael, 1839

***schencki*** Linsenmaier, 1968 E

*schenckiana* Linsenmaier, 1959 preocc.

***terminata*** Dahlbom, 1854 E added by Soon *et al.* (2014)

***vanlithi*** Linsenmaier, 1959 E S W I M

*rutiliventris* misident.

***viridula*** Linnaeus, 1761 E W

*bidentata* Linnaeus, 1767

***CHRYSURA*** Dahlbom, 1845

***hirsuta*** (Gerstäcker, 1869, *Chrysis*) S

*osmiae* (Thomson, 1870, *Chrysis*)

***radians*** (Harris, 1776, *Chrysis*) E W

*pustulosa* (Abeille de Perrin, 1878, *Chrysis*)

***PSEUDOSPINOLIA*** Linsenmaier, 1951

***neglecta*** (Shuckard, 1836, *Chrysis*) E W

***TRICHRYSIS*** Lichtenstein, 1876

***cyanea*** (Linnaeus, 1758, *Sphex*) E S W

Tribe ElampinI Dahlbom, 1854

***ELAMPUS*** Spinola, 1806

*NOTOZUS* Förster, 1853

***panzeri*** (Fabricius, 1804, *Chrysis*) E

*scutellaris* (Panzer, 1798, *Chrysis*) preocc.

*constrictus* misident.

***HEDYCHRIDIUM*** Abeille de Perrin, 1878

***ardens*** (Latreille, 1801, *Chrysis*) E S W I M

***coriaceum*** (Dahlbom, 1854, *Hedychrum*) E

***cupreum*** (Dahlbom, 1845, *Hedychrum*) E W

*integrum* (Dahlbom, 1854, *Hedychrum*) Morgan (1984)

***roseum*** (Rossi, 1790, *Chrysis*) E

***HEDYCHRUM*** Latreille, 1802

***niemelai*** Linsenmaier, 1959 E

*nobile* misident.

***nobile*** (Scopoli, 1763, *Sphex*) E added by Baldock & Hawkins (2013)^[[77]](#footnote-77)^

*lucidula* (Fabricius, 1775, *Chrysis*)

*regia* (Fabricius, 1793, *Chrysis*)

***rutilans*** Dahlbom, 1854 E

*intermedium* misident.

***OMALUS*** Panzer, 1801

***aeneus*** (Fabricius, 1787, *Chrysis*) E W

***puncticollis*** (Mocsáry, 1887, *Ellampus*)^[[78]](#footnote-78)^ E S W

***PHILOCTETES*** Abeille de Perrin, 1879

***truncatus*** (Dahlbom, 1831, *Chrysis*) E

***PSEUDOMALUS*** Ashmead, 1902

***auratus*** (Linnaeus, 1758, *Sphex*) E S W I

***violaceus*** (Scopoli, 1763, *Sphex*) E W

Family **Dryinidae** Haliday, 1833^[[79]](#footnote-79)^

Subfamily Anteoninae Perkins, 1912

***ANTEON*** Jurine, 1807

*CHELOGYNUS* Haliday, 1838

*NEOCHELOGYNUS* Perkins, 1905

***arcuatum*** Kieffer, 1905 E S W I

*imberbis* Kieffer, 1905

*bensoni* Richards, 1939

*jurineanum* in part, Perkins, 1976

***brachycerum*** (Dalman, 1823, *Dryinus*) E S W I

*lyde* (Walker, 1837, *Dryinus*)

*nigricornis* Kieffer, 1905

*triareolatus* Kieffer, 1905

*brevicollis* Kieffer, 1905

*flavitarsis* Kieffer, 1905

*indivisus* Kieffer, 1905

*nigroclavatus* Kieffer, 1905

*curvatus* Kieffer, 1906

*obscuricornis* Kieffer, 1906

*suffolciensis* (Chitty, 1908, *Antaeon*)

*curvus* Kieffer, 1914

***ephippiger*** (Dalman, 1818, *Gonatopus*) E S W I

*collaris* (Dalman, 1818, *Gonatopus*)

*facialis* (Thomson, 1860, *Dryinus*)

*albidicollis* Kieffer, 1905

*rubrifrons* Kieffer, 1905

*rufovariegatus* (Berland, 1928, *Chelogynus*)

*albidocolle* Richards, 1939

*pyonganensis* Moczar, 1983

***exiguum*** (Haupt, 1941, *Chelogynus*) E W added by Burn (1995)

*subarcticus* Hellén, 1935 nom. nud.

*flaviscapus* Jansson, 1950

*subarcticus* Hellén, 1953

***faciale*** (Thomson, 1860, *Dryinus*) E

*pseudohilare* Burn, 1990

***flavicorne*** (Dalman, 1818, *Gonatopus*) E S W I

*sericeus* Kieffer, 1905

*subflavicornis* Haupt, 1941

***fulviventre*** (Haliday, 1828, *Dryinus*) E S W I M

*fuscipes* (Thomson, 1860, *Dryinus*)

*similis* Kieffer, 1905

*gracilicollis* Kieffer, 1905

*flavinervis* Kieffer, 1905

*parvulus* Kieffer, 1905

*xanthostigma* Kieffer, 1905

*flaviscapus* Kieffer, 1905

*parvus* Kieffer, 1906

*alutaceus* (Richards, 1935, *Chelogynus*)

***gaullei*** Kieffer, 1905 E S W I

*cameroni* Kieffer, 1905

*trivialis* Kieffer, 1905

*rufulocollis* (Chitty, 1908, *Antaeon*)

***infectum*** (Haliday, 1837, *Dryinus*) E S I

*luteicornis* misident.^[[80]](#footnote-80)^

*inclytus* (Haliday, 1837, *Dryinus*)

*lateralis* (Thomson, 1860, *Dryinus*)

*fusiformis* Kieffer, 1905

*punctatus* Kieffer, 1905

*ellimani* (Chitty, 1908, *Antaeon*)

***jurineanum*** Latreille, 1809 E S W I

*brevicornis* (Dalman, 1818, *Gonatopus*)

*cursor* (Haliday, 1828, *Dryinus*)

*otiartes* (Walker, 1837, *Dryinus*)

*sisithrus* (Walker, 1837, *Dryinus*)

*nanus* (Haliday, 1837, *Dryinus*)

*crenulatus* Kieffer, 1905

*thomsoni* Kieffer, 1905

*vicinus* Kieffer, 1905

*marginatus* Kieffer, 1905

*rectus* Kieffer, 1905

*scoticus* Kieffer, 1905

*barbatus* (Chitty, 1908, *Antaeon*)

*brunneipes* (Berland, 1928, *Xenanteon*)

nec *jurineanum* Richards, 1939

nec *jurineanum* Perkins, 1976

***pubicorne*** (Dalman, 1818, *Gonatopus*)^[[81]](#footnote-81)^ E S W I

*tenuicornis* (Dalman, 1823, *Dryinus*)

*cephalotes* (Ljungh, 1824, *Gonatopus*)

*lucidus* (Haliday, 1828, *Dryinus*)

*penidas* (Walker, 1837, *Dryinus*)

*alorus* (Walker, 1837, *Dryinus*)

*fuscoclavatus* Kieffer, 1905

*triangularis* Kieffer, 1905

*divisus* Kieffer, 1905

*vulgaris* Kieffer, 1905

*breviventralis* (Chitty, 1908, *Antaeon*)

*delicatulus* (Chitty, 1908, *Antaeon*)

*serratus* (Maneval, 1935, *Chelogynus*)

*exiguus* (Haupt, 1941, *Chelogynus*)

*mongolicum* Moczar, 1983

***reticulatum*** Kieffer, 1905 E added by Olmi (1989)

***scapulare*** (Haliday, 1837, *Dryinus*) E

*longiforceps* Kieffer, 1905

*carinatus* Kieffer, 1905

*lanionis* (Haupt, 1941, *Chelogynus*)

***tripartitum*** Kieffer, 1905 E S I

*tricarinatus* Kieffer, 1905

*kiefferi* (Chitty, 1908, *Antaeon*)

*angusticollis* (Berland, 1928, *Chelogynus*)

*prehensor* (Maneval, 1935, *Chelogynus*)

*berlandi* (Richards, 1936, *Chelogynus*)

*silvaticus* (Ponomarenko, 1970, *Chelogynus*)

***LONCHODRYINUS*** Kieffer, 1905

*PRENANTEON* Kieffer, 1913

*PSILANTEON* Kieffer, 1913

***ruficornis*** (Dalman, 1818, *Gonatopus*) E S W I M

*basalis* (Dalman, 1818, *Gonatopus*)

*frontalis* (Dalman, 1818, *Gonatopus*)

*fuscicornis* (Dalman, 1818, *Gonatopus*)

*longicornis* (Dalman, 1823, *Dryinus*)

*crassimanus* (Haliday, 1828, *Dryinus*)

*daos* (Walker, 1837, *Dryinus*)

*ilus* (Walker, 1837, *Dryinus*)

*misor* (Walker, 1837, *Dryinus*)

*lapponicus* (Thomson, 1860, *Dryinus*)

*retusus* (Thomson, 1860, *Dryinus*)

*lepidus* (Förster, 1861, *Chelogynus*)

*subapterus* (Kieffer, 1905, *Anteon*)

*aequalis* (Kieffer, 1905, *Anteon*)

*melanocera* (Kieffer, 1905, *Anteon*)

*vitellinipes* (Kieffer, 1905, *Anteon*)

*procericornis* (Kieffer, 1905, *Anteon*)

*declivis* (Kieffer, 1905, *Anteon*)

*pallidinervis* (Kieffer, 1905, *Anteon*)

*integer* (Kieffer, 1905, *Anteon*)

*curvinervis* (Kieffer, 1905, *Anteon*)

*fractinervis* (Kieffer, 1905, *Anteon*)

*hyalinipennis* (Kieffer, 1905, *Anteon*)

*longifilis* (Kieffer, 1906, *Anteon*)

*halidayi* (Kieffer, 1906, *Anteon*)

*morleyi* (Chitty, 1908, *Antaeon*)

*luffnessensis* (Chitty, 1908, *Antaeon*)

*beaumonti* (Chitty, 1908, *Antaeon*)

*walkeri* (Kieffer, 1914, *Chelogynus*)

*parcepunctatus* (Kieffer, 1914, *Prenanteon*)

*palustris* (Oglobin, 1924, *Prenanteon*)

*filicornis* (Oglobin, 1924, *Prenanteon*)

*euscelisi* (Haupt, 1941, *Prenanteon*)

*semenovi* (Ponomarenko, 1970, *Prenanteon*)

*foveatus* (Richards, 1971, *Prenanteon*)

*pektusanense* (Moczar, 1983, *Prenanteon*)

*clavatum* (Moczar, 1983, *Prenanteon*)

Subfamily Aphelopinae Perkins, 1912

***APHELOPUS*** Dalman, 1823^[[82]](#footnote-82)^

*ANTAPHELOPUS* Benoit, 1951

*GYMNAPHELOPUS* Benoit, 1951

***atratus*** (Dalman, 1823, *Dryinus*) E S W I

*holomelas* Richards, 1939

***camus*** Richards, 1939 E W

*heidelbergensis* Richards, 1939

***melaleucus*** (Dalman, 1818, *Gonatopus*) E S W I

*albipes* (Ratzeburg, 1848, *Ceraphron*)

***nigriceps*** Kieffer, 1905 E S W I

***querceus*** Olmi, 1984 E added by Burn (1995)

***serratus*** Richards, 1939 E S W I

Subfamily Bocchinae Richards, 1939

***MYSTROPHORUS*** Förster, 1856

***formicaeformis*** Ruthe, 1859 E

Subfamily Dryininae Kieffer, 1906

***DRYINUS*** Latreille, 1804

*CAMPYLONYX* Westwood, 1835

*PARADRYINUS* Perkins, 1905

*CHLORODRYINUS* Perkins, 1905

*PLASTODRYINUS* Kieffer, 1906

*MESODRYINUS* Kieffer, 1906

*HESPERODRYINUS* Perkins, 1907

***collaris*** (Linnaeus, 1767, *Sphex*) E

*formicarius* Latreille, 1805

*ampuliciformis* (Westwood, 1835, *Campylonyx*)

*corsicae* (Kieffer, 1914, *Lestodryinus*)

***niger*** Kieffer, 1904 E

*brittanicus* (Richards, 1939, *Mesodryinus*)

Subfamily Gonatopodinae Kieffer, 1906

***GONATOPUS*** Ljungh, 1810

*DICONDYLUS* Haliday, 1829-30

*LABEO* Haliday, 1833

*PSEUDOGONATOPUS* Perkins, 1905

*NEOGONATOPUS* Perkins, 1905

*PACHYGONATOPUS* Perkins, 1905

*CHALCOGONATOPUS* Perkins, 1905

*EUGONATOPUS* Perkins, 1905

*PLATYGONATOPUS* Kieffer, 1906

*AGONATOPUS* Perkins, 1907

*AGONATOPOIDES* Perkins, 1907

*EUCAMPTONYX* Perkins, 1907

*CYRTOGONATOPUS* Kieffer, 1907

*DIGONATOPUS* Kieffer, 1913

*LABERIUS* Kieffer, 1914

*TRICHOGONATOPUS* Hellén, 1930

*METAGONATOPUS* Oglobin, 1932

*ALLOGONATOPUS* Haupt, 1938

*DONISTHORPINA* Richards, 1939

*PLECTROGONATOPUS* Richards, 1939

*TETRODONTOCHELYS* Richards, 1939

*EPIGONATOPOIDES* Richards, 1939

*RHYNCHOGONATOPUS* Benoit, 1953

*MADECAGONATOPUS* Benoit, 1953

*CYRTOGONATOPOIDES* Ponomarenko, 1966

*MEGAGONATOPUS* Olmi & Currado, 1976

*TETRADONTOCHELYS* Perkins, 1976

***albosignatus*** Kieffer, 1905 E W

*separatus* (Richards, 1939, *Pseudogonatopus*)

Burn & Olmi (2011)

***bicolor*** (Haliday, 1828, *Dryinus*) E S W I

*vitripennis* (Haliday, 1833, *Labeo*)

*excisus* (Westwood, 1833, *Antaeon*)

*conjunctus* Kieffer, 1905

*bifarius* Kieffer, 1906

*decretorius* Haupt, 1916

*lindbergi* (Heikinheimo, 1957, *Dicondylus*)

***clavipes*** (Thunberg, 1827, *Gelis*) E S W I

*sepsoides* Westwood, 1833

*pilosus* Thomson, 1860

*nigerrimus* (Förster, 1861, *Labeo*)

*pusillus* Szépligeti, 1901, *Labeo*)

*hispanicus* Kieffer, 1905

*sociabilis* Kieffer, 1907

*borealis* Sahlberg, 1910

*wagneri* Strand, 1919

*barbatellus* Richards, 1939

*campestris* Ponomarenko, 1965

*rhaensis* Ponomarenko, 1970

***distinctus*** Kieffer, 1905 E W

*septemdentatus* Sahlberg, 1910

*robustus* (Ceballos, 1927, *Dicondylus*)

***distinguendus*** Kieffer, 1905 E W I

*flavicornis* Thomson, 1860 preocc.

*luteicornis* Kieffer, 1905

*excavatus* Sahlberg, 1910

*liechtensteini* Picard, 1932

*procerus* (Haupt, 1938, *Allogonatopus*)

*thomsoni* Hellén, 1953

*rossicus* Ponomarenko, 1965

***formicicolus*** (Richards, 1939, *Donisthorpina*) E W

***helleni*** Raatikainen, 1961 E added by Burn (1997)

*dichromus* Kieffer, 1906

*rufescens* Hellén, 1935

***lunatus*** Klug, 1810 E W

*bifasciatus* Kieffer, 1904

*gracilicornis* Kieffer, 1904

*filicornis* Kieffer, 1905

*gracilis* Kieffer, 1905

*marshalli* Kieffer, 1905

*myrmecophilus* Kieffer, 1905

*gracilipes* Kieffer, 1906

*raptoripes* Strand, 1919

***pedestris*** Dalman, 1818 E

*ljunghii* Westwood, 1833

*leucostomus* Sahlberg, 1910

*arnoldii* (Ponomarenko, 1966, *Pachygonatopus*)

***striatus*** Kieffer, 1905 E S W I

*richardsi* (Moczar, 1965, *Plectrogonatopus*)

*tauricus* (Ponomarenko, 1965, *Agonatopoides*)

***HAPLOGONATOPUS*** Perkins, 1905

*MONOGONATOPUS* Richards, 1939

***oratorius*** (Westwood, 1833, *Gonatopus*) E

Family **Embolemidae** Förster, 1856

***EMBOLEMUS*** Westwood, 1833

*MYRMECOMORPHUS* Westwood, 1833

*POLYPLANUS* Nees, 1834

*EMBOLIMUS* Agassiz, 1846

*FORMILA* De Romand, 1846

*PEDINOMMA* Förster, 1856

*AMPULICOMORPHA* Ashmead, 1893

*AMPULICIMORPHA* Brues, 1933

***ruddii*** (Westwood, 1833, *Myrmecomorphus*) E S W

*rufescens* (Westwood, 1833, *Myrmecomorphus*)

*sickershusanus* (Nees, 1834, *Polyplanus*)

*antennalis* (Kieffer, 1906, *Pedinomma*) Hilpert (1989)

*holochlora* (Kieffer, 1906, *Pedinomma*)

*hypochlora* (Kieffer, 1906, *Pedinomma*)

*rufus* Kieffer, 1906

Superfamily **VESPOIDEA^[[83]](#footnote-83)^**

Family **Formicidae** Latreille, 1809^[[84]](#footnote-84)^

Subfamily Dolichoderinae Forel, 1878

[***LINEPITHEMA*** Mayr, 1866

# ***humile*** (Mayr, 1868, *Hypoclinea*)^[[85]](#footnote-85)^]

***TAPINOMA*** Förster, 1850

*MICROMYRMA* Dufour, 1857

*SEMONIUS* Forel, 1910

*TAPINOPTERA* Santschi, 1925

*ZATAPINOMA* Wheeler, 1928

*NEOCLYSTOPSENELLA* Kurian, 1955

***erraticum*** (Latreille, 1798, *Formica*) E

*caerulescens* (Losana, 1834, *Formica*)

*glabrella* (Nylander, 1849, *Formica*)

*collina* Förster, 1850

*bononiensis* Emery, 1925

*breve* Emery, 1925

*tauridis* Emery, 1925

*transcaucasica* Karavaiev, 1927

***subboreale*** Seifert, 2012 E

*ambiguum* misident.^[[86]](#footnote-86)^

*madeirense* misident.

# ***melanocephalum*** (Fabricius, 1793, *Formica*)

*nana* (Jerdon, 1851, *Formica*) preocc.

*pellucida* (Smith, 1857, *Myrmica*)

*familiaris* (Smith, 1860, *Formica*) preocc.

*australe* Santschi, 1928

*australis* Santschi, 1928

Subfamily Formicinae Latreille, 1802

***FORMICA*** Linnaeus, 1758

*FORMICINA* Schuckard, 1840

*NEOFORMICA* Wheeler, 1913

*RAPTIFORMICA* Forel, 1913

*SERVIFORMICA* Forel, 1913

*COPTOFORMICA* Müller, 1923

*ADFORMICA* Lomnicki, 1925

***aquilonia*** Yarrow, 1955 S I

*schmidti* Ruzsky, 1920 preocc.

***cunicularia*** Latreille, 1798 E W

*fuscorufibarbis* Forel, 1874

*glauca* Ruzsky, 1896

*rubescens* Forel, 1904

*caucasica* Wheeler, 1913

*volgensis* Ruzsky, 1914

*katuniensis* Ruzsky, 1915

*montivaga* Santschi, 1928

*montaniformis* Kuznetsov-Ugamsky, 1929

*fuscoides* Dlussky, 1967

***exsecta*** Nylander, 1846 E S

*exsectopressilabris* Forel, 1874

*rubens* Forel, 1874

*etrusca* Emery, 1909

*dalcqi* Bondroit, 1918

*sudetica* Scholz, 1924

*wheeleri* Creighton, 1935

*kontuniemii* Betrem, 1954

*nemoralis* Dlussky, 1964

***fusca*** Linnaeus, 1758 E S W I M

*libera* Scopoli, 1763

*flavipes* Geoffroy, 1785

*barbata* Razoumowski, 1789

*tristis* Christ, 1791

*glebaria* Nylander, 1846

*marcida* Wheeler, 1913

*pallipes* Kuznetsov-Ugamsky, 1926

*rufipes* Stitz, 1930

***lemani*** Bondroit, 1917 E S W I M

*borealis* Vashkevich, 1924

***lugubris*** Zetterstedt, 1838 E S W I

*congerens* Nylander, 1846

*santschii* Wheeler, 1913

*nylanderi* Bondroit, 1920

*unicolor* Ruzsky, 1926

*montana* Sadil, 1953

***picea*** Nylander, 1846^[[87]](#footnote-87)^ E W

*candida* misident.

*glabra* White, 1884 preocc.

*transkaucasica* Nasonov, 1889

*orientalis* Ruzsky, 1915

*piceoinplana* Emery, 1925

*inplana* Emery, 1925

*lochmatteri* Stärcke, 1935

***pratensis*** Retzius, 1783 E^[[88]](#footnote-88)^

*nigricans* Bondroit, 1912

*cordieri* Bondroit, 1917

*grouvellei* Bondroit, 1918

*ciliata* Ruzsky, 1926

*thyssei* Stärcke, 1942

*pratensoides* Gösswald, 1951

***rufa*** Linnaeus, 1761 E W

*ferruginea* Christ, 1791

*dorsata* Panzer, 1798

*major* Nylander, 1849

*piniphila* Schenck, 1852

*apicalis* Smith, 1858

*rufopratensis* Forel, 1874

*meridionalis* Nasonov, 1889

*gaullei* Bondroit, 1917

***rufibarbis*** Fabricius, 1793 E

*nicaeensis* Leach, 1825

*stenoptera* Förster, 1850

*cinereorufibarbis* Forel, 1874

*defensor* Smith, 1878

*fraterna* Smith, 1878

*piligera* Lomnicki, 1925

***sanguinea*** Latreille, 1798 E S W

*dominula* Nylander, 1846

*fusciceps* Emery, 1895

*mollesonae* Ruzsky, 1903

*clarior* Ruzsky, 1905

*flavorubra* Forel, 1909

*borea* Santschi, 1925

*strennua* Santschi, 1925

*griseopubescens* Kuznetsov-Ugamsky, 1926

*monticola* Kuznetsov-Ugamsky, 1926

*rotundata* Kuznetzsov-Ugamsky, 1926

*arenicola* Kuznetsov-Ugamsky, 1928

*leninei* Santschi, 1928

*tristis* Karavaiev, 1929

***LASIUS*** Fabricius, 1804

*CHTHONOLASIUS* Ruzsky, 1912

*DENDROLASIUS* Ruzsky, 1912

*CAUTOLASIUS* Wilson, 1955

*AUSTROLASIUS* Faber, 1967

***alienus*** (Foerster, 1850, *Formica*) E S W I

*americanus* Emery, 1893

*pannonica* Röszler, 1942

***brunneus*** (Latreille, 1798, *Formica*) E W

*pallida* (Latreille, 1798, *Formica*)

*timida* (Förster, 1850, *Formica*)

*alienobrunneus* Forel, 1874

*nigrobrunneus* (Donisthorpe, 1926, *Acanthomyops*)

***emarginatus*** (Olivier, 1792, *Formica*) E added by Pontin (2008)^[[89]](#footnote-89)^

*brunneoemarginatus* Forel, 1874

*brunneoides* Forel, 1874

*nigroemarginatus* Forel, 1874

*illyricus* Zimmermann, 1935

*pontica* Stärcke, 1944

***flavus*** (Fabricius, 1781, *Formica*) E S W I M

*ruficornis* (Fabricius, 1804, *Formica*)

*brevicornis* Emery, 1893

*fuscoides* Ruzsky, 1902

*odoratus* Ruzsky, 1905

*claripennis* Wheeler, 1917

*microps* Wheeler, 1917

*morbosa* (Bondroit, 1918, *Formicina*)

*ibericus* Santschi, 1925

*apennina* Menozzi, 1925

*olivacea* Karavaiev, 1926

*helvus* Cook, 1953

***fuliginosus*** (Latreille, 1798, *Formica*) E S W I M

***meridionalis*** (Bondroit, 1920, *Formicina*) E W

***mixtus*** (Nylander, 1846, *Formica*) E S W I

# ***# neglectus*** Van Loon, Boomsma & Andrasfalvy, 1990 E

added by Fox (2009)

***niger*** (Linnaeus, 1758, *Formica*) E S W I M

*nigerrima* (Christ, 1791, *Formica*)

*pallescens* (Schenck, 1852, *Formica*)

*alienoniger* Forel, 1874

*emeryi* Ruzsky, 1905

*nitidus* (Kuznetzsov-Ugamsky, 1927, *Acanthomyops*)

*minimus* (Kuznetsov-Ugamsky, 1928, *Acanthomyops*)

*transylvanica* Röszler, 1943

***platythorax*** Seifert, 1991 E S W I added by Seifert (1992)

***psammophilus*** Seifert, 1992 E W added by Seifert (1992)

***sabularum*** (Bondroit, 1918, *Formicina*) E W M

***umbratus*** (Nylander, 1846, *Formica*) E S W I M

*aphidicola* (Walsh, 1863, *Formica*)

*exacutus* Ruzsky, 1902

*affinoumbratus* Donisthorpe, 1914

*belgarum* (Bondroit, 1918, *Formicina*)

*silvestrii* Wheeler, 1928

*hirtiscapus* Stärcke, 1937

*osakana* Santschi, 1941

*nyaradi* (Röszler, 1943, *Chthonolasius*)

*epinotalis* Buren, 1944

***PARATRECHINA*** Motschulsky, 1863

# ***longicornis*** (Latreille, 1802, *Formica*)

*vagans* (Jerdon, 1851, *Formica*)

*gracilescens* (Nylander, 1856, *Formica*)

*currens* Motschoulsky, 1863

## ***PLAGIOLEPIS*** Mayr, 1861

*APOROMYRMEX* Faber, 1969

*PARAPLAGIOLEPIS* Faber, 1969

# ***schmitzii*** Forel, 1895 E^[[90]](#footnote-90)^

*barbara* Santschi, 1911

*crosi* Santschi, 1920

*madeirensis* Emery, 1921

Subfamily Myrmicinae Lepeletier, 1835^[[91]](#footnote-91)^

Tribe Attini Smith, 1858

***PHEIDOLE*** Westwood, 1841

# ***megacephala*** (Fabricius, 1793, *Formica*)^[[92]](#footnote-92)^

*edax* (Forskål, 1775, *Formica*)

*trinodis* (Losana, 1834, *Myrmica*)

*pusilla* (Heer, 1852, *Oecophthora*)

*laevigata* (Smith, 1855, *Myrmica*)

*agilis* (Smith, 1857, Myrmica)

*testacea* (Smith, 1858, *Atta*)

*janus* Smith, 1858

*suspiciosa* (Smith, 1859, *Myrmica*)

*laevigata* Mayr, 1862

*perniciosa* (Gerstäcker, 1862, *Oecophthora*)

*picata* Forel, 1891

*scabrior* Forel, 1891

*gietleni* Forel,1905

*bernhardae* Emery, 1915

Tribe Crematogastrini Forel, 1893

[***CARDIOCONDYLA*** Emery, 1869

***britteni*** Crawley, 1920^[[93]](#footnote-93)^]

[***CREMATOGASTER*** Lund, 1831

# ***scutellaris*** (Olivier, 1792, *Formica*)^[[94]](#footnote-94)^

*haematocephala* (Leach, 1825, *Formica*)

*rediana* (Gené, 1841, *Myrmica*)

*rubriceps* (Nylander, 1849, *Myrmica*)

*ruficeps* (Mayr, 1853, *Acrocoelia*)

*grouvellei* Bondroit, 1918

*lichtensteini* Bondroit, 1918

*corsica* Santschi, 1921

*degener* Santschi, 1937]

***FORMICOXENUS*** Mayr, 1855

*SYMMYRMICA* Wheeler, 1904

***nitidulus*** (Nylander, 1846, *Myrmica*) E S

*laeviuscula* (Förster, 1850, *Myrmica*)

*picea* (Wasmann, 1906, *Leptothorax*)

***LEPTOTHORAX*** Mayr, 1855^[[95]](#footnote-95)^

*DORONOMYRMEX* Kutter, 1945

*MYCHOTHORAX* Ruzsky, 1904

***acervorum*** (Fabricius, 1793, *Formica*) E S W I M

*lacteipennis* (Zetterstedt, 1838, *Myrmica*)

*nigrescens* Ruzsky, 1905

*superus* Ruzsky, 1905

*kamtschaticum* Ruzsky, 1920

*orientalis* (Kuznetsov-Ugamsky, 1928, *Mychothorax*)

***MYRMECINA*** Curtis, 1829

*ARCHAEOMYRMEX* Mann, 1921

***graminicola*** (Latreille, 1802, *Formica*) E W

*latreillei* Curtis, 1829

*striatula* (Nylander, 1849, *Myrmica*)

*bidens* (Förster, 1850, *Myrmica*)

*kutteri* Forel, 1914

*grouvellei* Bondroit, 1918

*gotlandica* Karavaiev, 1930

*oelandica* Karavaiev, 1930

*dentata* Santschi, 1939

***STRONGYLOGNATHUS*** Mayr, 1853

*MYRMUS* Schenck, 1853 preocc.

***testaceus*** (Schenck, 1852, *Eciton*) E

*emarginatus* (Schenck, 1852, *Myrmus*)

*diveri* Donisthorpe, 1936

***TEMNOTHORAX*** Mayr, 1861

*MACROMISCHA* Roger, 1863

*DICHOTHORAX* Emery, 1895

*MYRMOXENUS* Ruzsky, 1902

*PROTOMOGNATHUS* Wheeler, 1905

*ANTILLAEMYRMEX* Mann, 1920

*CROESOMYRMEX* Mann, 1920

*CHALEPOXENUS* Menozzi, 1923

*MYRMAMMOPHILUS* Menozzi, 1925

*MYRAFANT* Smith, 1950

*ICOTHORAX* Hamann & Klemm, 1967

***albipennis*** (Curtis, 1854, *Stenamma*) E W

*tuberum* misident. Orledge (1998)

*tuberointerruptus* (Bondroit, 1918, *Leptothorax*)

***interruptus*** (Schenck, 1852, *Myrmica*) E

*simpliciuscula* (Nylander, 1856, *Myrmica*)

*tuberoaffinis* (Bondroit, 1918, *Leptothorax*)

***nylanderi*** (Foerster, 1850, *Myrmica*) E W

*cingulata* (Schenck, 1852, *Myrmica*)

*nylanderocorticalis* (Forel, 1874, *Leptothorax*)

*nylanderotuberum* (Ruzsky, 1902, *Leptothorax*)

# ***unifasciatus*** (Latreille, 1798, *Formica*)^[[96]](#footnote-96)^

*anoplogynus* (Emery, 1869, *Leptothorax*)

*unifasciatointerruptus* (Forel, 1874, *Leptothorax*)

*kirillovi* (Ruzsky, 1905, *Leptothorax*)

*brauneri* (Karavaiev, 1937, *Leptothorax*)

*salina* (Karavaiev, 1937, *Leptothorax*)

*ucrainicus* (Arnol’di, 1977, *Leptothorax*)

***TETRAMORIUM*** Mayr, 1855

*TETROGMUS* Roger, 1857

*ANERGATES* Forel, 1874

*XIPHOMYRMEX* Forel, 1887

*TRIGLYPHOTHRIX* Forel, 1890

*RHOPTROMYRMEX* Mayr, 1901

*ATOPULA* Emery, 1912

*MACROMISCHOIDES* Wheeler, 1920

*LOBOMYRMEX* Kratochvil, 1941

*TELEUTOMYRMEX* Kutter, 1950

***atratulus*** (Schenck, 1852, *Myrmica*) E

*friedlandi* (Creighton, 1934, *Anergates*)

*#* ***bicarinatum*** (Nylander, 1846, *Myrmica*)

*guineense* misident.

*cariniceps* (Guérin-Méneville, 1852, *Myrmica*)

*kollari* (Mayr, 1853, *Myrmica*)

*modesta* (Smith, 1860, *Myrmica*)

*reticulata* (Smith, 1862, *Myrmica*)

***caespitum*** (Linnaeus, 1758, *Formica*)^[[97]](#footnote-97)^ E S W I M

*fusca* (Leach, 1825, *Formica*)

*fuscula* (Nylander, 1846, *Myrmica*)

*modesta* (Foerster, 1850, *Myrmica*)

*himalayanum* Viehmeyer, 1914

*hammi* Donisthorpe, 1915

*immigrans* Santschi, 1927

*indocile* Santschi, 1927

*transbaicalense* Ruzsky, 1936

*transversinodis* (Enzmann, 1946, *Myrmica*)

*fusciclavum* Consani & Zngheri, 1952

*jiangxiense* Wang & Xiao, 1988

# ***simillimum*** (Smith, 1851, *Myrmica*)

*parallela* (Smith, 1859, *Myrmica*)

*pygmaeum* Emery, 1877

*denticulatum* Forel, 1902

*bantouana* Santschi, 1910

*opacior* Forel, 1913

*exoleta* Santschi, 1914

*brevispinosa* (Borgmeier, 1928, *Wasmannia*)

*insulare* Santschi, 1928

Tribe Myrmicini Lepeletier, 1835

***MYRMICA*** Latreille, 1804

*SIFOLINIA* Emery, 1907

*SOMMIMYRMA* Menozzi, 1925

*SYMBIOMYRMA* Arnol’di, 1930

*PARAMYRMICA* Cole, 1957

*DODECAMYRMICA* Arnol’di, 1968

***hirsuta*** Elmes, 1978 E W

***karavajevi*** (Arnol’di, 1930, *Symbiomyrma*) E W

*laurae* misident.

*pechei* (Samsinak, 1957, *Sifolinia*)

*faniensis* van Boven, 1970

*winterae* (Kutter, 1973, *Sifolinia*)

***lobicornis*** Nylander, 1846 E S W

*denticornis* Curtis, 1854

*arduennae* Bondroit, 1911

*angustifrons* Stärcke, 1927

*brunescens* Karavaiev, 1929

*burtshakabramovitshi* Karavaiev, 1929

*starki* Karavaiev, 1929

*foreli* Santschi, 1931

*alpestris* Arnol’di, 1934

*kievensis* Karavaiev, 1934

*lissahorensis* Stitz, 1939

***lonae*** Finzi, 1926 S added by Seifert (2000)

***rubra*** (Linnaeus, 1758, *Formica*) E

*laevinodis* Nylander, 1846

*longiscapus* Curtis, 1854

*champlaini* Forel, 1901

*europaea* Finzi, 1926

*bruesi* Weber, 1947

*microrubra* Seifert, 1993

***ruginodis*** Nylander, 1846 E S W I M

*dimidiata* Say, 1836

*diluta* Nylander, 1849

*ruginodolaevinodis* Forel, 1874

*silvestrii* Wheeler, 1928

*sontica* Santschi, 1937

*yoshiokai* Weber, 1947

*macrogyna* Brian & Brian, 1949

*microgyna* Brian & Brian, 1949

*mutata* Sadil, 1952

***sabuleti*** Meinert, 1861 E S W I M

*scabrinodolobicornis* Forel, 1874

***scabrinodis*** Nylander, 1846 E S W I M

*rugulosoides* Forel, 1915

*pilosiscapus* Bondroit, 1920

*ahngeri* Karavaiev, 1926

*scabrinodosabuleti* Sadil, 1952

***schencki*** Viereck, 1903 E W I

*kutteri* Finzi, 1926

*subopaca* Arnol'di, 1934

*betuliana* Ruzsky, 1946

*schenckioides* Boer & Noordijk, 2005

***specioides*** Bondroit, 1918 E

*silvestrianum* Emery, 1924

*striata* Finzi, 1926

*sancta* Karavaiev, 1926

*nevodovskii* (Karavaiev, 1926, *Leptothorax*)

*turcica* Santschi, 1931

*puerilis* Stärcke, 1942

*dolens* Stärcke, 1942

*balcanica* Sadil, 1952

*scabrinodoides* Sadil, 1952

*tschuliensis* Arnol’di, 1976

*kozakorum* Radchenko & Elmes, 2010

***sulcinodis*** Nylander, 1846 E S W

*perelegans* Curtis, 1854

*nigripes* Ruzsky, 1895

*myrmecophila* Wasmann, 1910

*sulcinodoruginodis* Donisthorpe, 1915

*sulcinodoscabrinodis* Forel, 1915

*derzhavini* Ruzsky, 1920

*vicaria* Kuznetsov-Ugamsky, 1928

*eximia* Kupyanskaya, 1990

***vandeli*** Bondroit, 1920 E W added by Elmes *et al.* (2003)

Tribe Solenopsidini Forel, 1893

***MONOMORIUM*** Mayr, 1855

# ***floricola*** (Jerdon, 1851, *Atta*)

*cinnabari* Roger, 1863

*poecilum* Roger, 1863

*specularis* Mayr, 1866

*impressum* Smith, 1876

*philippinensis* Forel, 1910

*furina* Forel, 1911

*floreanum* Stitz, 1932

*angusticlava* Donisthorpe, 1947

# ***pharaonis*** (Linnaeus, 1758, *Formica*)

*antiguensis* (Fabricius, 1793, *Formica*)

*domestica* (Shuckard, 1838, *Myrmica*)

*minuta* (Jerdon, 1851, *Atta*)

*vastator* (Smith, 1857, *Myrmica*)

*contigua* (Smith, 1858, *Myrmica*)

*fragilis* (Smith, 1858, *Myrmica*)

***SOLENOPSIS*** Westwood, 1840

*DIPLORHOPTRUM* Mayr, 1855

*OCTELLA* Forel, 1915

*SYNSOLENOPSIS* Forel, 1918

*DIAGYNE* Santschi, 1923

*EUOPHTHALMA* Creighton, 1930

*LABAUCHENA* Santschi, 1930

*OEDALEOCERUS* Creighton, 1930

*BISOLENOPSIS* Kusnezov, 1953

*PARANAMYRMA* Kusnezov, 1953

*GRANISOLENOPSIS* Kusnezov, 1957

*LILIDRIS* Kusnezov, 1957

***fugax*** (Latreille, 1798, *Formica*) E

*flavidula* (Nylander, 1849, *Myrmica*)

*latroides* Ruzsky, 1905

*orientalis* Ruzsky, 1905

*kasalinensis* Emery, 1909

*pontica* Santschi, 1934

*scythica* Santschi, 1934

*furtiva* Santschi, 1934

*balachowskyi* Bernard, 1950

*banyulensis* Bernard, 1950

*duboscqui* Bernard, 1950

*laevithorax* Bernard, 1950

*monticola* Bernard, 1950

*nicaeensis* Bernard, 1950

*provincialis* Bernard, 1950

*pygmaea* Bernard, 1950

*richardi* Bernard, 1950

*robusta* Bernard, 1950

*rugosa* Bernard, 1950

*tertialis* Ettershank, 1966

*avium* (Bernard, 1978, *Diplorhoptrum*)

*delta* (Bernard, 1978, *Diplorhoptrum*)

*insulare* (Bernard, 1978, *Diplorhoptrum*)

*pilosum* (Bernard, 1978, *Diplorhoptrum*)

Tribe Stenammini Ashmead, 1905

***STENAMMA*** Westwood, 1839

*ASEMORHOPTRUM* Mayr, 1861

*THERYELLA* Santschi, 1921

***debile*** (Förster, 1850, *Myrmica*) E W I added by DuBois (1993)

*minkii* (Förster, 1850, *Myrmica*)

*golosejevi* Karavaiev, 1926

*ucrainicum* Arnol’di, 1928

*polonicum* Begdon, 1932

*orousseti* Casevitz-Weulerrse, 1990

***westwoodii*** Westwood, 1839 E^[[98]](#footnote-98)^

Subfamily Ponerinae Lepeletier, 1835

***HYPOPONERA*** Santschi, 1938

[# ***gibbinota*** (Forel, 1912, *Ponera*)^[[99]](#footnote-99)^]

# ***punctatissima*** (Roger, 1859, *Ponera*) E S W I

*androgyna* (Roger, 1859, *Ponera*)

*tarda* (Charsley, 1877, *Ponera*)

*jugata* (Forel, 1892, *Ponera*)

*brevis* (Santschi, 1911, *Ponera*)

*cognata* (Santschi, 1912, *Ponera*)

*durbanensis* (Forel, 1914, *Ponera*)

*incisa* (Santschi, 1914, *Ponera*)

*sordida* (Santschi, 1914, *Ponera*)

*petri* (Forel, 1916, *Ponera*)

*exacta* (Santschi, 1923, *Ponera*)

*mina* (Wheeler, 1927, *Ponera*)

*mumfordi* (Wheeler, 1931, *Ponera*)

*argonautorum* (Arnol’di, 1932, *Ponera*)

*mesoepinotalis* (Weber, 1942, *Ponera*)

*breviceps* (Bernard, 1953, *Ponera*)

*ursoidea* (Bernard, 1953, *Ponera*)

*sulcitana* (Stefani, 1970, *Ponera*)

# ***ergatandria*** (Forel, 1893, *Ponera*) E

*Ponera ergatandria* Forel, 1893

*schauinslandi* (Emery, 1899, *Ponera*) Seifert (2013)

*kalakauae* (Forel, 1899, *Ponera*)

*aemula* (Santschi, 1911, *Ponera*)

*bondroiti* (Forel, 1911, *Ponera*)

***PONERA*** Latreille, 1804

*PSEUDOCRYPTOPONE* Wheeler, 1933

*SELENOPONE* Wheeler, 1933

*PTEROPONERA* Bernard, 1950

***coarctata*** (Latreille, 1802, *Formica*) E W

*contracta* (Latreille, 1802, *Formica*)

*lucida* Emery, 1898

*atlantis* Santschi, 1921

***testacea*** Emery, 1895 E added by Attewell *et al.* (2010)

*crassisquama* Emery, 1916

Family **Mutillidae** Latreille, 1802^[[100]](#footnote-100)^

Subfamily Mutillinae Latreille, 1802

***MUTILLA*** Linnaeus, 1758

***europaea*** Linnaeus, 1758 E S

***SMICROMYRME*** Thomson, 1870

***rufipes*** (Fabricius, 1787, *Mutilla*) E

Subfamily Myrmosinae Fox, 1894

***MYRMOSA*** Latreille, 1796

***atra*** Panzer, 1801^[[101]](#footnote-101)^ E W I M

*melanocephala* (Fabricius, 1793, *Mutilla*) preocc.

Family **Pompilidae** Latreille, 1805^[[102]](#footnote-102)^

Psammocharidae Banks, 1910

Subfamily Pepsinae Lepeletier, 1845

Tribe Pepsini Lepeletier, 1845

***AUPLOPUS*** Spinola 1841

*PILPOMUS* Costa, 1859

*PSEUDAGENIA* Kohl, 1844

***carbonarius*** (Scopoli, 1763, *Sphex*) E W

*punctum* (Fabricius, 1804, *Ceropales*)

*canaliculatus* (Schenck, 1857, *Agenia*)

*submarginatus* Lepeletier, 1845

*albigena* Lepeletier, 1845

*collinus* Haupt, 1962

*silvalis* Haupt, 1962

***CALIADURGUS*** Pate, 1946

*CALICURGUS* Lepeletier, 1845 preocc.

***fasciatellus*** (Spinola, 1808, *Pompilus*) E W

*calcaratus* (Dahlbom, 1829, *Pompilus*)

*maculipennis* (Dahlbom, 1829, *Pompilus*)

*albispinus* (Herrich-Schäffer, 1830, *Pompilus*)

*curtus* (Zetterstedt, 1838, *Pompilus*)

*gyllenhali* (Dahlbom, 1843, *Priocnemis*)

*labiatus* (Lepeletier, 1845, *Anoplius*)

*unimacula* (Lepeletier, 1845, *Anoplius*)

*odontellus* (Lepeletier, 1845, *Calicurgus*)

*bivirgulatus* (Costa, 1881, *Pompilus*)

*fuscopennis* (Verhoeff, 1892, *Priocnemis*)

***CRYPTOCHEILUS*** Panzer, 1806

*CALICURGUS* Brullé, 1833

*CHLOROCHEILUS* Wolf, 1965 nom. nud.

subgenus ***ADONTA*** Bilberg, 1820

*SALIUS* Fabricius, 1804 preocc.

*HOMONOTUS* Dahlbom, 1845 preocc.

***notatus*** (Rossi, 1792, *Sphex*) E W

?*guttus* (Spinola, 1808, *Pompilus*)

*affinis* (Vander Linden, 1827, *Pompilus*)

*iracundus* (Dufour, 1841, *Pompilus*)

*apricus* (Lepeletier, 1845, *Calicurgus*)

*melanius* (Lepeletier, 1845, *Calicurgus*)

?*binotatus* (Marquet, 1879, *Priocnemis*) preocc.

*marquetii* (Dalla Torre, 1897, *Salius*)

*orientalis* Haupt, 1927

***DIPOGON*** Fox, 1897

subgenus ***DEUTERAGENIA*** Sustera, 1912

***bifasciatus*** (Geoffroy, 1785, *Ichneumon*) E

*hircanus* (Fabricius, 1798, *Pompilus*)

*intermedius* (Dahlbom, 1843, *Agenia*)

***subintermedius*** (Magretti, 1886, *Pogonius*) E S W

*nitidus* (Haupt, 1927, *Deuteragenia*)

***variegatus*** (Linnaeus, 1758, *Sphex*) E S W I

*erythropus* (Kohl, 1888, *Agenia*)

*structor* (Ferton, 1897, *Agenia*)

*faggiolii* (Haupt, 1927, *Deuteragenia*)

***PRIOCNEMIS*** Schiødte, 1837

subgenus ***PRIOCNEMIS*** Schiødte, 1837

*MACULIPENNIS* Junco, 1946

***agilis*** (Shuckard, 1837, *Pompilus*) E S W

*obtusiventris* Schiødte, 1837

*fraterculus* Junco, 1946

***confusor*** Wahis, 2006 E I

*gracilis* Haupt, 1927 preocc.

*gussakowskiji* Wolf, 2004 preocc.

***cordivalvata*** Haupt, 1927 E

***exaltata*** (Fabricius, 1775, *Sphex*) E S W I M

*gibba* (Scopoli, 1763, *Sphex*)

*revo* (Harris, 1780, *Sphex*)

*nudipes* Dahlbom, 1845

*longicornis* Haupt, 1927

*valkeilai* Wolf, 1959

***fennica*** Haupt, 1927 E W I

***hyalinata*** (Fabricius, 1793, *Sphex*) E W

*femoralis* (Dahlbom, 1829, *Pompilus*)

*discrepans* (Costa, 1887, *Pseudagenia*)

*trifurcus* Radoszkowski, 1888

*vitripennis* Verhoeff, 1892

*notatulus* (Saunders, 1896, *Salius*)

*pseudofemoralis* Sustera, 1938

*taigaica* Wolf, 1967

***parvula*** Dahlbom, 1845 E S W

*minor* (Zetterstedt, 1879, *Pompilus*)

*mocsaryi* Gussakovskij, 1930

*klosei* Haupt, 1937

*haupti* Sustera, 1938

*vinetorum* Blüthgen, 1944

*minutalis* Wahis, 1979 nec auct.

***propinqua*** (Lepeletier, 1845, *Calicurgus*) E

*agenoides* Dubois, 1920

***pusilla*** (Schiødte, 1837, *Pompilus*) E W

***schioedtei*** Haupt, 1927 E S W

subgenus ***UMBRIPENNIS*** Junco, 1946

*PRIOCNEMISSUS* Haupt, 1949

***coriacea*** Dahlbom, 1843 E

*capciosus* Junco, 1946

***perturbator*** (Harris, 1780, *Sphex*) E S W I

*fusca* misident.

*ambustor* (Panzer, 1804, *Ichneumon*)

*serripes* (Dahlbom, 1829, *Pompilus*)

*ambulator* (Lepeletier, 1845, *Calicurgus*)

*sepicola* (Smith, 1851, *Pompilus*)

*ater* Wolf, 1960

***susterai*** Haupt, 1927 E W

*clementi* Haupt, 1927

*gasconia* Wolf, 1975

Subfamily Pompilinae Latreille, 1805

***AGENIOIDEUS*** Ashmead, 1902

*APOROIDEUS* Ashmead, 1902

*GYMNOCHARES* Banks, 1917

***cinctellus*** (Spinola, 1808, *Pompilus*) E W

*clypeatus* (Dahlbom, 1829, *Pompilus*)

*punctipes* (Dahlbom, 1832, *Pompilus*)

*tibialis* (Lepeletier, 1845, *Anoplius*)

***sericeus*** (Vander Linden, 1827, *Pompilus*) E added by Baldock (2006)

*vicinus* (Lepeletier, 1845, *Pompilus*)

*subserricornis* (Kohl, 1879, *Pompilus*)

*declivus* (Tournier, 1889, *Pompilus*)

*gaullei* (Tournier, 1889, *Pompilus*)

*hungaricus* (Moczar, 1944, *Anospilus*)

***ANOPLIUS*** Dufour, 1834

subgenus ***ANOPLIUS*** Dufour, 1834

***caviventris*** (Aurivillius, 1907, *Pompilus*) E W

*cardui* (Perkins, 1917, *Pompilus*)

*carbonarius* Haupt, 1937

*atricolor* Moczar, 1944

***concinnus*** (Dahlbom, 1845, *Pompilus*) E S W I

*vacillans* (Wesmael, 1851, *Pompilus*)

*approximatus* (Smith, 1877, *Pompilus*)

*bifidus* (Morawitz, 1891, *Pompilus*)

*distinguendus* (Morawitz, 1891, *Pompilus*)

*haereticus* (Tournier, 1889, *Pompilus*)

***nigerrimus*** (Scopoli, 1763, *Sphex*) E S W I M

*nigrus* (Fabricius, 1775, *Sphex*)

*incisus* (Tischbein, 1850, *Pompilus*)

*melanarius* (Schenck, 1857, *Pompilus*) preocc.

*excerptus* (Tournier, 1889, *Pompilus*)

*difficilis* (Tournier, 1889, *Pompilus*)

*wheeleri* Banks, 1939

*banksi* Dreisbach, 1950

subgenus ***ARACHNOPHROCTONUS*** Howard, 1901

*POMPILINUS* Ashmead, 1902

***infuscatus*** (Vander Linden, 1827, *Pompilus*) E W

*minor* (Herrich-Schäffer, 1830, *Pompilus*)

*sericatus* (Shuckard, 1835, *Pompilus*)

*chalybeatus* (Schiødte, 1837, *Pompilus*)

*difformis* (Schiødte, 1837, *Pompilus*)

*dispar* (Dahlbom, 1843, *Pompilus*)

*sabulicola* (Thomson, 1874, *Pompilus*)

*meticulosa* (Costa, 1882, *Pompilus*)

*aeruginosus* (Tournier, 1890, *Pompilus*)

*aerarius* (Tournier, 1890, *Pompilus*)

*argentatus* (Tournier, 1890, *Pompilus*)

*calcatus* (Tournier, 1890, *Pompilus*)

*onus* (Tournier, 1890, *Pompilus*)

*stellatus* (Tournier, 1890, *Pompilus*)

*utendus* (Tournier, 1890, *Pompilus*)

*vivus* (Tournier, 1890, *Pompilus*)

*xysticus* (Tournier, 1890, *Pompilus*)

*petulans* Haupt, 1962

*cinctellus* (Haupt, 1962, *Paracyphonyx*)

*lusitanicus* Wolf & Diniz, 1970

*fortunatus* Wolf, 1975

*simii* Wolf, 1978

***viaticus*** (Linnaeus, 1758, *Sphex*) E W I

*fuscus* (Linnaeus, 1761, *Sphex*)

*paganus* (Dahlbom, 1843, *Pompilus*)

*propinquus* (Smith, 1879, *Pompilus*)

*tibialis* (Tournier, 1890, *Pompilus*) preocc.

*delatorius* (Tournier, 1890, *Pompilus*)

*immixtus* (Tournier, 1890, *Pompilus*)

*pleropicus* (Tournier, 1890, *Pompilus*)

*valesicus* (Tournier, 1890, *Pompilus*)

*macrurus* (Dalla Torre, 1897, *Pompilus*)

*holomelas* (Mantero, 1905, *Pompilus*) preocc.

***APORUS*** Spinola, 1808

***unicolor*** Spinola, 1808 E W

*femoralis* Vander Linden, 1827

*castor* (Kohl, 1838, *Pompilus*)

***ARACHNOSPILA*** Kincaid, 1900

subgenus ***AMMOSPHEX*** Wilcke, 1942

*ANOPOMPILINUS* Dreisbach, 1949

*ARIDOPOMPILUS* Wolf, 1965

*BOREOPOMPILUS* Wolf, 1965

*HOLARCTOPOMPILUS* Wolf, 1965

*SAXATILIPOMPILUS* Wolf, 1965

***anceps*** (Wesmael, 1851, *Pompilus*) E S W I M

?*vaga* (Harris, 1870, *Sphex*) preocc.

*unguicularis* (Thomson, 1870, *Pompilus*)

*crobaci* (Tournier, 1890, *Pompilus*)

*expleta* (Tournier, 1890, *Pompilus*)

*lustrica* (Tournier, 1890, *Pompilus*)

*nava* (Tournier, 1890, *Pompilus*)

*radiosa* (Tournier, 1890, *Pompilus*)

*saxea* (Tournier, 1890, *Pompilus*)

*peninsulana* (Wolf, 1966, *Pompilus*)

*serica* Wolf & Moczar, 1972

***consobrina*** (Dahlbom, 1843, *Pompilus*) E W

*ater* (Brullé, 1840, *Pompilus*) preocc.

*excisa* (Pérez, 1895, *Pompilus*) preocc.

*nivariae* (Dalla Torre, 1897, *Pompilus*)

*guimarensis* (Saunders, 1904, *Pompilus*)

*lanuginosa* (Haupt, 1927, *Psammochares*)

*heringi* (Haupt, 1928, *Psammochares*)

*emissa* (Haupt, 1930, *Psammochares*)

*laufferi* (Junco, 1960, *Pompilus*)

*alpina* (Wolf, 1965, *Pompilus*)

*pyrenaica* (Wolf, 1965, *Pompilus*)

*continentalis* (Wolf, 1966, *Pompilus*)

*sicula* (Wolf, 1966, *Pompilus*)

***trivialis*** (Dahlbom, 1843, *Pompilus*) E W

*gibba* misident.

*aerumnata* (Tournier, 1889, *Pompilus*)

*corruptor* (Haupt, 1927, *Psammochares*)

*michalki* (Blüthgen, 1961, *Ammosphex*)

*insubrica* (Wolf, 1965, *Pompilus*)

***wesmaeli*** (Thomson, 1870, *Pompilus*) E W

subgenus ***ANOPLOCHARES*** Banks, 1939

***minutula*** (Dahlbom, 1842, *Pompilus*) E W

*cellularis* (Dahlbom, 1843, *Pompilus*)

*neglecta* (Dahlbom, 1843, *Pompilus*)

*inermis* (Lepeletier, 1845, *Anoplius*)

*anoplius* (Dalla Torre, 1897, *Pompilus*)

*simplicicra* (Priesner, 1960, *Pompilus*)

*apenninusurata* Wolf, 1970

***spissa*** (Schiødte, 1837, *Pompilus*) E S W

*apennina* Wolf, 1970

subgenus ***ARACHNOSPILA*** Kincaid, 1900

*PYCNOPOMPILUS* Ashmead, 1902

***rufa*** (Haupt, 1927, *Psammochares*) E

*adulterina* (Haupt, 1937, *Psammochares*)

*melanota* Wolf, 1975

***EPISYRON*** Schiødte, 1837

*SPILOPOMPILUS* Ashmead, 1902

***gallicum*** (Tournier, 1889, *Pompilus*) E added by Baldock (2006)

*intermedius* Haupt, 1930

*tertius* Blüthgen, 1944

***rufipes*** (Linnaeus, 1758, *Sphex*) E W I

*laevigata* (Rossius, 1794, *Sphex*)

*gracilis* (Lepeletier, 1845, *Pompilus*) preocc.

*septemmaculatus* (Wesmael, 1851, *Pompilus*)

*argyrolepis* (Costa, 1887, *Pompilus*)

*compressus* (Tournier, 1889, *Pompilus*) preocc.

*aequatus* (Tournier, 1889, *Pompilus*)

*pygidialis* (Tournier, 1889, *Pompilus*)

*deuterus* (Dalla Torre, 1897, *Pompilus*)

*ephialtes* (Dalla Torre, 1897, *Pompilus*)

*sardous* Wolf, 1961

***EVAGETES*** Lepeletier, 1845

*SOPHROPOMPILUS* Ashmead, 1902

*ASTHENOCTENUS* Arnold, 1937 preocc.

*ASTHENOCTENIDIA* Pate, 1946

*LEUCHIMON* Haupt, 1930

*TRICHOSYRON* Haupt, 1930

*PSAMMOCHAROIDES* Moczar, 1946

*STREPTOSELLA* Dreisbach, 1950

*CARINEVAGETES* Wolf, 1970

*CONTEMPTEVAGETES* Wolf, 1970

***crassicornis*** (Shuckard, 1835, *Pompilus*) E S W I M

*dahlbomi* (Thomson, 1870, *Pompilus*)

*subarcticus* Wolf, 1964

***dubius*** (Vander Linden, 1827, *Aporus*) E

*bicolor* Lepeletier, 1845

*servillei* Costa, 1882

*rattus* (Dalla Torre, 1897, *Pompilus*)

*obscurodubius* Wolf, 1970

*theodori* Wolf, 1970

***pectinipes*** (Linnaeus, 1758, *Sphex*) E

*quadrispinosus* (Kohl, 1886, *Pompilus*)

*aculeatus* (Thomson, 1870, *Pompilus*)

*minotaurus* Wolf, 1970

***HOMONOTUS*** Dahlbom, 1843

*ISONOTUS* Dahlbom, 1842 preocc.

*WESMAELINIUS* Costa, 1886

***sanguinolentus*** (Fabricius, 1793, *Sphex*) E

*dispar* (Latreille, 1809, *Pompilus*)

*bidens* (Lepeletier, 1845, *Anoplius*)

*affinis* (Eversmann, 1849, *Pompilus*) preocc.

*nigrus* (Marquet, 1879, *Ferrola*)

*nasutus* Morawitz, 1888

*doctor* (Dalla Torre, 1897, *Pompilus*)

***POMPILUS*** Fabricius, 1798

*CHIONOPOMPILUS* Priesner, 1955

***cinereus*** (Fabricius, 1775, *Sphex*) E S W I M

*plumbeus* (Fabricius, 1787, *Sphex*)

*pulcher* Fabricius, 1798

*pruinosus* Smith, 1879 preocc.

*chevrieri* Tournier, 1889

*leprosus* Dalla Torre, 1897

*lusitanicus* Wolf & Diniz, 1970

*gotlandicus* Wolf, 1972

Subfamily Ceropalinae Radoszkowski, 1888

***CEROPALES*** Latreille, 1796

*CERATOPALES* Schulz, 1906

*HYPSICERAEUS* Morice & Durrant, 1915

***maculata*** (Fabricius, 1775, *Evania*) E S W I M

*rustica* (Müller, 1776, *Sphex*)

*multicolor* (Fourcroy, 1785, *Ichneumon*)

*frontalis* (Panzer, 1799, *Pompilus*)

*perligerus* (Costa, 1882, *Priocnemis*)

*balearica* Costa, 1893

***variegata*** (Fabricius, 1798, *Evania*) E

*destefanii* Costa, 1887

Family **Sapygidae** Latreille, 1810

***MONOSAPYGA*** Pic, 1920

***clavicornis*** (Linnaeus, 1758, *Apis*) E W

***SAPYGA*** Latreille, 1796

***quinquepunctata*** (Fabricius, 1781, *Scolia*) E W

Family **Tiphiidae** Leach, 1915

Subfamily Methochinae

***METHOCHA*** Latreille, 1804^[[103]](#footnote-103)^

*METHOCA* misspelling

***articulata*** Latreille, 1792 E W

?*formicaria* (Latreille, 1792, *Mutilla*) nom. dub.

*ichneumonides* Latreille, 1805

*mutillaria* Latreille, 1806

*sanvitali* (Latreille, 1809, *Tengyra*)

*italica* (Costa, 1858, *Spinolia*)

Subfamily Tiphiinae

***TIPHIA*** Fabricius, 1775

***femorata*** Fabricus, 1775 E W

***minuta*** Vander Linden, 1827 E S W I M

Family **Vespidae** Laicharting, 1781

Subfamily Eumeninae^[[104]](#footnote-104)^

***ANCISTROCERUS*** Wesmael, 1836

***antilope*** (Panzer, 1798, *Vespa*) E S W

*pictus* (Curtis, 1826, *Odynerus*)

***claripennis*** Thomson, 1874 E

?*quadratus* (Panzer, 1799, *Vespa*) nom. dub.^[[105]](#footnote-105)^

***gazella*** (Panzer, 1798, *Vespa*) E S W I

*emarginata* (Fabricius, 1793, *Vespa*) preocc.

***nigricornis*** (Curtis, 1826, *Odynerus*) E S W I M

*callosus* (Thomson, 1870, *Odynerus*)

*excisus* (Thomson, 1870, *Odynerus*)

*sexpunctata* (Christ, 1791, *Vespa*)

***oviventris*** (Wesmael, 1836, *Odynerus*)^[[106]](#footnote-106)^ E S W I M

*constans* (Herrich-Schäffer, 1839, *Odynerus*)

*viduus* (Herrich-Schäffer, 1839, *Odynerus*)

***parietinus*** (Linnaeus, 1758, *Vespa*) E S W I M

*domesticus* (Christ, 1791, *Sphex*)

*affinis* (Herrich-Schäffer, 1839, *Odynerus*)

***parietum*** (Linnaeus, 1758, *Vespa*) E S W I M

***scoticus*** (Curtis, 1826, *Odynerus*) E S W I M

*trimarginatus* misident.

*albotricinctus* (Zetterstedt, 1838, *Odynerus*)

***trifasciatus*** (Müller, 1776, *Vespa*) E S W I M

*trimarginatus* (Zetterstedt, 1838, *Odynerus*)

*tricinctus* (Herrich-Schäffer, 1839, *Odynerus*)

***EUMENES*** Latreille, 1802

***coarctatus*** (Linnaeus, 1758, *Vespa*) E W

*?****papillarius*** (Christ, 1791, *Sphex*)

***EUODYNERUS*** Dalla Torre, 1904

subgenus ***PAREUODYNERUS*** Blüthgen, 1938

***quadrifasciatus*** (Fabricius, 1793, *Vespa*) E

*tomentosus* (Thomson, 1870, *Odynerus*)

***GYMNOMERUS*** Blüthgen, 1938

***laevipes*** (Shuckard, 1837, *Odynerus*) E W

***MICRODYNERUS*** Thomson, 1874

***exilis*** (Herrich-Schäffer, 1839, *Odynerus*) E

*bivittatus* (Lepeletier, 1841, *Odynerus*)

***ODYNERUS*** Latreille, 1802

subgenus ***ODYNERUS*** Latreille, 1802

***melanocephalus*** (Gmelin, 1790, *Vespa*) E W

***spinipes*** (Linnaeus, 1758, *Vespa*) E S W I M

*quinquefasciata* (Fabricius, 1793, *Vespa*) preocc.

*muticus* (Zetterstedt, 1838, *Odynerus*)

subgenus ***SPINICOXA*** Blüthgen, 1938

***reniformis*** (Gmelin, 1790, *Vespa*) E

***simillimus*** Morawitz, 1867 E

***PSEUDEPIPONA*** Saussure, 1856

***herrichii*** (Saussure, 1855, *Odynerus*) E

*variegata* misident.

*basalis* (Smith, 1857, *Odynerus*)

***SYMMORPHUS*** Wesmael, 1836

***bifasciatus*** (Linnaeus, 1761, *Vespa*) E S W I M

*sinuatus* (Fabricius, 1793, *Vespa*) preocc.

*mutinensis* (Baldeni, 1894, *Odynerus*)

*sinuatissimus* Richards, 1935

***connexus*** (Curtis, 1826, *Odynerus*) E

*bifasciatus* misident.

***crassicornis*** (Panzer, 1798, *Vespa*) E W

***gracilis*** (Brullé, 1832, *Odynerus*) E W

Subfamily POLISTINAE^[[107]](#footnote-107)^

***POLISTES*** Latreille, 1802

***dominula*** (Christ, 1791, *Vespa*) E

*gallicus* misident.

*italica* Herrich-Schäffer, 1840 *nom. nud*.

*pectoralis* Herrich-Schäffer, 1841

*lefebvrei* Guérin, 1844

*bucharensis* Erichson, 1849

*maculatus* Rudow, 1889

*merceti* Dusmet, 1903

*rufescens* Buysson, 1912

*ornata* Weyrauch, 1938

*pacfica* Weyrauch, 1939 preocc.

*pseudopacificus* Giordani Soika, 1970

*muchei* Gusenleitner, 1976

***# gallicus*** (Linnaeus, 1767, *Vespa*) I^[[108]](#footnote-108)^ added by McClenaghan (1979)

*pictior* Radoszkowski, 1872

*foederata* Kohl, 1898

*omissa* (Weyrauch, 1938, *Polistula*)

Subfamily Vespinae^[[109]](#footnote-109)^

***DOLICHOVESPULA*** Rohwer, 1916

*PSEUDOVESPULA* Bischoff, 1931

*BOREOVESPULA* Blüthgen, 1943

*METAVESPULA* Blüthgen, 1943

***media*** (Retzius, 1783, *Vespa*) E S W M added by Falk (1982)

*geerii* (Lepeletier, 1836, *Vespa*)

*crassa* (Herrich-Schäffer, 1841, *Vespa*)

*similis* (Schenck, 1853, *Vespa*)

*rufoscutellata* (Schenck, 1853, *Vespa*)

*flavicincta* (Schenck, 1853, *Vespa*)

*lineolata* (Pérez, 1910, *Vespa*)

*conjugens* Paul, 1943

*sugare* Ishikawa, 1969

*borealis* Lee, 1986 preocc.

***norwegica*** (Fabricius, 1781, *Vespa*) E S W I M

*britannica* (Leach, 1814, *Vespa*)

*marginata* (Kirby, 1837, *Vespa*) preocc.

*borealis* (Zetterstedt, 1838, *Vespa*) preocc.

*albida* (Sladen, 1918, *Vespa*)

*arctica* (Friese, 1919, *Vespa*) preocc.

*zetterstedti* Blüthgen, 1937

***saxonica*** (Fabricius, 1793, *Vespa*) E W added by Allen & Archer (1989)

*bavarica* (von Schrank, 1802, *Vespa*)

*tridens* (Schenck, 1853, *Vespa*)

*nipponica* Yamane, 1975

*kamtschatkensis* Eck, 1983

*nigrescens* Eck, 1983

***sylvestris*** (Scopoli, 1763, *Vespa*) E S W I M

*parietum* (Harris, 1776, *Vespa*)

*holsatica* (Fabricius, 1793, *Vespa*)

*frontalis* (Latreille, 1802, *Vespa*)

*campanaria* (Fowler, 1833, *Vespa*)

*anglica* (Smith, 1843, *Vespa*)

*pilosella* (Costa, 1858, *Vespa*)

*sumptuosa* (De Buysson, 1905, *Vespa*)

*xinjiangensis* Lee, 1986

***VESPA*** Linnaeus, 1758

*MACROVESPA* Dalla Torre, 1904

*NYCTOVESPA* van der Vecht, 1959

***crabro*** Linnaeus, 1758^[[110]](#footnote-110)^ E W

*vexator* Harris, 1776

*major* Retzius, 1783

?*pratensis* Geoffroy, 1785

*germana* Christ, 1791

*crabroniformis* Smith, 1852

*borealis* Radoszkowski, 1863

*anglica* Gribodo, 1892 preocc.

*oberthuri* du Buysson, 1902

*flavo-fasciata* Cameron, 1903

*tartarea* du Buysson, 1905

*altaica* Pérez, 1910

*caspica* Pérez, 1910

*vulgata* Birula, 1925

*meridionalis* Birula, 1925

*chinensis* Birula, 1925 preocc.

*birulai* Bequaert, 1931

*gribodoi* Bequaert, 1931

***VESPULA*** Thomson, 1869

*PSEUDOVESPA* Schmiedeknecht, 1881

*PARAVESPULA* Blüthgen, 1938

*ALLOVESPULA* Blüthgen, 1943

*RUGOVESPULA* Archer, 1982

***austriaca*** (Panzer, 1799, *Vespa*) E S W I

*borealis* (Smith, 1843, *Vespa*) preocc.

*arborea* (Smith, 1849, *Vespa*)

*biloba* (Schilling, 1850, *Vespa*)

***germanica*** (Fabricius, 1793, *Vespa*) E S W I

?*maculata* (Scopoli, 1763, *Vespa*) preocc.

?*macularis* (Olivier, 1792, *Vespa*)

***rufa*** (Linnaeus, 1758, *Vespa*) E S W I M

*schrenckii* (Radoszkowski, 1861, *Vespa*)

*sibiria* (André, 1884, *Vespa*)

*grahami* Archer, 1981

*obscura* Lee, 1986

?*yichunensis* Lee, 1986

***vulgaris*** (Linnaeus, 1758, *Vespa*) E S W I M

*sexcincta* (Panzer, 1799, *Vespa*)

*pseudogermanica* (Stolfa, 1932, *Vespa*)

**References**

Agnoli, G. L. 2005. The genus *Methocha* in Europe: a discussion on taxonomy, distribution and likely origin of its known species and subspecies (Hymenoptera Tiphiidae Methochinae). *Bulletin of Insectology* **58**: 35-47.

Alford, D. V. 1975. *Bumblebees*: Davis-Poynter.

Allen, G. W. 1987. *Stigmus pendulus* Panzer (Hymenoptera: Sphecidae) new to Britain. *Entomologist's Gazette* **38**: 214.

Allen, G. W. and Archer, M. E. 1989. *Dolichovespula saxonica* (Fabricius, 1793) (Hym., Vespidae) found in Britain with a key to British *Dolichovespula*. *Entomologist’s Monthly Magazine* **125**: 103-105.

Archer, M. E. 2003. *The British Potter and Mason Wasps - A Handbook*: Bees, Wasps and Ants Recording Society.

Archer, M. E. 2007. Current knowledge of British aculeate Hymenoptera with special reference to the occurrence of high quality species on priority habitats. *British Journal of Entomology and Natural History* **20**: 75-94.

Archer, M. E. 2014. The vespoid wasps (Tiphiidae, Mutillidae, Sapygidae, Scoliidae and Vespidae) of the British Isles. *Handbooks for the Identification of British Insects* **6(vi)**: 1-82.

Archer, M. E. and Burn, J. T. 2012. Distributional records of the Bethylidae, Embolemidae and Dryinidae (Hymenoptera, Aculeata) from the British Isles. *BWARS Newsletter* **Spring 2012**: 19-25.

Attewell, P. J., Collingwood, C. A. and Godfrey, A. 2010. *Ponera testacea* (Emery, 1895) (Hym.: Formicidae) new to Britain from Dungeness, East Kent. *Entomologist’s Record and Journal of Variation* **122**: 113-119.

Baker, D. B. 1994. On the nomenclature of two sibling species of *Andrena tibialis* (Kirby, 1802) group (Hymenoptera, Apoidea). *Entomologist's Gazette* **45**: 281-290.

Baldock, D. 2006. Wildlife reports – Bees, wasps and ants. *British Wildlife* **17**: 361-363.

Baldock, D. and Hawkins, R. 2013. British *Hedychrum* may be two species. *BWARS Newsletter* **Spring 2013**: 54.

Bertsch, A., Schweer, H., Titze, A. and Tanaka, H. 2005. Male labial gland secretions and mitochondrial DNA markers support species status of *Bombus cryptarum* and *B. magnus* (Hymenoptera, Apidae). *Insectes Sociaux* **52**: 45-54.

Blüthgen, P. 1959. Beitrag zur Synonymie der palaarktischen Faltenwespen (Hym. Diploptera). *Deutsche Entomologische Zeitschrift (NF)* **6**: 148-151.

Bolton, B. 1995. A taxonomic and zoogeographical census of the extant ant taxa (Hymenoptera: Formicidae). *Journal of Natural History* **29**: 1037-1056.

Bolton, B. 2003. Synopsis and classification of Formicidae. *Memoirs of the American Entomological Institute* **71**: 1-370.

Brothers, D. J. 1975. Phylogeny and classification of the aculeate Hymenoptera, with special reference to the Mutillidae. *University of Kansas Science Bulletin* **50**: 483-648.

Burn, J. T. 1995. Two dryinids new to Britain (Hym., Aculeata, Chrysidoidea). *Entomologist’s Monthly Magazine* **131**: 1572-1575.

Burn, J. T. 1997. Additions and changes to the Chrysidoidea (Hym., Aculeata) occurring in Britain. *Entomologist's Monthly Magazine* **133**: 65-66.

Burn, J. T. and Olmi, M. 2011. *Gonatopus albosignatus* Kieffer (Hym., Dryinidae), a British species; a senior synonym of *Pseudogonatopus separatus* Richards. *Entomologist's Monthly Magazine* **147**: 1763-1765.

Carpenter, J. M. 1996. Distributional checklist of species of the genus *Polistes* (Hymenoptera: Vespidae; Polistinae, Polistini). *American Museum Novitates* **3188**: 1-39.

Carpenter, J. M. and Kojima, K. 1997. Checklist of the species in the subfamily Vespinae (Insecta: Hymenoptera: Vespidae). *Natural History Bulletin of Ibaraki University* **1**: 51-92.

Collins, G. A. and Roy, H. E. 2012. Provisional atlas of the aculeate Hymenoptera of Britain and Ireland. Part 8. In. Wallingford: Biological Records Centre.

Cross, I. C. 2002. *Colletes hederae* Schmidt & Westrich (Hym., Apidae) new to mainland Britain with notes on its ecology in Dorset. *Entomologist’s Monthly Magazine* **138**: 201-203.

Danforth, B. N., Fang, J. and Sipes, S. 2006. Analysis of family-level relationships in bees (Hymenoptera: Apiformes) using 28S and two previously unexplored nuclear genes: CAD and RNA polymerase II. *Molecular Phylogenetics and Evolution* **39**: 358-372.

Day, M. C. 1988. Spider wasps (Hymenoptera: Pompilidae). *Handbooks for the Identification of British Insects* **6(iv)**: 1-60.

Dollfuss, H. 1986. Eine Revision der Gattung *Spilomena* Shuckard der westlichen und zentralen palaärktischen Region (Hymenoptera, Sphecidae). *Annalen des Naturhistorischen Museum in Wien* **88-89B**: 481-510.

Dollfuss, H. 1995. A worldwide revision of *Pemphredon* Latreille 1796 (Hymenoptera, Sphecidae). *Linzer Biologische Beiträge* **27**: 905-1019.

DuBois, M. B. 1993. What's in a name? A clarification of *Stenamma westwoodi*, *S. debile*, and *S. lippulum*. *Sociobiology* **21**: 299-334.

Earwaker, R. and Sutton, P. 2015. *Hedychrum nobile* (Scopoli, 1763), and *Dasypoda hirtipes* (Fabricius, 1793), new to Bedfordshire. *BWARS Newsletter* **Spring 2015**: 19-20.

Edde, P. A. and Amatobi, C. I. 2000. Relative resistance of some cowpea varieties to *Callosobruchus maculatus* (F.) (Coleoptera: Bruchidae). *Journal of Sustainable Agriculture* **17**: 67-77.

Edwards, R. 1997. Provisional atlas of the aculeate Hymenoptera of Britain and Ireland. Part 1. In. Huntingdon: Biological Records Centre. p. 139.

Edwards, R. 1998. Provisional atlas of the aculeate Hymenoptera of Britain and Ireland. Part 2. In. Huntingdon: Biological Records Centre.

Edwards, R. and Broad, G. R. 2005. *Provisional atlas of the aculeate Hymenoptera of Britain and Ireland. Part 5*. Huntingdon: Biological Records Centre.

Edwards, R. and Broad, G. R. 2006. *Provisional atlas of the aculeate Hymenoptera of Britain and Ireland. Part 6*. Huntingdon: Biological Records Centre.

Edwards, R. and Roy, H. 2009. Provisional atlas of the aculeate Hymenoptera of Britain and Ireland. Part 7. In. Wallingford: Biological Records Centre. p. 145 pp.

Edwards, R. and Telfer, M. E. 2001. *Provisional atlas of the aculeate Hymenoptera of Britain and Ireland. Part 3*. Huntingdon: Biological Records Centre.

Edwards, R. and Telfer, M. E. 2002. *Provisional atlas of the aculeate Hymenoptera of Britain and Ireland. Part 4*. Huntingdon: Biological Records Centre.

Elmes, G. W., Radchemko, A. G. and Thomas, J. A. 2003. First records of *Myrmica vandeli* Bondroit (Hymenoptera, Formicidae) for Britain. *British Journal of Entomology and Natural History* **16**: 145-152.

Else, G. R. and Felton, J. C. 1994. *Mimumesa unicolor* (Van der Linden, 1829) (Hymenoptera: Sphecidae), a wasp new to the British list, with observations on related species. *Entomologist’s Gazette* **45**: 107-114.

Else, G. R. and Spooner, G. M. 1987. Hymenoptera: Aculeata. The Ants, Bees and Wasps. In: Shirt, D. B., editor. *British Red Data Books: 2. Insects*. Peterborough: Nature Conservancy Council.

Else, G. R., Burn, J. T., Olmi, M. and Bolton, B. 2004. Check list of British Hymenoptera Aculeata. In: Archer, M. E., editor. *BWARS Members' Handbook*. Huntingdon: Centre for Ecology & Hydrology. p. 113-131.

Engel, M. S. 1999. The taxonomy of recent and fossil honey bees (Hymenoptera: Apidae; *Apis*). *Journal of Hymenoptera Research* **8**: 165-196.

Engel, M. S. 2005. Family-group names for bees (Hymenoptera: Apoidea). *American Museum Novitates* **3476**: 1-33.

Falk, S. J. 1982. *Dolichovespula media* (Retzius) – a new British social wasp. *Proceedings and Transactions of the British Entomological and Natural History Society* **15**: 14-16.

Felton, J. C. 1987. The genus *Nitela* (Latreille) (Hym., Sphecidae) in Southern England. *Entomologist's Monthly Magazine* **123**: 235-238.

Felton, J. C. 1988. The genus *Trypoxylon* Latreille (Hym., Sphecidae) in Kent, with a first record for *T. minus* de Beaumont. *Entomologist's Monthly Magazine* **124**: 221-224.

Fox, M. 2009. *Lasius neglectus* (the elecric ant?) new to Britain. *BWARS Newsletter* **Autumn 2009**: 18.

Fox, M. 2011. *Temnothorax unifasciatus* and *T. tuberum* in Britain. *BWARS Newsletter* **Autumn 2011**: 24.

Gayubo, S. F. and Felton, J. C. 2000. The European species of the genus *Nitela* Lareille, 1809 (Hymenoptera: Sphecidae). *Annales de la Société entomologique de France* **(N.S.) 36**: 291-313.

Gordh, G. and Móczár, L. 1990. A catalog of the world Bethylidae (Hymenoptera: Aculeata). *Memoirs of the American Entomological Institute* **46**: 1-364+vii.

Goulson, D. and Williams, P. 2001. *Bombus hypnorum* (Hymenoptera: Apidae), a new British bumblebee? *British Journal of Entomology and Natural History* **14**: 129-131.

Guichard, K. M. 2002. *Passaloecus turionum* Dahlbom, 1845 (Hymenoptera, Sphecidae) new to the British list. *Entomologist's Gazette* **53**: 33-36.

Gusenleitner, F. and Schwarz, M. 2002. Weltweite Checkliste der Bienengattung *Andrena* mit Bemerkungen und Ergänzungen zu paläarktischen Arten (Hymenoptera, Apidae, Andreninae, *Andrena*). *Entomofauna* **Supplement 12**: 1-1280.

Gusenleitner, F. and Schwarz, M. 2012. The valid name for Megachile leachella Curtis 1828 (Hymenoptera: Apidae) and some comments. *Linzer Biologische Beiträge* **44**: 863-873.

Hawkins, R. D. 2011. *Lasioglossum sexstrigatum* (Hymenoptera: Apidae, Halictinae) new to Britain. *British Journal of Entomology and Natural History* **24**: 90-92.

Hilpert, H. 1989. Zum Vorkommen einiger Dryiniden in Südwestdeutschland sowie Bemerkungen zu *Embolemus ruddii* Westwood, 1833. *Spixiana* **11**: 263-269.

Jervis, M. A. 1977. A new key for the identification of the British species of *Aphelopus* (Hym; Dryinidae). *Systematic Entomology* **2**: 301-303.

Kimsey, L. S. and Carpenter, J. M. 2012. The Vespinae of North America (Vespidae, Hymenoptera). *Journal of Hymenoptera Research* **28**: 37-65.

Knowles, A. P. and Else, G. R. 2006. *Miscophus bicolor* Jurine (Hymenoptera: Crabronidae), a wasp new to Britain. *British Journal of Entomology and Natural History* **18**: 229-232.

Kunz, P. X. 1994. Die Goldwespen (Chrysididae) Baden-Wurttembergs. Taxonomie, Bestimmung, Verbreitung, Kartierung und Okologie - mit einem Bestimmungsschlussel fur die deutschen Arten. *Beihefte zu den Veroeffentlichungen uer Naturschutz und Landschaftspflege in Baden-Wuerttemberg* **77**: 1-188.

Lelej, A. S. 2002. *Catalogue of the Mutillidae (Hymenoptera) of the Palaearctic region*. Vladivostok: Dalnauka.

McClenaghan, I. 1979. *Polistes omissus* Weyrauch (Hymenoptera: Vespidae) in Bangor, Co. Down: a species new to British list. *Irish Naturalists' Journal* **19**: 448.

Mitroiu, M., Gusenleitner, J. and Noyes, J. S. 2004. *Fauna Europaea: Hymenoptera: Vespidae: Eumeninae*: http://www.fauna-eu.org/.

Morgan, D. 1984. Cuckoo-wasps. Hymenoptera, Chrysididae. *Handbooks for the Identification of British Insects* **6(v)**: 1-37.

Notton, D. G. and Dathe, H. H. 2008. William Kirby’s types of *Hylaeus* Fabricius (Hymenoptera, Colletidae) in the collection of the Natural History Museum, London. *Journal of Natural History* **42**: 1861-1865.

Notton, D. G., Popovici, O. A., Achterberg, C. v., De Rond, J. and Burn, J. T. 2014. Parasitoid wasps new to Britain (Hymenoptera: Platygastridae, Eurytomidae, Braconidae & Bethylidae). *European Journal of Taxonomy* **99**: 1-20.

O'Connor, J. P., Nash, R. and Broad, G. R. 2009. *An annotated checklist of the Irish Hymenoptera*. Dublin: The Irish Biogeographical Society.

Olmi, M. 1984. A revision of the Dryinidae (Hymenoptera). *Memoirs of the American Entomological Institute* **37**: 1-1913.

Olmi, M. 1989. Supplement to the revision of the world Dryinidae (Hymenoptera Chrysidoidea). *Frustula Entomologica (n.s.)* **12**: 109-395.

Orledge, G. M. 1998. The identity of *Leptothorax albipennis* (Curtis) (Hymenoptera: Formicidae) and its presence in Great Britain. *Systematic Entomology* **23**: 25-33.

Packer, L. 1981. *Crossocerus distinguendus* (Morawitz) (Hym., Sphecidae), a crabronine wasp new to Britain. *Entomologist's Monthly Magazine* **117**: 97-98.

Perkins, J. F. 1976. Hymenoptera: Bethyloidea (excluding Chrysididae). *Handbooks for the Identification of British Insects* **6(iiia)**: 1-38.

Pesenko, Y. A. 2007. Subgeneric classification of the Palaearctic bees of the genus *Evylaeus* Robertson (Hymenoptera: Halictidae). *Zootaxa* **1500**: 1-54.

Polaszek, A. and Krombein, K. V. 1994. The genera of Bethylinae (Hymenoptera: Bethylidae). *Journal of Hymenoptera Research* **3**: 91-105.

Pontin, A. J. 2008. *Lasius emarginatus* (Olivier, 1792) (Hym., Formicidae) in the Isle of Wight. *Entomologist’s Monthly Magazine* **144**: 156.

Pulawski, W. J. 1984. The status of *Trypoxylon figulus* (Linnaeus, 1758), *medium* de Beaumont, 1945, and *minus* de Beaumont, 1945 (Hymenoptera: Sphecidae). *Proceedings of the California Academy of Sciences* **43**: 123-140.

Pulawski, W. J. 2013. *Catalog of Sphecidae sensu lato (=Apoidea excluding Apidae)*: http://www.calacademy.org/scientists/projects/catalog-of-sphecidae.

Richards, O. W. 1978. Aculeata. Pp. 126-140 in: Fitton, M. G., Graham, M. W. R. de V., Bouček, Z. R. J., Fergusson, N. D. M., Huddleston, T., Quinlan, J., Richards, O. W., editors. *Kloet and Hincks. A check list of British insects. Part 4: Hymenoptera*: Handbooks for the Identification of British Insects 11, ix + 159 pp.

Richards, O. W. 1939. The British Bethylidae (s. l.) (Hymenoptera). *Transactions of the Royal Entomological Society of London* **89**: 185-344.

Richards, O. W. 1980. Scolioidea, Vespoidea and Sphecoidea. Hymenoptera, Aculeata. *Handbooks for the Identification of British Insects* **6(iiib)**: 1-118.

Rond, J. de 1994. *Bethylus hyalinus*: a freak after all! (Hymenoptera: Bethylidae). *Entomologische Berichten* **54(2)**: 20-22.

Rosa, P., Forshage, M., Paukkunen, J. and Soon, V. 2015. *Cleptes pallipes* Lepeletier synonym of *Cleptes semiauratus* (Linnaeus) and description of *Cleptes striatipleuris* sp. nov. (Hymenoptera: Chrysididae, Cleptinae). *Zootaxa* **4039**: 543-552.

Schlick-Steiner, B. C., Steiner, F. M., Moder, K., Seifert, B., Sanetra, M., Dyreson, E., Stauffer, C. and Christian, E. 2006. A multidisciplinary approach reveals cryptic diversity in Western Palearctic *Tetramorium* ants (Hymenoptera: Formicidae). *Molecular Phylogenetics and Evolution* **40**: 259-273.

Schwarz, M., Gusenleitner, F., Westrich, P. and Dathe, H. H. 1996. Katalog der Bienen Österreichs, Deutschlands und der Schweiz (Hymenoptera, Apidae). *Entomofauna* **Supplement 8**: 1-398.

Seifert, B. 1992. A taxonomic revision of the Palaearctic members of the ant subgenus Lasius s. str. (Hymenoptera, Formicidae). *Abhandlungen und Berichte des Naturkundemuseums Görlitz* **66**: 1-67.

Seifert, B. 2000. *Myrmica lonae* Finzi, 1926 - a species separate from *Myrmica sabuleti* Meinert, 1861 (Hymenoptera: Formicidae). *Abhandlungen und Berichte des Naturkundemuseums Görlitz* **72**: 195-205.

Seifert, B. 2004. The "Black Bog Ant" *Formica picea* NYLANDER, 1846 - a species different from *Formica candida* SMITH, 1878 (Hymenoptera: Formicidae). *Myrmecologische Nachrichten* **6**: 29-38.

Seifert, B. 2012. Clarifying naming and identification of the outdoor species of the ant genus *Tapinoma* Förster, 1850 (Hymenoptera: Formicidae) in Europe north of the Mediterranean region with description of a new species. *Myrmecological News* **16**: 139-147.

Seifert, B. 2013. *Hypoponera ergatandria* (Forel, 1893) – a cosmopolitan tramp species different from *H. punctatissima* (Roger, 1859) (Hymenoptera: Formicidae). *Soil Organisms* **85**: 189-201.

Smith, M. N. and Williams, M. A. 2008. *Lasius emarginatus* (Olivier, 1792) (Hymenoptera: Formicidae) confirmed as a British species. *BWARS Newsletter* **Autumn 2008**: 14-15.

Soon, V., Budrys, E., Orlovskyte, S., Paukkunen, J., Ødegaard, F., Ljubomirov, T. and Saarma, U. 2014. Testing the validity of Northern European species in the *Chrysis ignita* species group (Hymenoptera: Chrysididae) with DNA Barcoding. *Zootaxa* **3786**: 301-330.

Terayama, M. 2003. Phylogenetic Systematics of the Family Bethylidae (Insecta: Hymenoptera) Part II. Keys to subfamilies, tribes and genera in the world. *Academic Reports of the Faculty of English of Tokyo Polytechnic University* **26**: 16-29.

van der Vecht, J. and Fischer, F. C. J. 1972. *Hymenopterorum Catalogus (nova editio). Pars 8. Palaearctic Eumenidae*: W. Junk.

Vikberg, V. 2000. A re-evaluation of five European species of *Spilomena* with a key to European species and relevance to the fauna of North Europe, especially Finland (Hymenoptera: Pemphredonidae). *Entomologica Fennica* **11**: 35-55.

Wahis, R. 1986. Catalogue systematique et codage des hymenopteres pompilides de la region ouest-Europeene. Notes Fauniques de Gembloux. *Notes Fauniques de Gembloux* **12**.

Wahis, R. 2006. Mise à jour du Catalogue systématique des Hyménoptères Pompilides de la région oues-européene. Additions et Corrections. *Notes Fauniques de Gembloux* **59**: 31-36.

Ward, P. S., Brady, S. G., Fisher, B. L. and Schultz, T. R. 2015. The evolution of myrmicine ants: phylogeny and biogeography of a hyperdiverse ant clade (Hymenoptera: Formicidae). *Systematic Entomology* **40**: 61-81.

Williams, P. H., Cameron, S. A., Hines, H. M., Cederberg, B. and Rasmont, P. 2008. A simplified subgeneric classification of the bumblebees (*Bombus*). *Apidologie* **39**: 46-74.

Williams, P. H., Brown, M. J. F., Carolan, J. C., An, J., Goulson, D., Aytekin, A. M., Best, L. R., Byvaltsev, A. M., Cederberg, B., Dawson, R., Huang, J., Ito, M., Monfared, A., Raina, R. H., Schmid-Hempel, P., Sheffield, C. S., Sima, P. and Xie, Z. 2012. Unveiling cryptic species of the bumblebee subgenus *Bombus s. str.* worldwide with COI barcodes (Hymenoptera: Apidae). *Systematics and Biodiversity* **10**: 21-56.

1. The higher-level classification of the Apoidea follows Pulawski (2013) and Engel (2005). The ‘traditional’ family Sphecidae is paraphyletic with respect to the bee families (here referred to as the Anthophila, an informal taxon, following Engel, 2005) and in Britain and Ireland comprises the families Crabronidae and Sphecidae. Species-level classification of Crabronidae and Sphecidae follows Pulawski (2013). [↑](#footnote-ref-1)
2. Based on pers. comm. from D. Baldock. [↑](#footnote-ref-2)
3. The British population is considered to belong to the subspecies *annulatus* Lepeletier & Brullé (synonyms: *proximus*, *hyalinus*, *transversalis*, *obliquus*, *propinquus*, *berlandi*). [↑](#footnote-ref-3)
4. Represented by the subspecies *armatus* (Vander Linden, 1829, *Crabro*). [↑](#footnote-ref-4)
5. Earlier records of *unicolor* mostly refer to *nitidus*. [↑](#footnote-ref-5)
6. Only recorded as a vagrant in Deal, Kent; resident on the Channel Isles, which lie outside of the geographical coverage of this checklist. [↑](#footnote-ref-6)
7. Recorded initially as *Nitela spinolae* Latreille, 1809 (Felton, 1987) but described later as a new species (Gayubo & Felton, 2000). [↑](#footnote-ref-7)
8. Taxonomy follows Dollfuss (1995). [↑](#footnote-ref-8)
9. Richards (1980) refers to this species as *Pemphredon enslini* (Wagner, 1932, *Dineurus*). There has been much confusion over the identities of these taxa (see Knowles in Collins & Roy, 2012) but Pulawski (2013) treats *austriaca* and *enslini* as separate species. [↑](#footnote-ref-9)
10. Has been called *curruca* (Dahlbom, 1844, *Celia*) in several publications, following Dollfuss’s (1986) synonymy, but was reinstated as a valid species by Vikberg (2000). [↑](#footnote-ref-10)
11. Misidentification of American authors in older literature. [↑](#footnote-ref-11)
12. Engel (2005) proposed that the name Anthophila, without formal rank, be used for the bees. Note that ‘apiformes’ (and the corresponding ‘spheciformes’) is an equivalent term proposed by Brothers (1975). [↑](#footnote-ref-12)
13. Taxonomy mostly follows Gusenleitner & Schwarz (2002). [↑](#footnote-ref-13)
14. Probably extinct in Britain. [↑](#footnote-ref-14)
15. The summer brood of *rosae*. [↑](#footnote-ref-15)
16. Although usually referred to as *scotica* in Britain (e.g. Else *et al.*, 2004), Schwarz *et al.* (1996) and Gusenleitner & Schwarz (2002) have treated *scotica* as a synonym of *carantonica* Peréz, 1916; however, Else (in prep.), following P. Westrich’s (in lit.) interpretation of the type of *carantonica*, regards this is a separate species, with *carantonica* a junior synonym of *trimmerana*. [↑](#footnote-ref-16)
17. The population on the Isles of Scilly and Channel Islands has been described as the subspecies *sarnia* Richards, 1979. [↑](#footnote-ref-17)
18. Probably extinct in Britain. [↑](#footnote-ref-18)
19. Probably extinct in Britain. [↑](#footnote-ref-19)
20. Probably extinct in Britain. [↑](#footnote-ref-20)
21. Probably extinct in Britain. [↑](#footnote-ref-21)
22. Although treated as a synonym of *pilipes* by Gusenleitner & Schwarz (2002*a*) here *nigrospina* is treated as a valid species, following Baker (1994). [↑](#footnote-ref-22)
23. Probably extinct in Britain. [↑](#footnote-ref-23)
24. Probably extinct in Britain. [↑](#footnote-ref-24)
25. There have been various subspecies names proposed for honey bees, with the supposedly native north west European population being *A. mellifera mellifera*. See Engel (1999) for a full taxonomy of *Apis mellifera*. [↑](#footnote-ref-25)
26. Various subspecific names have been used for British *Bombus* but these are generally poorly justified and we have treated these as synonymous here. Subgeneric classification follows Williams *et al.* (2008). [↑](#footnote-ref-26)
27. Full synonymy for this subgenus is given by Williams *et al.* (2012), who shed some light on the confusing classification of *cryptarum* and *magnus* relative to *lucorum*. [↑](#footnote-ref-27)
28. Extinct in Britain since the 1940s. [↑](#footnote-ref-28)
29. Populations in Ireland have been referred to the subspecies *B. hortorum ivernicus*. [↑](#footnote-ref-29)
30. English populations have been referred to the subspecies *B. ruderatus perniger*. [↑](#footnote-ref-30)
31. Populations in Western Scotland have been referred to the subspecies *B. campestris swynnertoni*. [↑](#footnote-ref-31)
32. The population on the Hebrides has been referred to the subspecies *B. jonellus hebridensis*. [↑](#footnote-ref-32)
33. The population on the Isle of Man has been referred to the subspecies *B. jonellus monapiae*. [↑](#footnote-ref-33)
34. A replacement name for the preccupied *vogtii*; populations on the Shetlands have been referred to the subspecies *B. jonellus vogtianus*. [↑](#footnote-ref-34)
35. Extinct in Britain since the 1980s but an attempt is being made to reintroduce the species to southern England. [↑](#footnote-ref-35)
36. British populations have been referred to the subspecies *B. subterraneus latreillellus* whereas the introduced individuals from Sweden have been ascribed to the nominate subspecies. [↑](#footnote-ref-36)
37. Populations on the Irish mainland and southern England have been referred to the subspecies *B. muscorum sladeni*. [↑](#footnote-ref-37)
38. The population on the Aran islands has been referred to the subspecies *B. muscorum allenellus*. [↑](#footnote-ref-38)
39. The population on Orkney has been referred to the subspecies *B. muscorum orcadensis*. [↑](#footnote-ref-39)
40. Populations on the Isles of Scilly have been referred to the subspecies *B. muscorum scyllonius*. [↑](#footnote-ref-40)
41. Populations in mainland Scotland and Northern England have been referred to the subspecies *B. muscorum celticus*. [↑](#footnote-ref-41)
42. Populations on the Shetland islands have been referred to the subspecies *B. muscorum agricolae*. [↑](#footnote-ref-42)
43. Populations in southern England and Wales have been referred to the subspecies *B. pascuorum vulgo*. [↑](#footnote-ref-43)
44. Populations in Ireland have been referred to the subspecies *B. pascuorum floralis*. [↑](#footnote-ref-44)
45. Populations in Scotland and Northern England have been referred to the subspecies *B. pascuorum septentrionalis*. [↑](#footnote-ref-45)
46. There is confusion surrounding the dates of capture of the supposedly British specimens of *pomorum*, collected near Deal in Kent (summarised by Alford, 1975). [↑](#footnote-ref-46)
47. Probably extinct in Britain. [↑](#footnote-ref-47)
48. Probably extinct in Britain. [↑](#footnote-ref-48)
49. The British population has been described as a separate subspecies, *celticus* O’Toole, 1974, and Else, Field & O’Toole (in Edwards, 1997) suggest that the British population may be specifically distinct. [↑](#footnote-ref-49)
50. Probably extinct in Britain [↑](#footnote-ref-50)
51. Notton & Dathe (2008) designated as lectotype a female specimen that corresponds to the species here called *annularis*, with the species generally called *annularis* taking the name *dilatatus*. [↑](#footnote-ref-51)
52. Probably extinct in Britain. [↑](#footnote-ref-52)
53. The subspecies *perkinsi* Blüthgen, 1926, is recognised from Britain. [↑](#footnote-ref-53)
54. Probably extinct in Britain. [↑](#footnote-ref-54)
55. Nomenclature updated from Pesenko (2007) but, as Pesenko’s study was limited to the Palaearctic region, we do not follow his elevation of *Evylaeus* to a valid genus, nor have we adopted his numerous subgenera. [↑](#footnote-ref-55)
56. The Irish population has been described as the subspecies *hibernicum* Ebmer, 1970, with other populations referred to the subspecies *scoticum* Ebmer, 1970. [↑](#footnote-ref-56)
57. Probably extinct in Britain. [↑](#footnote-ref-57)
58. Probably extinct in Britain. [↑](#footnote-ref-58)
59. Probably extinct in Britain. [↑](#footnote-ref-59)
60. Probably extinct in Britain. [↑](#footnote-ref-60)
61. Represented by the subspecies *nigrithorax* Dalla Torre, 1877 [↑](#footnote-ref-61)
62. Probably extinct in Britain. [↑](#footnote-ref-62)
63. Probably extinct in Britain. [↑](#footnote-ref-63)
64. Includes the subspecies *hiberniae* Perkins, 1925. [↑](#footnote-ref-64)
65. Probably extinct in Britain. [↑](#footnote-ref-65)
66. Includes the nominate subspecies and *hibernica* Perkins, 1925 [↑](#footnote-ref-66)
67. Probably extinct in Britain. [↑](#footnote-ref-67)
68. According to Danforth *et al.* (2006), Dasypodainae may be better treated as a separate family, but this requires further corroboration. [↑](#footnote-ref-68)
69. Some distribution records taken from Archer & Burn (2012). [↑](#footnote-ref-69)
70. Taxonomy mostly follows Gordh & Móczár (1990). Distribution data from Perkins (1976). [↑](#footnote-ref-70)
71. Listed as a separate species (described from English specimens, now missing) by Gordh & Móczár (1990) but we follow Richards (1939) who believed this not to be a separate species from *formiciformis*. [↑](#footnote-ref-71)
72. Supposedly described from the New Forest, the type is lost and the species has not been satisfactorily interpreted since. [↑](#footnote-ref-72)
73. Synonymy and distribution data from Notton *et al.* (2014). [↑](#footnote-ref-73)
74. Synonymy follows Rosa *et al.* (2015). [↑](#footnote-ref-74)
75. Some distribution data from Morgan (1984). [↑](#footnote-ref-75)
76. The number of species recognised in the *Chrysis ignita* group has varied from author to author. Soon *et al.*’s (2014) analysis of species limits found there to be 15 described European species plus cryptic species, a results more in line with Morgan’s (1984) recognition of multiple species than, for example, Kunz’s (1994) much more conservative treatment. [↑](#footnote-ref-76)
77. Tentative identification by Baldock & Hawkins (2013) but subsequently confirmed (e.g. Earwaker & Sutton, 2015). [↑](#footnote-ref-77)
78. Treated as a subspecies of *aeneus* in Fauna Europaea. [↑](#footnote-ref-78)
79. Some distributional data and synonymy from Olmi (1984), supplemented by Perkins (1976) and Olmi (1989). [↑](#footnote-ref-79)
80. *Sensu* Richards (1939) and Perkins (1976). [↑](#footnote-ref-80)
81. Included as a synonym of *brachycerum* by Richards (1978). [↑](#footnote-ref-81)
82. Data on five species reared in Wales from Jervis (1977). [↑](#footnote-ref-82)
83. Some distribution data for Mutillidae, Sapygidae, Tiphiidae and Vespidae from Archer (2014). [↑](#footnote-ref-83)
84. Nomenclature, synonymy and higher classification based on Bolton (1995, 2003) and AntWeb (http://www.antweb.org/), with additional references given. [↑](#footnote-ref-84)
85. Although this highly invasive species has the potential to establish itself in hothouses, given the correct climate, I am unaware of any established colonies being found in Britain. Any introductions have been of isolated workers. [↑](#footnote-ref-85)
86. Seifert (2012) clarified the identity of the north European *Tapinoma*. [↑](#footnote-ref-86)
87. Not a homonyn under article 23.9.5 of the Code (Seifert, 2004). [↑](#footnote-ref-87)
88. Probably extinct in Britain (Edwards & Broad, 2005). [↑](#footnote-ref-88)
89. Further records are given by Smith & Williams (2008). [↑](#footnote-ref-89)
90. Two nests persisted on the Isle of Wight from 2007-2008 (BB, pers. obs., specimens in NHM, BB coll. and B. Seifert coll.) but appear to have died out since. A native of the Azores and Madeira. [↑](#footnote-ref-90)
91. The tribal classification follows Ward *et al.* (2015). [↑](#footnote-ref-91)
92. A tramp species, in the past regarded as a proper introduction in Britain but no established colonies have been reported in the period 1970–present. On a world-wide scale its importance as a tramp species seems to be diminishing. The origin of *megacephala* is certainly Afrotropical as it is a member of a large and successful species group that is otherwise confined to that region. [↑](#footnote-ref-92)
93. Described from a worker casually introduced in Britain; included here only because the type-locality is Britain. [↑](#footnote-ref-93)
94. An occasional accidental import with cork from southern Europe. It has never established in Britain and, with the decrease in the cork trade, imports of this species will probably become even rarer. [↑](#footnote-ref-94)
95. Many species previously placed in *Leptothorax* have been transferred to *Temnothorax* by Bolton (2003). [↑](#footnote-ref-95)
96. Recorded from England as an accidental introduction by Fox (2011). [↑](#footnote-ref-96)
97. *Tetramorium caespitum* agg. is now recognised as comprising several species but the species present in Britain is apparently the true *caespitum*, at least on the basis of specimens from southern England (Schlick-Steiner *et al.*, 2006). [↑](#footnote-ref-97)
98. Records from Ireland may relate to *debile* (Fox in Collins & Roy, 2012). [↑](#footnote-ref-98)
99. Described from a worker casually introduced to Britain. [↑](#footnote-ref-99)
100. Nomenclature follows Lelej (2002), who includes full synonymy. Only names that have been used in the British literature are included here. [↑](#footnote-ref-100)
101. The nominate subspecies occurs in England and Wales, *atra erythrocephala* Yarrow, 1954 in Ireland and the Isle Man. [↑](#footnote-ref-101)
102. Classification follows Wahis (1986, 2006). Some distribution data from Day (1988). [↑](#footnote-ref-102)
103. Taxonomy follows Agnoli (2005). [↑](#footnote-ref-103)
104. Taxonomy mostly follows van der Vecht & Fischer (1972) and Fauna Europaea (Mitroiu *et al.*, 2004). [↑](#footnote-ref-104)
105. Although this species has been referred to as *Ancistrocerus quadratus* in British faunistic works (e.g. Richards, 1980, Archer, 2003, 2014), the type of *Vespa quadrata* Panzer is lost and its identification as this species, whilst probable, is not certain (Blüthgen, 1959, van der Vecht & Fischer, 1972), therefore *claripennis* is the name most usually used for this species (e.g. in Fauna Europaea: Mitroiu *et al.*, 2004, accessed 2015). [↑](#footnote-ref-105)
106. The subspecies *hibernicus* Blüthgen, 1937 occurs in Ireland and the Outer Hebrides. [↑](#footnote-ref-106)
107. Taxonomy follows Carpenter (1996). [↑](#footnote-ref-107)
108. Recorded as *Polistes omissa* by McClenaghan (1979), who collected a single male in Co. Down; it is unknown whether a nest had been established but this southern European species is an unlikely colonist. [↑](#footnote-ref-108)
109. Nomenclature follows Carpenter & Kojima (1997) and Kimsey & Carpenter (2012). [↑](#footnote-ref-109)
110. British populations are considered to belong to the subspecies *gribodoi*. [↑](#footnote-ref-110)
